# Supplementary figures and images for: Acetylation of TIR domains in the TLR4-Mal-MyD88 complex regulates immune responses in sepsis (part 1 of 3)
Source: EMBO J. 2024 Sep 18;43(21):9. doi: 10.1038/s44318-024-00237-8 (PMC11535217; doi:10.1038/s44318-024-00237-8)

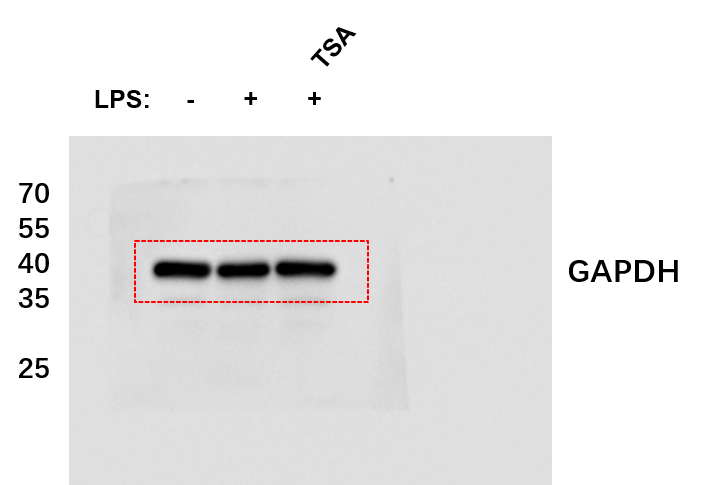

Supplement: Supplementary file 2 — Source data Fig. 1 [file 44318_2024_237_MOESM2_ESM.zip › Figure 1/Figure 1B/GAPDH.tif]

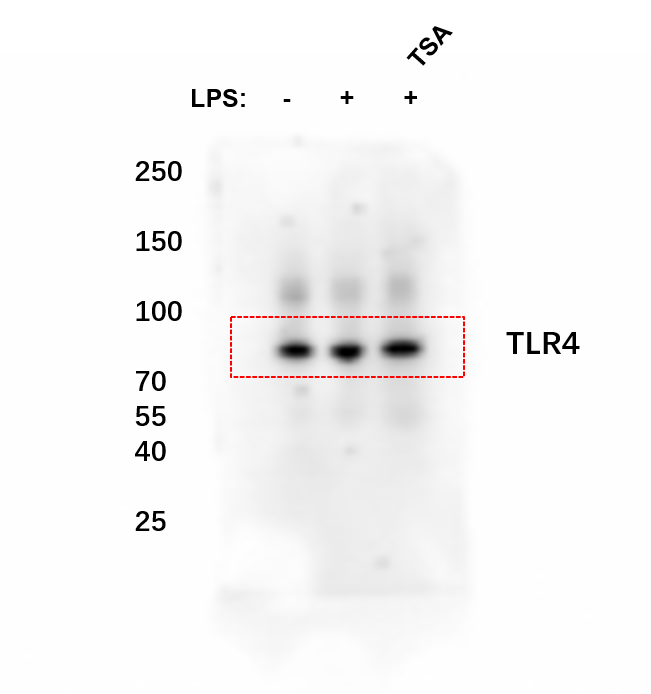

Supplement: Supplementary file 2 — Source data Fig. 1 [file 44318_2024_237_MOESM2_ESM.zip › Figure 1/Figure 1B/TLR4-.tif]

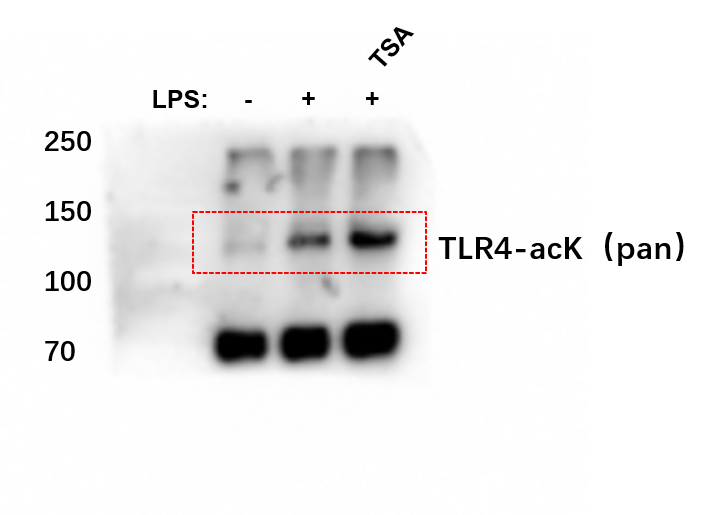

Supplement: Supplementary file 2 — Source data Fig. 1 [file 44318_2024_237_MOESM2_ESM.zip › Figure 1/Figure 1B/TLR4-acK(pan).tif]

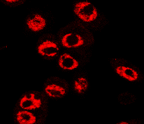

Supplement: Supplementary file 2 — Source data Fig. 1 [file 44318_2024_237_MOESM2_ESM.zip › Figure 1/Figure 1I/CBP.tif]

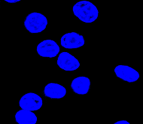

Supplement: Supplementary file 2 — Source data Fig. 1 [file 44318_2024_237_MOESM2_ESM.zip › Figure 1/Figure 1I/DAPI.tif]

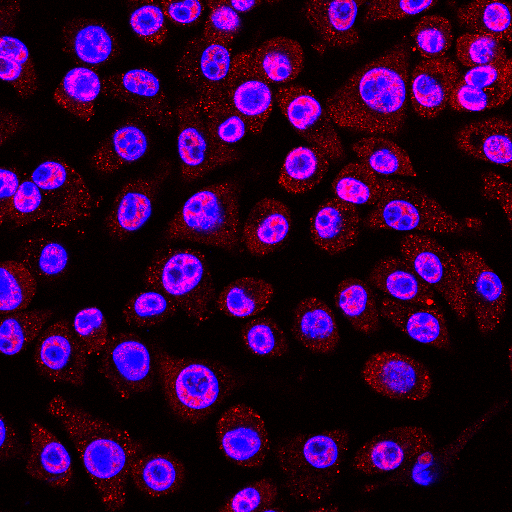

Supplement: Supplementary file 2 — Source data Fig. 1 [file 44318_2024_237_MOESM2_ESM.zip › Figure 1/Figure 1I/lps.tif]

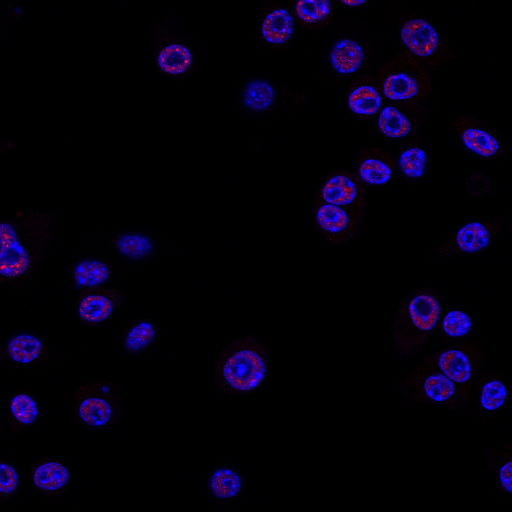

Supplement: Supplementary file 2 — Source data Fig. 1 [file 44318_2024_237_MOESM2_ESM.zip › Figure 1/Figure 1I/-lps.tif]

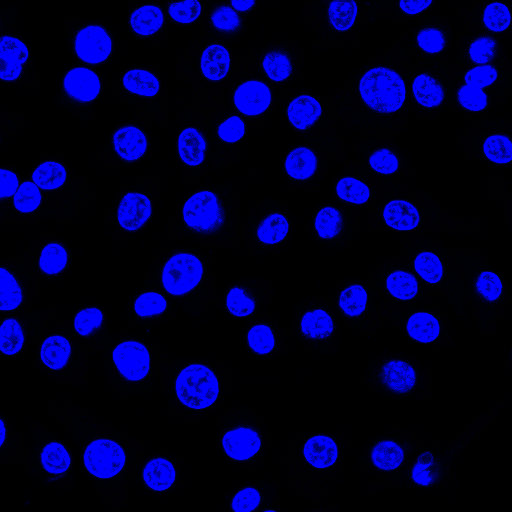

Supplement: Supplementary file 2 — Source data Fig. 1 [file 44318_2024_237_MOESM2_ESM.zip › Figure 1/Figure 1I/lps_ch00.tif]

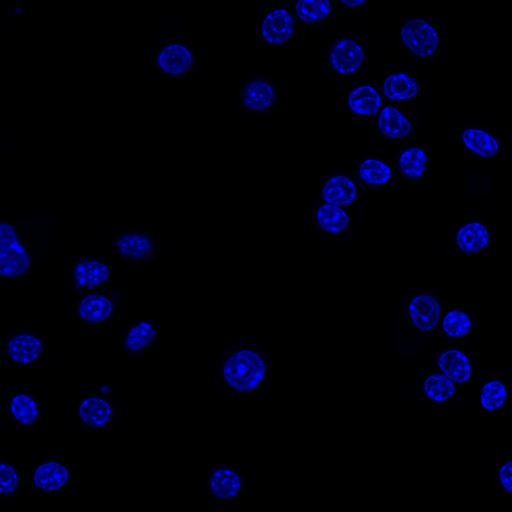

Supplement: Supplementary file 2 — Source data Fig. 1 [file 44318_2024_237_MOESM2_ESM.zip › Figure 1/Figure 1I/-lps_ch00.tif]

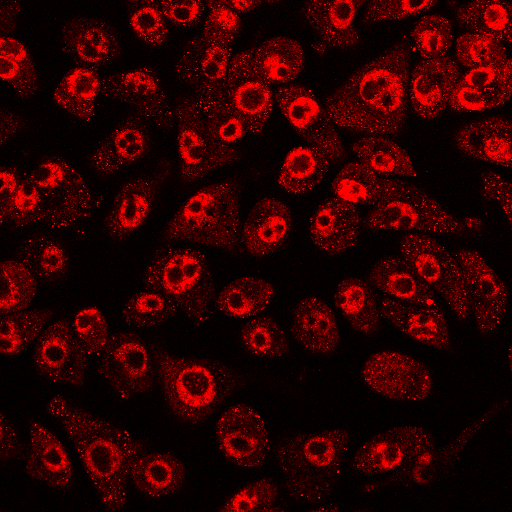

Supplement: Supplementary file 2 — Source data Fig. 1 [file 44318_2024_237_MOESM2_ESM.zip › Figure 1/Figure 1I/lps_ch01.tif]

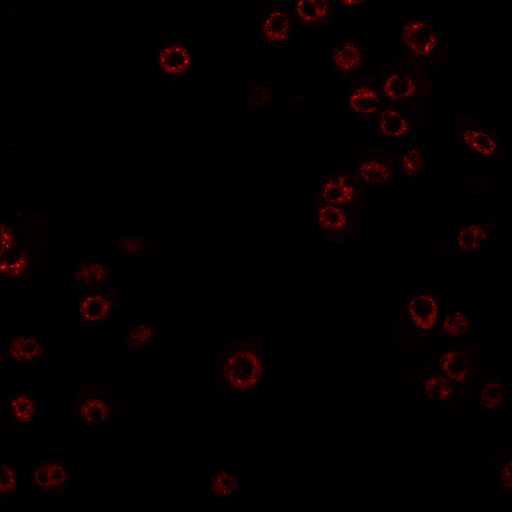

Supplement: Supplementary file 2 — Source data Fig. 1 [file 44318_2024_237_MOESM2_ESM.zip › Figure 1/Figure 1I/-lps_ch01.tif]

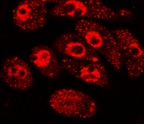

Supplement: Supplementary file 2 — Source data Fig. 1 [file 44318_2024_237_MOESM2_ESM.zip › Figure 1/Figure 1I/LPS+CBP.tif]

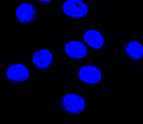

Supplement: Supplementary file 2 — Source data Fig. 1 [file 44318_2024_237_MOESM2_ESM.zip › Figure 1/Figure 1I/LPS+DAPI.tif]

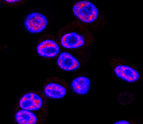

Supplement: Supplementary file 2 — Source data Fig. 1 [file 44318_2024_237_MOESM2_ESM.zip › Figure 1/Figure 1I/MERGE.tif]

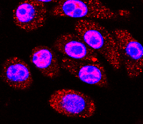

Supplement: Supplementary file 2 — Source data Fig. 1 [file 44318_2024_237_MOESM2_ESM.zip › Figure 1/Figure 1I/merge+LPS.tif]

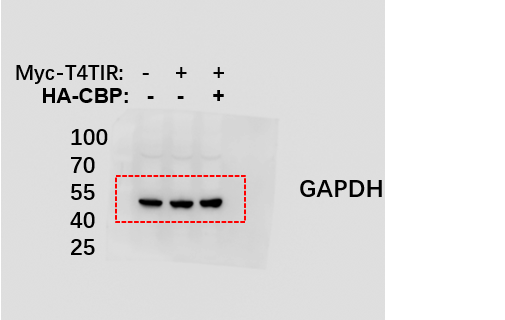

Supplement: Supplementary file 2 — Source data Fig. 1 [file 44318_2024_237_MOESM2_ESM.zip › Figure 1/Figure 1J/GAPDH.tif]

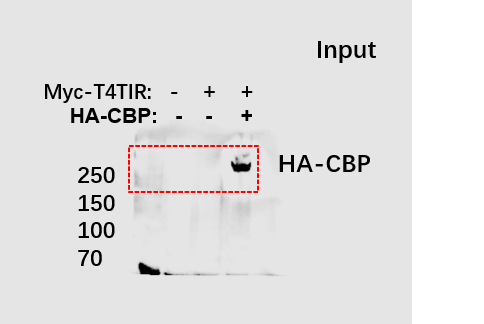

Supplement: Supplementary file 2 — Source data Fig. 1 [file 44318_2024_237_MOESM2_ESM.zip › Figure 1/Figure 1J/HA-CBP.tif]

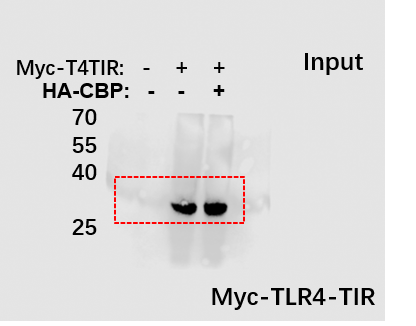

Supplement: Supplementary file 2 — Source data Fig. 1 [file 44318_2024_237_MOESM2_ESM.zip › Figure 1/Figure 1J/Myc-TLR4-TIR input.tif]

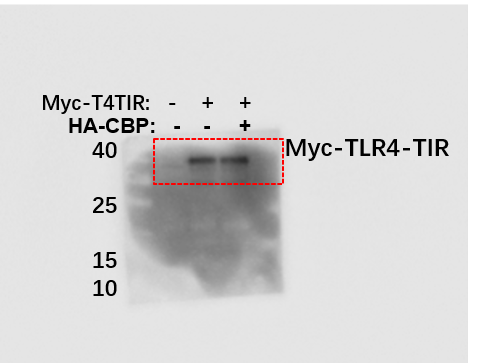

Supplement: Supplementary file 2 — Source data Fig. 1 [file 44318_2024_237_MOESM2_ESM.zip › Figure 1/Figure 1J/Myc-TLR4-TIR.tif]

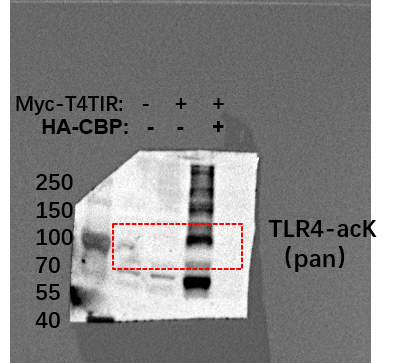

Supplement: Supplementary file 2 — Source data Fig. 1 [file 44318_2024_237_MOESM2_ESM.zip › Figure 1/Figure 1J/TLR4-acK(pan).tif]

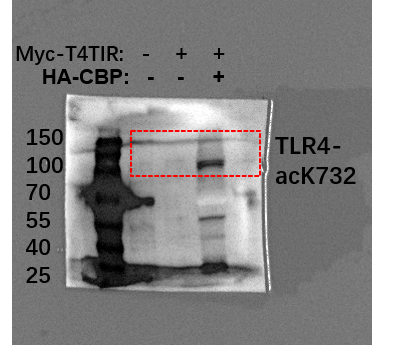

Supplement: Supplementary file 2 — Source data Fig. 1 [file 44318_2024_237_MOESM2_ESM.zip › Figure 1/Figure 1J/TLR4-acK732.tif]

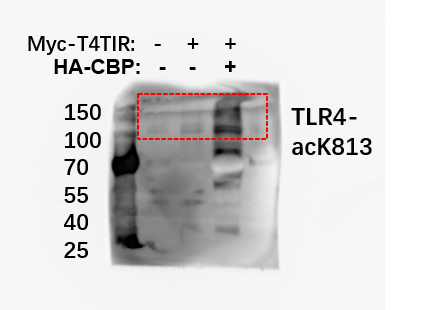

Supplement: Supplementary file 2 — Source data Fig. 1 [file 44318_2024_237_MOESM2_ESM.zip › Figure 1/Figure 1J/TLR4-acK813.tif]

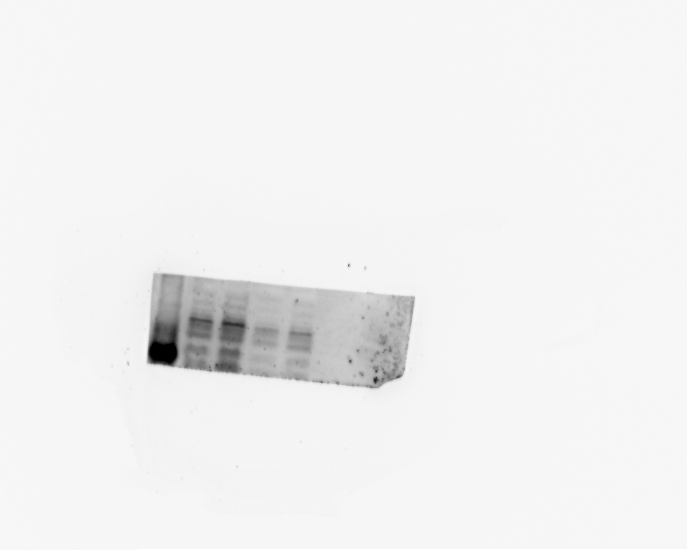

Supplement: Supplementary file 2 — Source data Fig. 1 [file 44318_2024_237_MOESM2_ESM.zip › Figure 1/Figure 1L/[╘¡╩╝]/FAN2-3.tif]

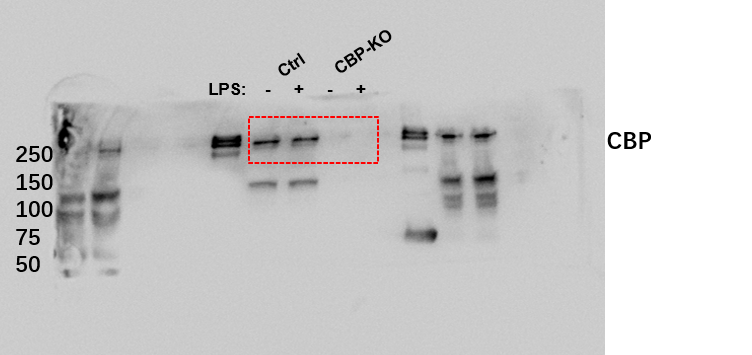

Supplement: Supplementary file 2 — Source data Fig. 1 [file 44318_2024_237_MOESM2_ESM.zip › Figure 1/Figure 1L/CBP-1.tif]

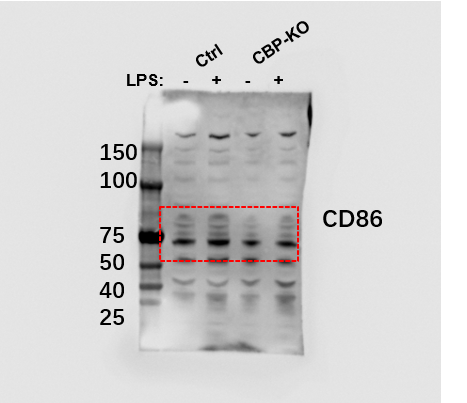

Supplement: Supplementary file 2 — Source data Fig. 1 [file 44318_2024_237_MOESM2_ESM.zip › Figure 1/Figure 1L/CD86-1.tif]

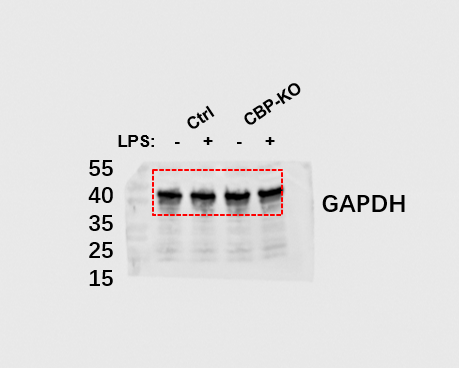

Supplement: Supplementary file 2 — Source data Fig. 1 [file 44318_2024_237_MOESM2_ESM.zip › Figure 1/Figure 1L/GAPDH-1.tif]

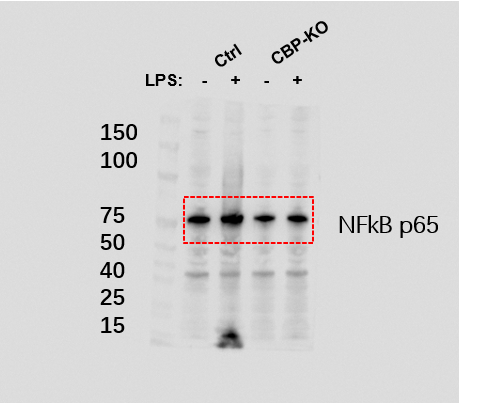

Supplement: Supplementary file 2 — Source data Fig. 1 [file 44318_2024_237_MOESM2_ESM.zip › Figure 1/Figure 1L/NFKB p65-1.tif]

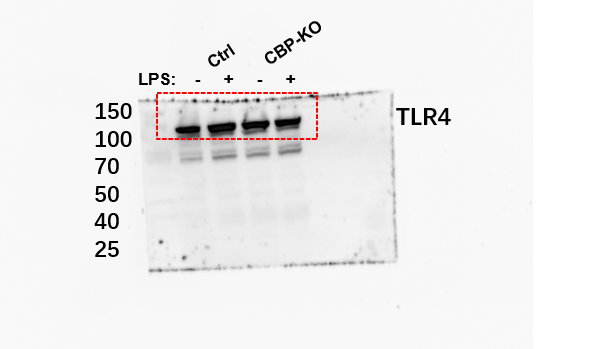

Supplement: Supplementary file 2 — Source data Fig. 1 [file 44318_2024_237_MOESM2_ESM.zip › Figure 1/Figure 1L/TLR4.tif]

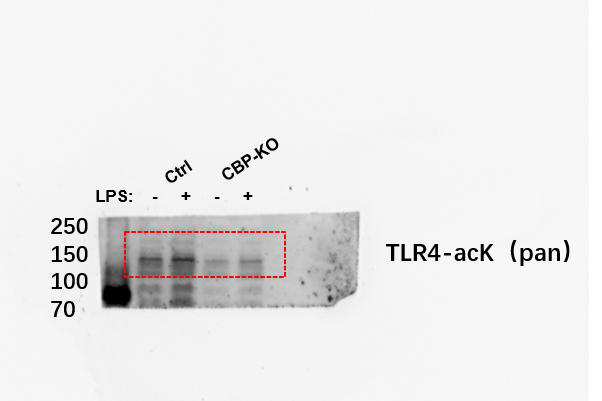

Supplement: Supplementary file 2 — Source data Fig. 1 [file 44318_2024_237_MOESM2_ESM.zip › Figure 1/Figure 1L/TLR4-acK pan.tif]

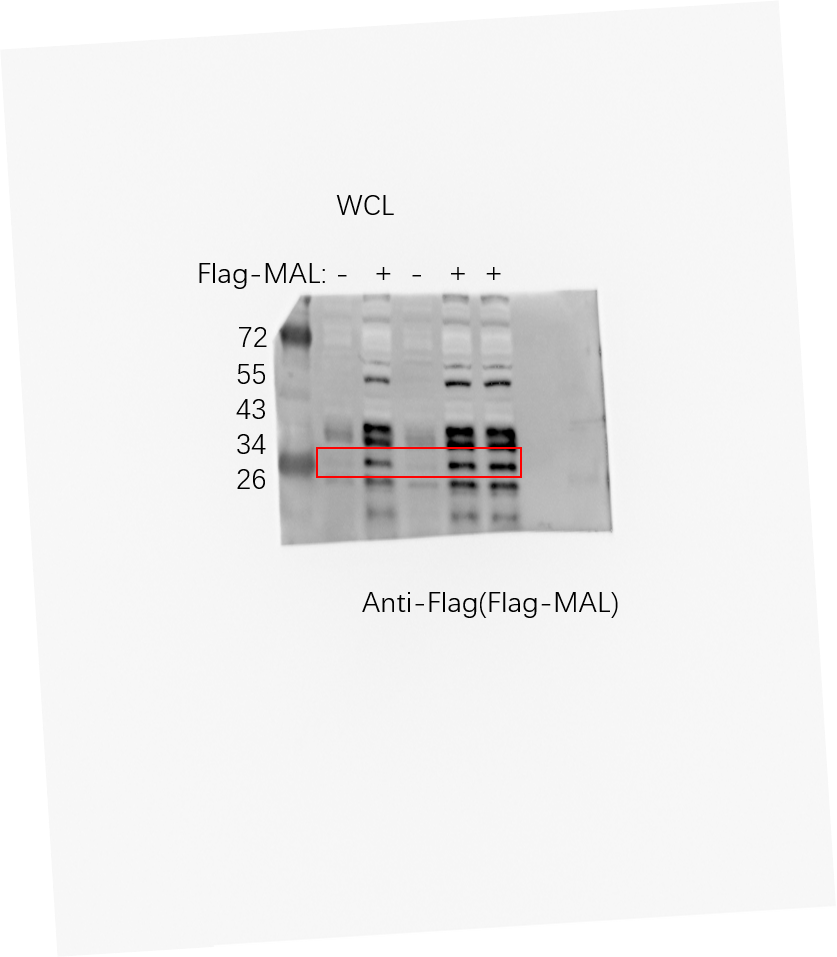

Supplement: Supplementary file 3 — Source data Fig. 2 [file 44318_2024_237_MOESM3_ESM.zip › Figure 2/Figure2A/anti-flag.tif]

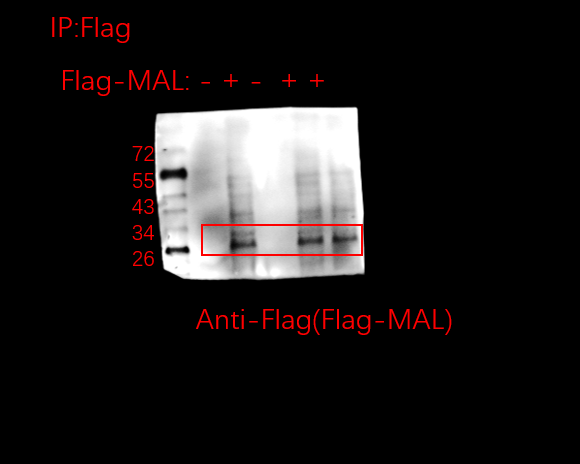

Supplement: Supplementary file 3 — Source data Fig. 2 [file 44318_2024_237_MOESM3_ESM.zip › Figure 2/Figure2A/IP anti-falg.tif]

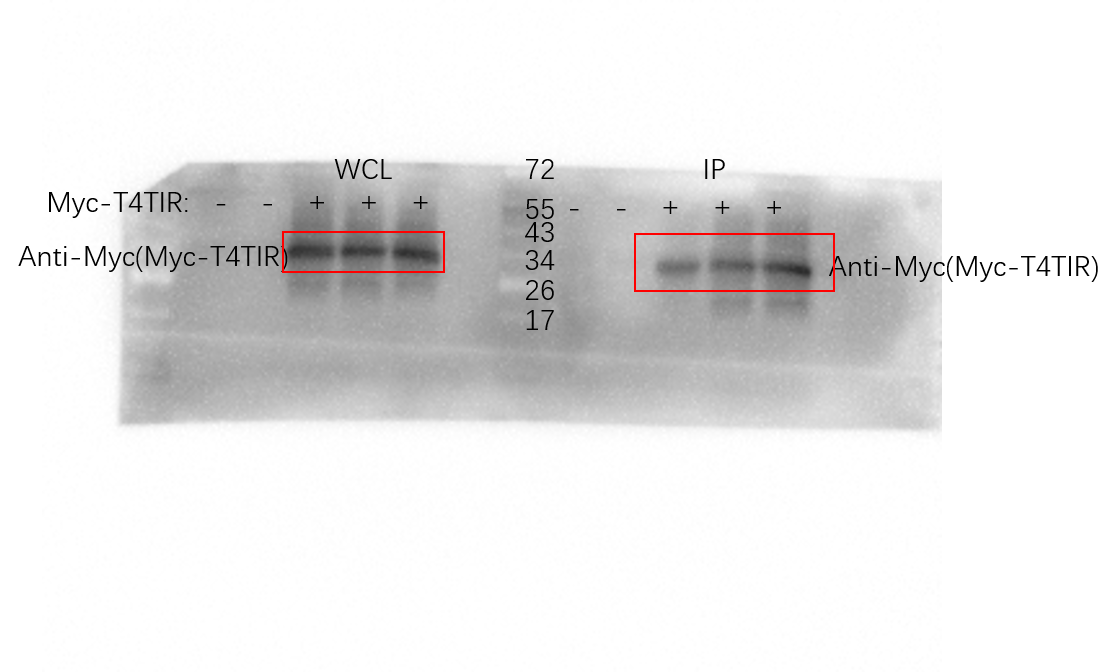

Supplement: Supplementary file 3 — Source data Fig. 2 [file 44318_2024_237_MOESM3_ESM.zip › Figure 2/Figure2A/myc2.tif]

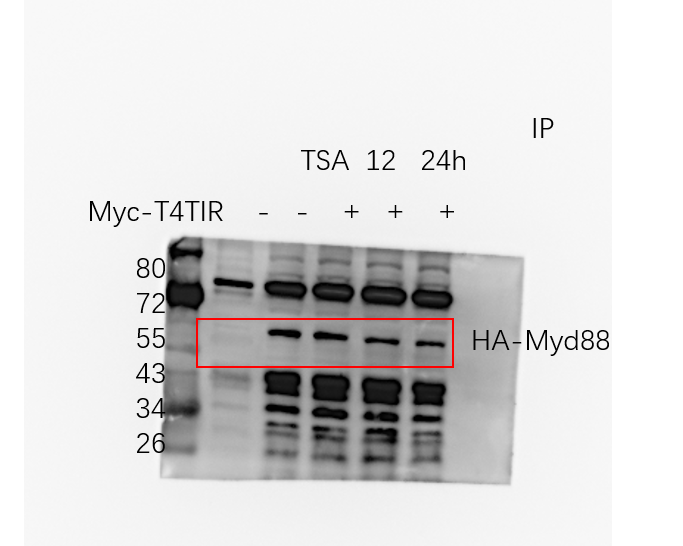

Supplement: Supplementary file 3 — Source data Fig. 2 [file 44318_2024_237_MOESM3_ESM.zip › Figure 2/Figure2B/IP HA-Myd88.tif]

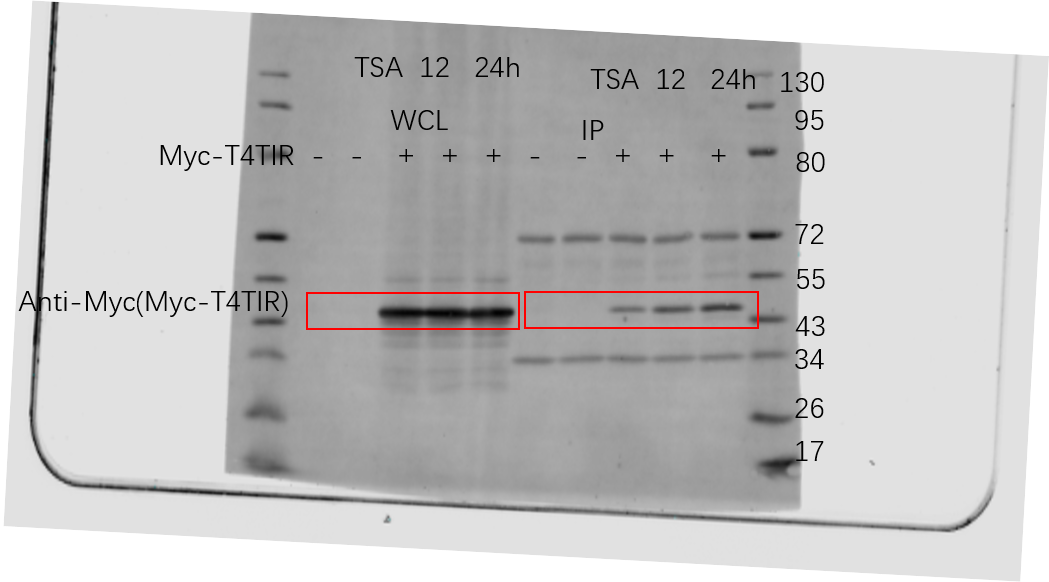

Supplement: Supplementary file 3 — Source data Fig. 2 [file 44318_2024_237_MOESM3_ESM.zip › Figure 2/Figure2B/myc-T4TIR.tif]

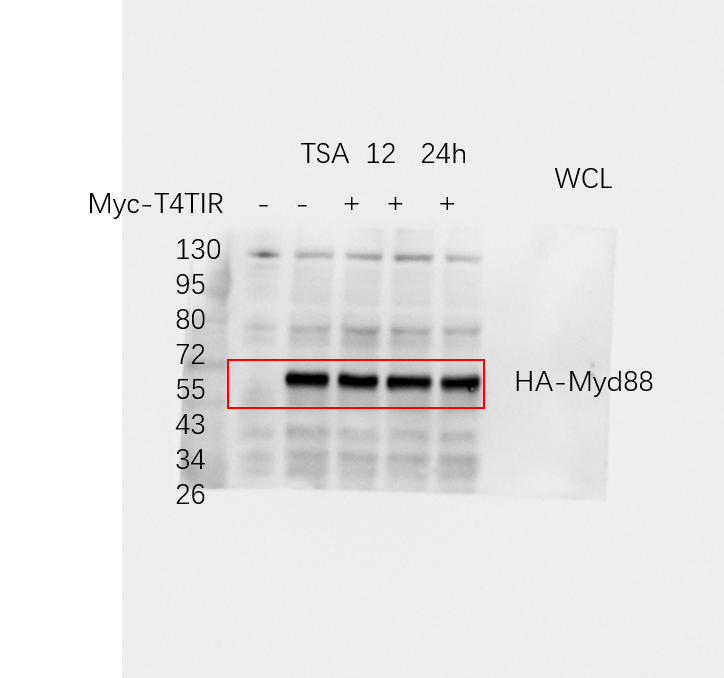

Supplement: Supplementary file 3 — Source data Fig. 2 [file 44318_2024_237_MOESM3_ESM.zip › Figure 2/Figure2B/WCL HA-Myd88.tif]

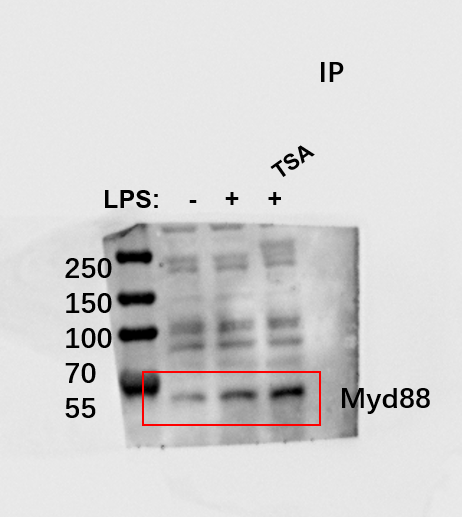

Supplement: Supplementary file 3 — Source data Fig. 2 [file 44318_2024_237_MOESM3_ESM.zip › Figure 2/Figure2C/IP myd88.tif]

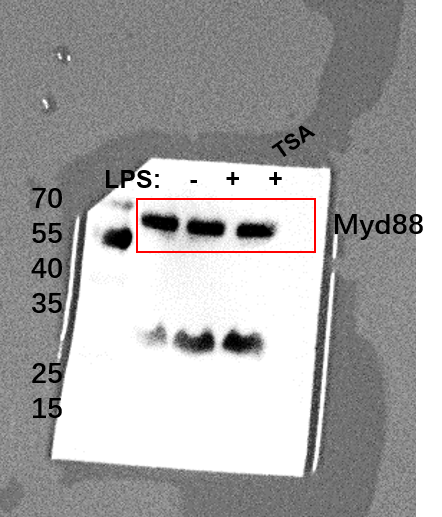

Supplement: Supplementary file 3 — Source data Fig. 2 [file 44318_2024_237_MOESM3_ESM.zip › Figure 2/Figure2C/Myd88.tif]

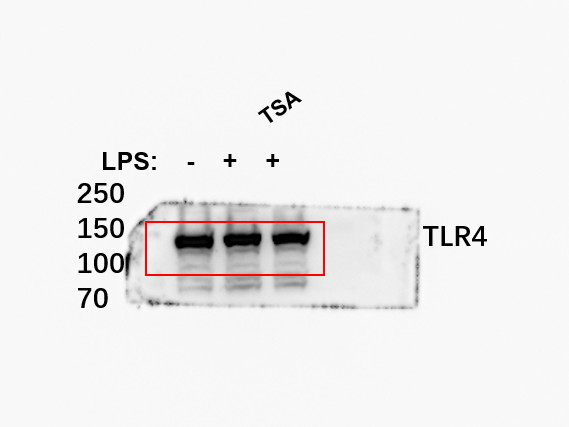

Supplement: Supplementary file 3 — Source data Fig. 2 [file 44318_2024_237_MOESM3_ESM.zip › Figure 2/Figure2C/TLR4.tif]

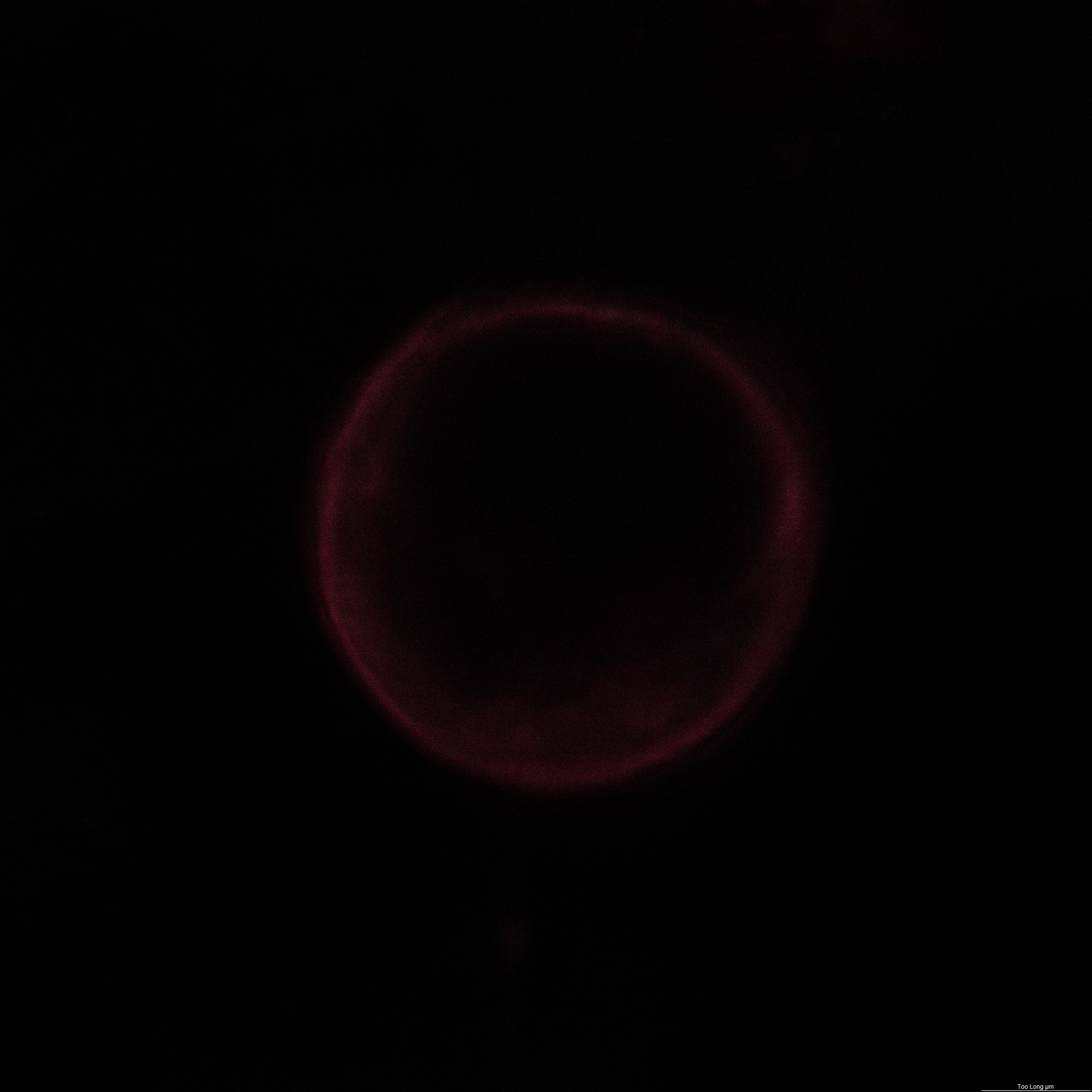

Supplement: Supplementary file 3 — Source data Fig. 2 [file 44318_2024_237_MOESM3_ESM.zip › Figure 2/Figure2D/[╘¡╩╝]/Series006_Crop001_Crop001.tif]

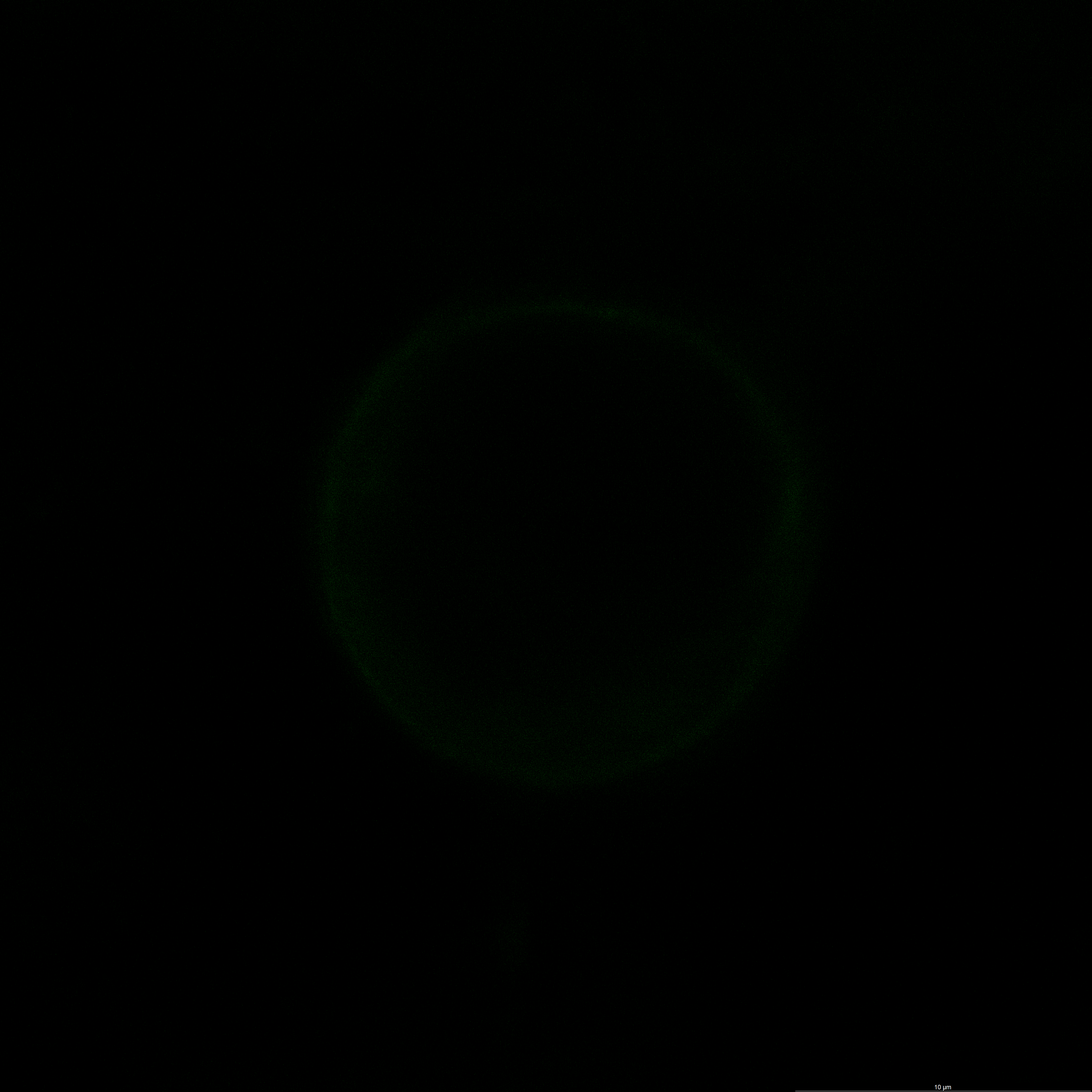

Supplement: Supplementary file 3 — Source data Fig. 2 [file 44318_2024_237_MOESM3_ESM.zip › Figure 2/Figure2D/[╘¡╩╝]/Series006_Crop001_Crop001_ch00.tif]

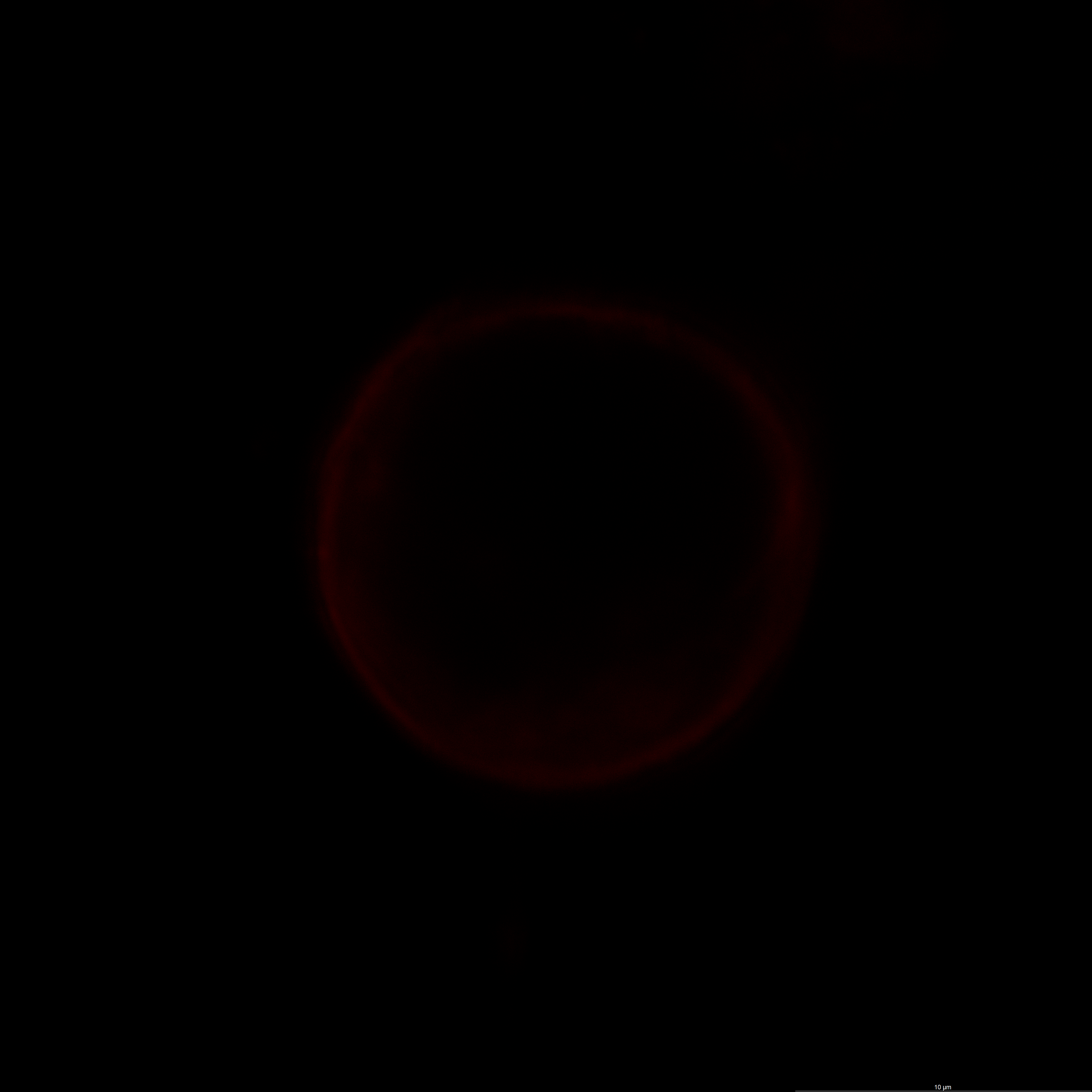

Supplement: Supplementary file 3 — Source data Fig. 2 [file 44318_2024_237_MOESM3_ESM.zip › Figure 2/Figure2D/[╘¡╩╝]/Series006_Crop001_Crop001_ch01.tif]

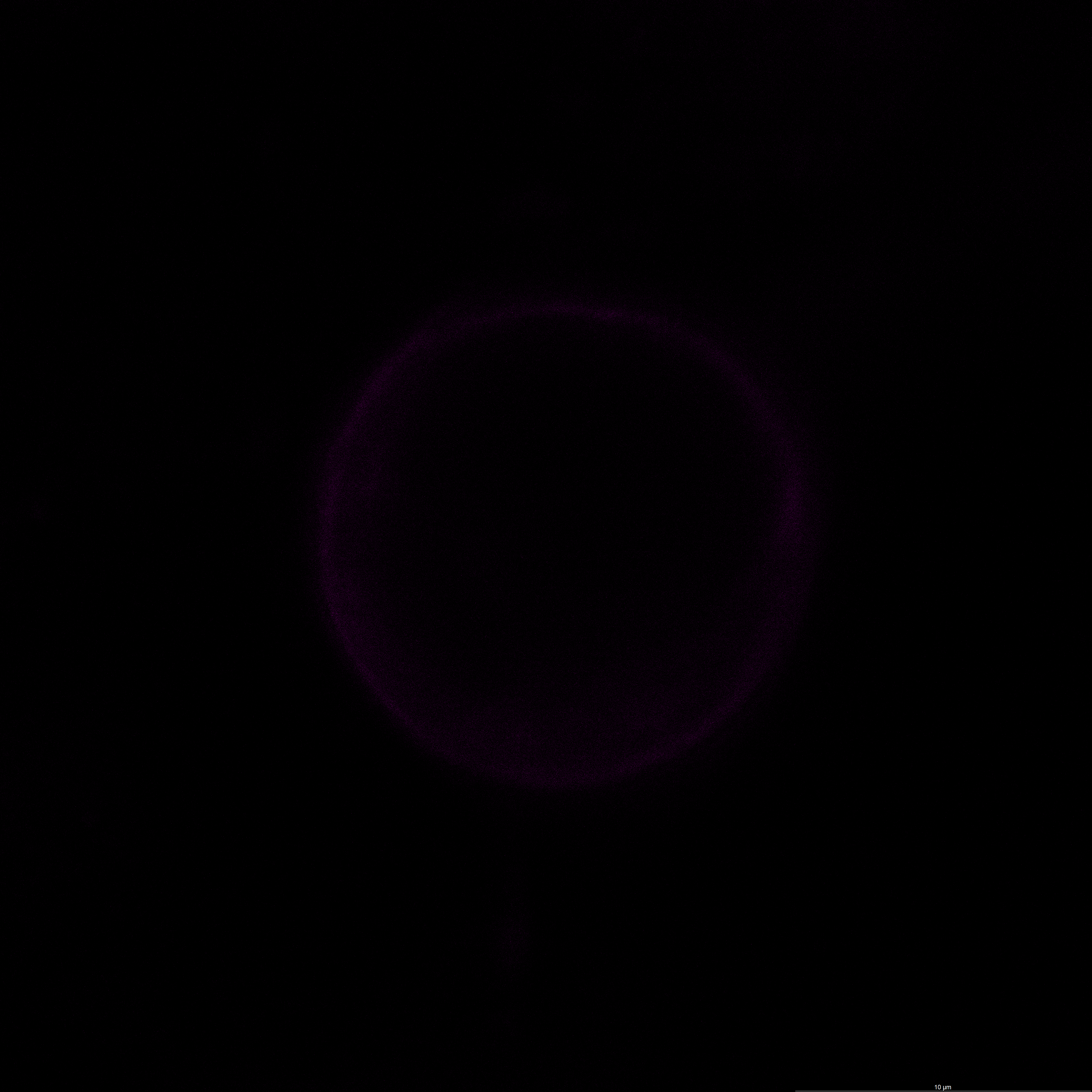

Supplement: Supplementary file 3 — Source data Fig. 2 [file 44318_2024_237_MOESM3_ESM.zip › Figure 2/Figure2D/[╘¡╩╝]/Series006_Crop001_Crop001_ch02.tif]

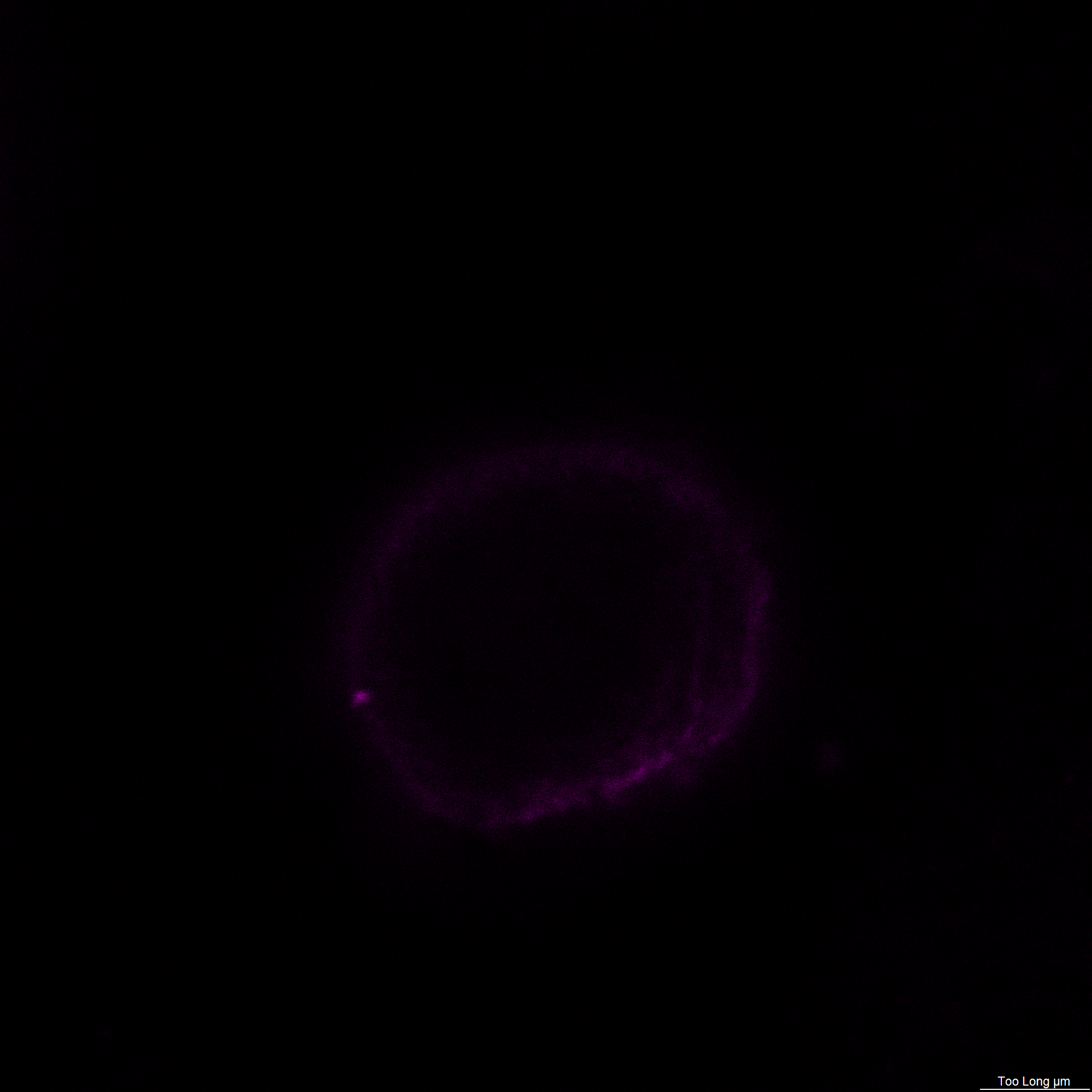

Supplement: Supplementary file 3 — Source data Fig. 2 [file 44318_2024_237_MOESM3_ESM.zip › Figure 2/Figure2D/[╘¡╩╝]/Series023_ch01.tif]

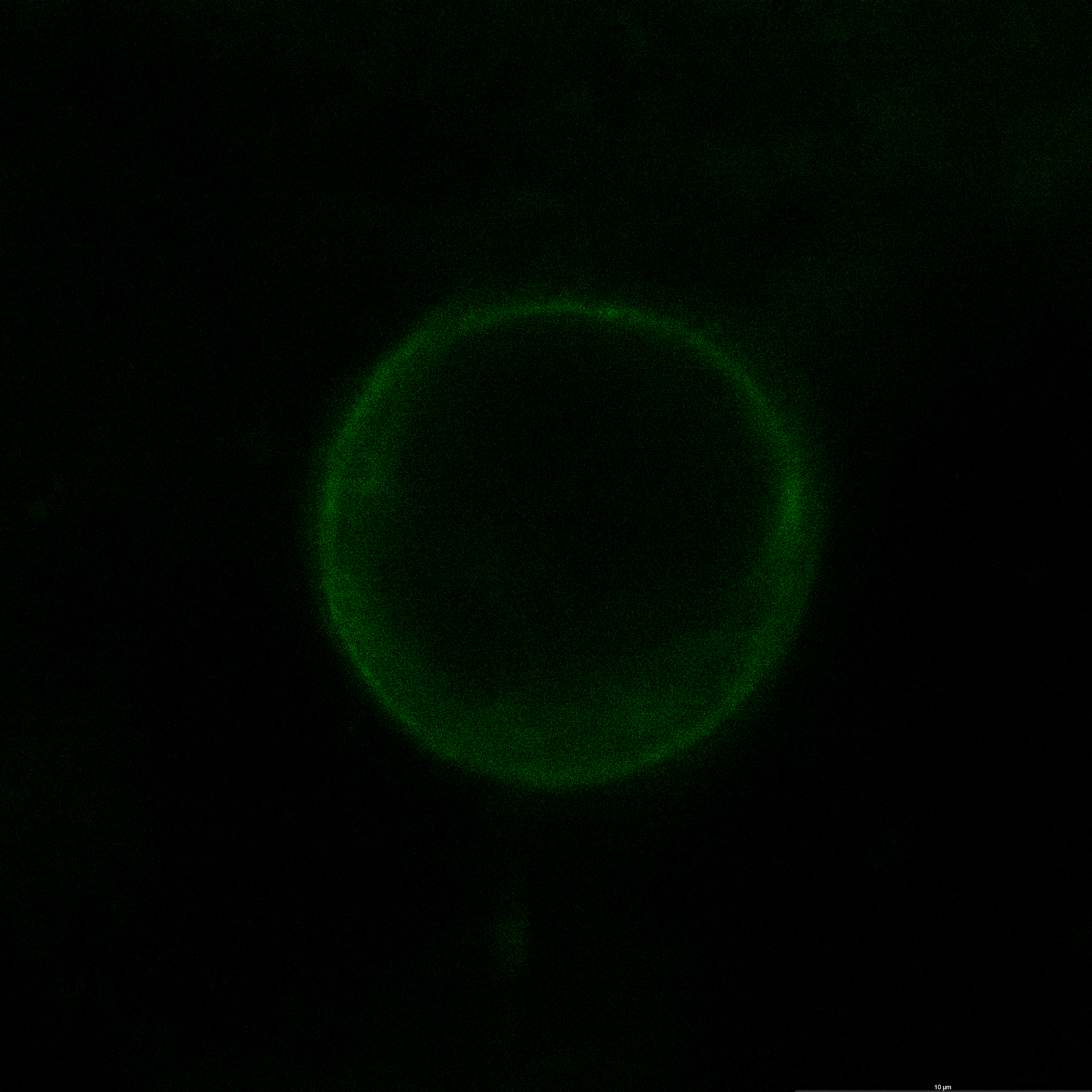

Supplement: Supplementary file 3 — Source data Fig. 2 [file 44318_2024_237_MOESM3_ESM.zip › Figure 2/Figure2D/MAL TSA.tif]

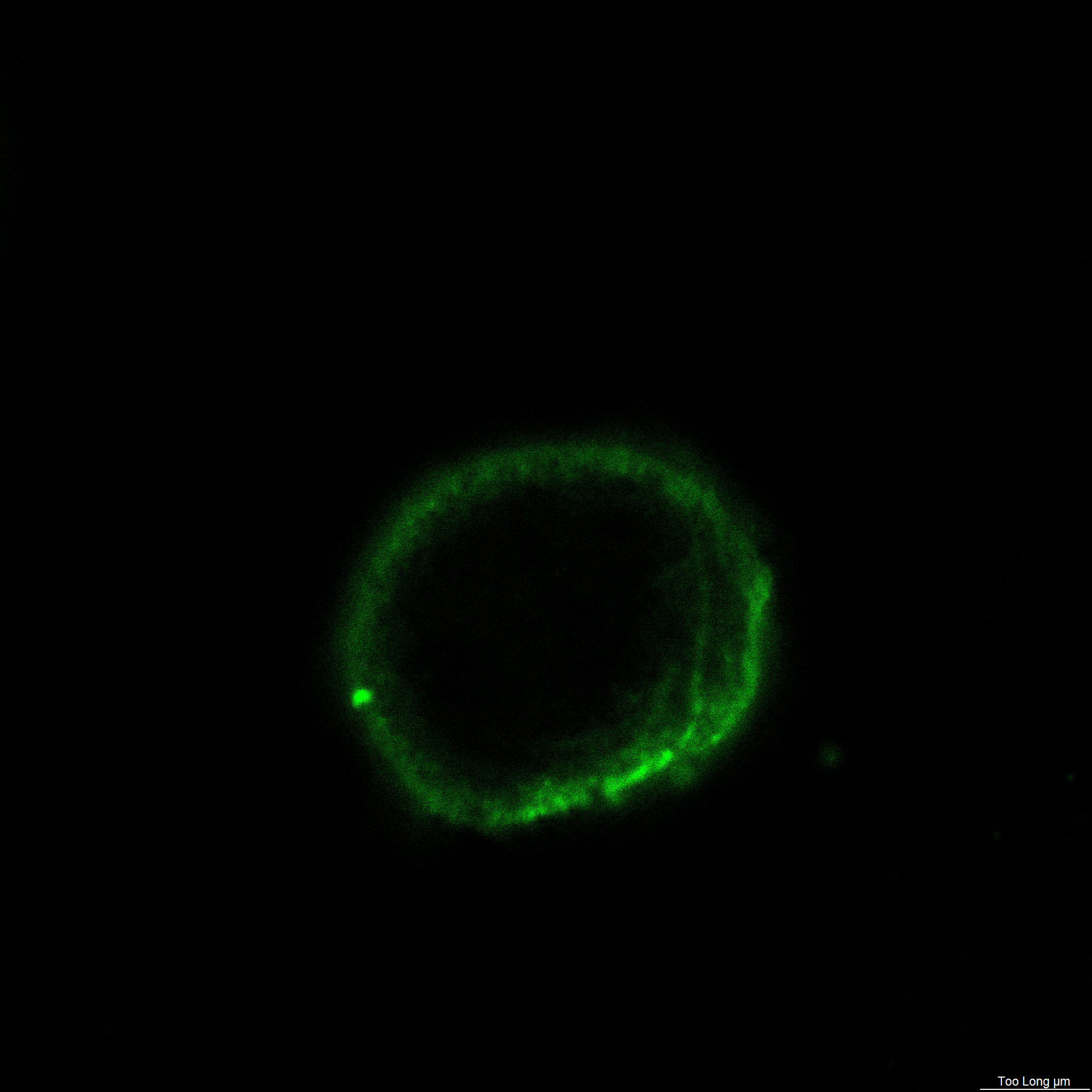

Supplement: Supplementary file 3 — Source data Fig. 2 [file 44318_2024_237_MOESM3_ESM.zip › Figure 2/Figure2D/MAL.tif]

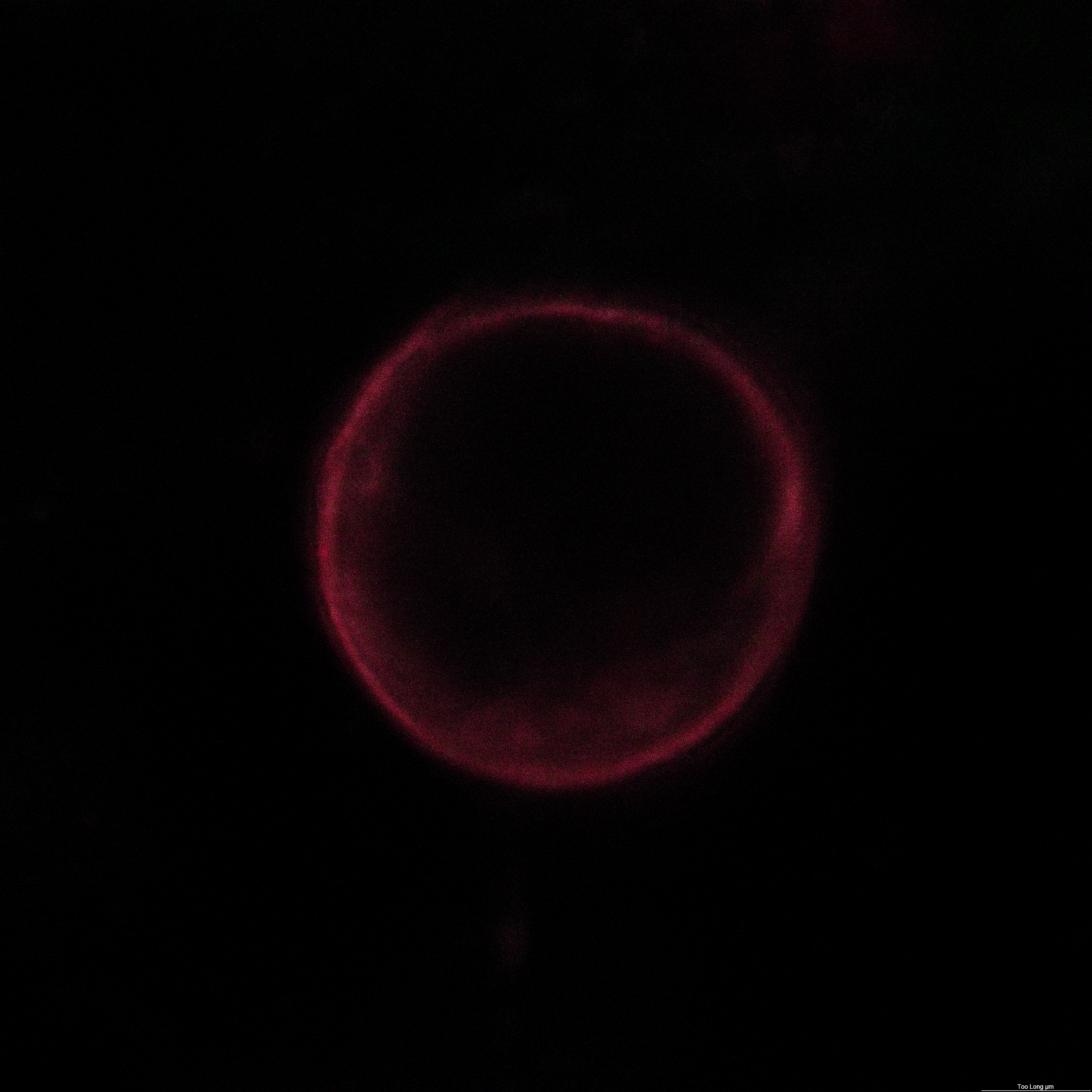

Supplement: Supplementary file 3 — Source data Fig. 2 [file 44318_2024_237_MOESM3_ESM.zip › Figure 2/Figure2D/Merge TSA.tif]

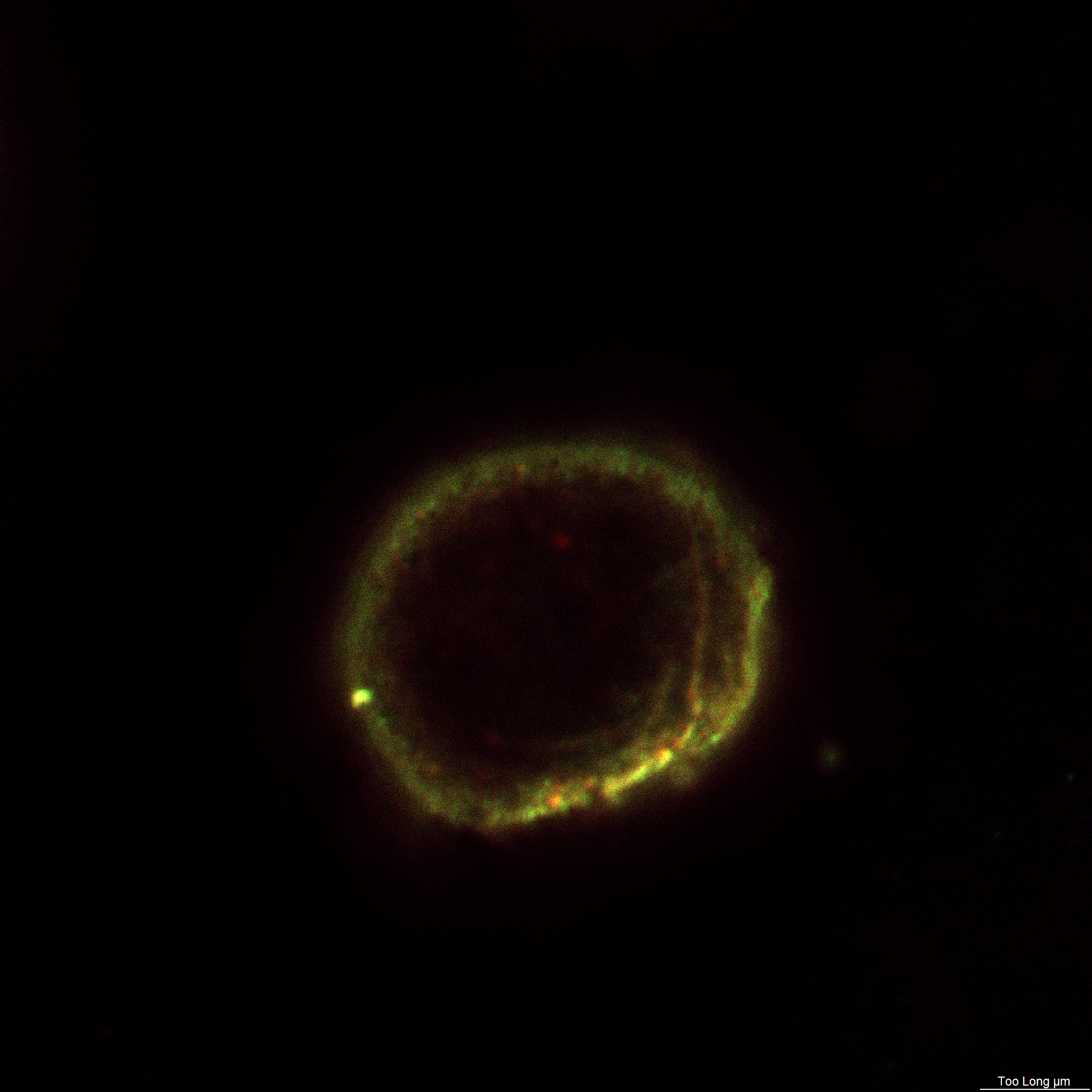

Supplement: Supplementary file 3 — Source data Fig. 2 [file 44318_2024_237_MOESM3_ESM.zip › Figure 2/Figure2D/Merge.tif]

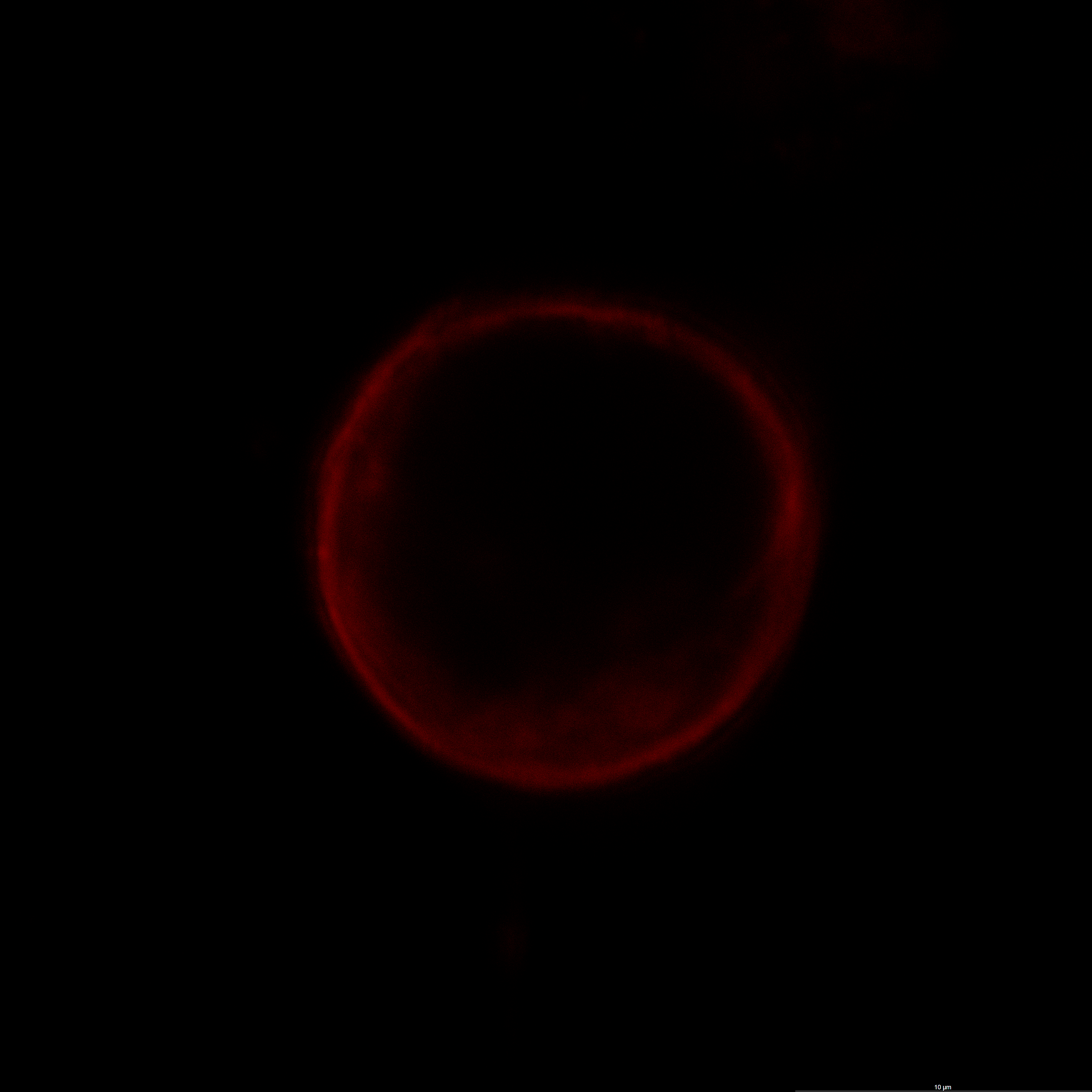

Supplement: Supplementary file 3 — Source data Fig. 2 [file 44318_2024_237_MOESM3_ESM.zip › Figure 2/Figure2D/MyD88 TSA.tif]

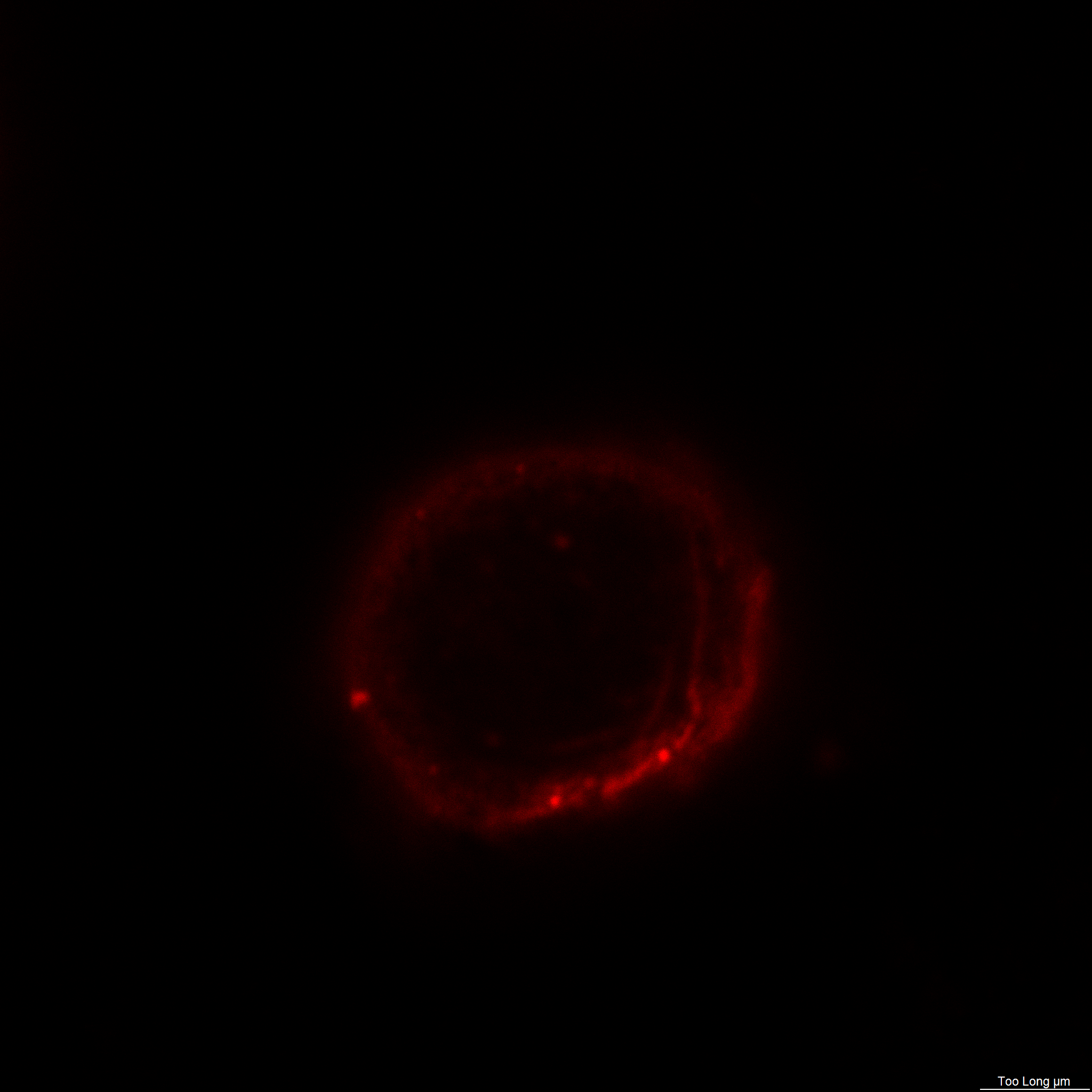

Supplement: Supplementary file 3 — Source data Fig. 2 [file 44318_2024_237_MOESM3_ESM.zip › Figure 2/Figure2D/MyD88.tif]

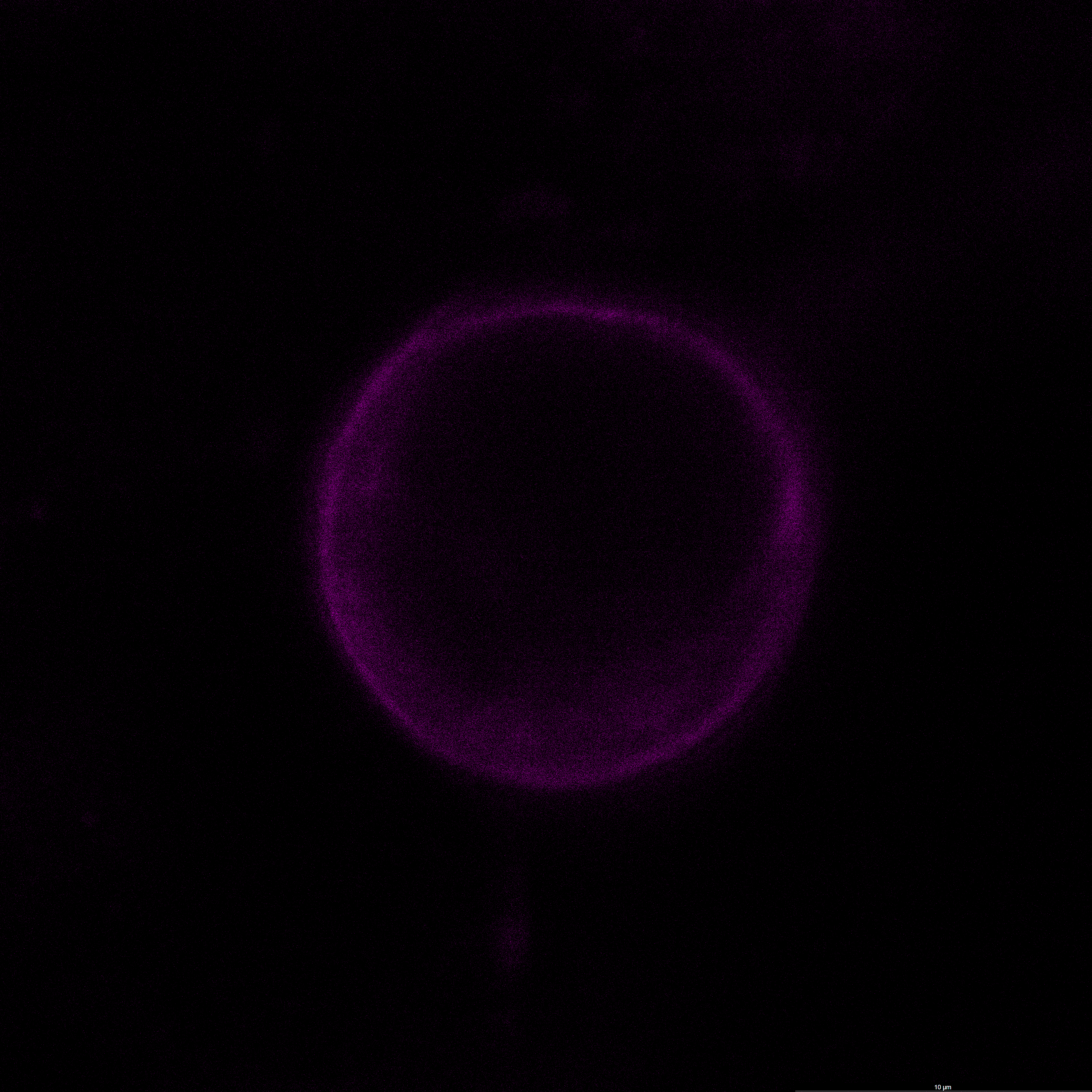

Supplement: Supplementary file 3 — Source data Fig. 2 [file 44318_2024_237_MOESM3_ESM.zip › Figure 2/Figure2D/TLR4 TSA.tif]

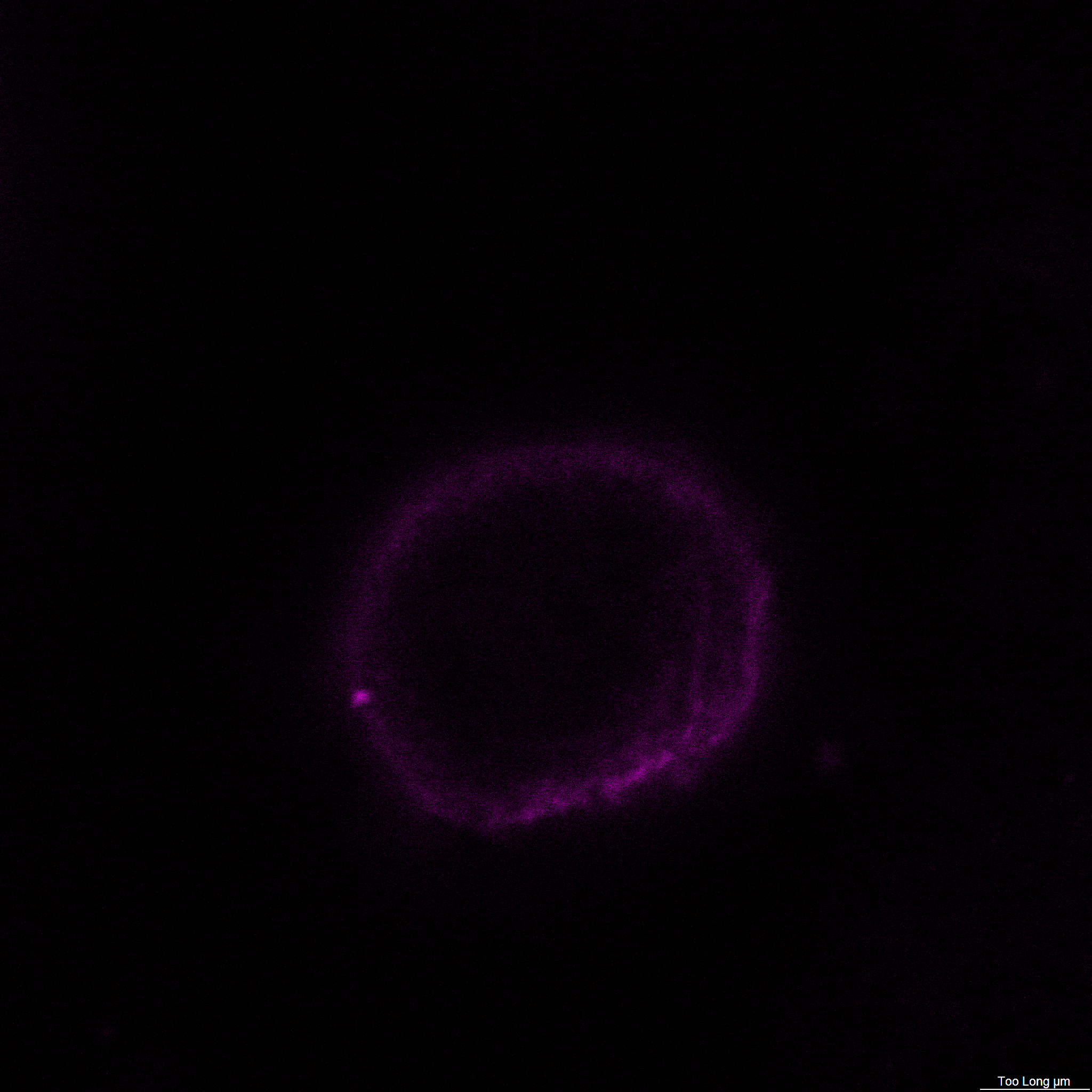

Supplement: Supplementary file 3 — Source data Fig. 2 [file 44318_2024_237_MOESM3_ESM.zip › Figure 2/Figure2D/TLR4.tif]

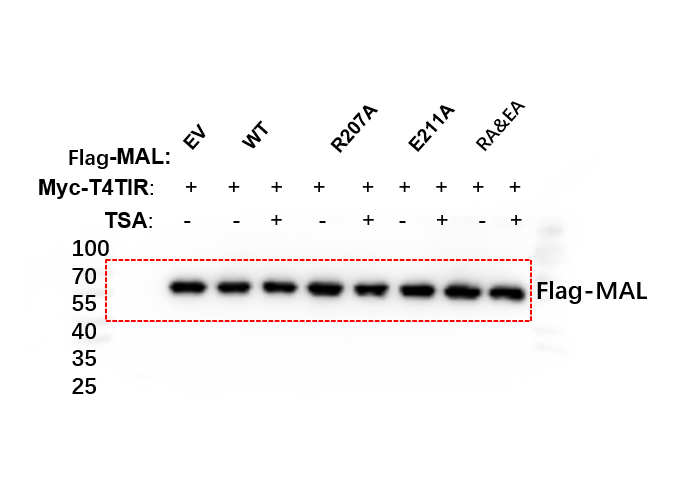

Supplement: Supplementary file 3 — Source data Fig. 2 [file 44318_2024_237_MOESM3_ESM.zip › Figure 2/Figure2H/Flag-mal.tif]

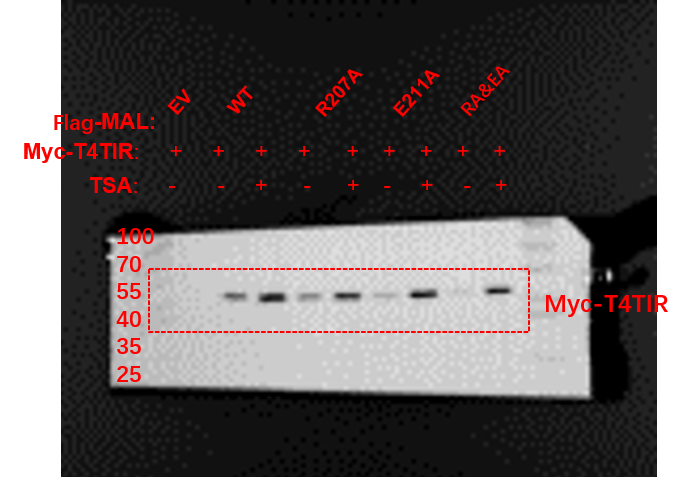

Supplement: Supplementary file 3 — Source data Fig. 2 [file 44318_2024_237_MOESM3_ESM.zip › Figure 2/Figure2H/IP Myc-T4TIR.tif]

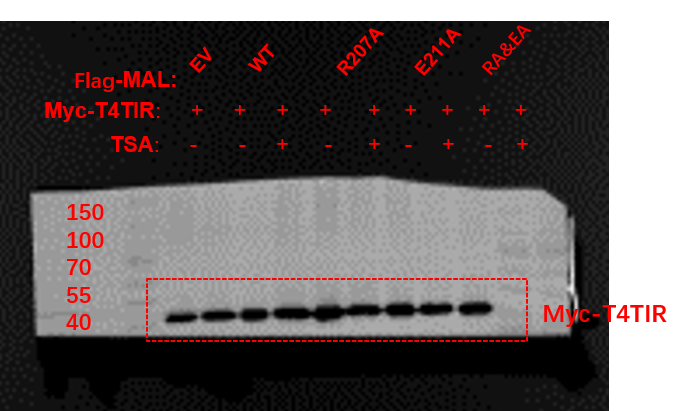

Supplement: Supplementary file 3 — Source data Fig. 2 [file 44318_2024_237_MOESM3_ESM.zip › Figure 2/Figure2H/Myc-T4TIR.tif]

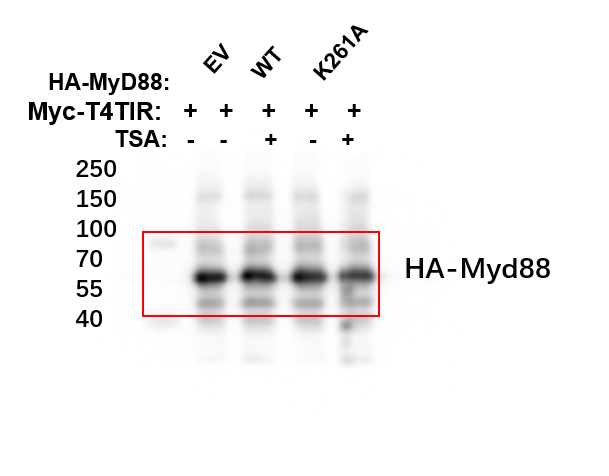

Supplement: Supplementary file 3 — Source data Fig. 2 [file 44318_2024_237_MOESM3_ESM.zip › Figure 2/Figure2I/HA-myd88.tif]

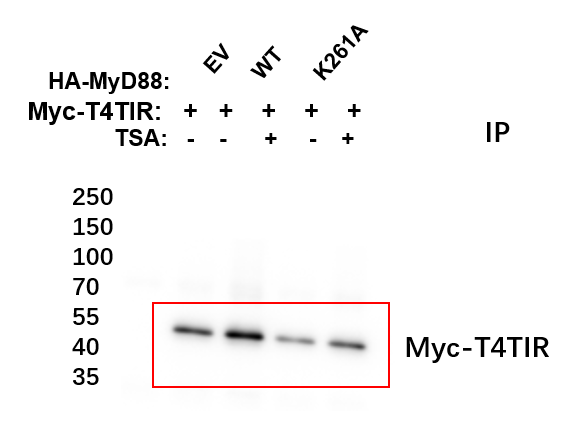

Supplement: Supplementary file 3 — Source data Fig. 2 [file 44318_2024_237_MOESM3_ESM.zip › Figure 2/Figure2I/IP Myc-T4TIR.tif]

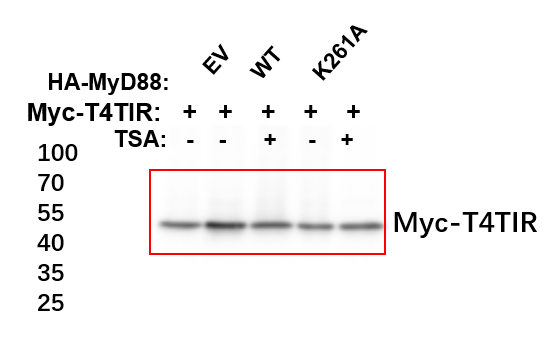

Supplement: Supplementary file 3 — Source data Fig. 2 [file 44318_2024_237_MOESM3_ESM.zip › Figure 2/Figure2I/Myc-T4TIR.tif]

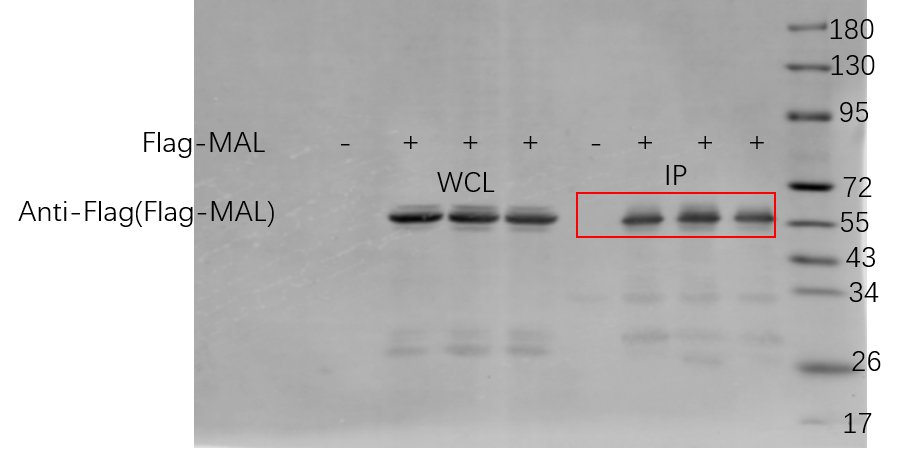

Supplement: Supplementary file 3 — Source data Fig. 2 [file 44318_2024_237_MOESM3_ESM.zip › Figure 2/Figure2J/Flag-mal.tif]

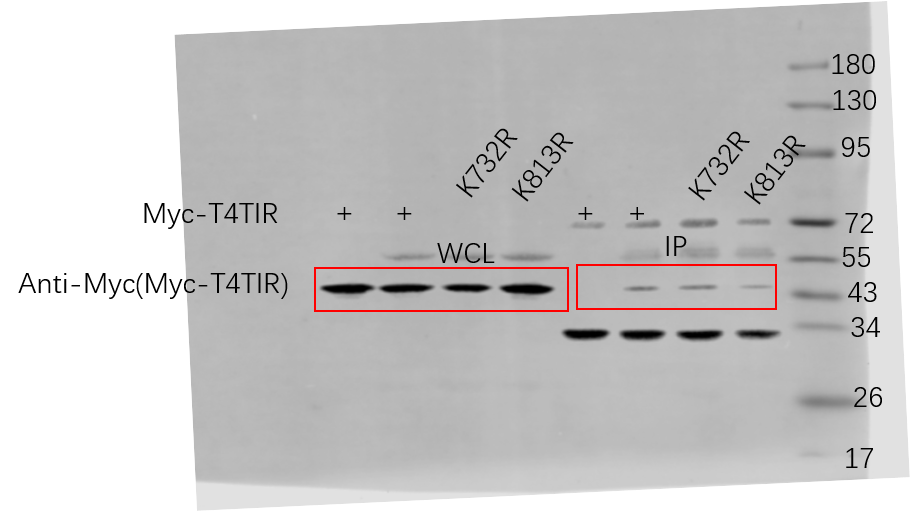

Supplement: Supplementary file 3 — Source data Fig. 2 [file 44318_2024_237_MOESM3_ESM.zip › Figure 2/Figure2J/Myc-T4TIR.tif]

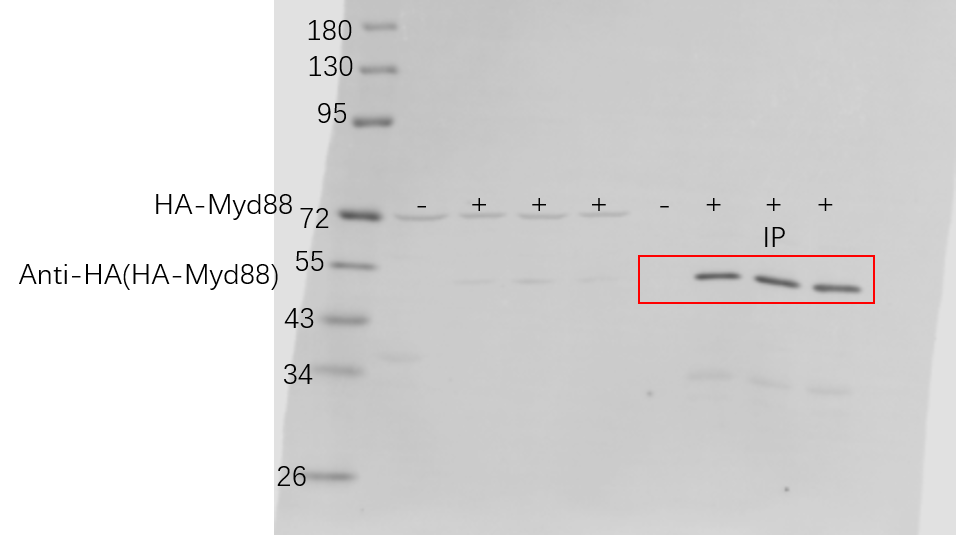

Supplement: Supplementary file 3 — Source data Fig. 2 [file 44318_2024_237_MOESM3_ESM.zip › Figure 2/Figure2K/HA-Myd88.tif]

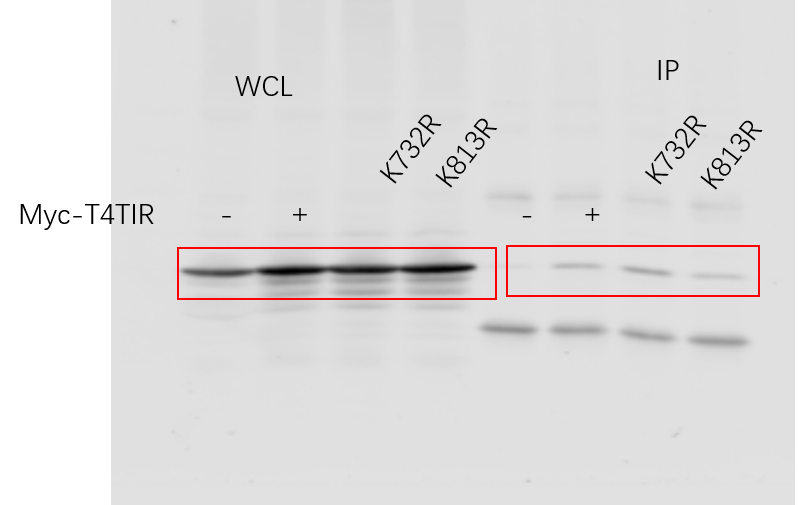

Supplement: Supplementary file 3 — Source data Fig. 2 [file 44318_2024_237_MOESM3_ESM.zip › Figure 2/Figure2K/Myc-T4TIR.tif]

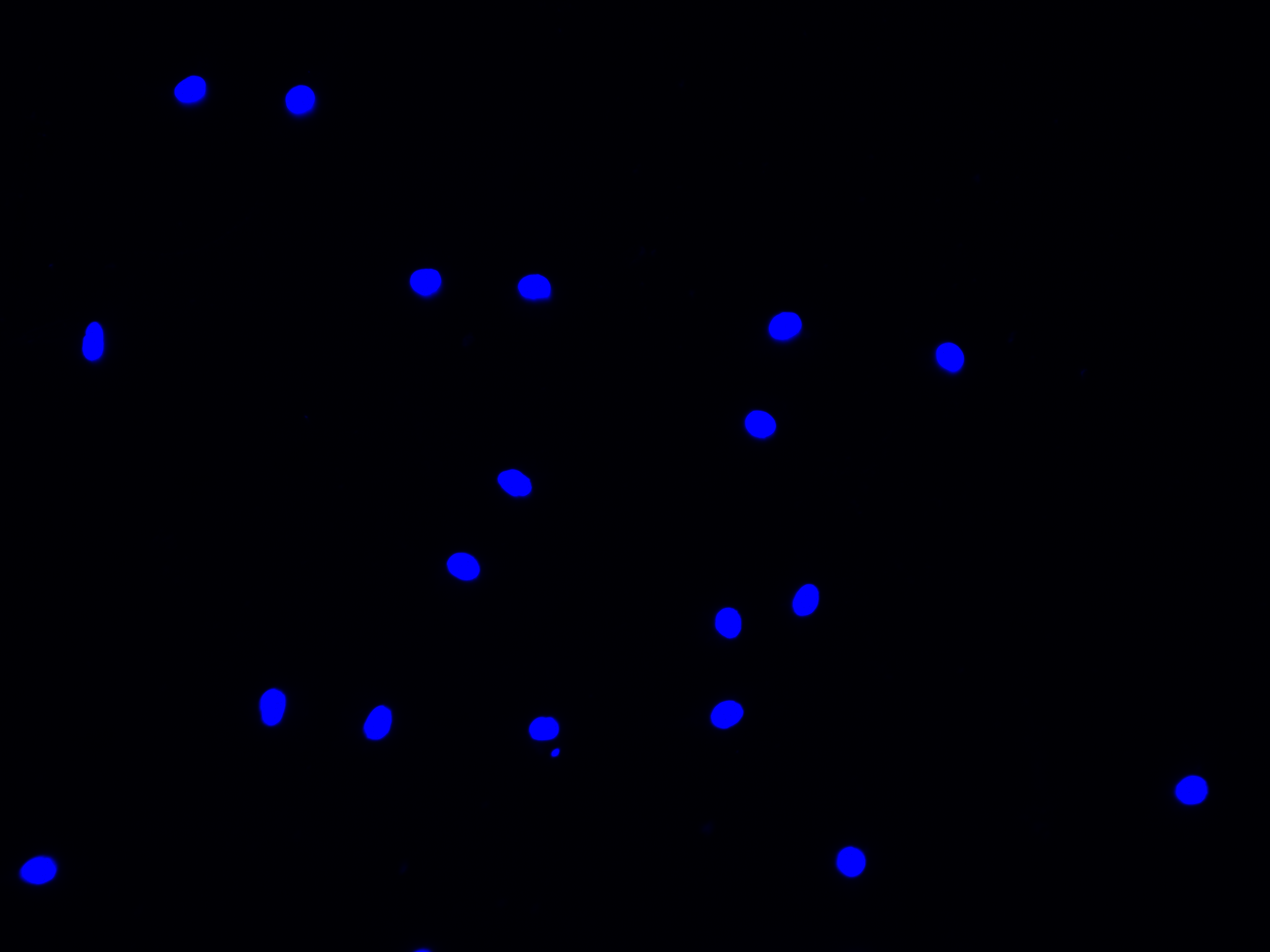

Supplement: Supplementary file 3 — Source data Fig. 2 [file 44318_2024_237_MOESM3_ESM.zip › Figure 2/Figure2L/730 lps_Bottom Slide_D_p00_0_A01f24d0.TIF]

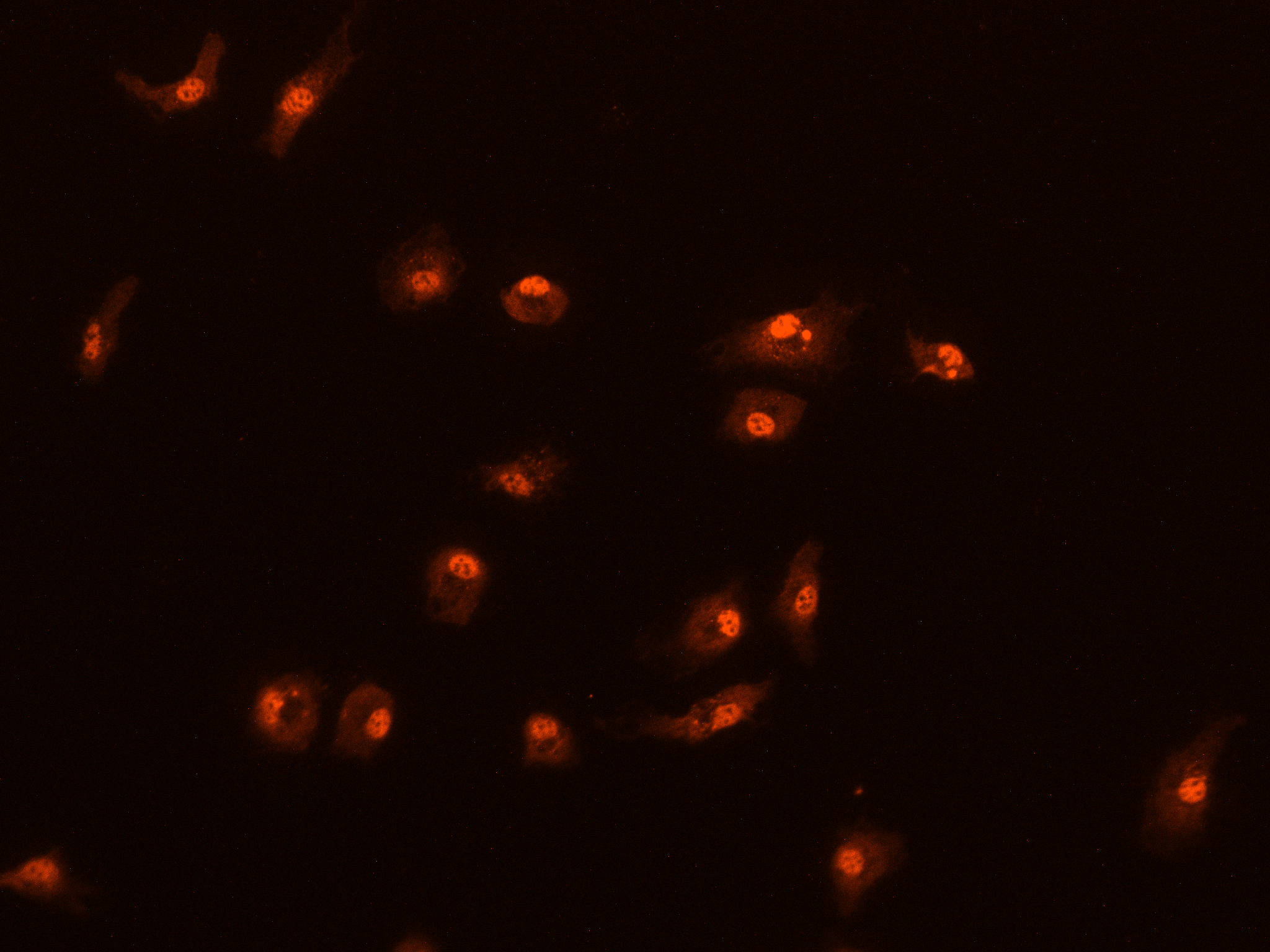

Supplement: Supplementary file 3 — Source data Fig. 2 [file 44318_2024_237_MOESM3_ESM.zip › Figure 2/Figure2L/730 lps_Bottom Slide_D_p00_0_A01f24d2.TIF]

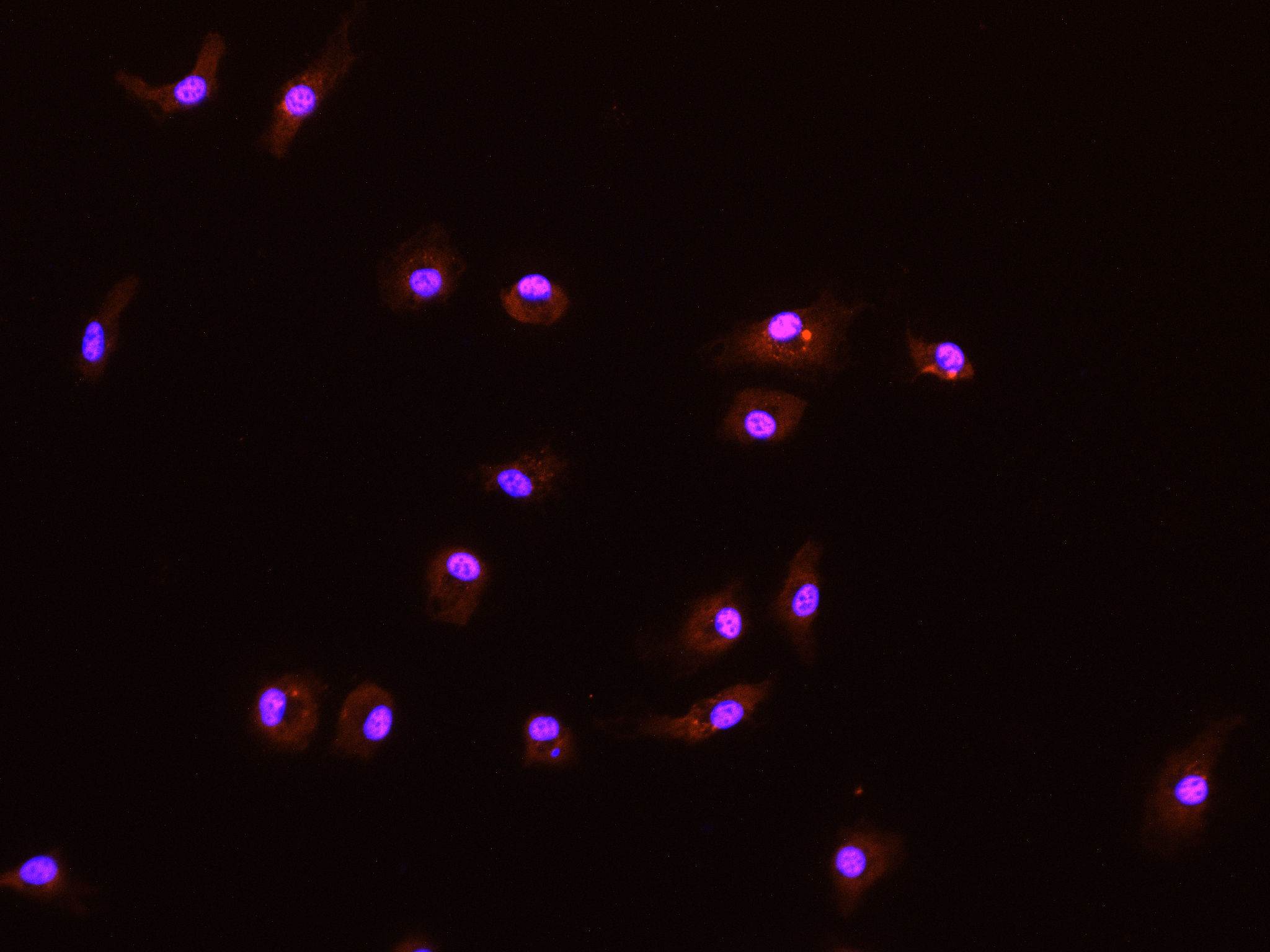

Supplement: Supplementary file 3 — Source data Fig. 2 [file 44318_2024_237_MOESM3_ESM.zip › Figure 2/Figure2L/730 lps_Bottom Slide_M_p00_0_A01f24d0.TIF]

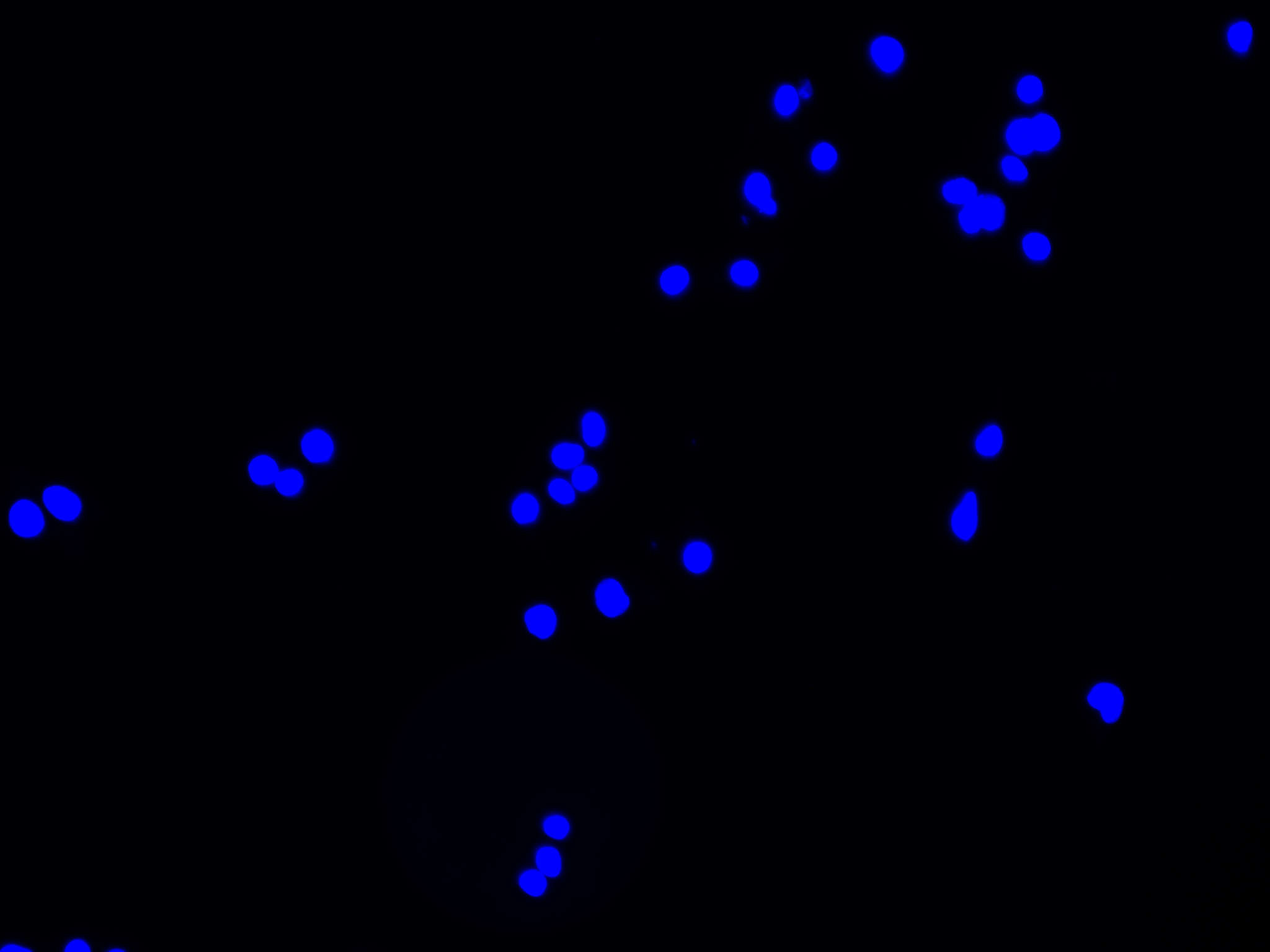

Supplement: Supplementary file 3 — Source data Fig. 2 [file 44318_2024_237_MOESM3_ESM.zip › Figure 2/Figure2L/730-lps_Bottom Slide_D_p01_0_A01f16d0.TIF]

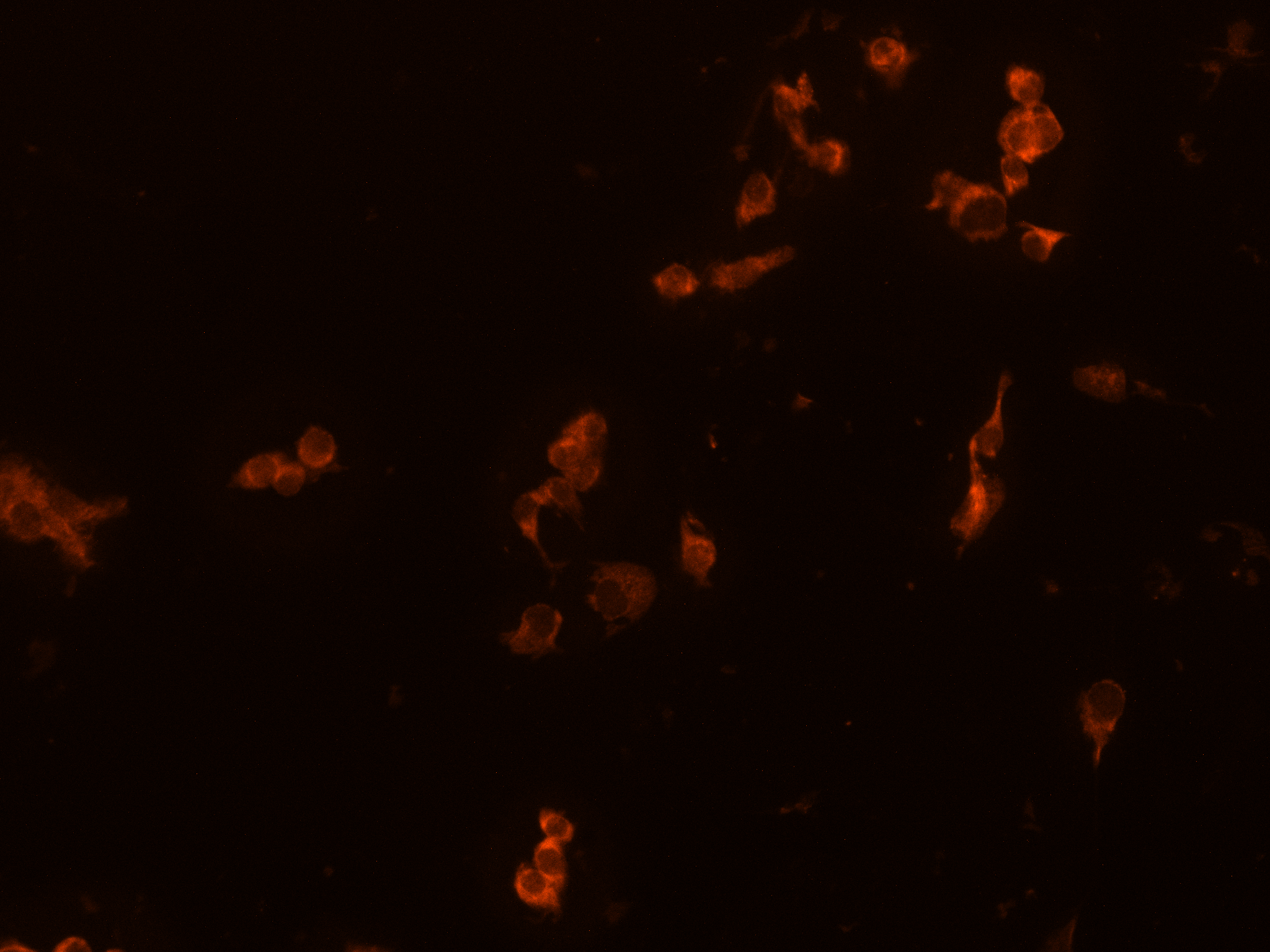

Supplement: Supplementary file 3 — Source data Fig. 2 [file 44318_2024_237_MOESM3_ESM.zip › Figure 2/Figure2L/730-lps_Bottom Slide_D_p01_0_A01f16d2.TIF]

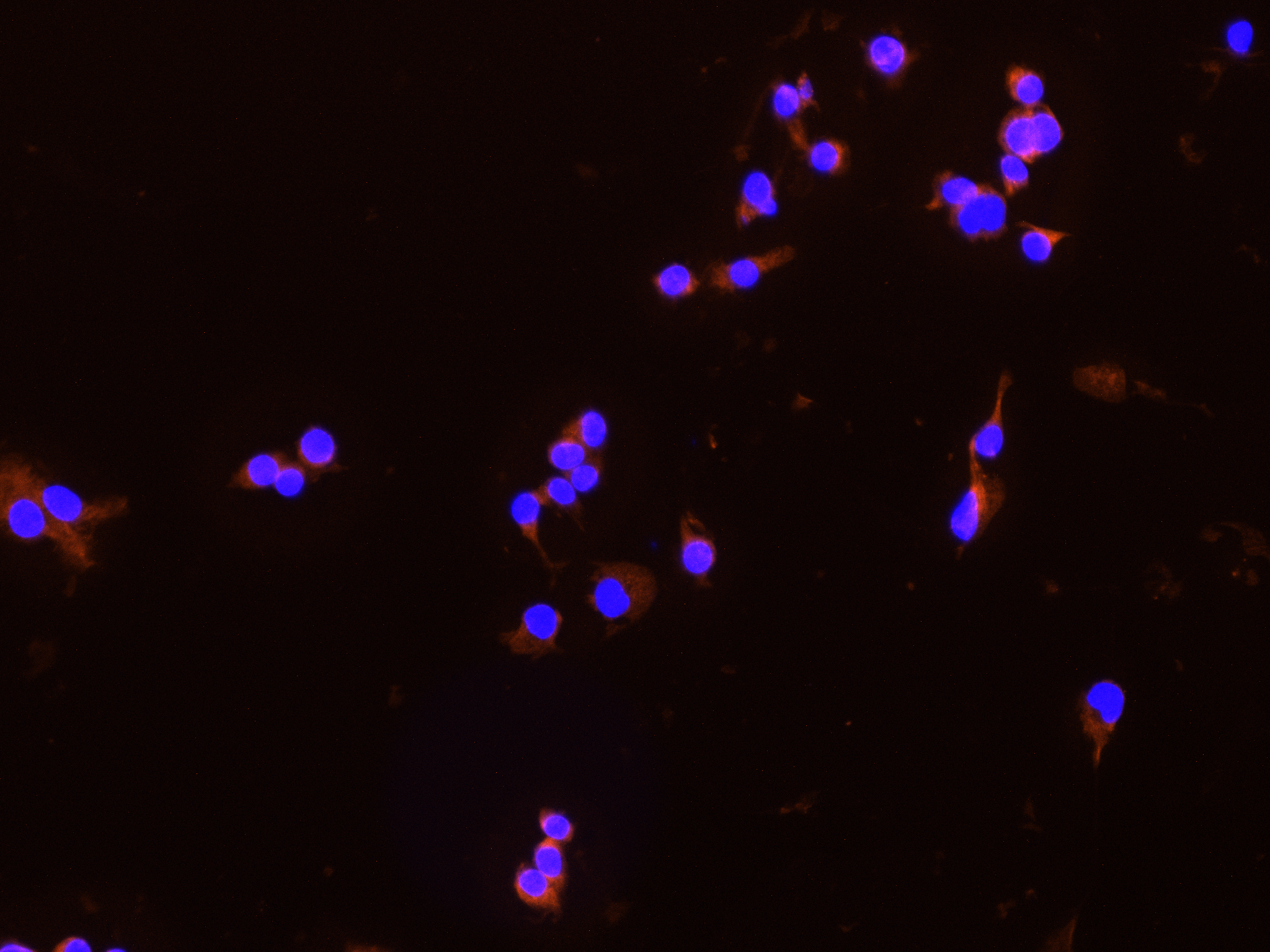

Supplement: Supplementary file 3 — Source data Fig. 2 [file 44318_2024_237_MOESM3_ESM.zip › Figure 2/Figure2L/730-lps_Bottom Slide_M_p01_0_A01f16d0.TIF]

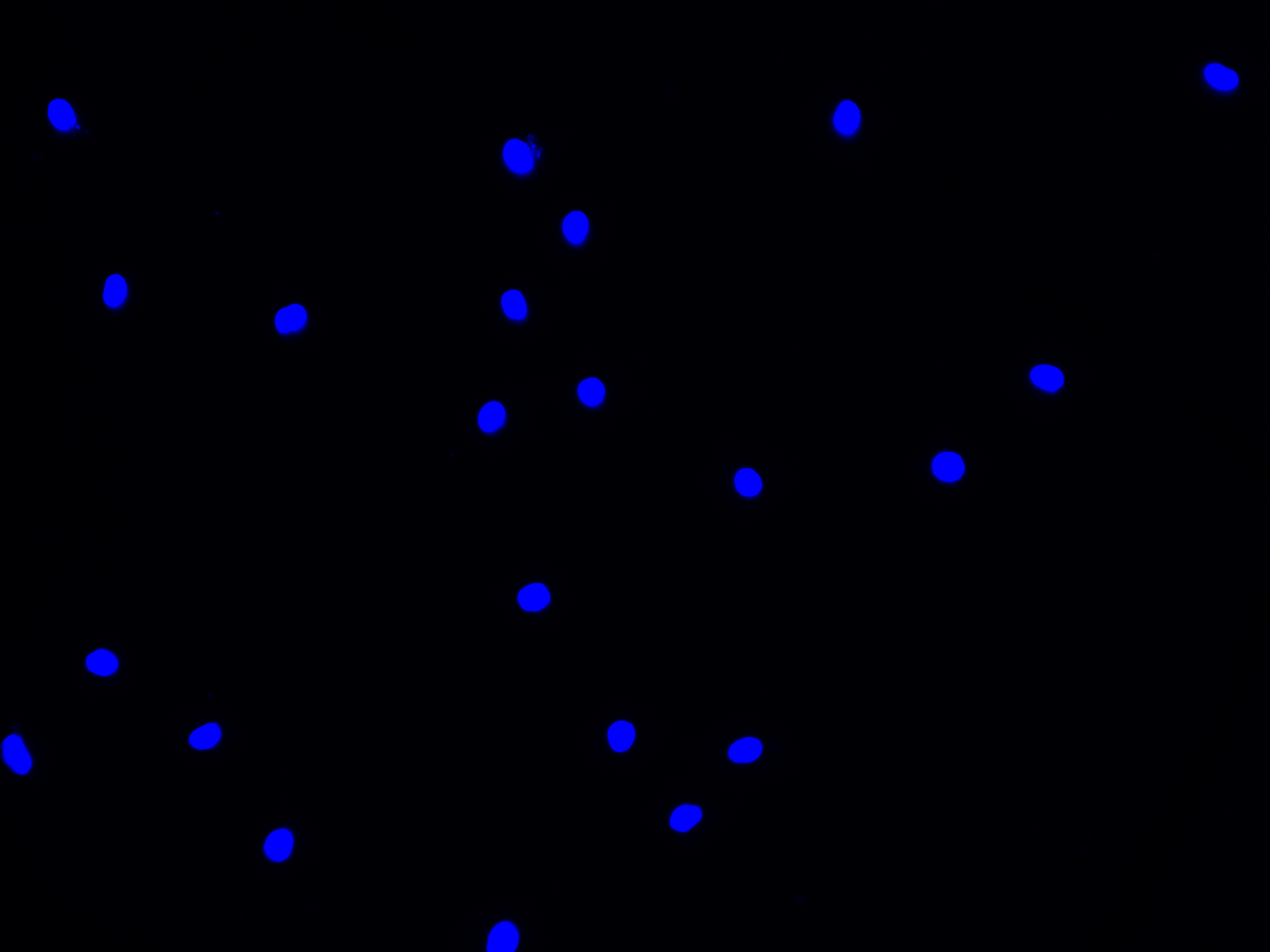

Supplement: Supplementary file 3 — Source data Fig. 2 [file 44318_2024_237_MOESM3_ESM.zip › Figure 2/Figure2L/810 lps_Bottom Slide_D_p00_0_A01f24d0.TIF]

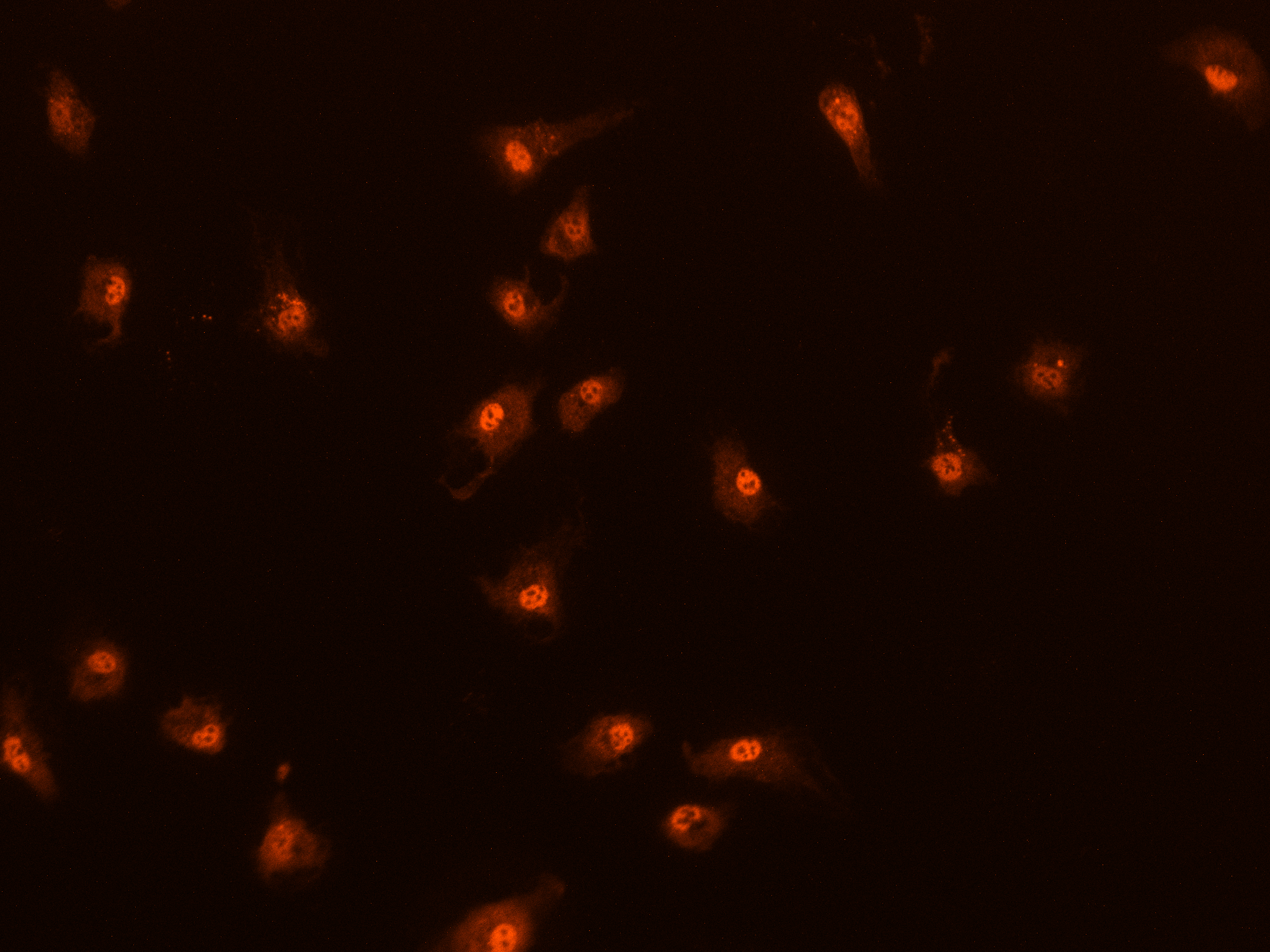

Supplement: Supplementary file 3 — Source data Fig. 2 [file 44318_2024_237_MOESM3_ESM.zip › Figure 2/Figure2L/810 lps_Bottom Slide_D_p00_0_A01f24d2.TIF]

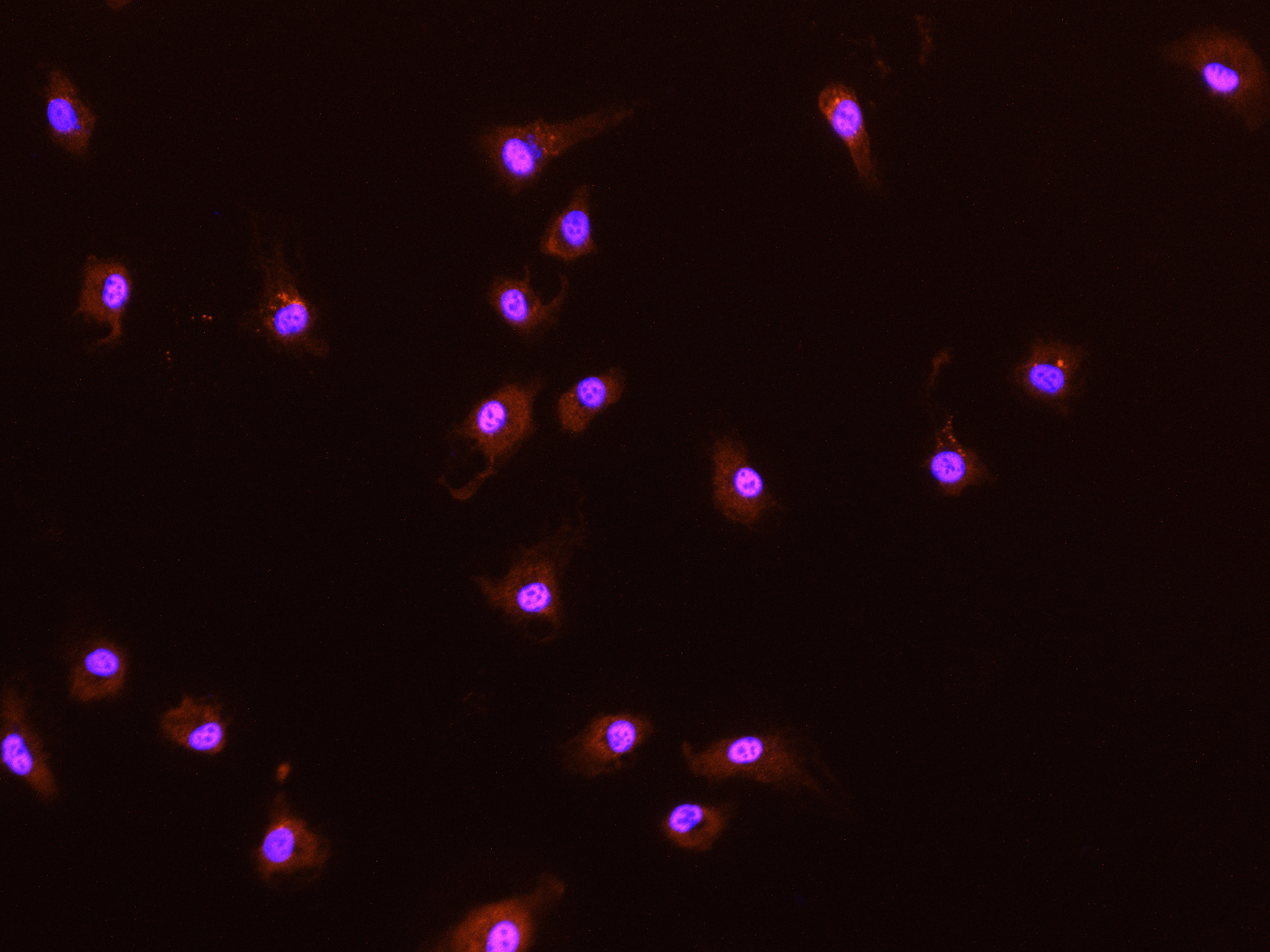

Supplement: Supplementary file 3 — Source data Fig. 2 [file 44318_2024_237_MOESM3_ESM.zip › Figure 2/Figure2L/810 lps_Bottom Slide_M_p00_0_A01f24d0.TIF]

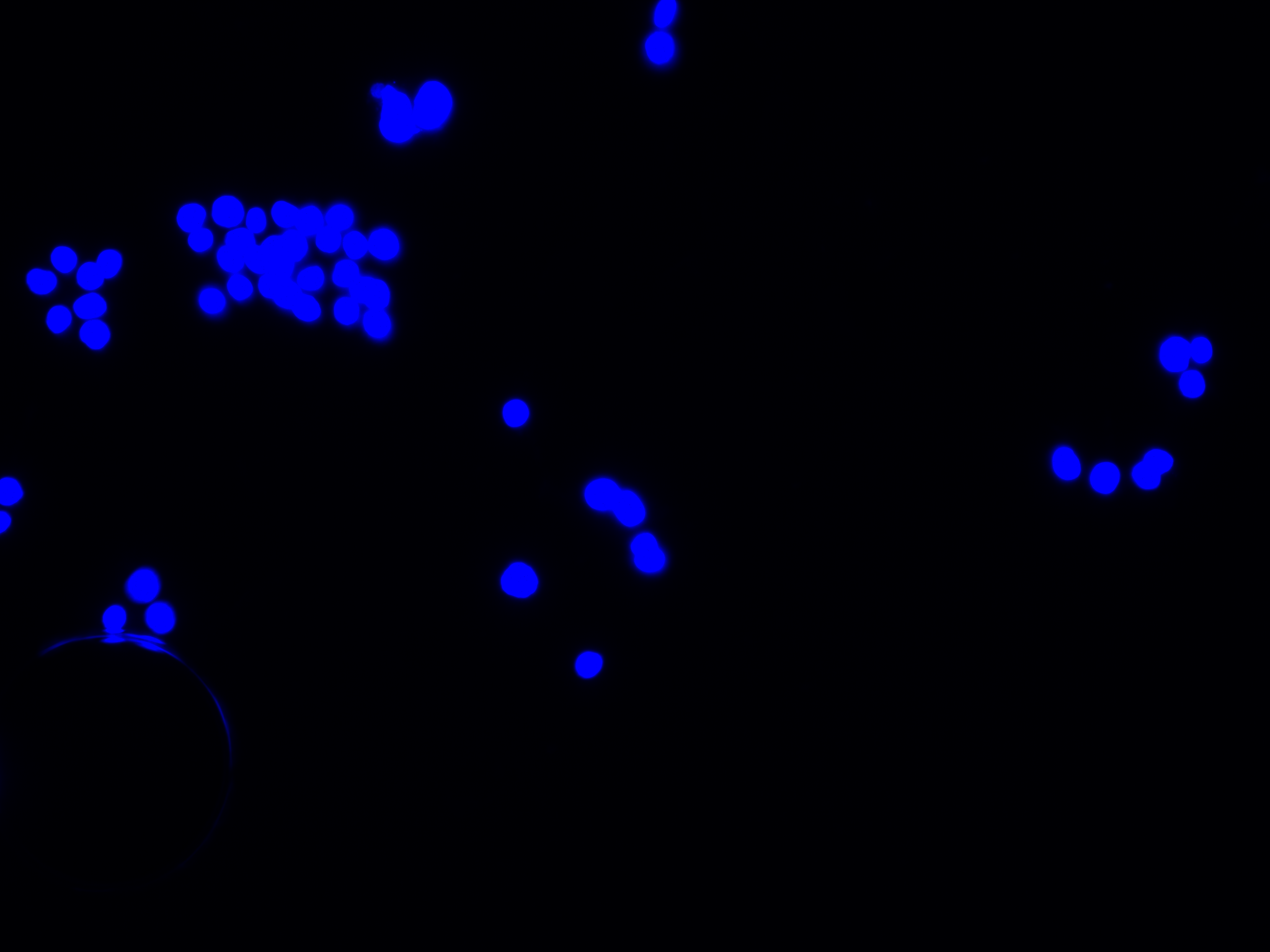

Supplement: Supplementary file 3 — Source data Fig. 2 [file 44318_2024_237_MOESM3_ESM.zip › Figure 2/Figure2L/810-lps_Bottom Slide_D_p01_0_A01f16d0.TIF]

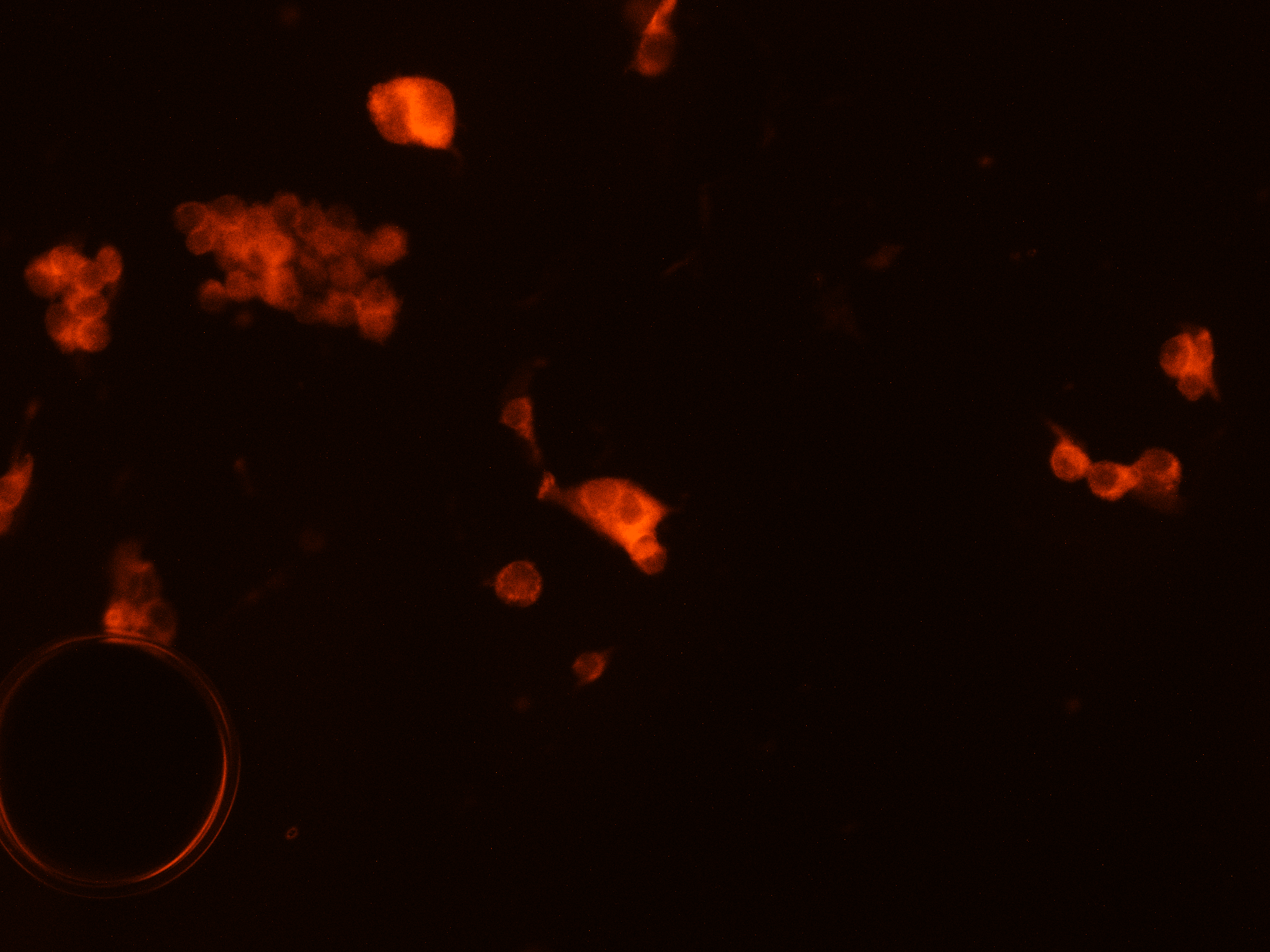

Supplement: Supplementary file 3 — Source data Fig. 2 [file 44318_2024_237_MOESM3_ESM.zip › Figure 2/Figure2L/810-lps_Bottom Slide_D_p01_0_A01f16d2.TIF]

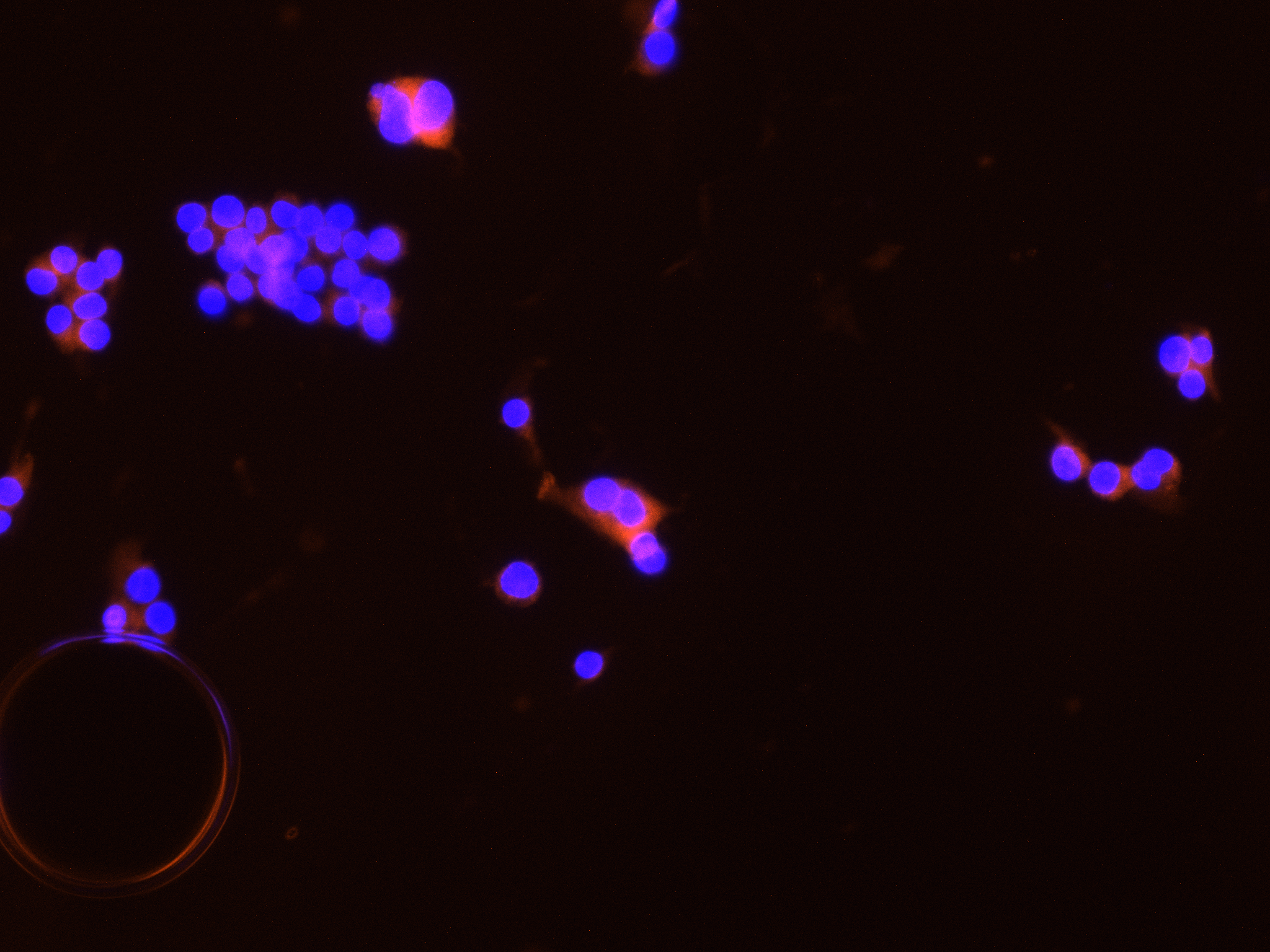

Supplement: Supplementary file 3 — Source data Fig. 2 [file 44318_2024_237_MOESM3_ESM.zip › Figure 2/Figure2L/810-lps_Bottom Slide_M_p01_0_A01f16d0.TIF]

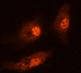

Supplement: Supplementary file 3 — Source data Fig. 2 [file 44318_2024_237_MOESM3_ESM.zip › Figure 2/Figure2L/K730R+LPS1.tif]

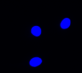

Supplement: Supplementary file 3 — Source data Fig. 2 [file 44318_2024_237_MOESM3_ESM.zip › Figure 2/Figure2L/K730R+LPS2.tif]

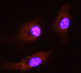

Supplement: Supplementary file 3 — Source data Fig. 2 [file 44318_2024_237_MOESM3_ESM.zip › Figure 2/Figure2L/K730R+LPS3.tif]

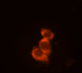

Supplement: Supplementary file 3 — Source data Fig. 2 [file 44318_2024_237_MOESM3_ESM.zip › Figure 2/Figure2L/K730R-LPS1.tif]

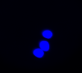

Supplement: Supplementary file 3 — Source data Fig. 2 [file 44318_2024_237_MOESM3_ESM.zip › Figure 2/Figure2L/K730R-LPS2.tif]

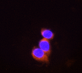

Supplement: Supplementary file 3 — Source data Fig. 2 [file 44318_2024_237_MOESM3_ESM.zip › Figure 2/Figure2L/K730R-LPS3.tif]

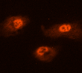

Supplement: Supplementary file 3 — Source data Fig. 2 [file 44318_2024_237_MOESM3_ESM.zip › Figure 2/Figure2L/K810R+LPS1.tif]

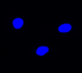

Supplement: Supplementary file 3 — Source data Fig. 2 [file 44318_2024_237_MOESM3_ESM.zip › Figure 2/Figure2L/K810R+LPS2.tif]

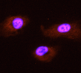

Supplement: Supplementary file 3 — Source data Fig. 2 [file 44318_2024_237_MOESM3_ESM.zip › Figure 2/Figure2L/K810R+LPS3.tif]

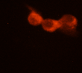

Supplement: Supplementary file 3 — Source data Fig. 2 [file 44318_2024_237_MOESM3_ESM.zip › Figure 2/Figure2L/K810R-LPS1.tif]

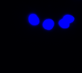

Supplement: Supplementary file 3 — Source data Fig. 2 [file 44318_2024_237_MOESM3_ESM.zip › Figure 2/Figure2L/K810R-LPS2.tif]

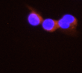

Supplement: Supplementary file 3 — Source data Fig. 2 [file 44318_2024_237_MOESM3_ESM.zip › Figure 2/Figure2L/K810R-LPS3.tif]

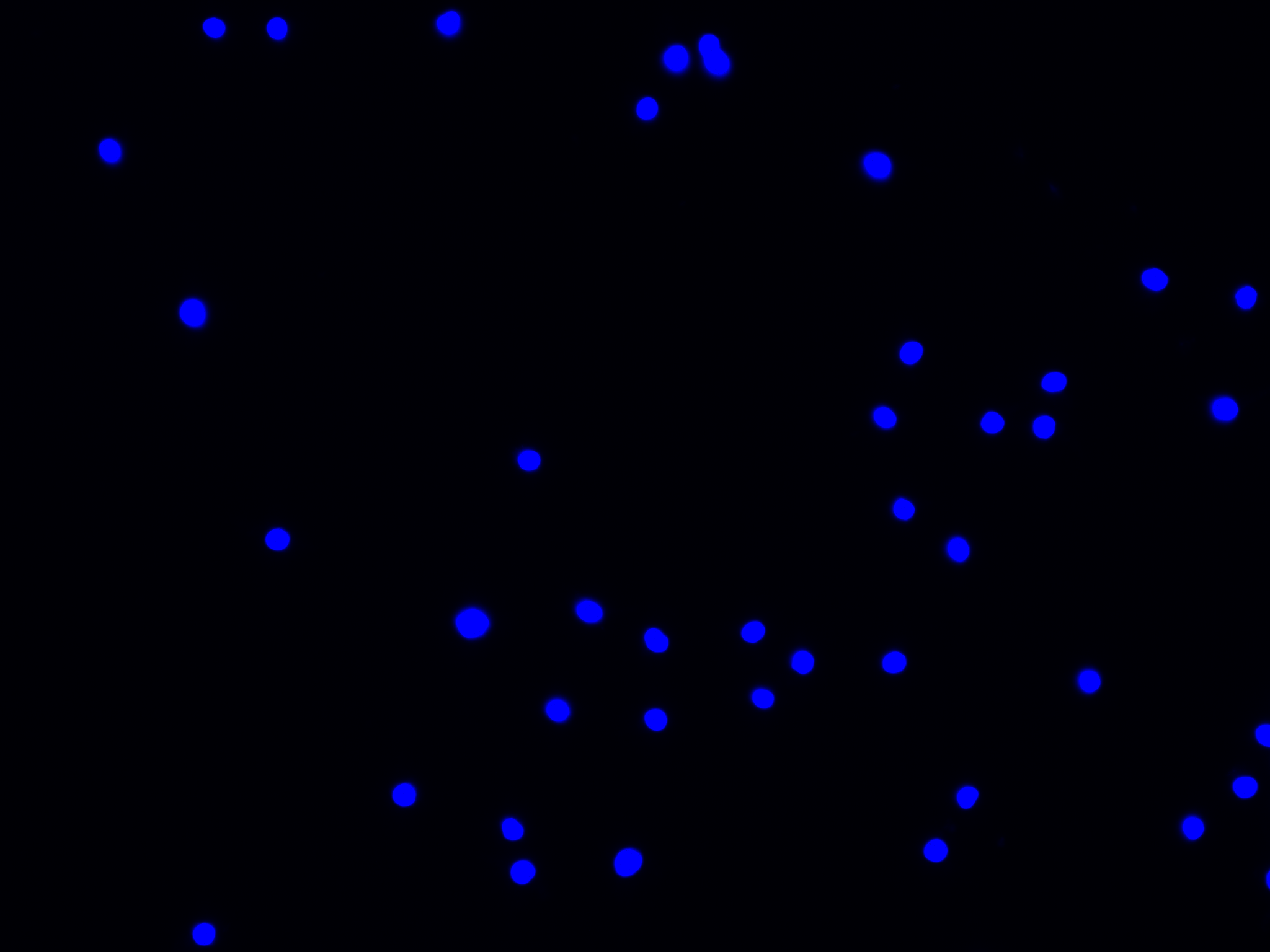

Supplement: Supplementary file 3 — Source data Fig. 2 [file 44318_2024_237_MOESM3_ESM.zip › Figure 2/Figure2L/wt lps_Bottom Slide_D_p00_0_A01f12d0.TIF]

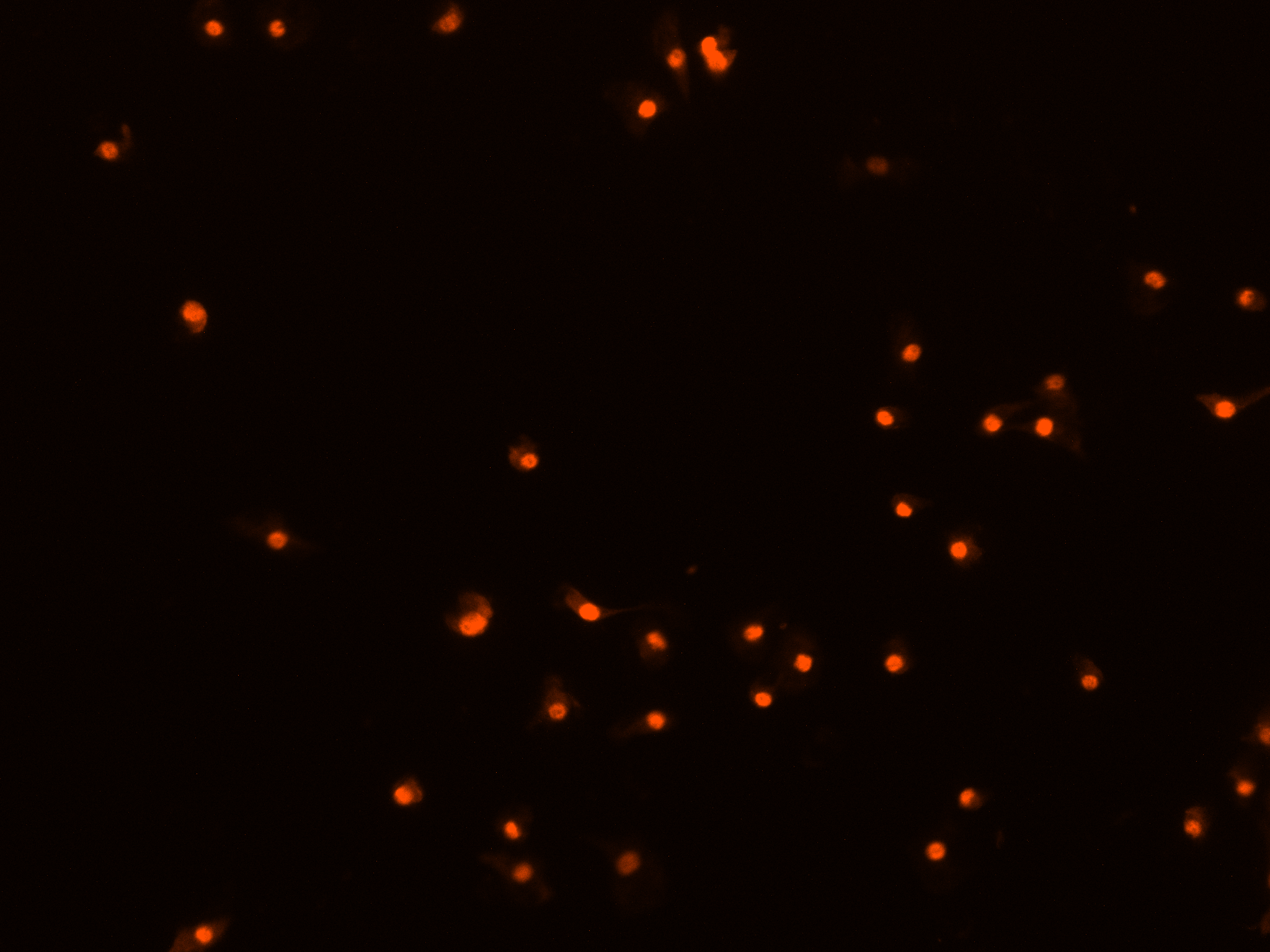

Supplement: Supplementary file 3 — Source data Fig. 2 [file 44318_2024_237_MOESM3_ESM.zip › Figure 2/Figure2L/wt lps_Bottom Slide_D_p00_0_A01f12d2.TIF]

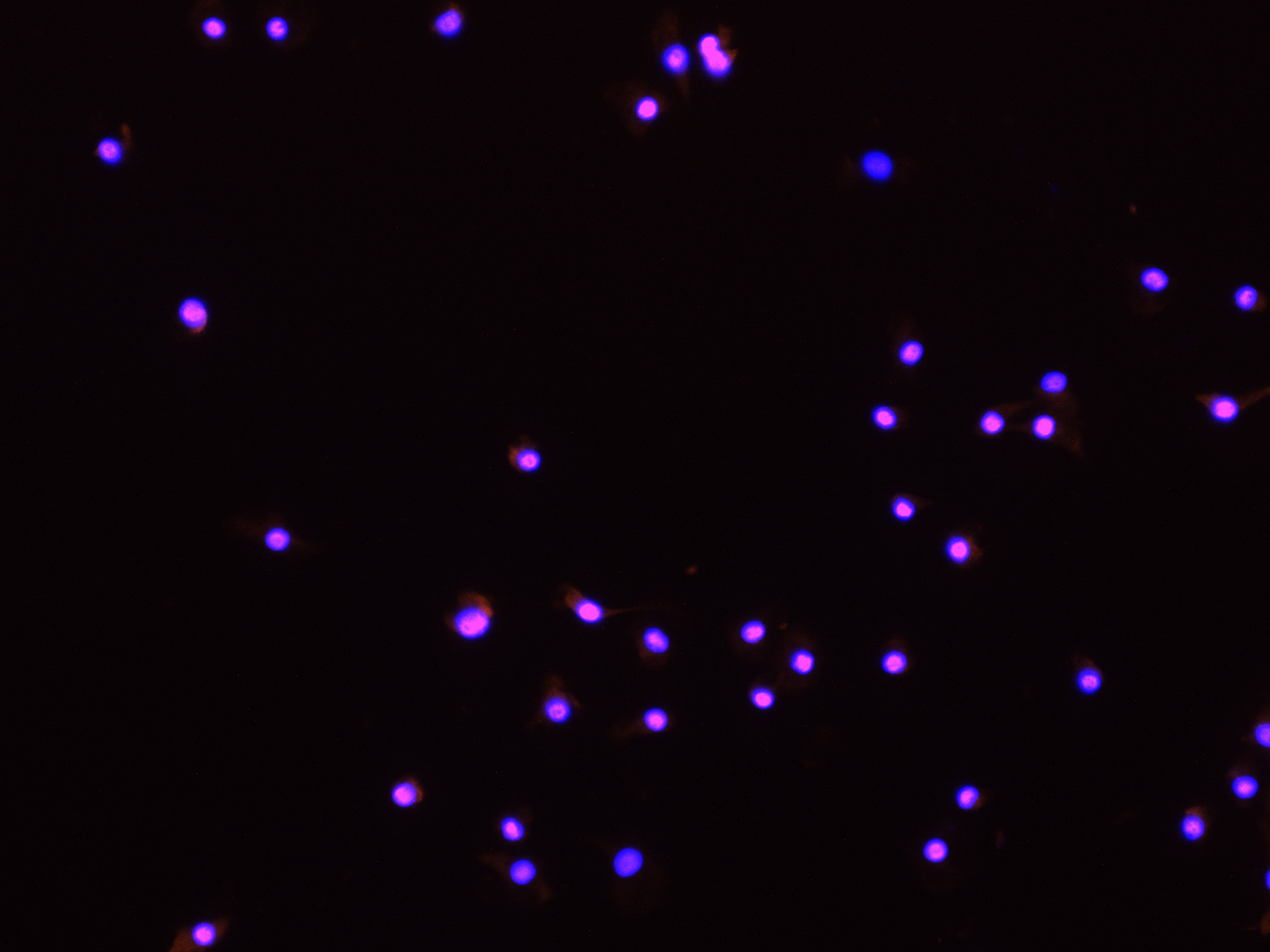

Supplement: Supplementary file 3 — Source data Fig. 2 [file 44318_2024_237_MOESM3_ESM.zip › Figure 2/Figure2L/wt lps_Bottom Slide_M_p00_0_A01f12d0.TIF]

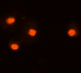

Supplement: Supplementary file 3 — Source data Fig. 2 [file 44318_2024_237_MOESM3_ESM.zip › Figure 2/Figure2L/WT+LPS1.tif]

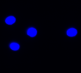

Supplement: Supplementary file 3 — Source data Fig. 2 [file 44318_2024_237_MOESM3_ESM.zip › Figure 2/Figure2L/WT+LPS2.tif]

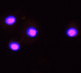

Supplement: Supplementary file 3 — Source data Fig. 2 [file 44318_2024_237_MOESM3_ESM.zip › Figure 2/Figure2L/WT+LPS3.tif]

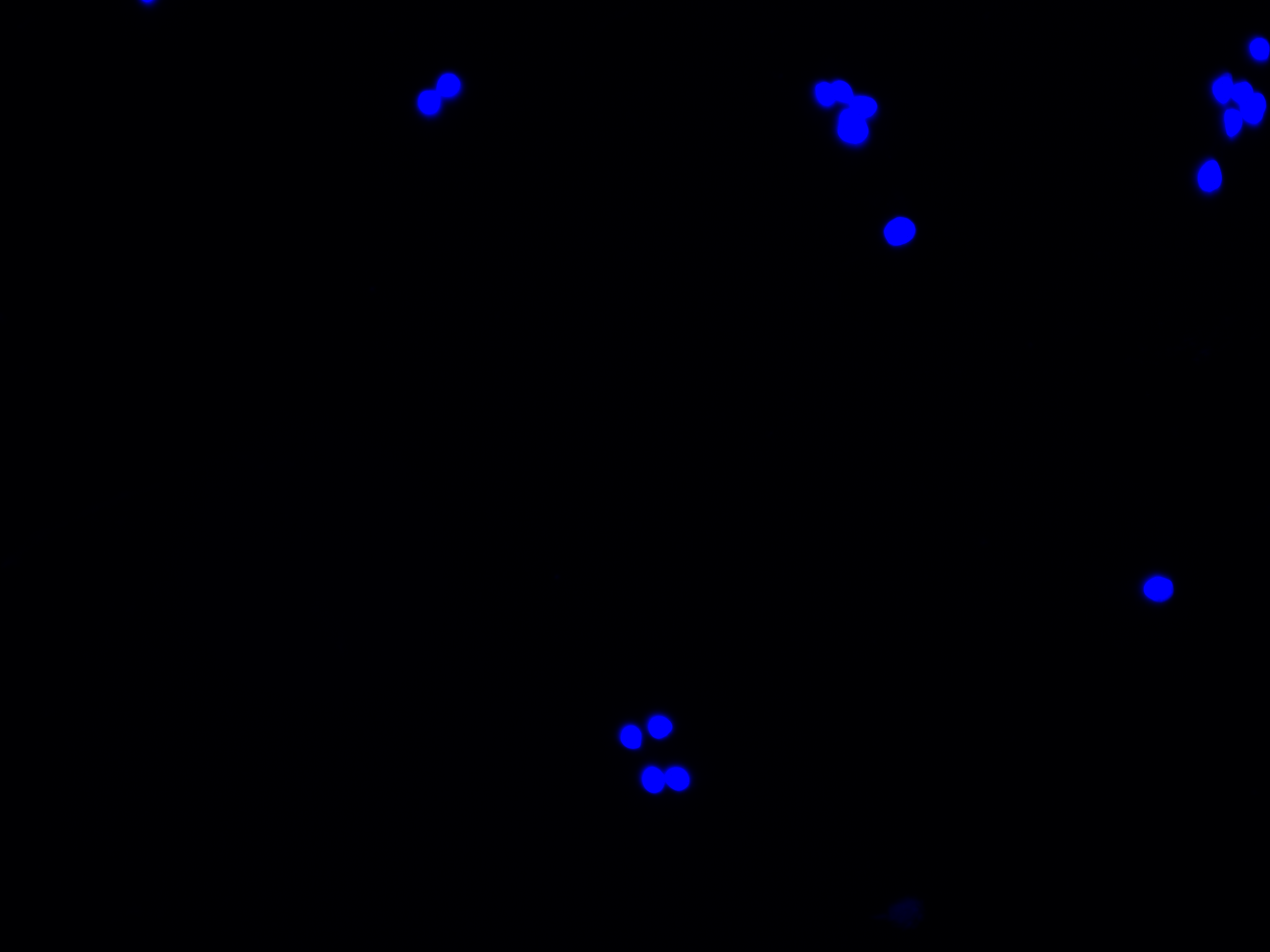

Supplement: Supplementary file 3 — Source data Fig. 2 [file 44318_2024_237_MOESM3_ESM.zip › Figure 2/Figure2L/wt-lps_Bottom Slide_D_p01_0_A01f12d0.TIF]

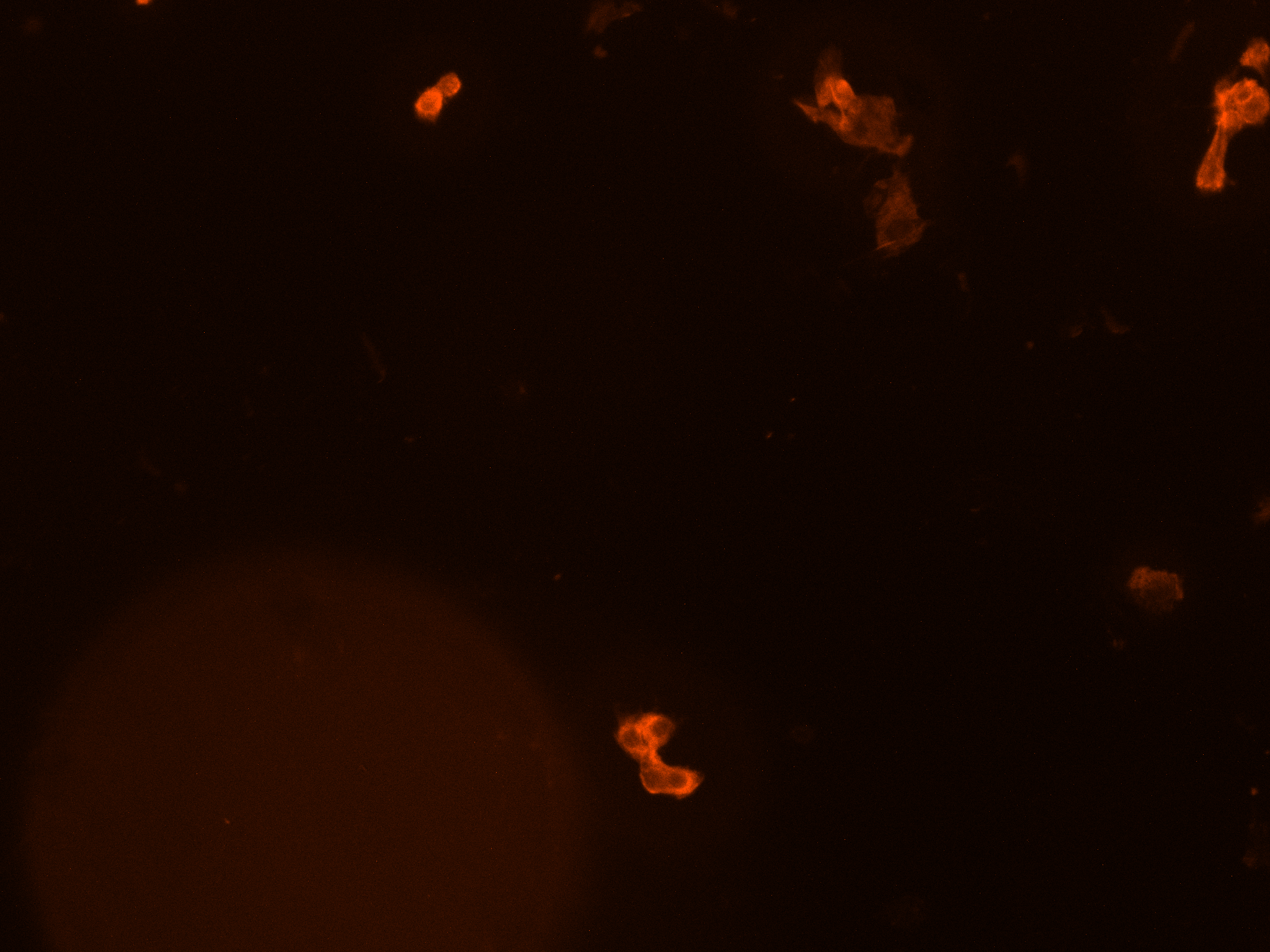

Supplement: Supplementary file 3 — Source data Fig. 2 [file 44318_2024_237_MOESM3_ESM.zip › Figure 2/Figure2L/wt-lps_Bottom Slide_D_p01_0_A01f12d2.TIF]

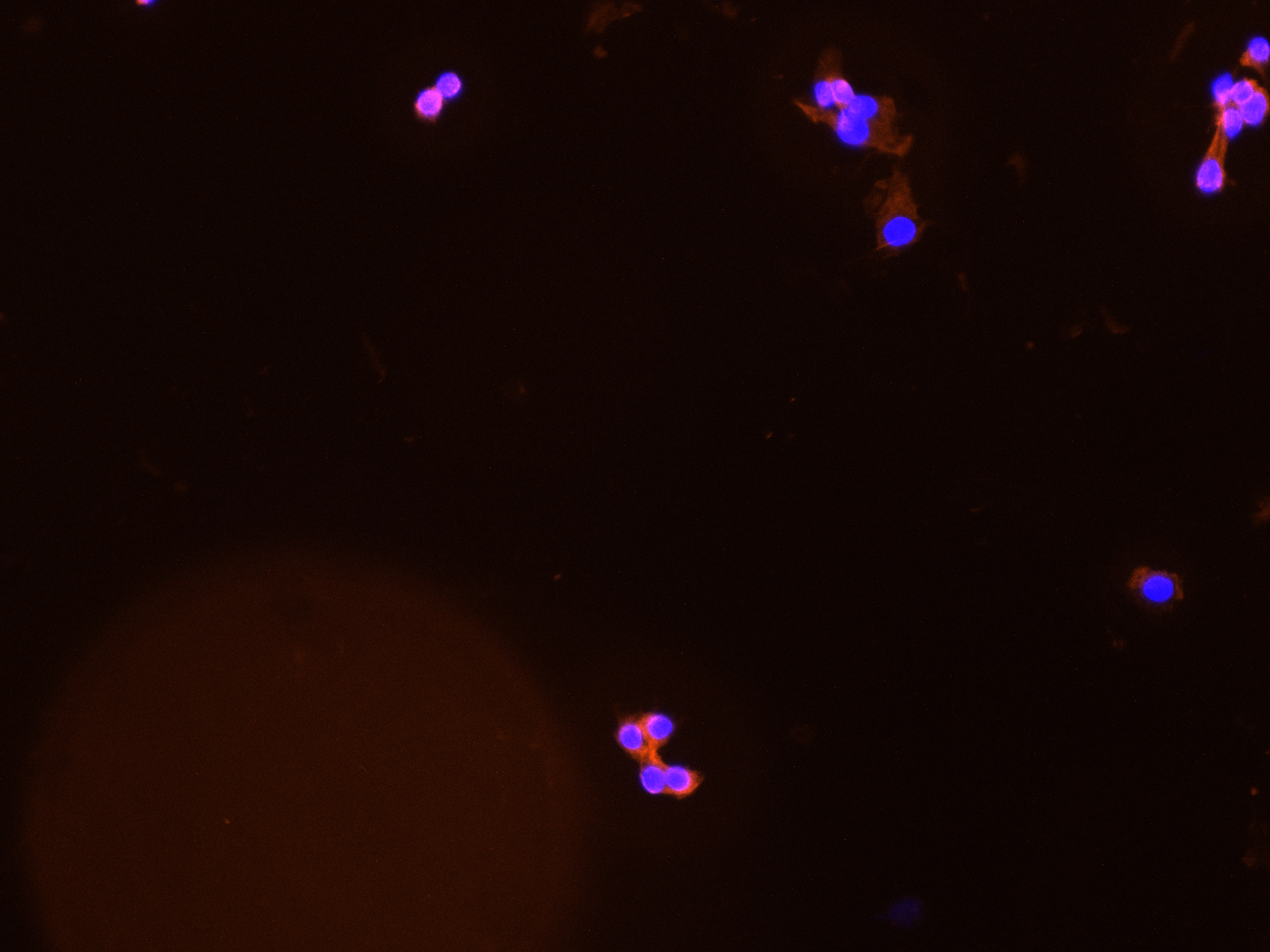

Supplement: Supplementary file 3 — Source data Fig. 2 [file 44318_2024_237_MOESM3_ESM.zip › Figure 2/Figure2L/wt-lps_Bottom Slide_M_p01_0_A01f12d0.TIF]

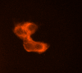

Supplement: Supplementary file 3 — Source data Fig. 2 [file 44318_2024_237_MOESM3_ESM.zip › Figure 2/Figure2L/WT-LPS1.tif]

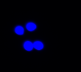

Supplement: Supplementary file 3 — Source data Fig. 2 [file 44318_2024_237_MOESM3_ESM.zip › Figure 2/Figure2L/WT-LPS2.tif]

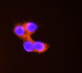

Supplement: Supplementary file 3 — Source data Fig. 2 [file 44318_2024_237_MOESM3_ESM.zip › Figure 2/Figure2L/WT-LPS3.tif]

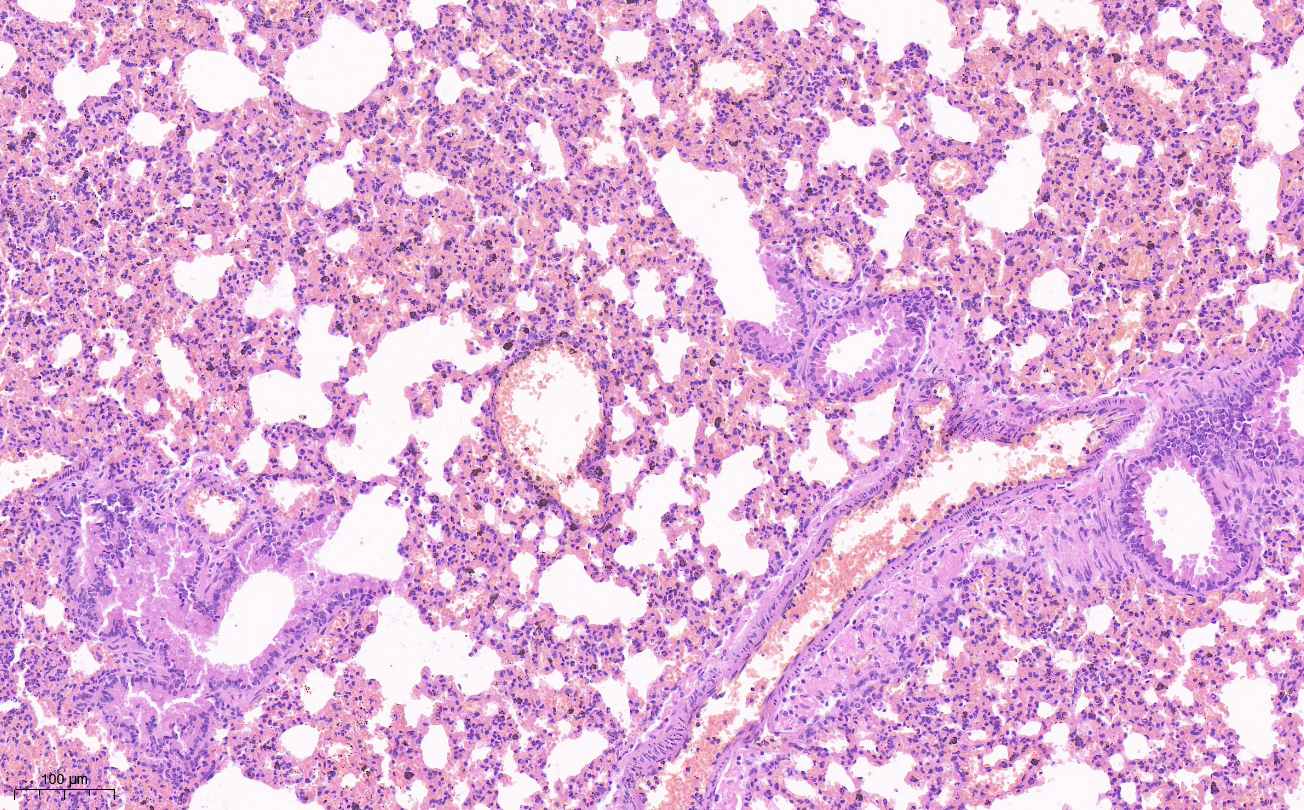

Supplement: Supplementary file 5 — Source data Fig. 4 [file 44318_2024_237_MOESM5_ESM.zip › Figure 4/Figure 4B/K730R+LPS LUNG_10.0x.tif]

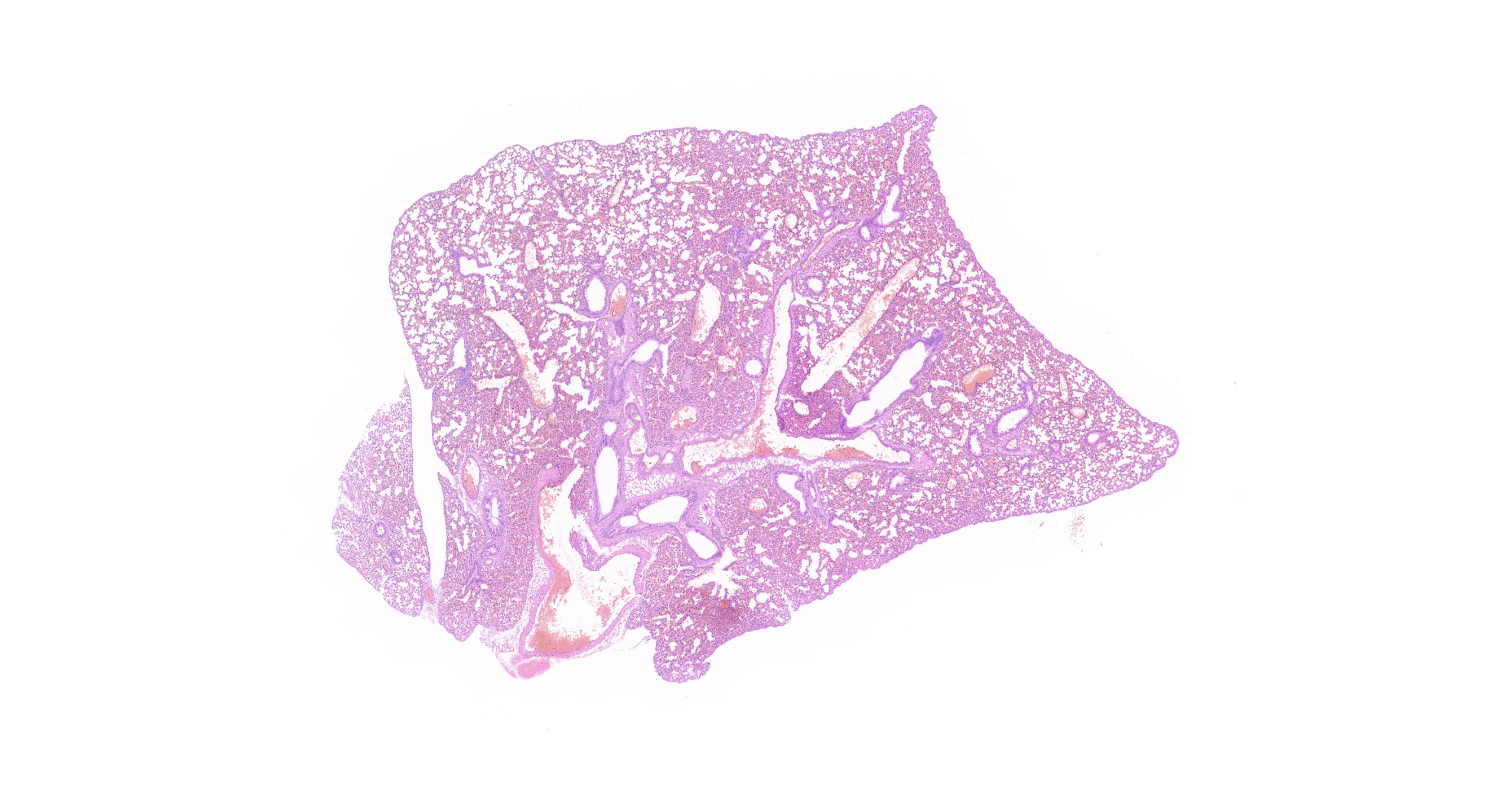

Supplement: Supplementary file 5 — Source data Fig. 4 [file 44318_2024_237_MOESM5_ESM.zip › Figure 4/Figure 4B/K730R+LPS LUNG_2.0x.tif]

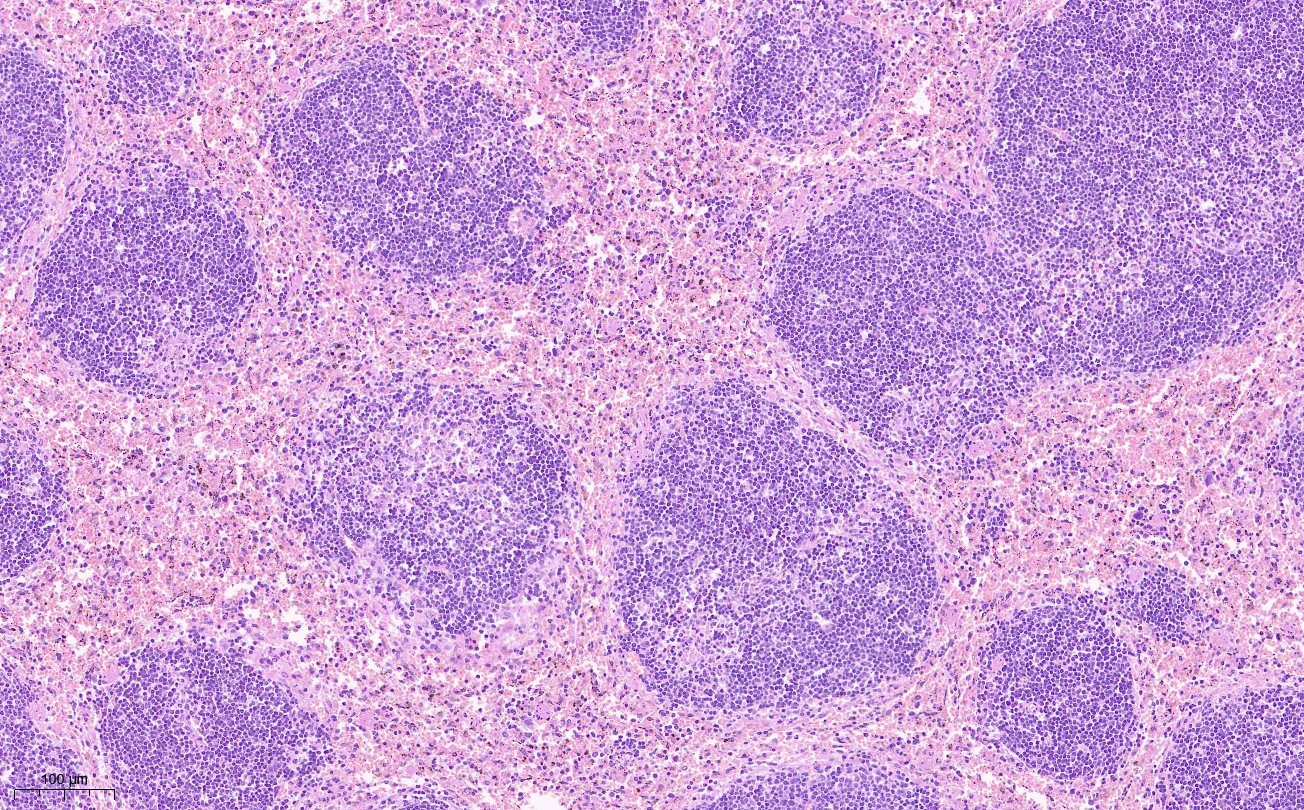

Supplement: Supplementary file 5 — Source data Fig. 4 [file 44318_2024_237_MOESM5_ESM.zip › Figure 4/Figure 4B/K730R+LPS SPLEEN_10.0x.tif]
