# Supplementary figures and images for: Acetylation of TIR domains in the TLR4-Mal-MyD88 complex regulates immune responses in sepsis (part 2 of 3)
Source: EMBO J. 2024 Sep 18;43(21):9. doi: 10.1038/s44318-024-00237-8 (PMC11535217; doi:10.1038/s44318-024-00237-8)

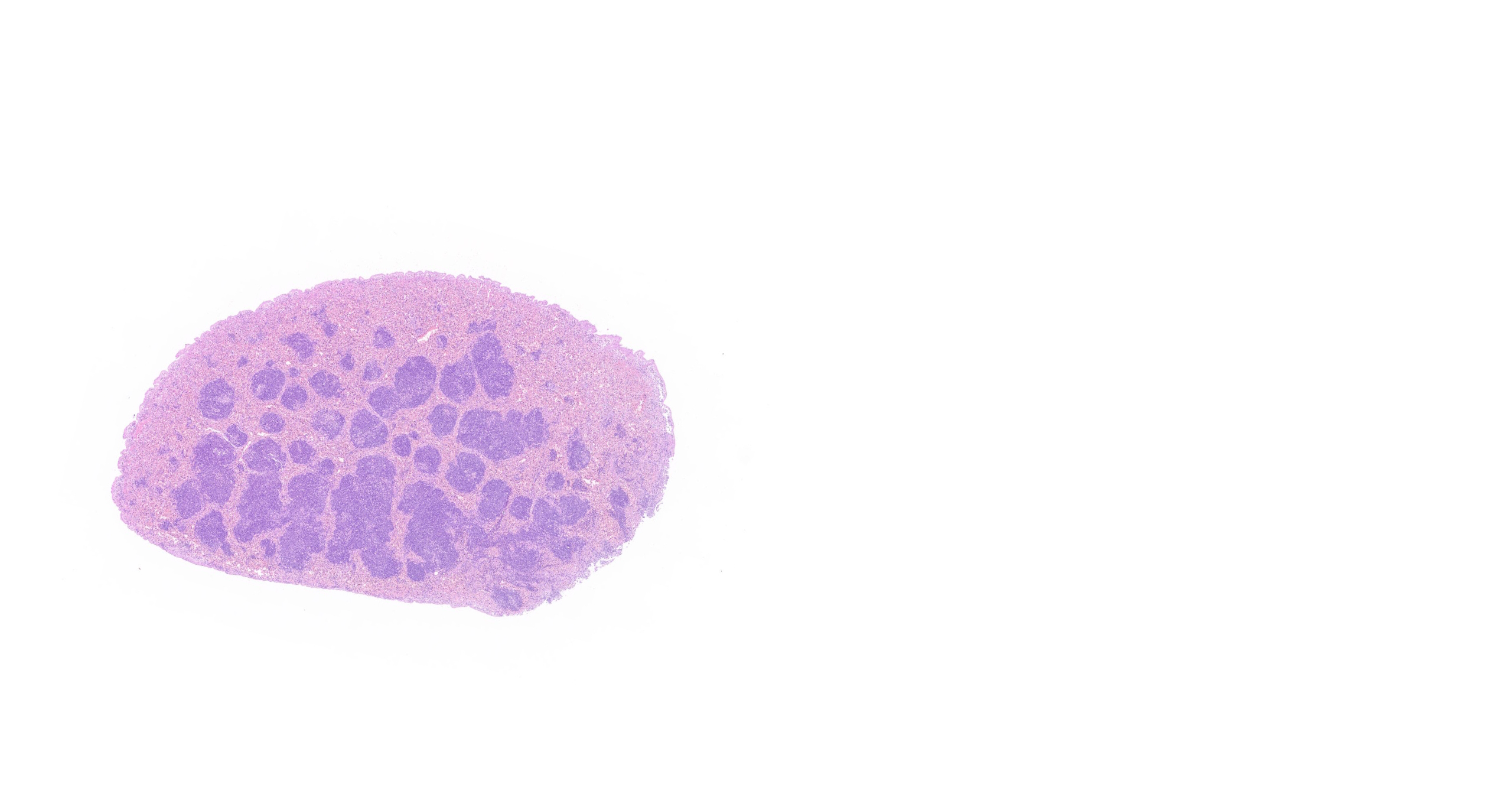

Supplement: Supplementary file 5 — Source data Fig. 4 [file 44318_2024_237_MOESM5_ESM.zip › Figure 4/Figure 4B/K730R+LPS SPLEEN_2.0x.tif]

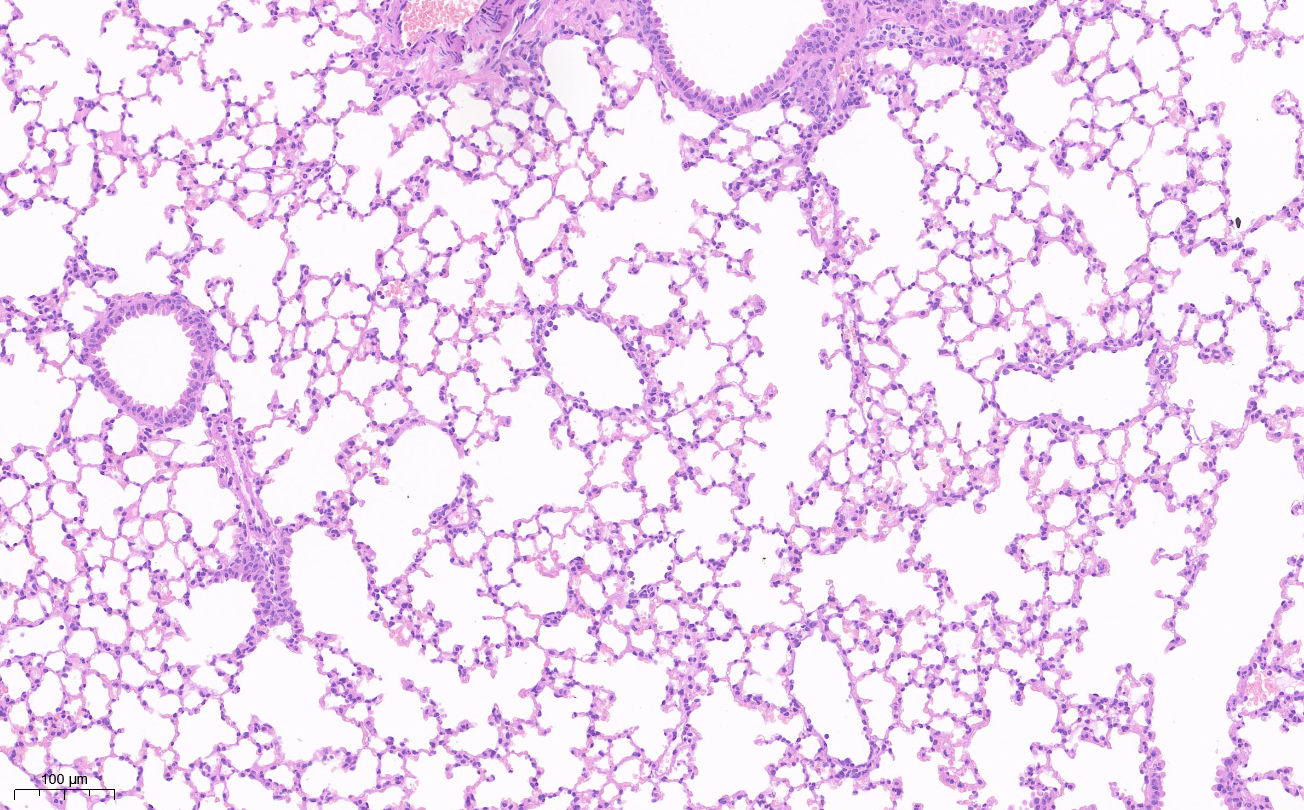

Supplement: Supplementary file 5 — Source data Fig. 4 [file 44318_2024_237_MOESM5_ESM.zip › Figure 4/Figure 4B/K810R+LPS LUNG_10.0x.tif]

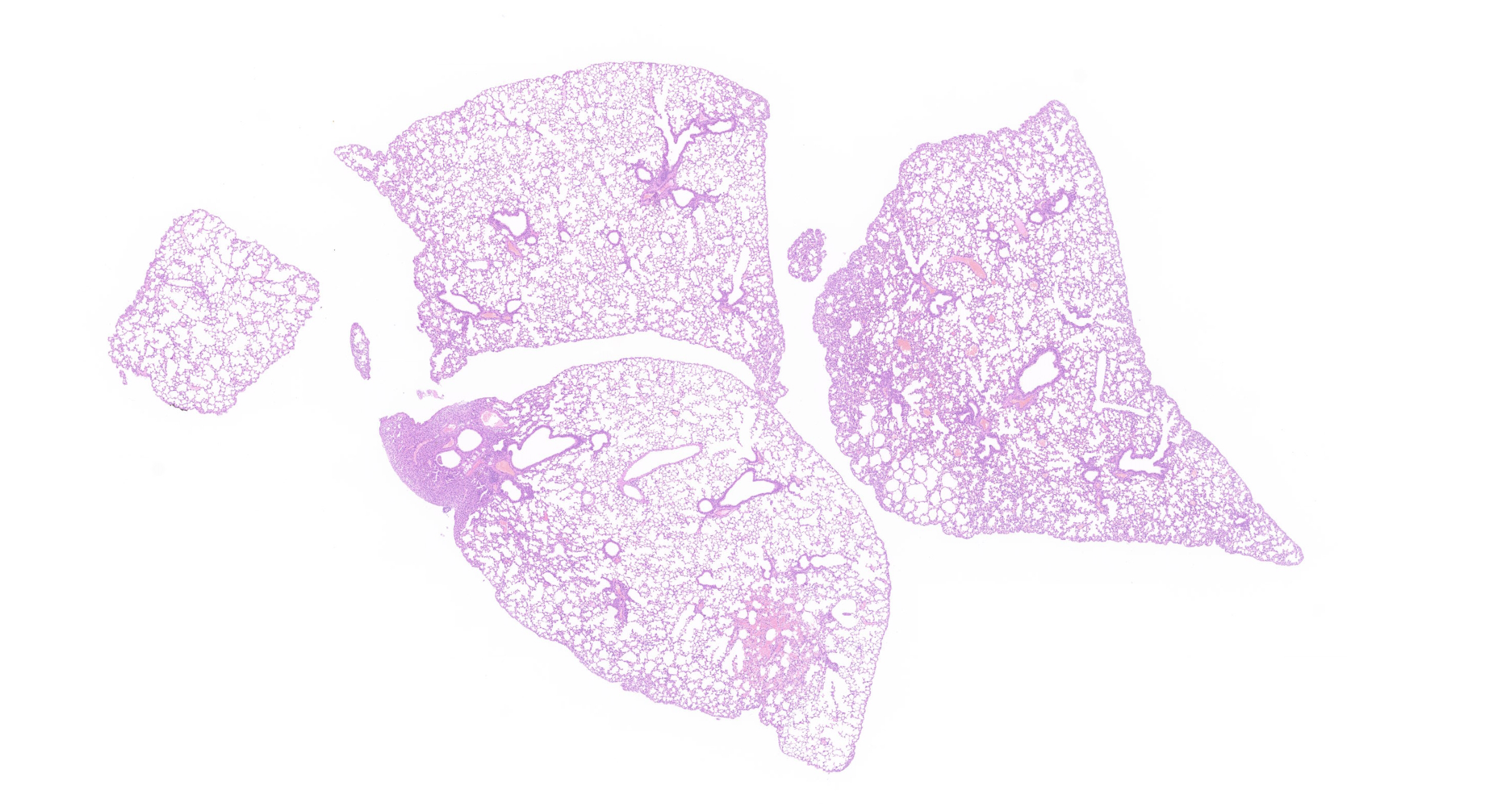

Supplement: Supplementary file 5 — Source data Fig. 4 [file 44318_2024_237_MOESM5_ESM.zip › Figure 4/Figure 4B/K810R+LPS LUNG_2.0x.tif]

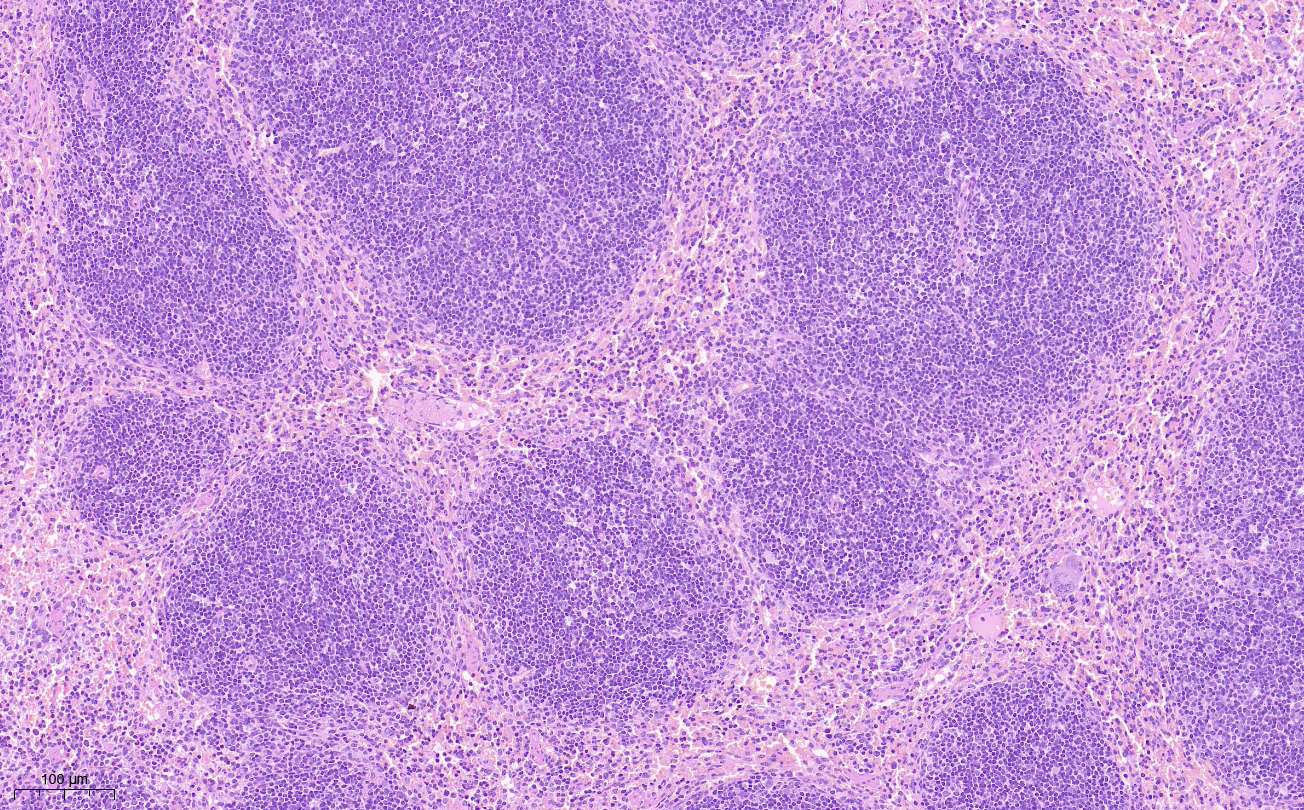

Supplement: Supplementary file 5 — Source data Fig. 4 [file 44318_2024_237_MOESM5_ESM.zip › Figure 4/Figure 4B/K810R+LPS SPLEEN_10.0x.tif]

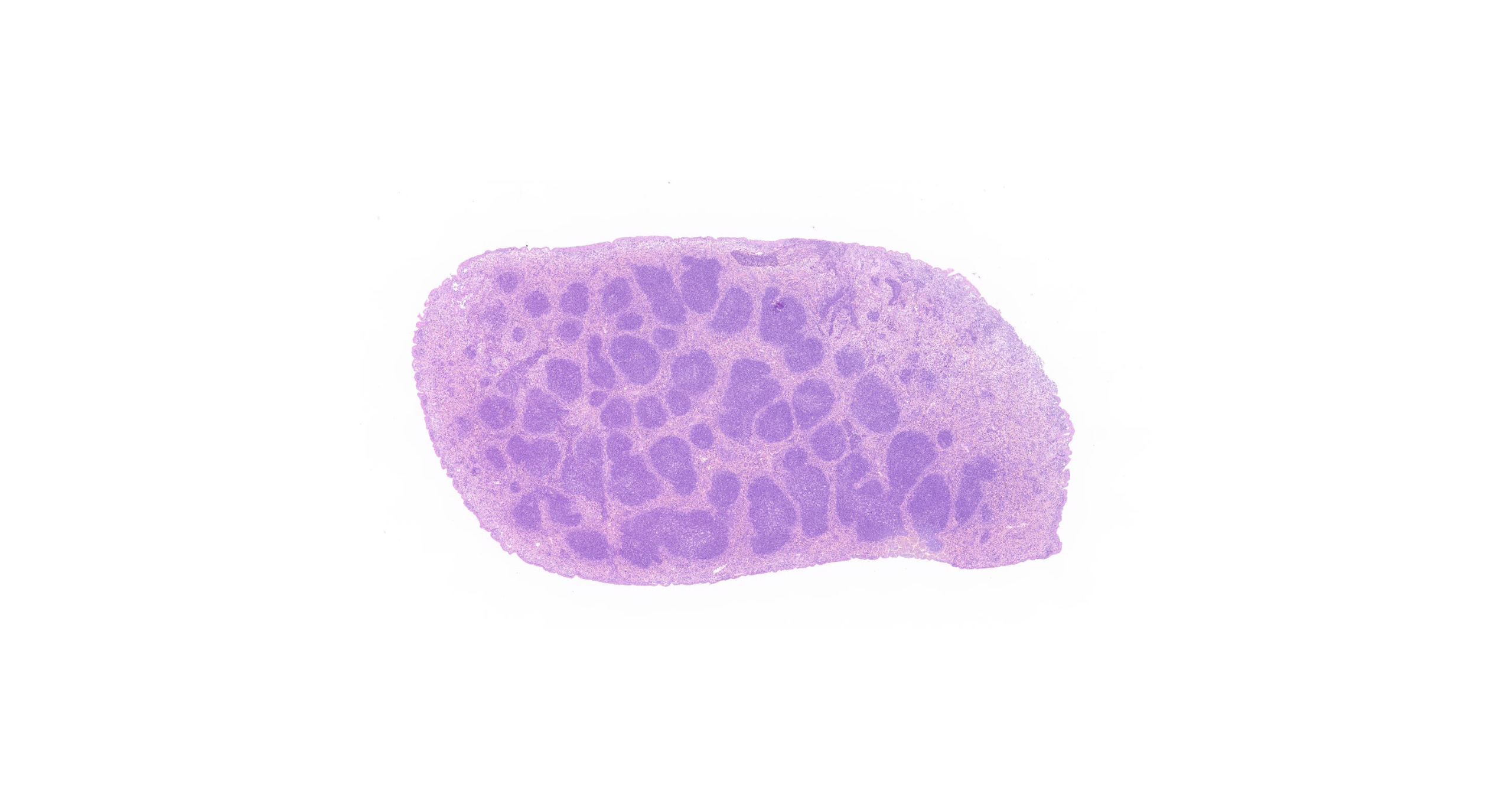

Supplement: Supplementary file 5 — Source data Fig. 4 [file 44318_2024_237_MOESM5_ESM.zip › Figure 4/Figure 4B/K810R+LPS SPLEEN_2.0x.tif]

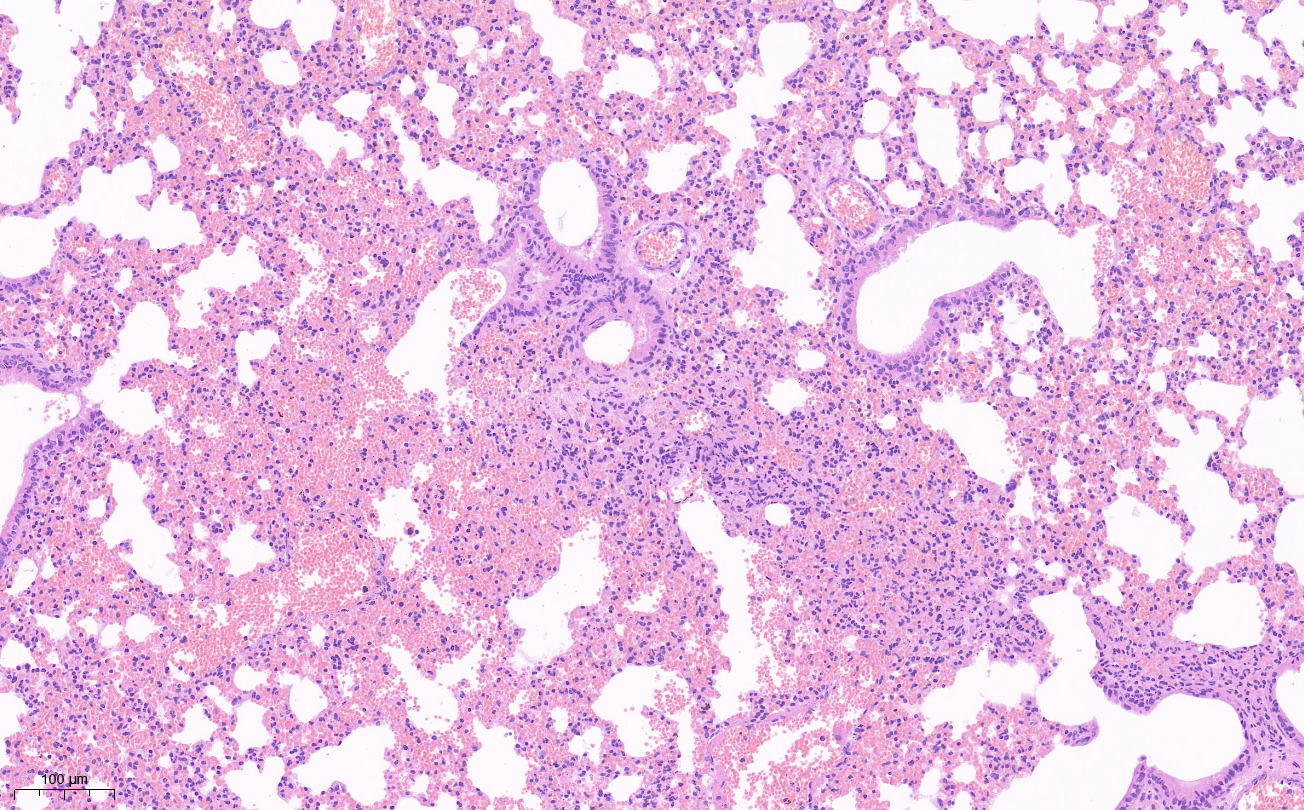

Supplement: Supplementary file 5 — Source data Fig. 4 [file 44318_2024_237_MOESM5_ESM.zip › Figure 4/Figure 4B/WT+LPS LUNG_10.0x.tif]

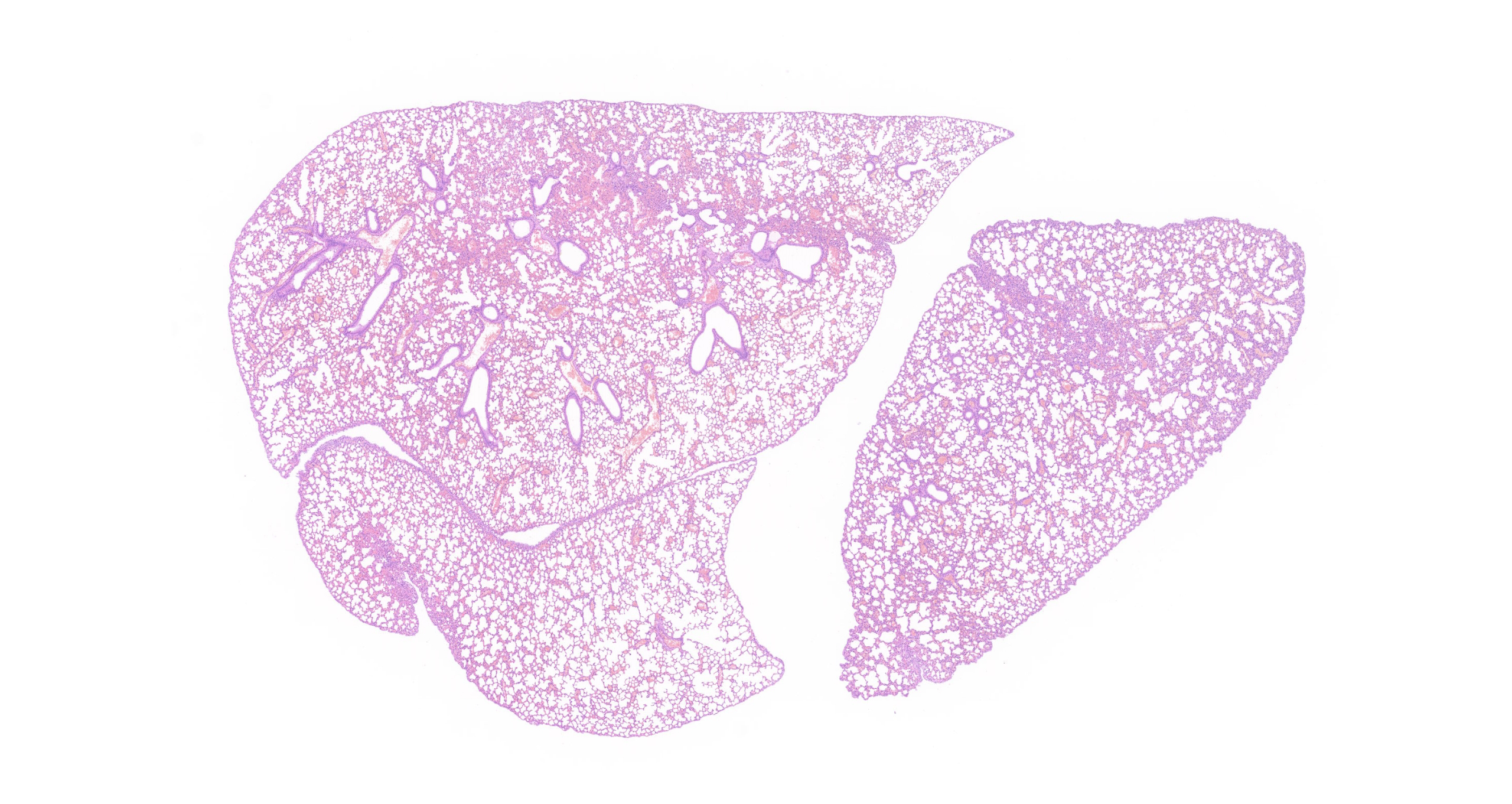

Supplement: Supplementary file 5 — Source data Fig. 4 [file 44318_2024_237_MOESM5_ESM.zip › Figure 4/Figure 4B/WT+LPS LUNG_2.0x.tif]

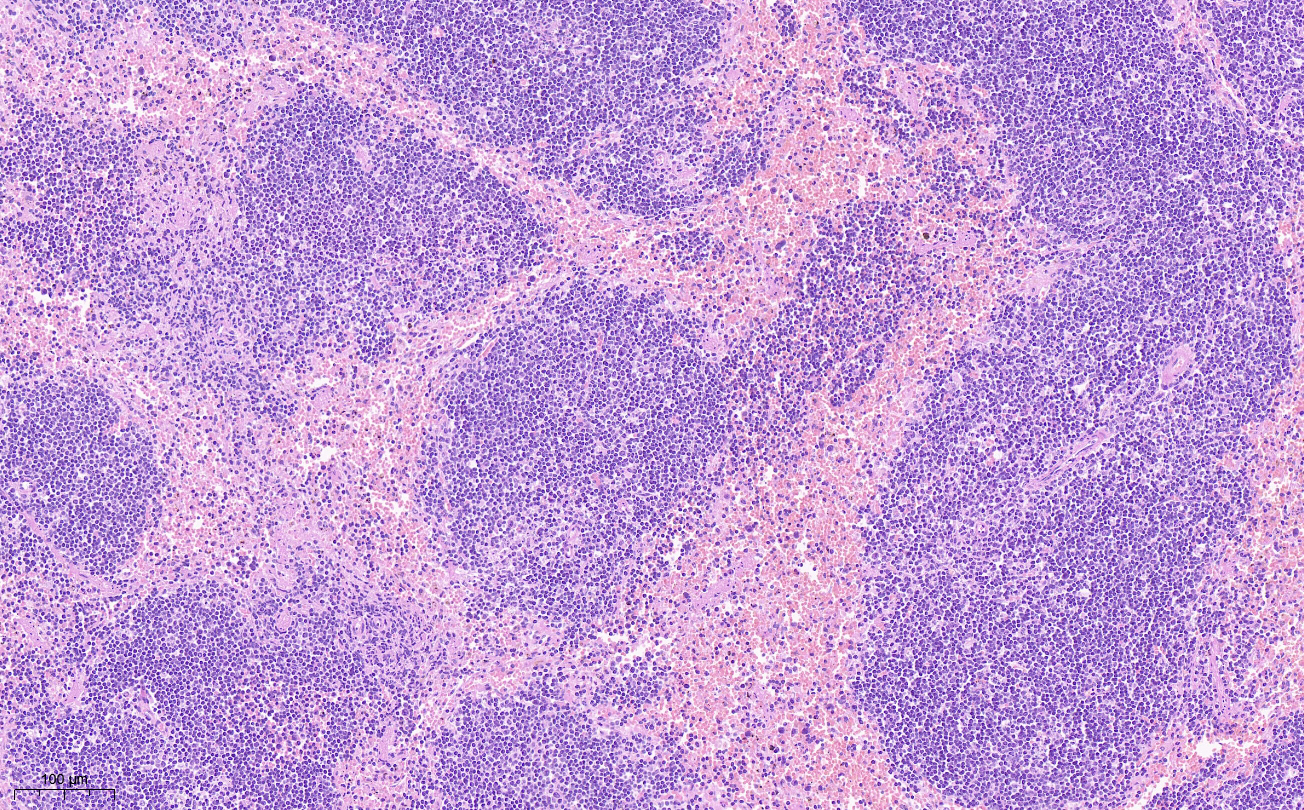

Supplement: Supplementary file 5 — Source data Fig. 4 [file 44318_2024_237_MOESM5_ESM.zip › Figure 4/Figure 4B/WT+LPS SPLEEN_10.0x.tif]

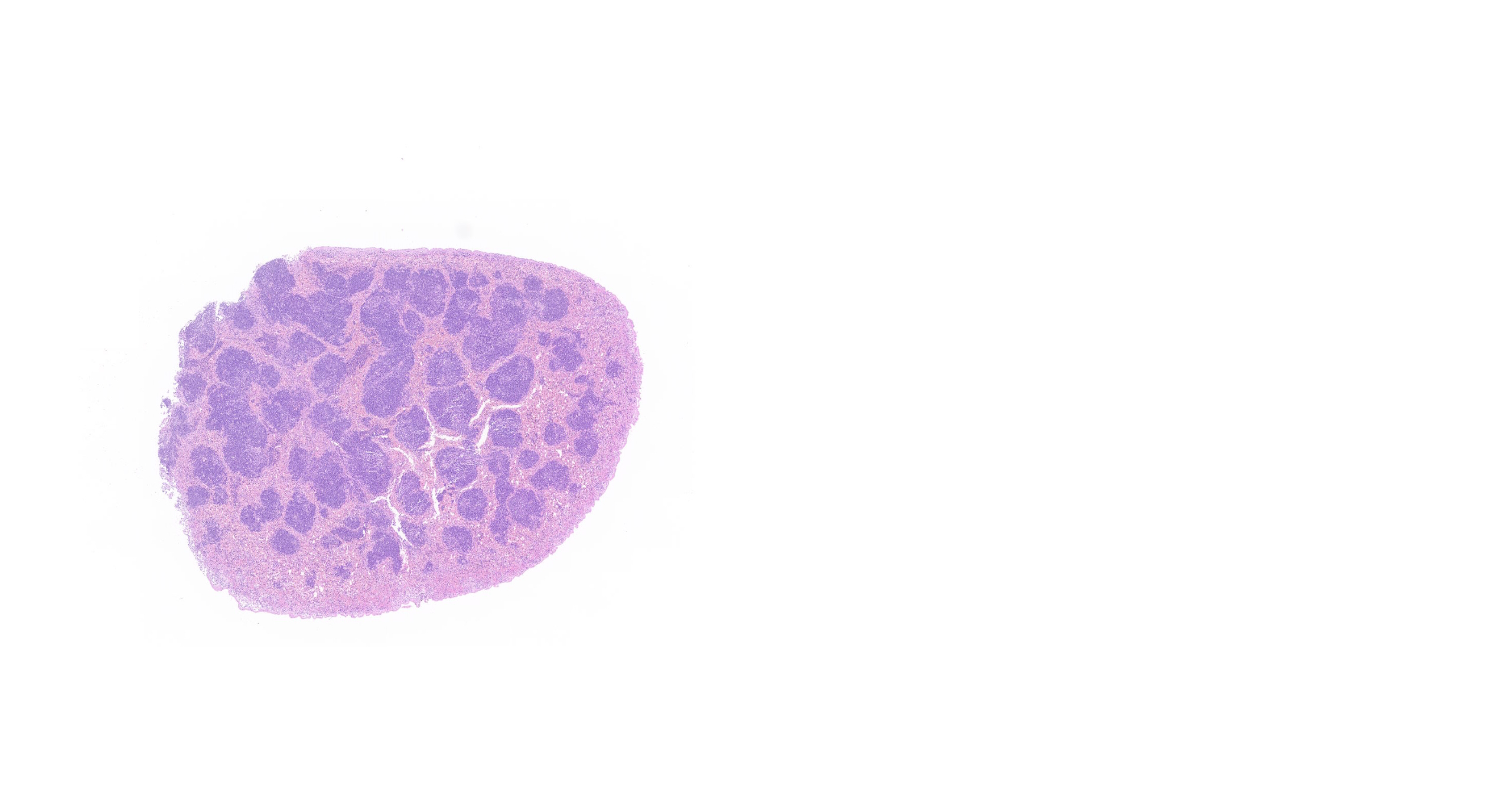

Supplement: Supplementary file 5 — Source data Fig. 4 [file 44318_2024_237_MOESM5_ESM.zip › Figure 4/Figure 4B/WT+LPS SPLEEN_2.0x.tif]

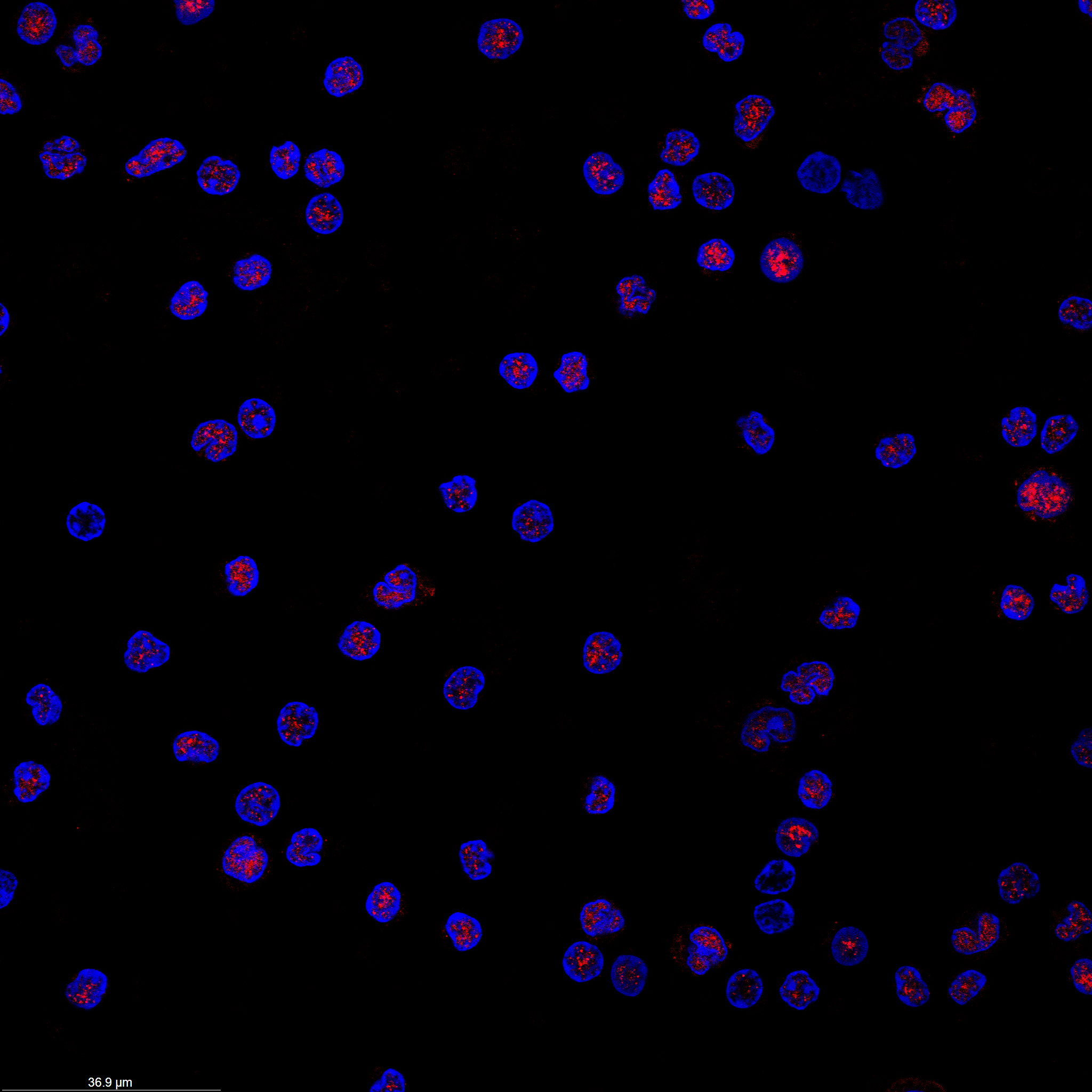

Supplement: Supplementary file 7 — Source data Fig. 6D-H [file 44318_2024_237_MOESM7_ESM.zip › Figure 6D-H/Figure 6F/nnn4-13.tif]

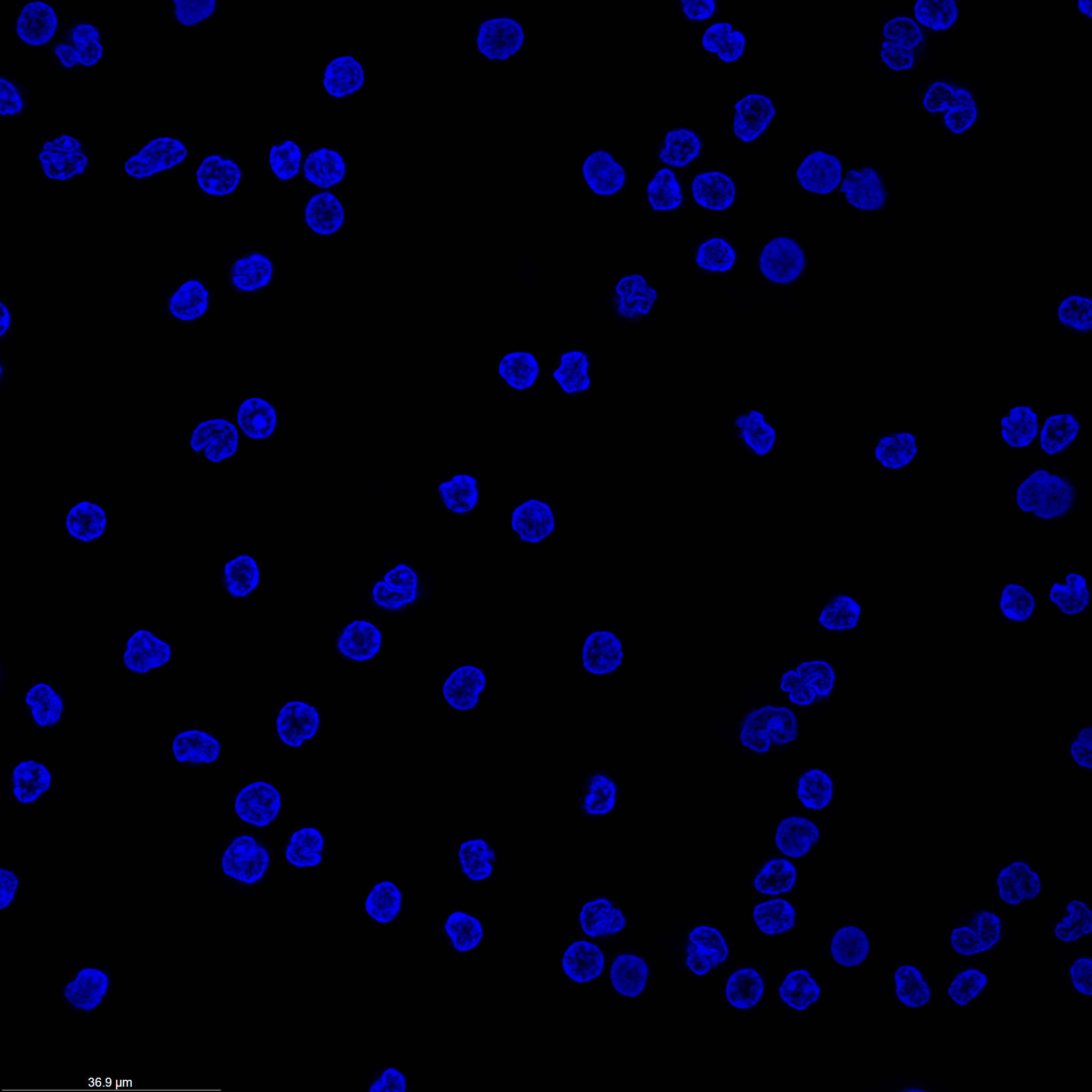

Supplement: Supplementary file 7 — Source data Fig. 6D-H [file 44318_2024_237_MOESM7_ESM.zip › Figure 6D-H/Figure 6F/nnn4-13_ch00.tif]

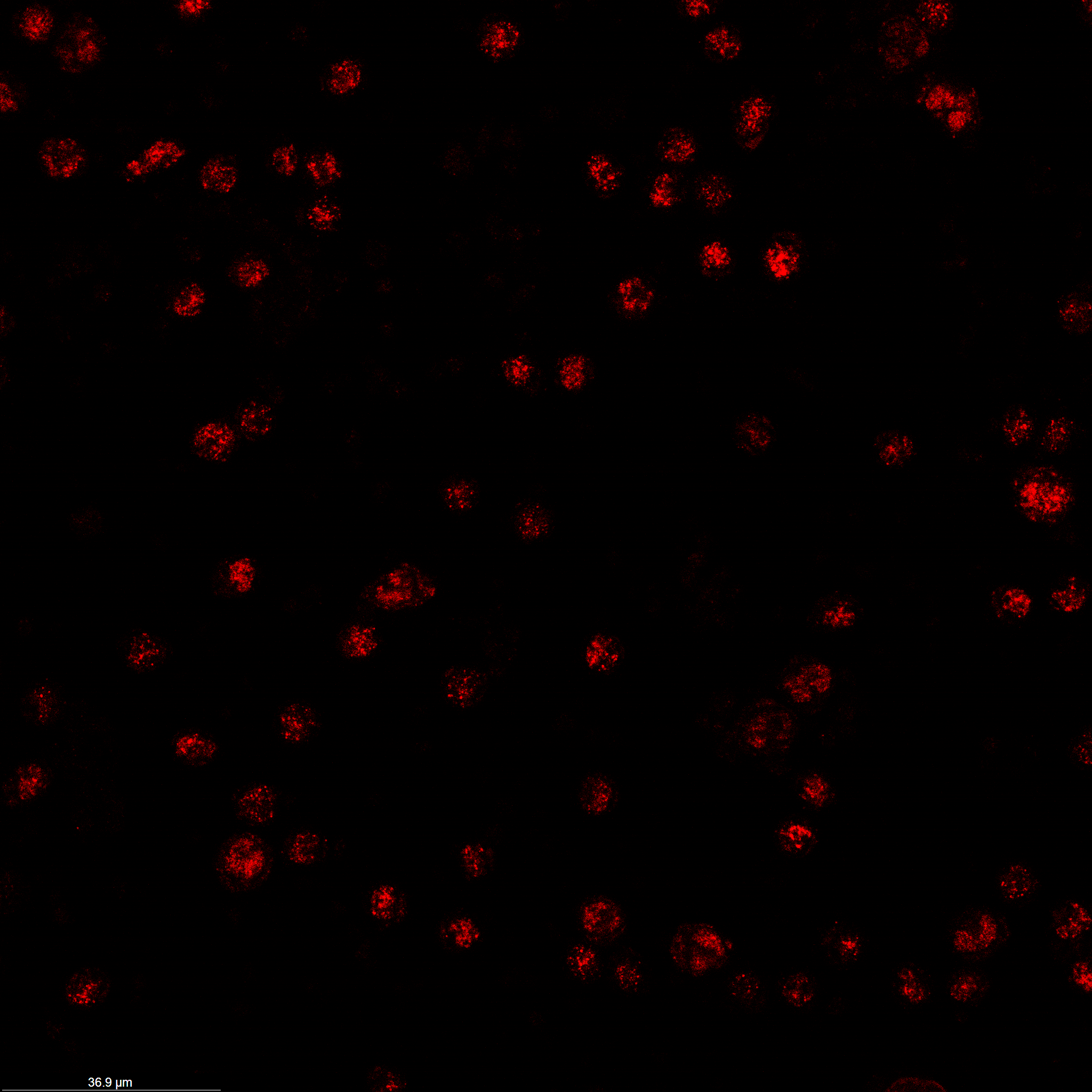

Supplement: Supplementary file 7 — Source data Fig. 6D-H [file 44318_2024_237_MOESM7_ESM.zip › Figure 6D-H/Figure 6F/nnn4-13_ch01.tif]

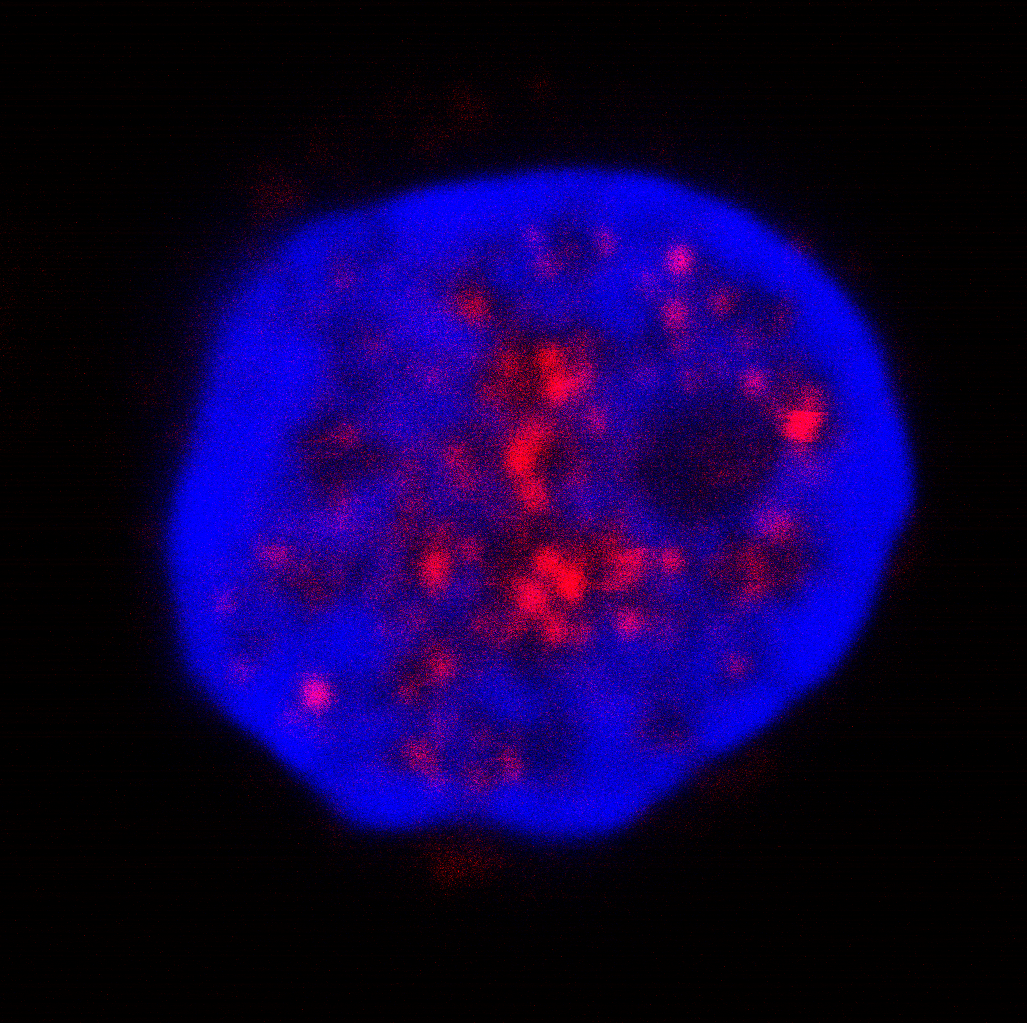

Supplement: Supplementary file 7 — Source data Fig. 6D-H [file 44318_2024_237_MOESM7_ESM.zip › Figure 6D-H/Figure 6F/nnn4-13-1.tif]

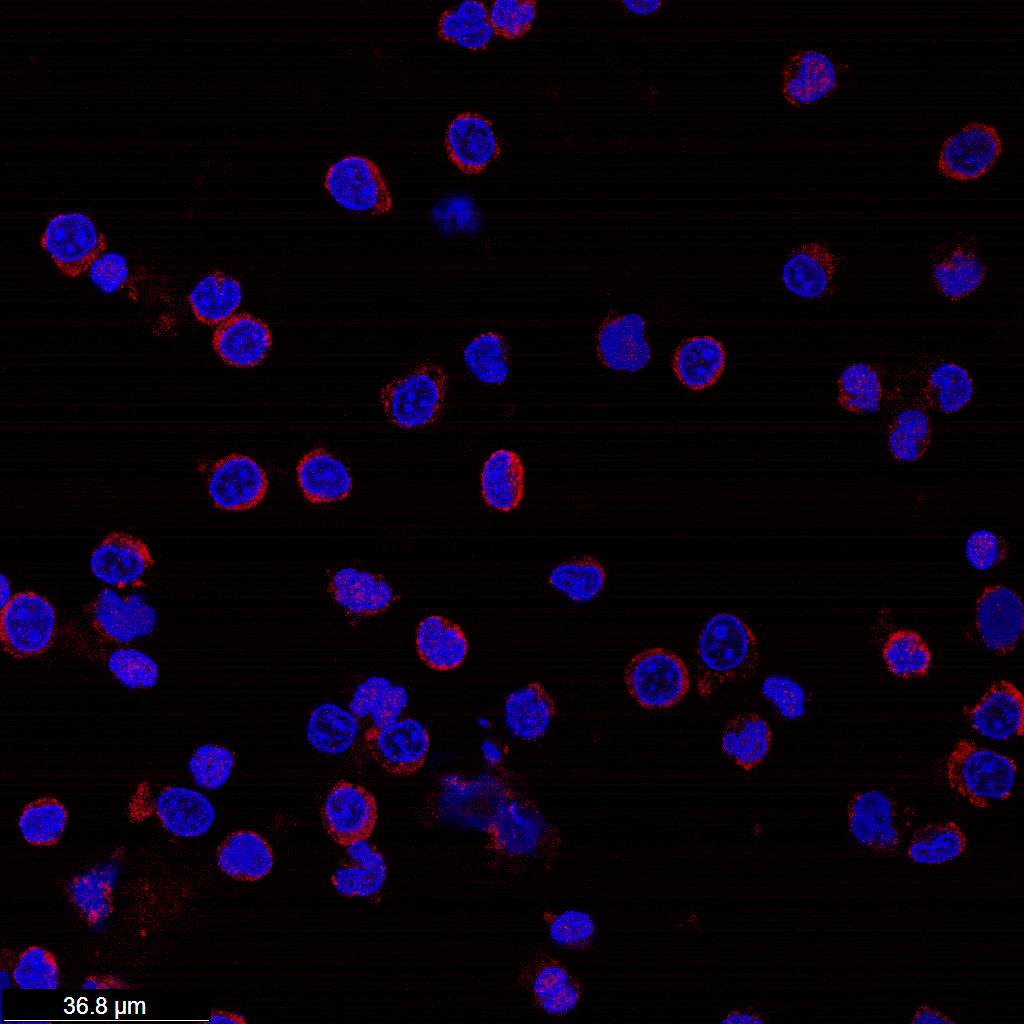

Supplement: Supplementary file 7 — Source data Fig. 6D-H [file 44318_2024_237_MOESM7_ESM.zip › Figure 6D-H/Figure 6F/S5-3.tif]

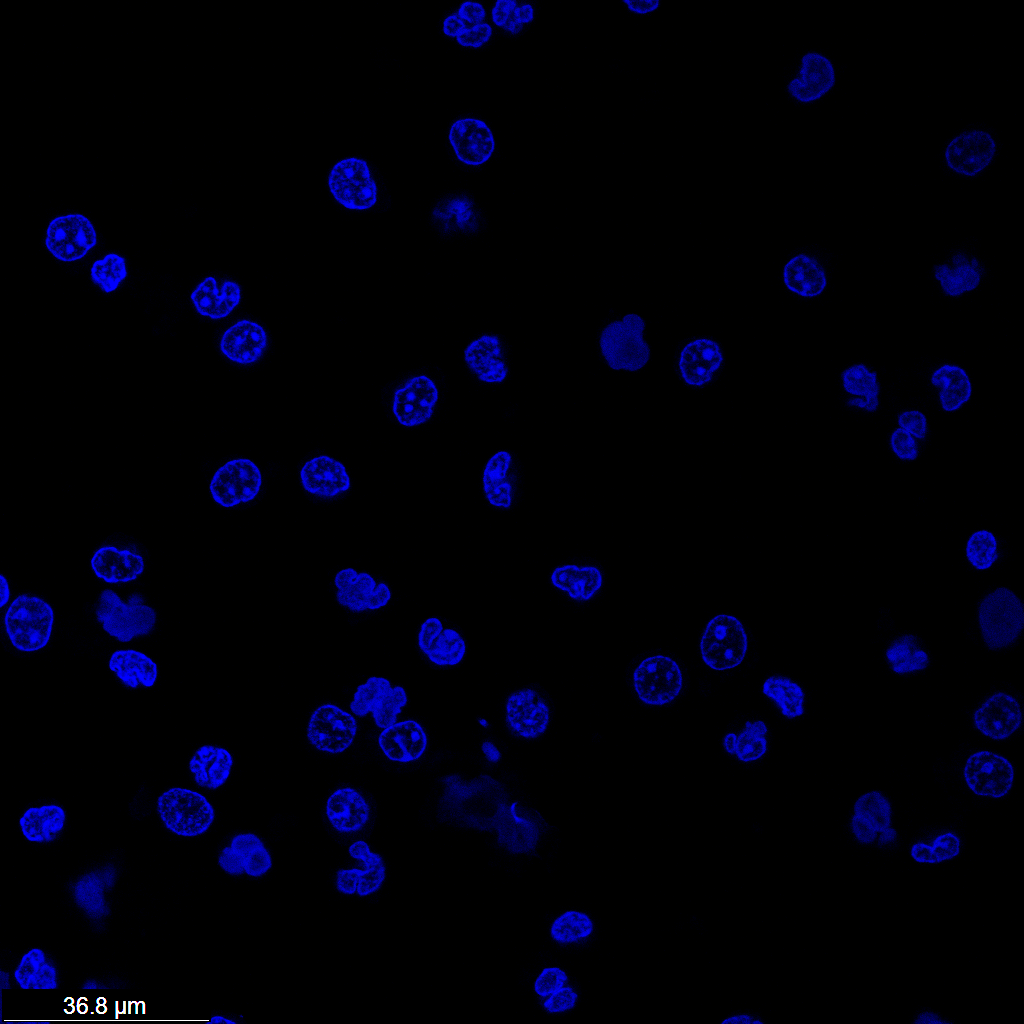

Supplement: Supplementary file 7 — Source data Fig. 6D-H [file 44318_2024_237_MOESM7_ESM.zip › Figure 6D-H/Figure 6F/S5-3_ch00.tif]

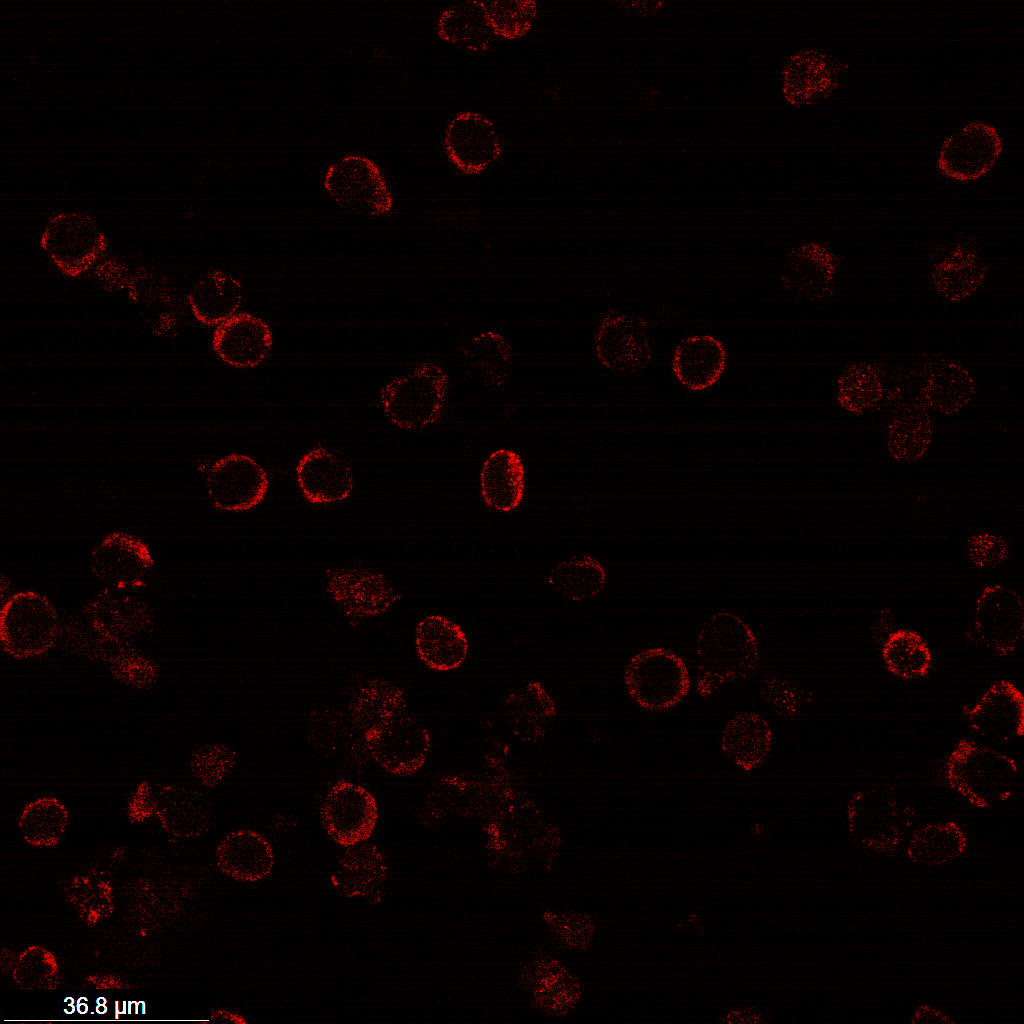

Supplement: Supplementary file 7 — Source data Fig. 6D-H [file 44318_2024_237_MOESM7_ESM.zip › Figure 6D-H/Figure 6F/S5-3_ch01.tif]

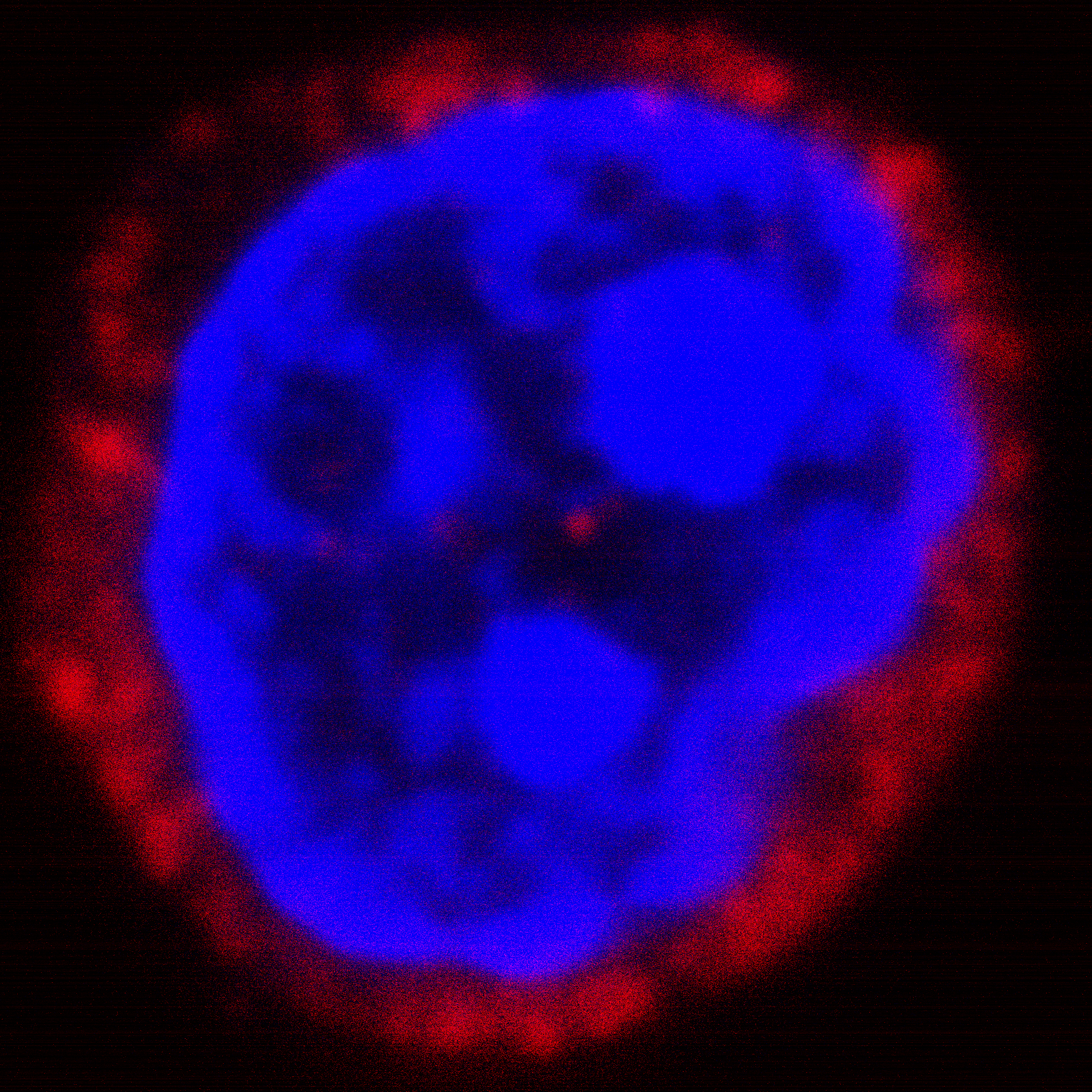

Supplement: Supplementary file 7 — Source data Fig. 6D-H [file 44318_2024_237_MOESM7_ESM.zip › Figure 6D-H/Figure 6F/S555-3.tif]

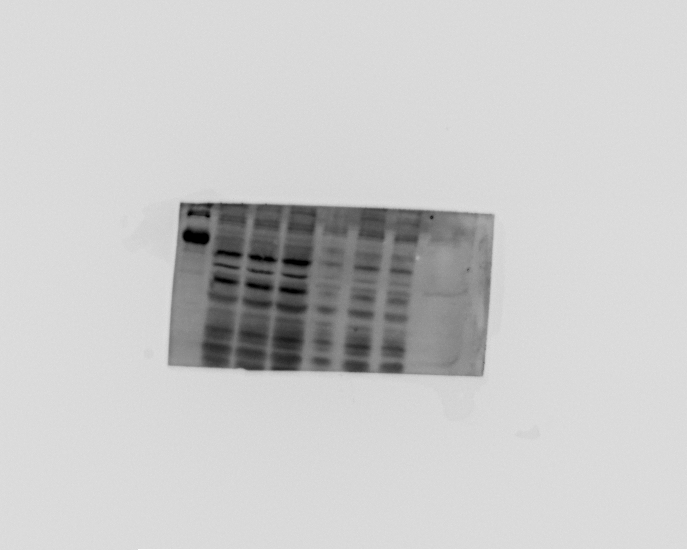

Supplement: Supplementary file 7 — Source data Fig. 6D-H [file 44318_2024_237_MOESM7_ESM.zip › Figure 6D-H/Figure 6G/[╘¡╩╝]/HDAC3-2.tif]

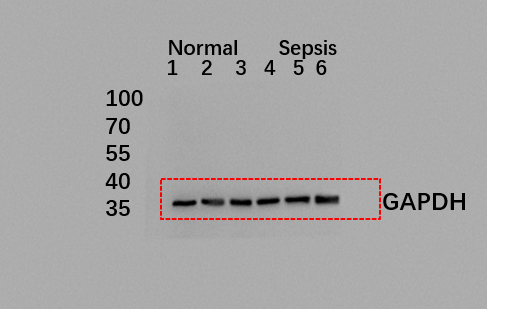

Supplement: Supplementary file 7 — Source data Fig. 6D-H [file 44318_2024_237_MOESM7_ESM.zip › Figure 6D-H/Figure 6G/GAPDH.tif]

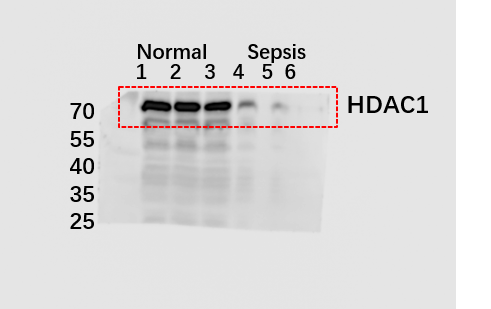

Supplement: Supplementary file 7 — Source data Fig. 6D-H [file 44318_2024_237_MOESM7_ESM.zip › Figure 6D-H/Figure 6G/HDAC1-1.tif]

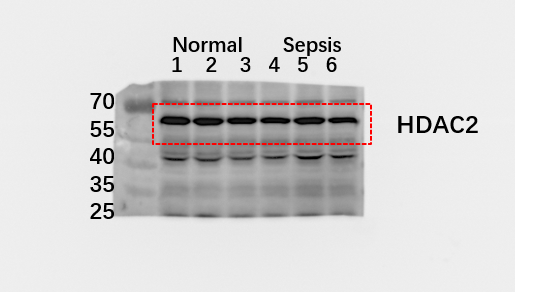

Supplement: Supplementary file 7 — Source data Fig. 6D-H [file 44318_2024_237_MOESM7_ESM.zip › Figure 6D-H/Figure 6G/HDAC2-1.tif]

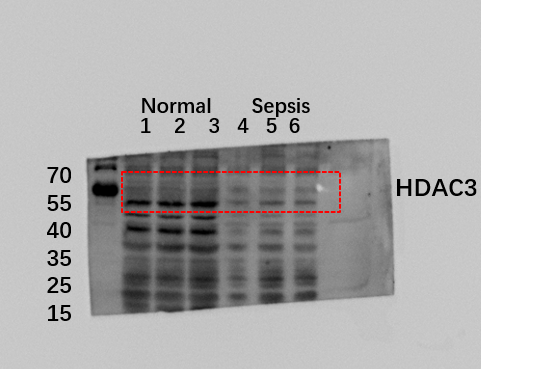

Supplement: Supplementary file 7 — Source data Fig. 6D-H [file 44318_2024_237_MOESM7_ESM.zip › Figure 6D-H/Figure 6G/HDAC3-1.tif]

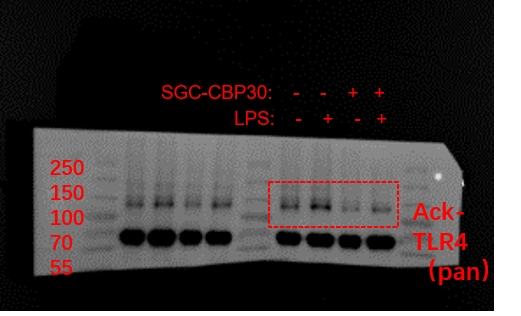

Supplement: Supplementary file 8 — Source data Fig. 7 [file 44318_2024_237_MOESM8_ESM.zip › Figure 7/Figure 7D/acK-TLR4 PAN.tif]

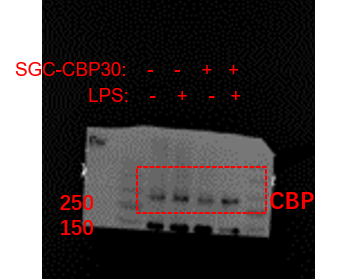

Supplement: Supplementary file 8 — Source data Fig. 7 [file 44318_2024_237_MOESM8_ESM.zip › Figure 7/Figure 7D/CBP.tif]

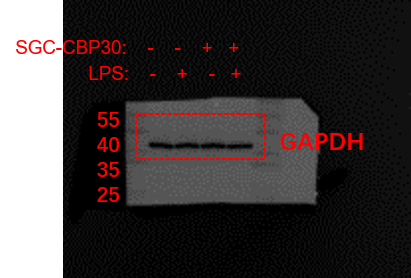

Supplement: Supplementary file 8 — Source data Fig. 7 [file 44318_2024_237_MOESM8_ESM.zip › Figure 7/Figure 7D/gapdh.tif]

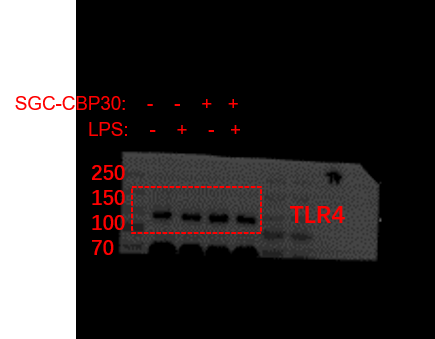

Supplement: Supplementary file 8 — Source data Fig. 7 [file 44318_2024_237_MOESM8_ESM.zip › Figure 7/Figure 7D/TLR4.tif]

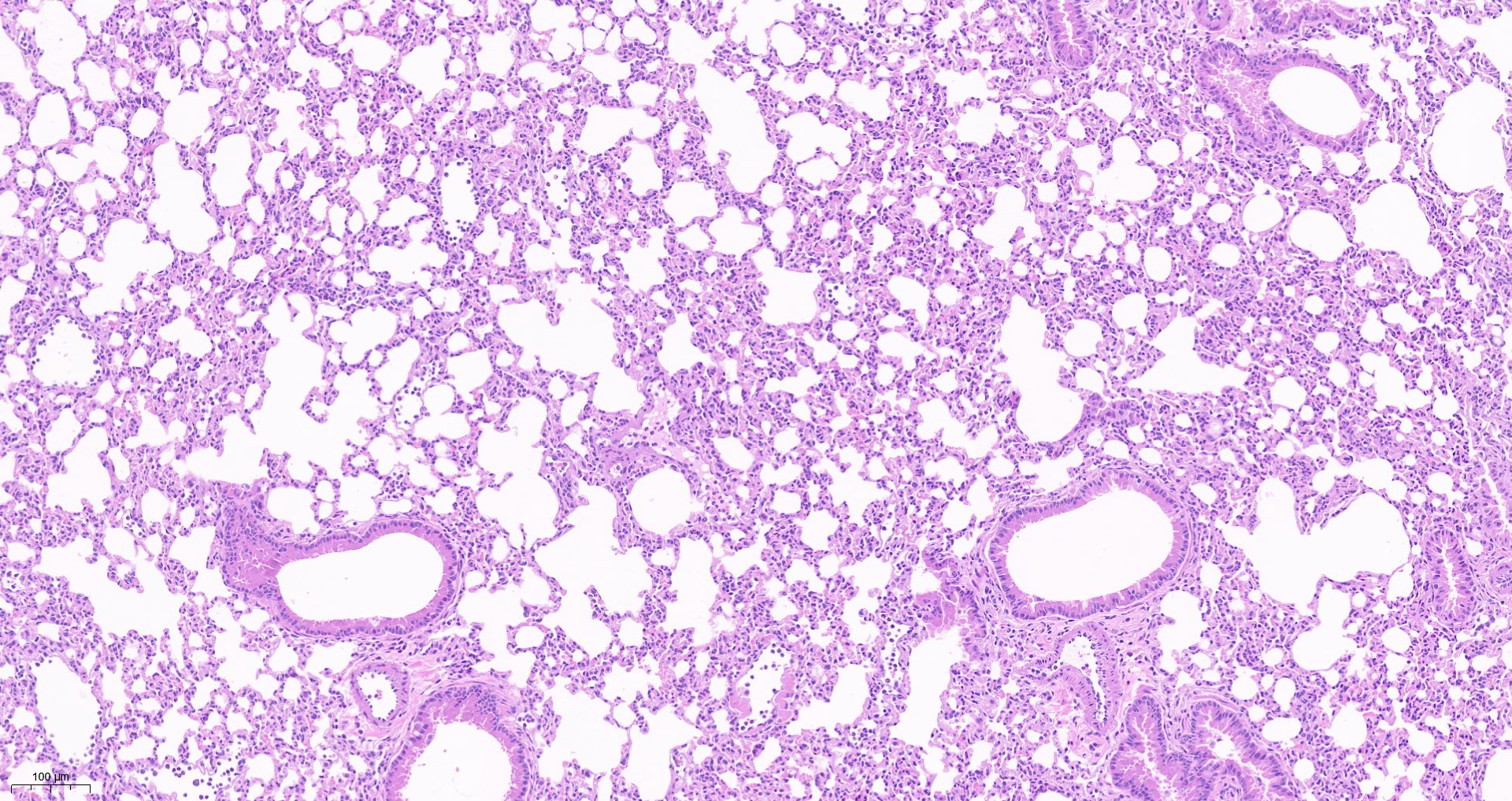

Supplement: Supplementary file 8 — Source data Fig. 7 [file 44318_2024_237_MOESM8_ESM.zip › Figure 7/Figure 7F/LPS_10.0x lung.tif]

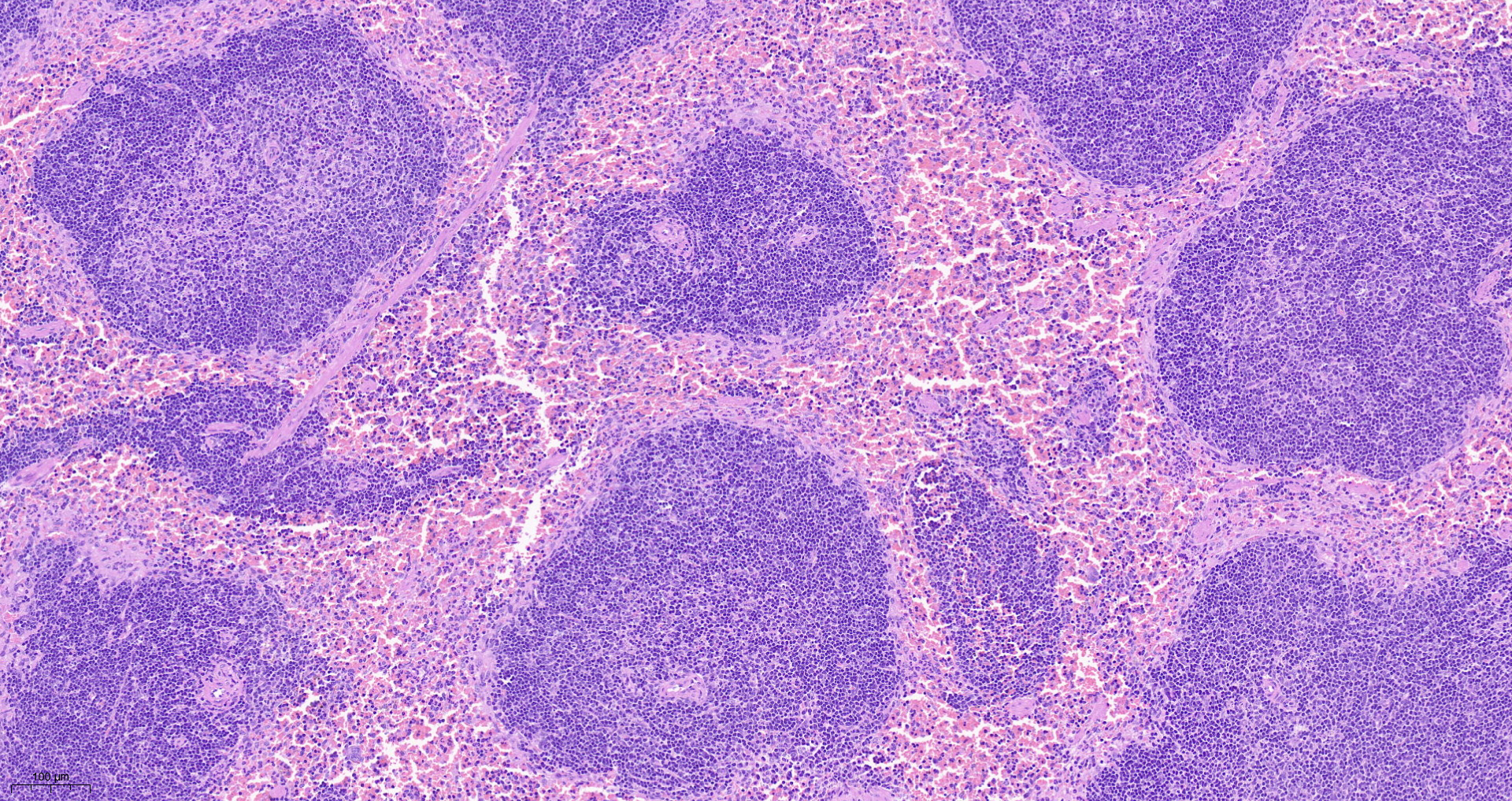

Supplement: Supplementary file 8 — Source data Fig. 7 [file 44318_2024_237_MOESM8_ESM.zip › Figure 7/Figure 7F/LPS_10.0x spleen.tif]

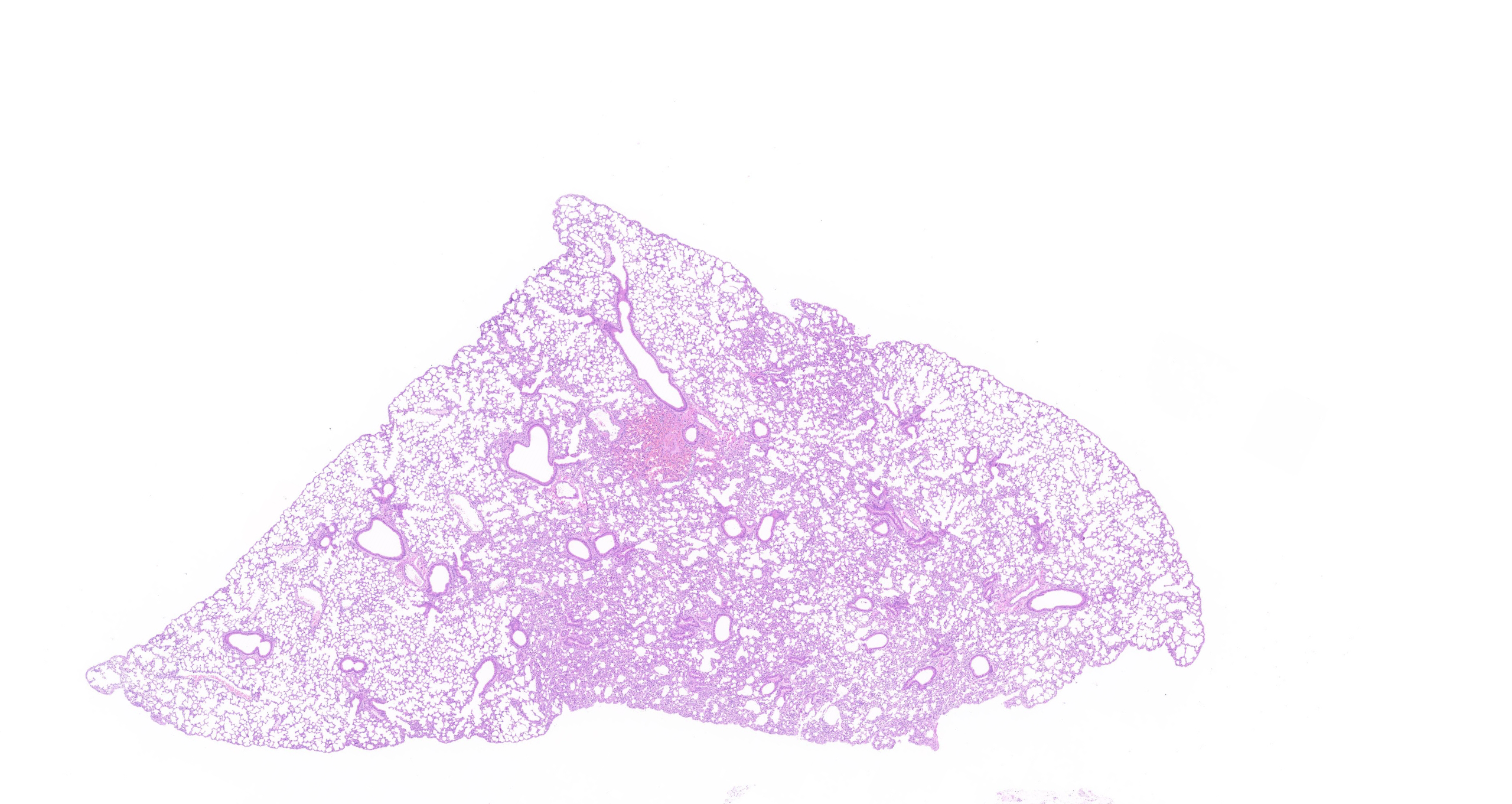

Supplement: Supplementary file 8 — Source data Fig. 7 [file 44318_2024_237_MOESM8_ESM.zip › Figure 7/Figure 7F/LPS_2.0x lung.tif]

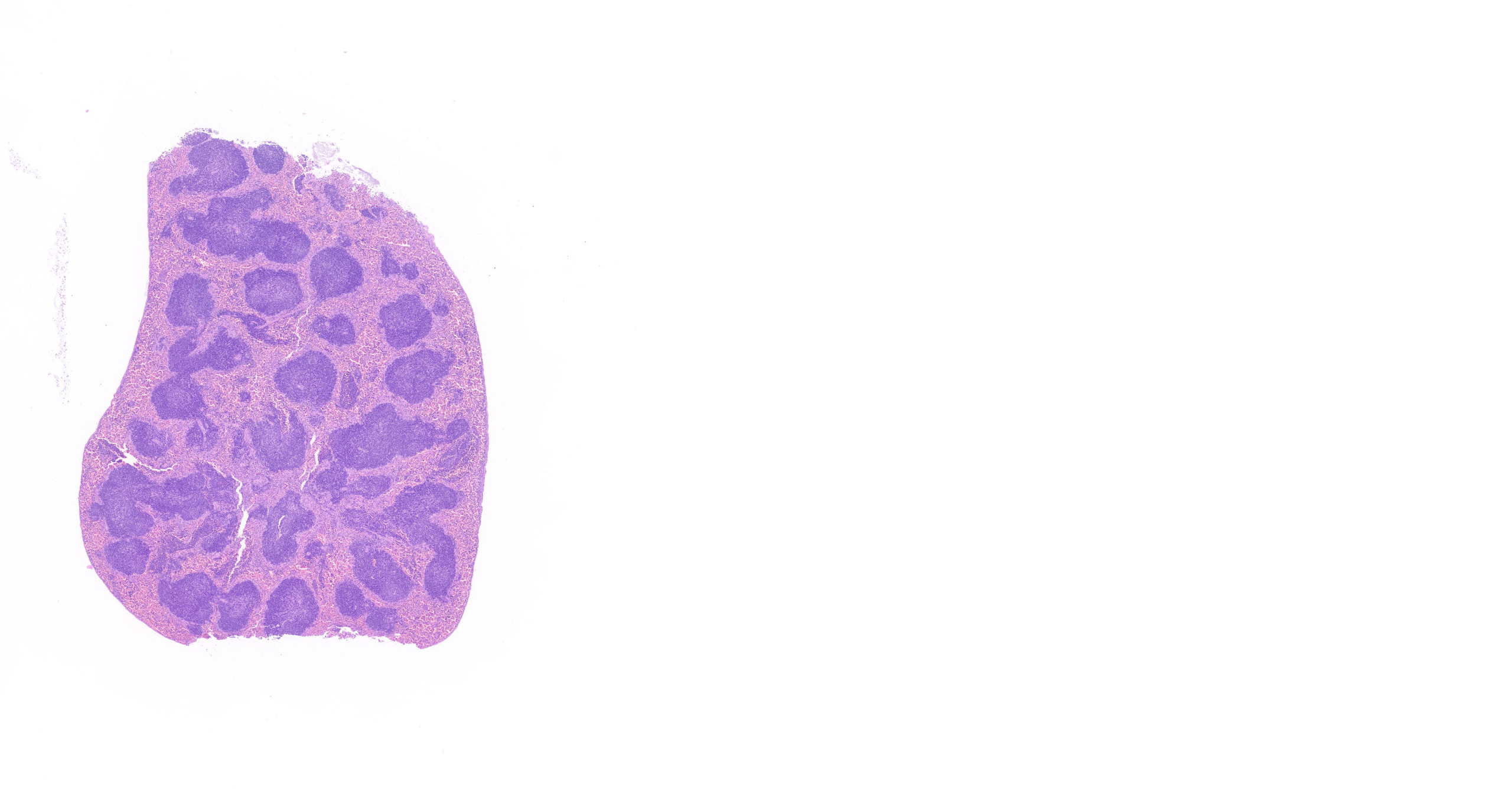

Supplement: Supplementary file 8 — Source data Fig. 7 [file 44318_2024_237_MOESM8_ESM.zip › Figure 7/Figure 7F/LPS_2.0x spleen.tif]

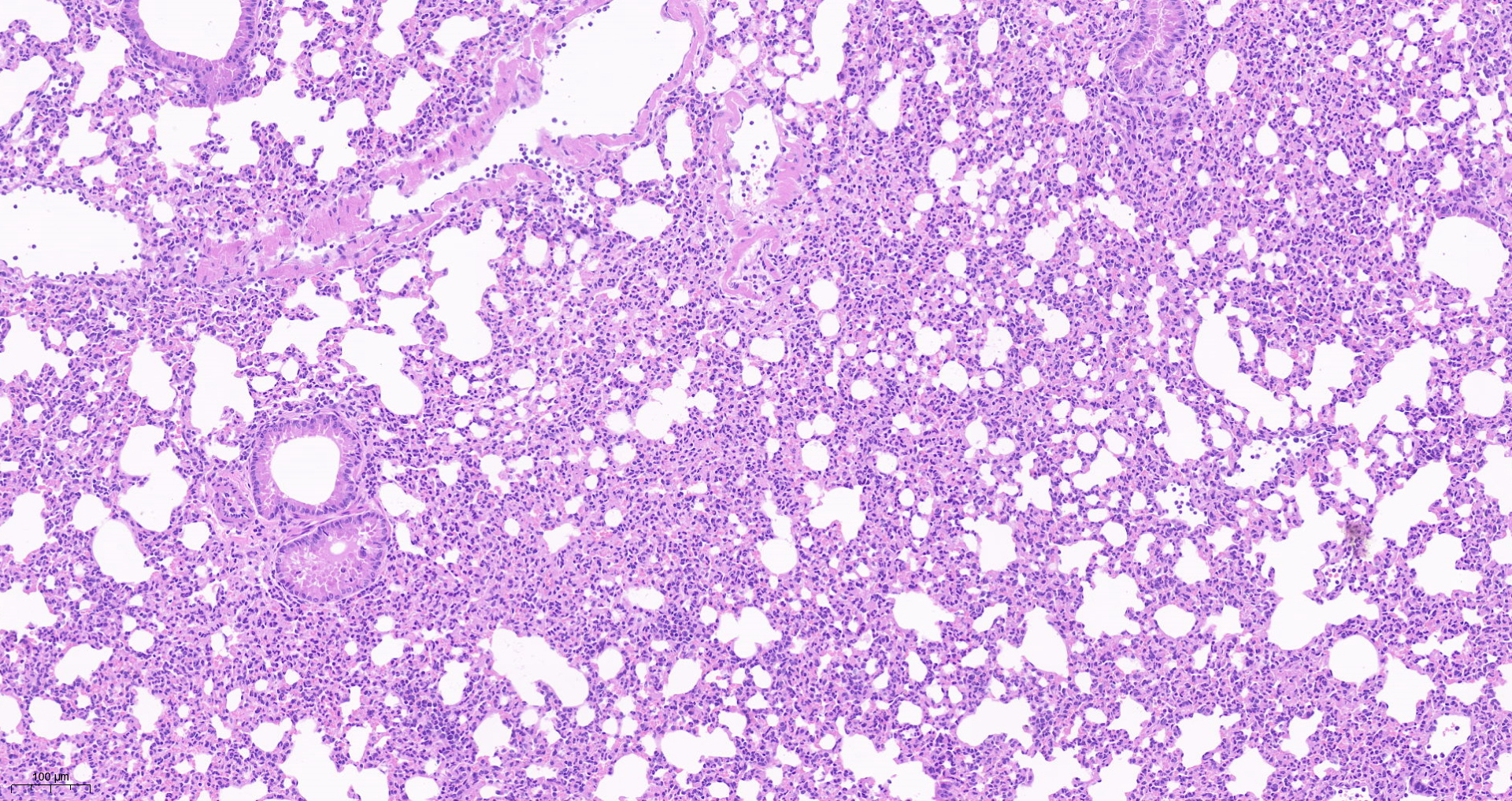

Supplement: Supplementary file 8 — Source data Fig. 7 [file 44318_2024_237_MOESM8_ESM.zip › Figure 7/Figure 7F/LPS+RG2833_10.0x lung.tif]

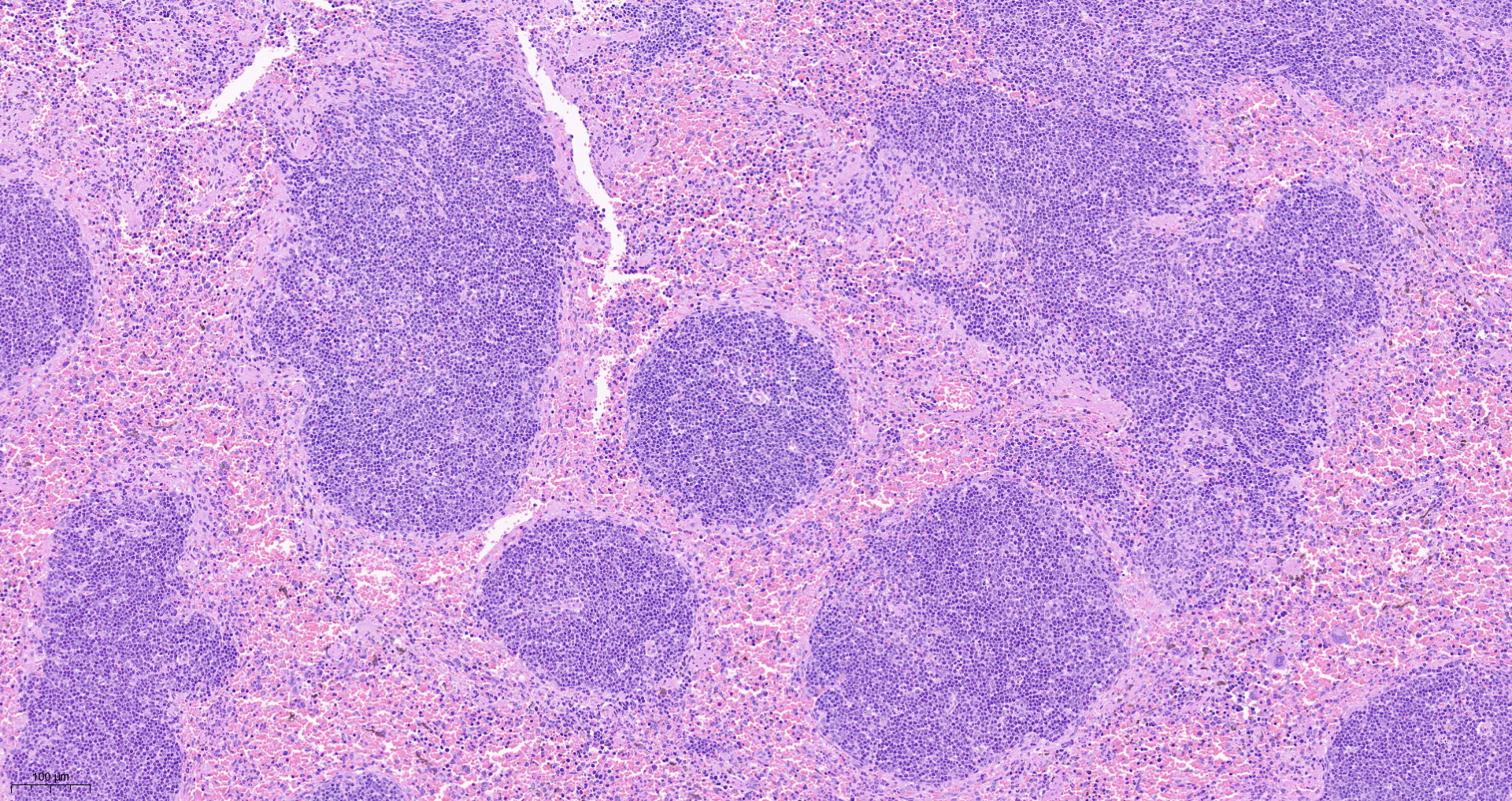

Supplement: Supplementary file 8 — Source data Fig. 7 [file 44318_2024_237_MOESM8_ESM.zip › Figure 7/Figure 7F/LPS+RG2833_10.0x spleen.tif]

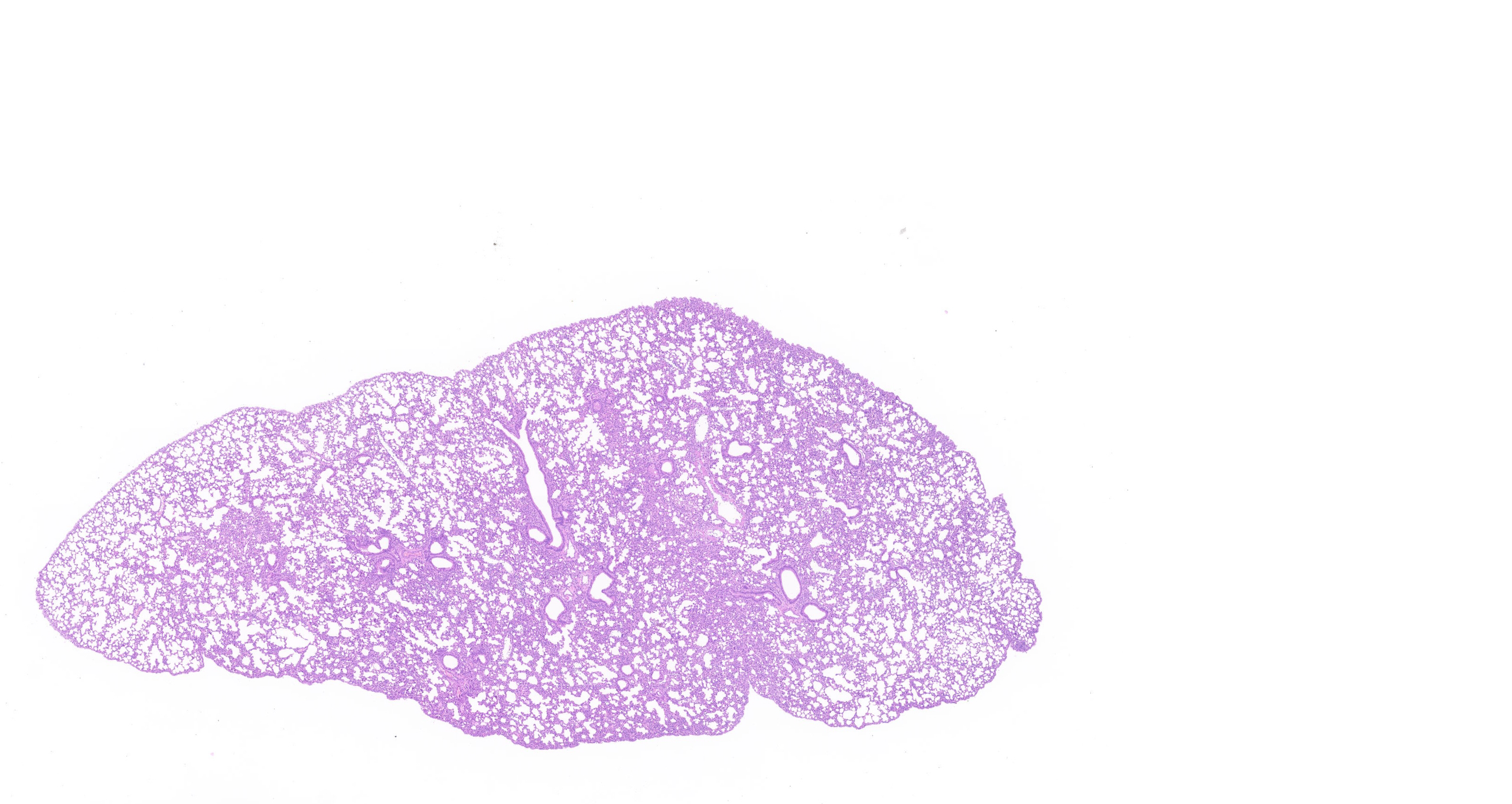

Supplement: Supplementary file 8 — Source data Fig. 7 [file 44318_2024_237_MOESM8_ESM.zip › Figure 7/Figure 7F/LPS+RG2833_2.0x lung.tif]

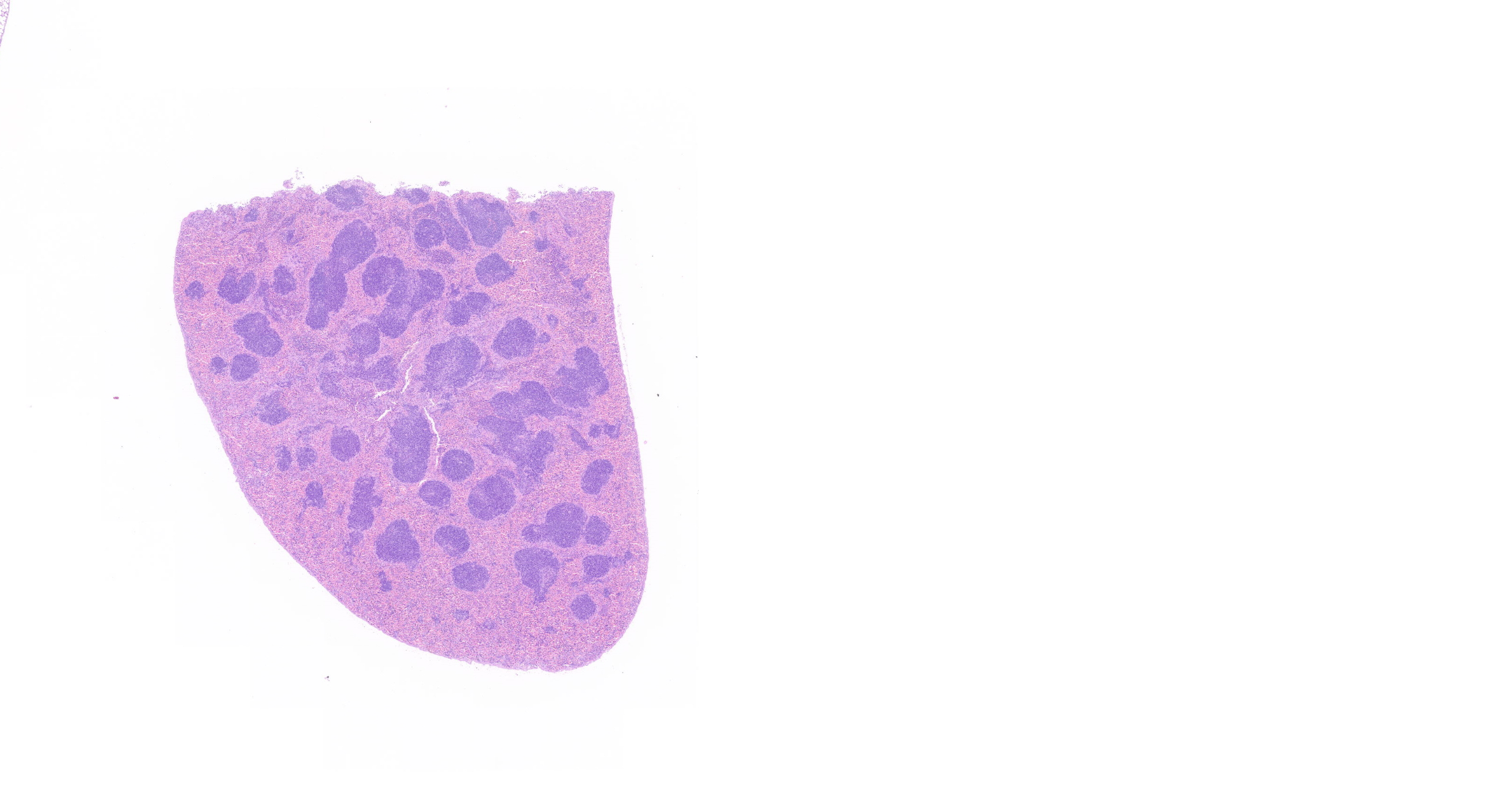

Supplement: Supplementary file 8 — Source data Fig. 7 [file 44318_2024_237_MOESM8_ESM.zip › Figure 7/Figure 7F/LPS+RG2833_2.0x spleen.tif]

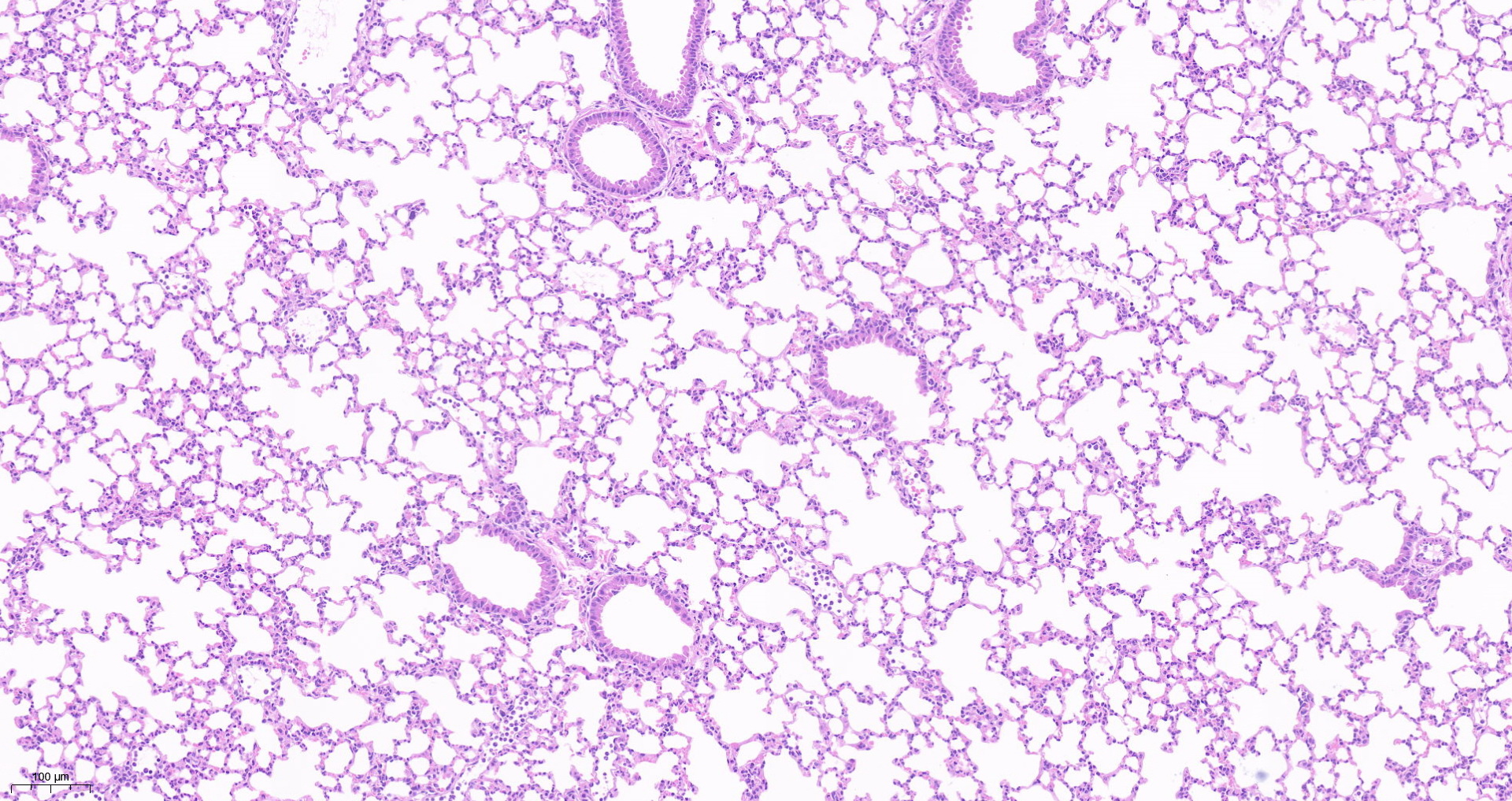

Supplement: Supplementary file 8 — Source data Fig. 7 [file 44318_2024_237_MOESM8_ESM.zip › Figure 7/Figure 7F/LPS+SGC-CBP30_10.0x lung.jpg]

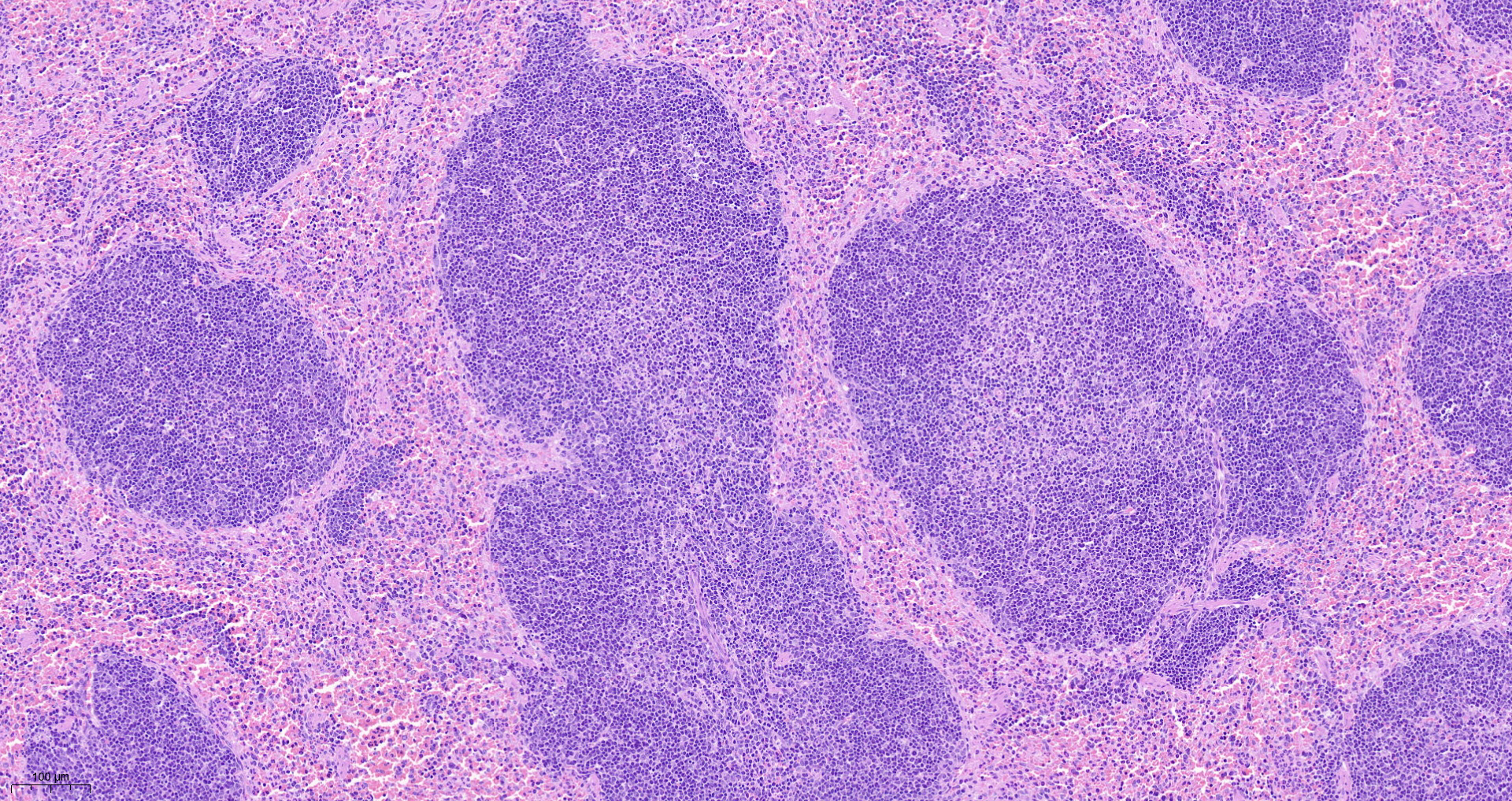

Supplement: Supplementary file 8 — Source data Fig. 7 [file 44318_2024_237_MOESM8_ESM.zip › Figure 7/Figure 7F/LPS+SGC-CBP30_10.0x spleen.tif]

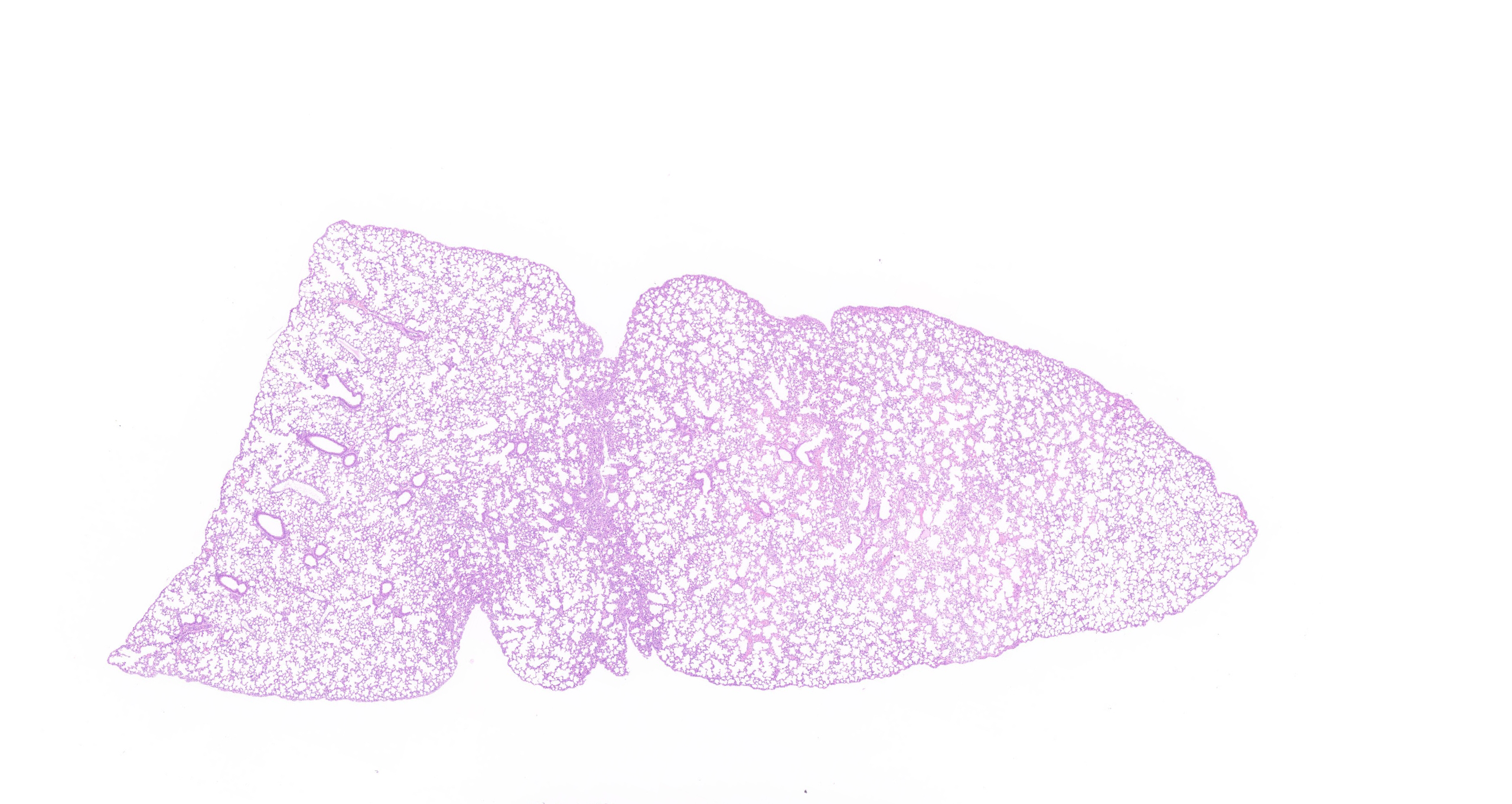

Supplement: Supplementary file 8 — Source data Fig. 7 [file 44318_2024_237_MOESM8_ESM.zip › Figure 7/Figure 7F/LPS+SGC-CBP30_2.0x lung.tif]

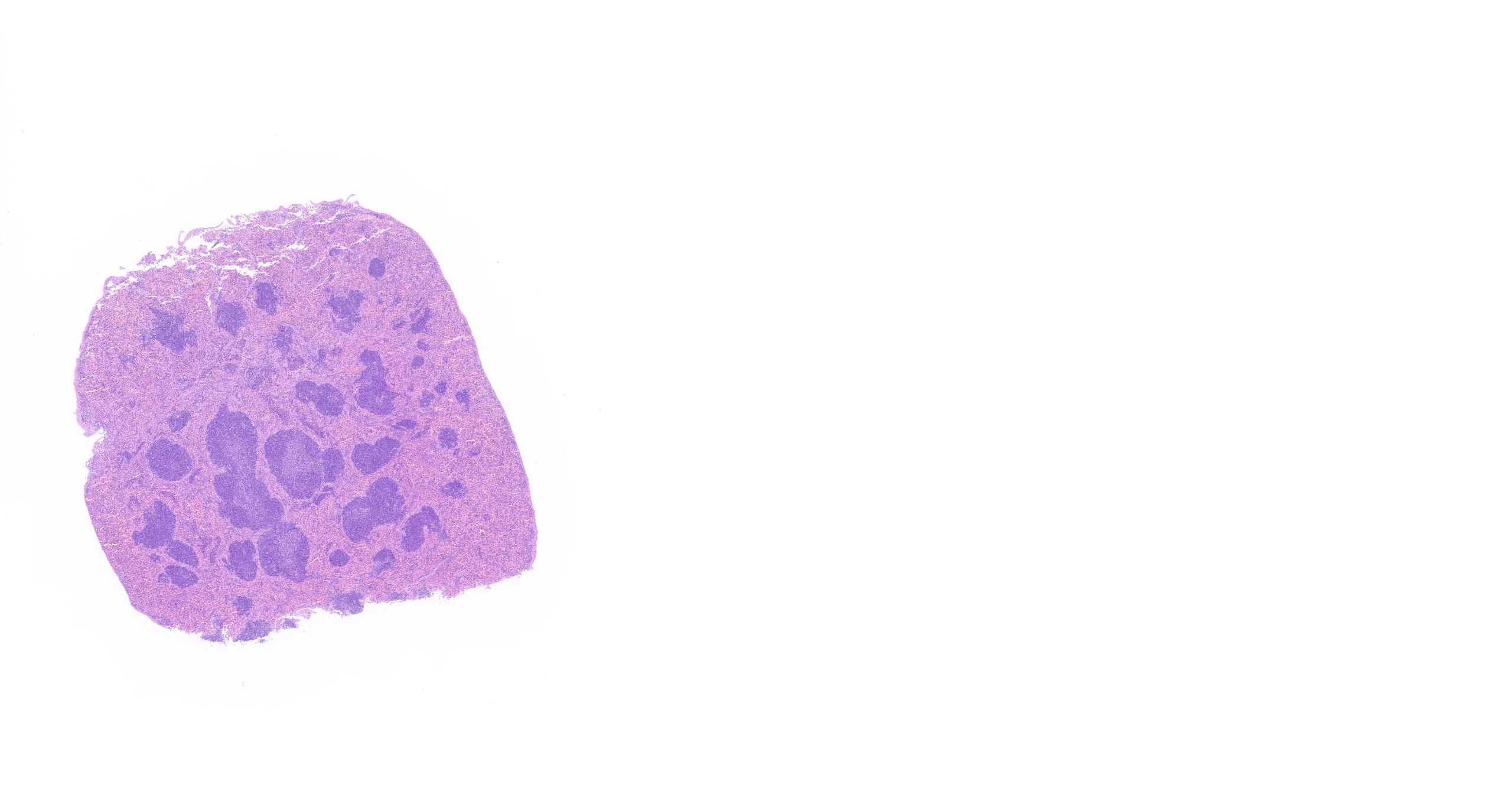

Supplement: Supplementary file 8 — Source data Fig. 7 [file 44318_2024_237_MOESM8_ESM.zip › Figure 7/Figure 7F/LPS+SGC-CBP30_2.0x spleen.tif]

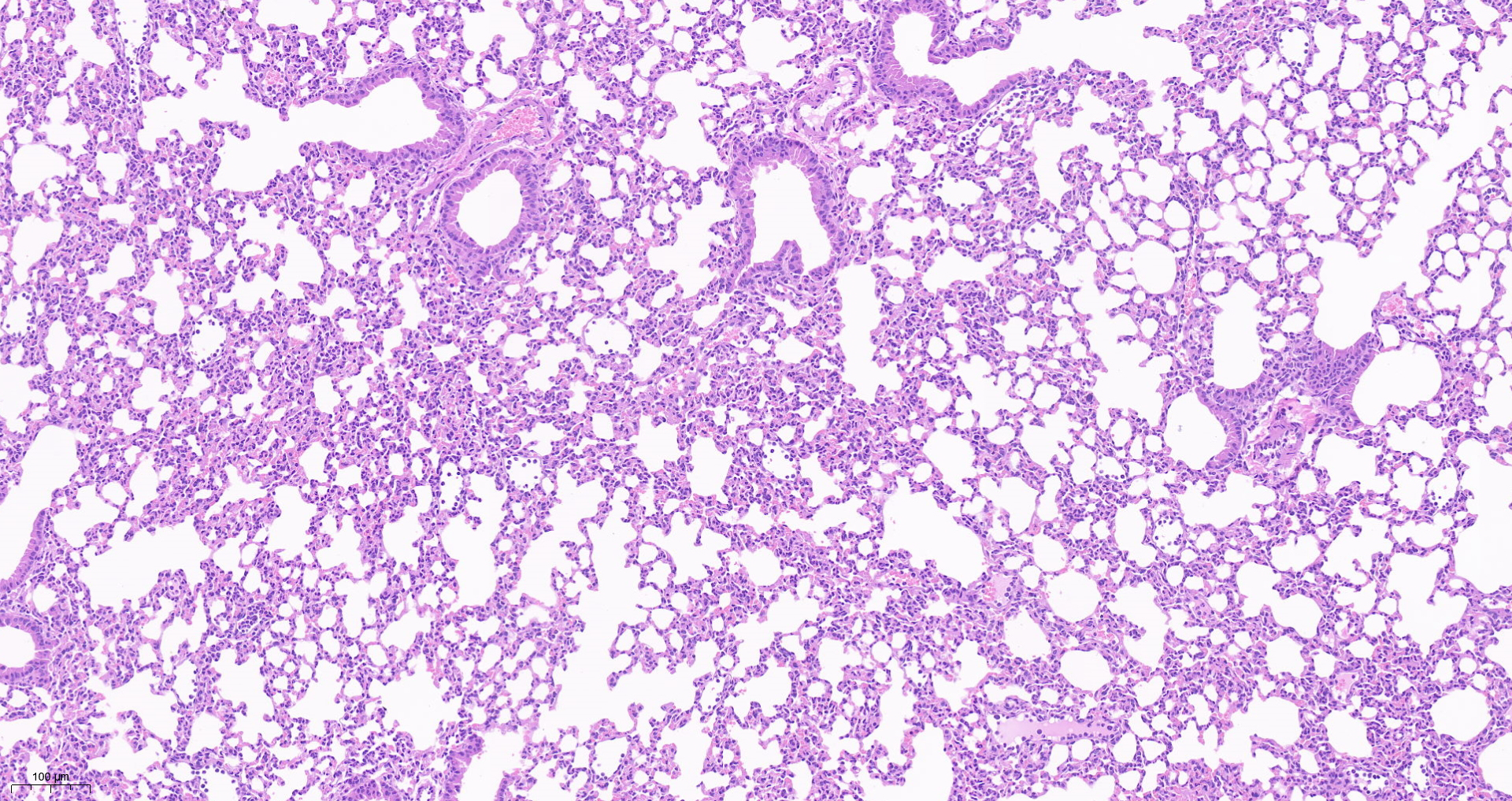

Supplement: Supplementary file 8 — Source data Fig. 7 [file 44318_2024_237_MOESM8_ESM.zip › Figure 7/Figure 7F/LPS+TSA_10.0x lung.tif]

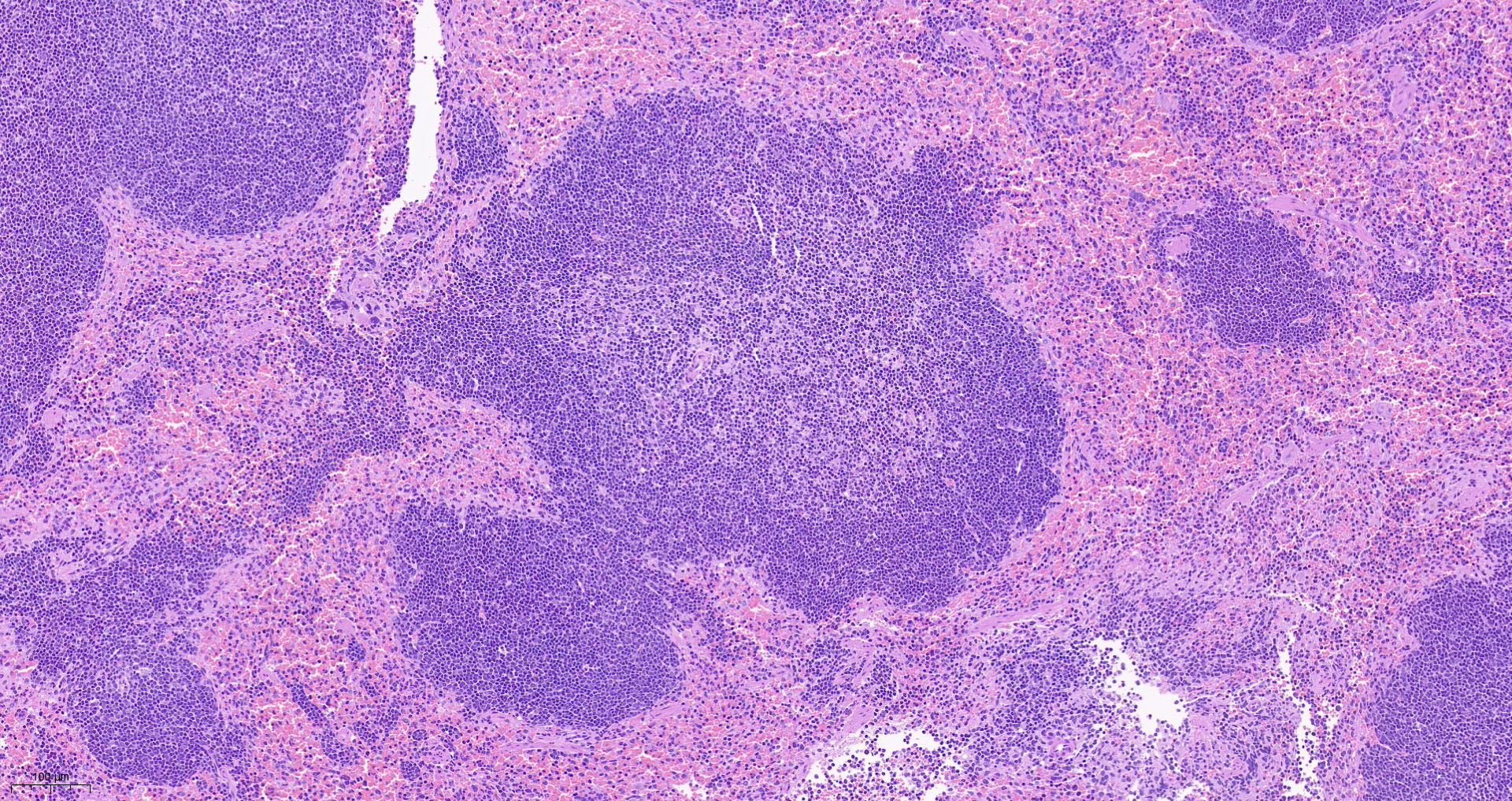

Supplement: Supplementary file 8 — Source data Fig. 7 [file 44318_2024_237_MOESM8_ESM.zip › Figure 7/Figure 7F/LPS+TSA_10.0x spleen.tif]

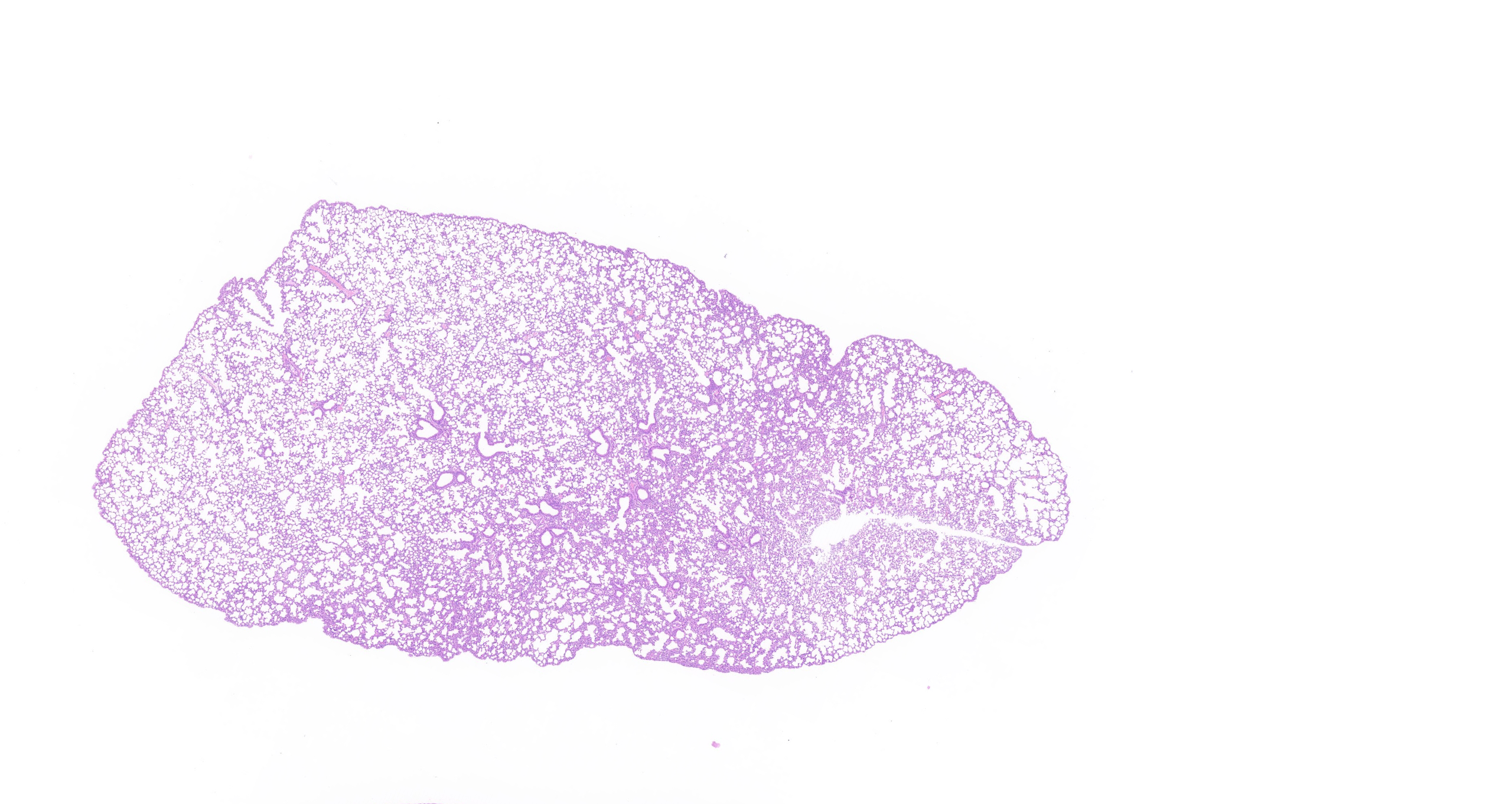

Supplement: Supplementary file 8 — Source data Fig. 7 [file 44318_2024_237_MOESM8_ESM.zip › Figure 7/Figure 7F/LPS+TSA_2.0x lung.tif]

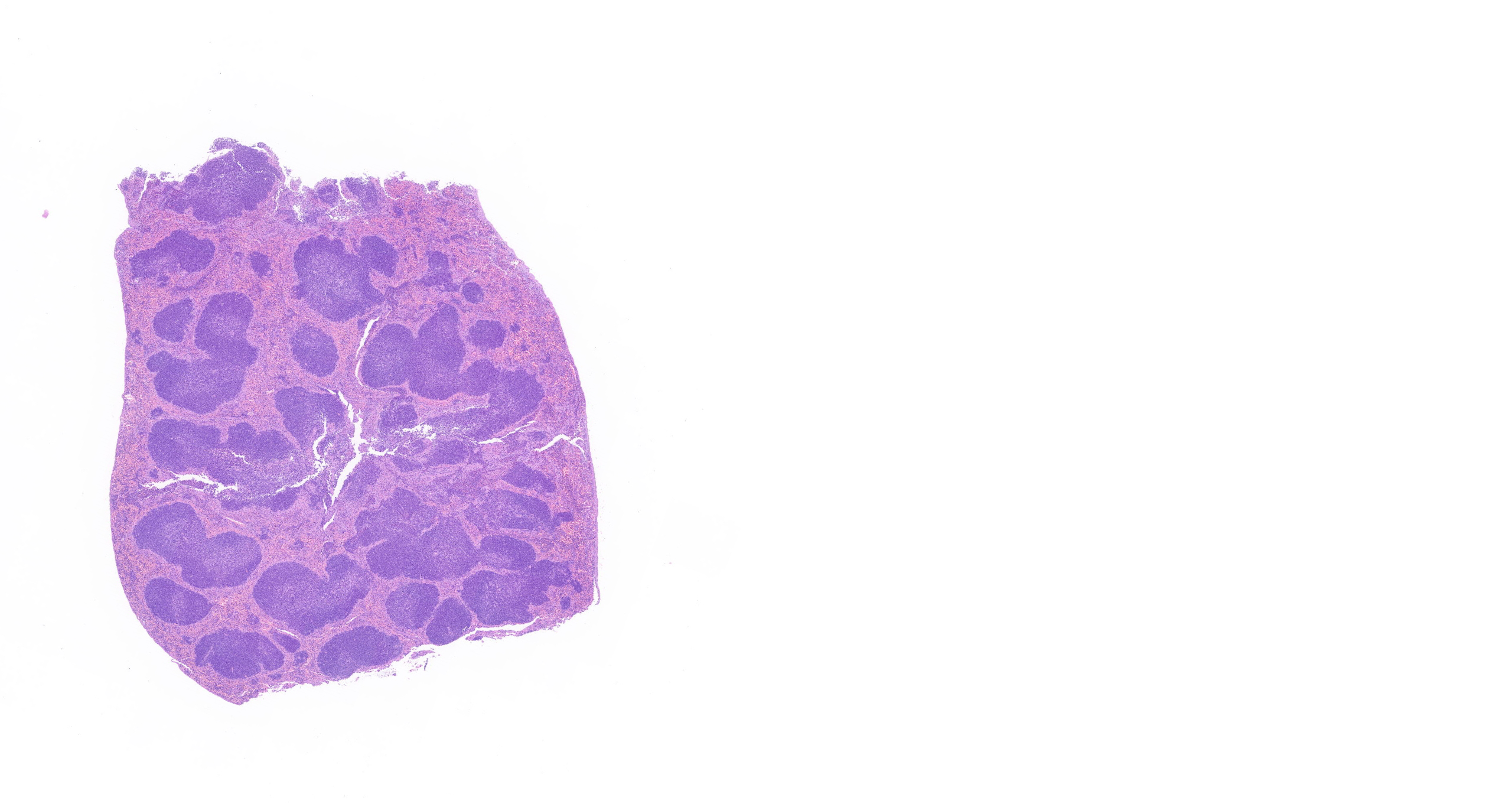

Supplement: Supplementary file 8 — Source data Fig. 7 [file 44318_2024_237_MOESM8_ESM.zip › Figure 7/Figure 7F/LPS+TSA_2.0x spleen.tif]

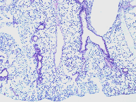

Supplement: Supplementary file 9 — EV Figure Source Data [file 44318_2024_237_MOESM9_ESM.zip › Figure EV3/Figure EV3C/K730R lung.tif]

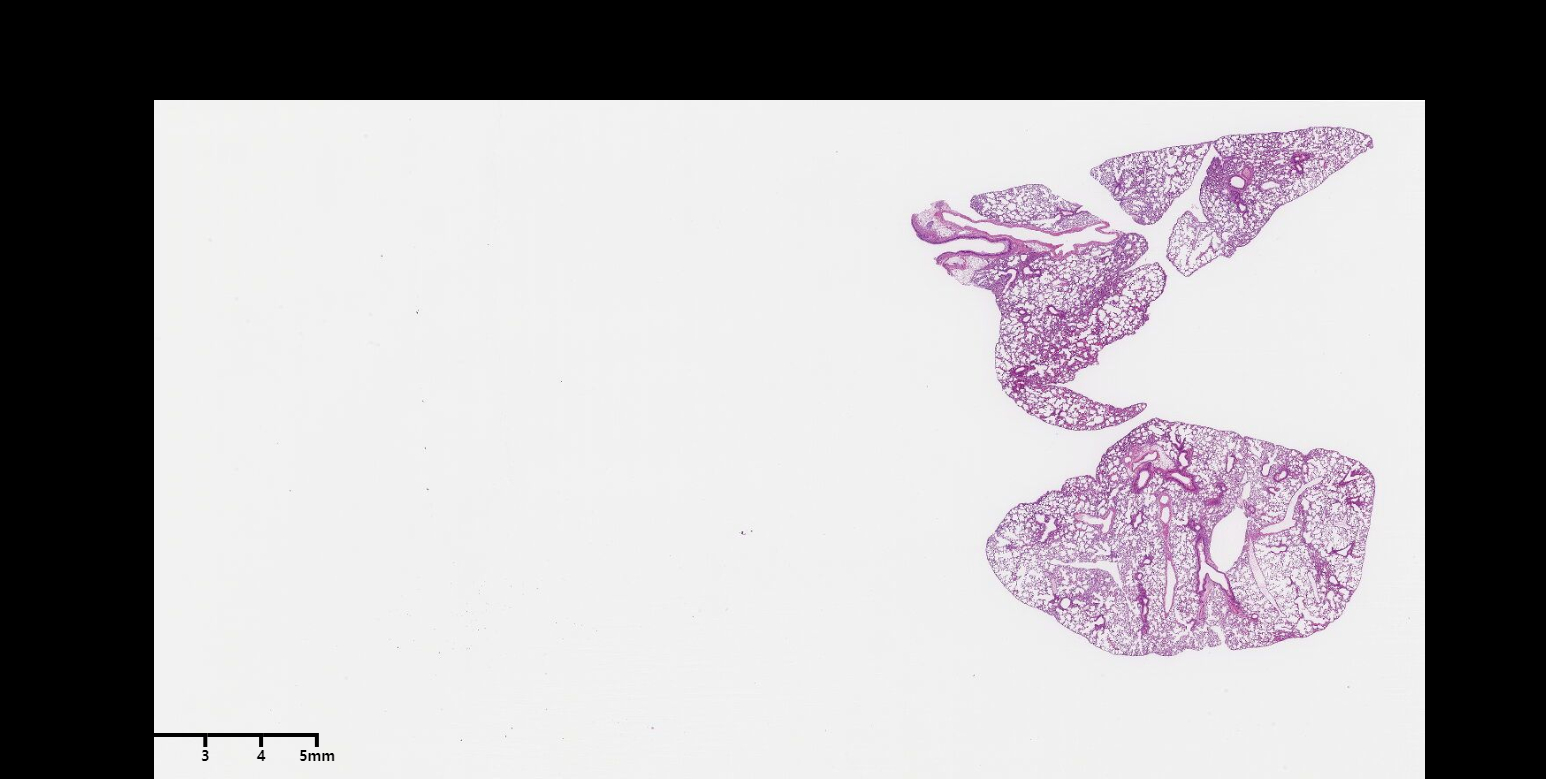

Supplement: Supplementary file 9 — EV Figure Source Data [file 44318_2024_237_MOESM9_ESM.zip › Figure EV3/Figure EV3C/K730R lung_0.50X_20240705033059.tif]

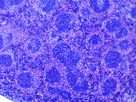

Supplement: Supplementary file 9 — EV Figure Source Data [file 44318_2024_237_MOESM9_ESM.zip › Figure EV3/Figure EV3C/K730R spleen.tif]

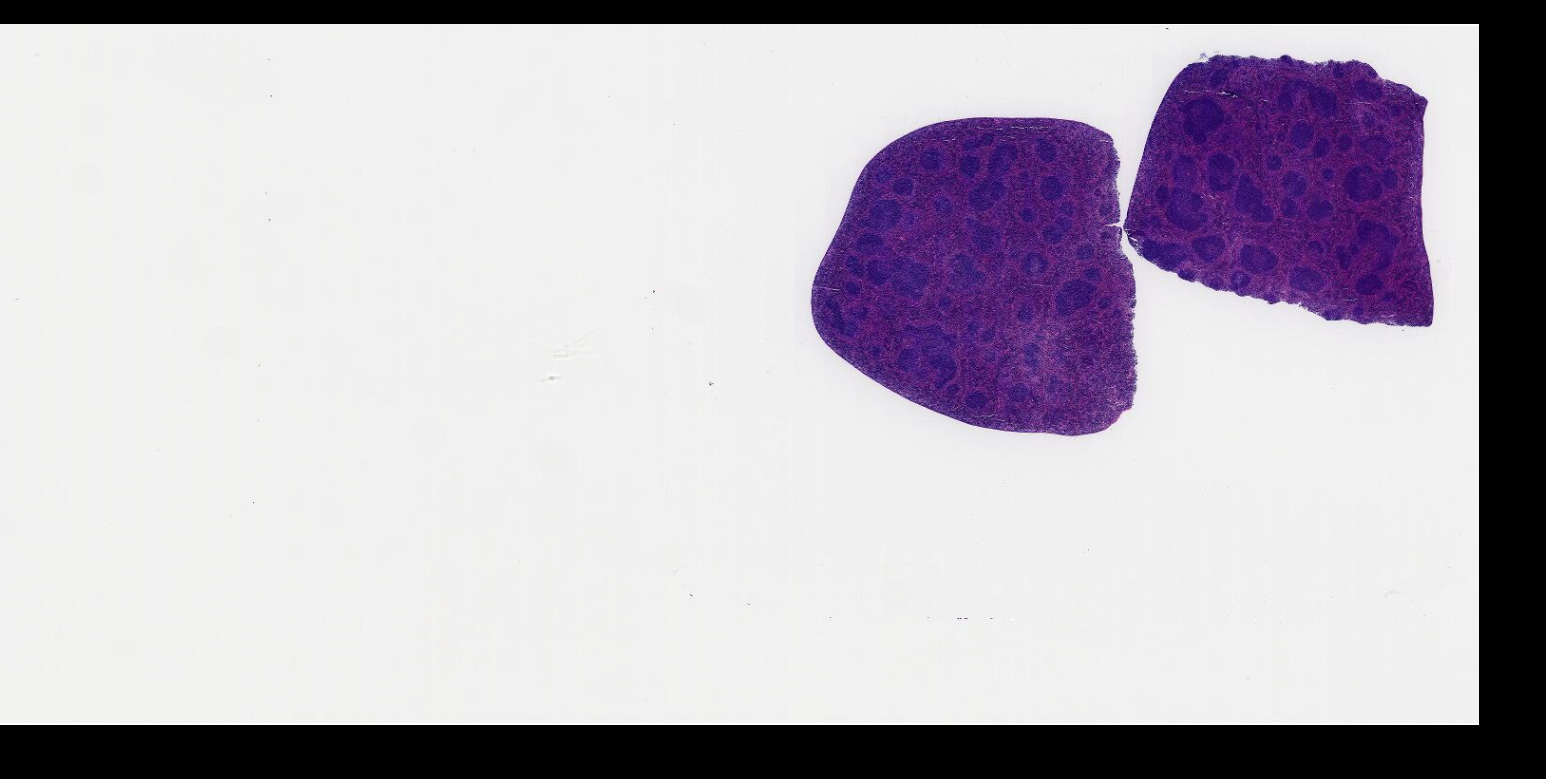

Supplement: Supplementary file 9 — EV Figure Source Data [file 44318_2024_237_MOESM9_ESM.zip › Figure EV3/Figure EV3C/K730R spleen_0.5X_20240705124647.tif]

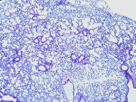

Supplement: Supplementary file 9 — EV Figure Source Data [file 44318_2024_237_MOESM9_ESM.zip › Figure EV3/Figure EV3C/K810R lung.tif]

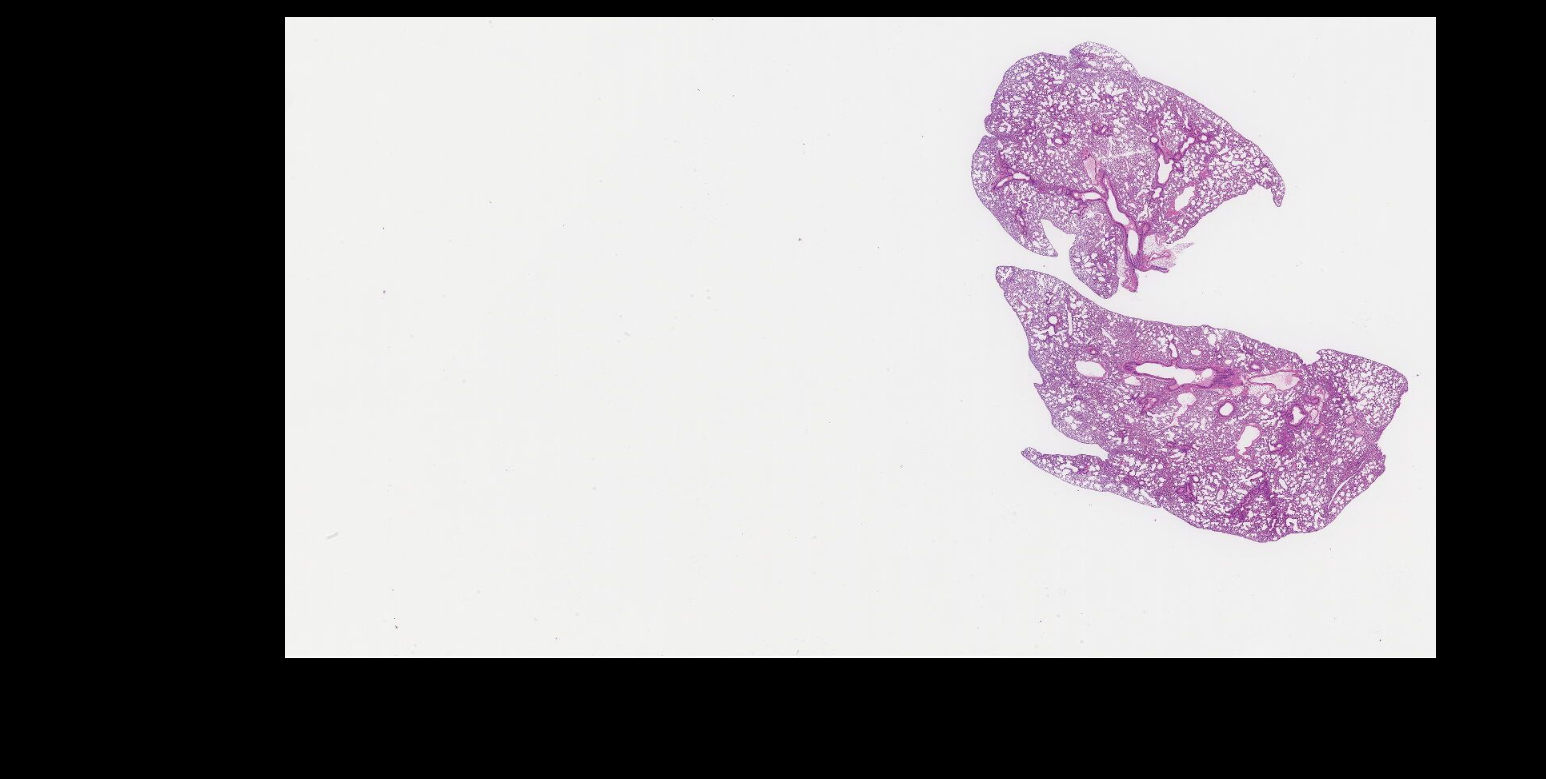

Supplement: Supplementary file 9 — EV Figure Source Data [file 44318_2024_237_MOESM9_ESM.zip › Figure EV3/Figure EV3C/K810R lung_0.50X_20240705011227.tif]

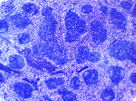

Supplement: Supplementary file 9 — EV Figure Source Data [file 44318_2024_237_MOESM9_ESM.zip › Figure EV3/Figure EV3C/K810R spleen.tif]

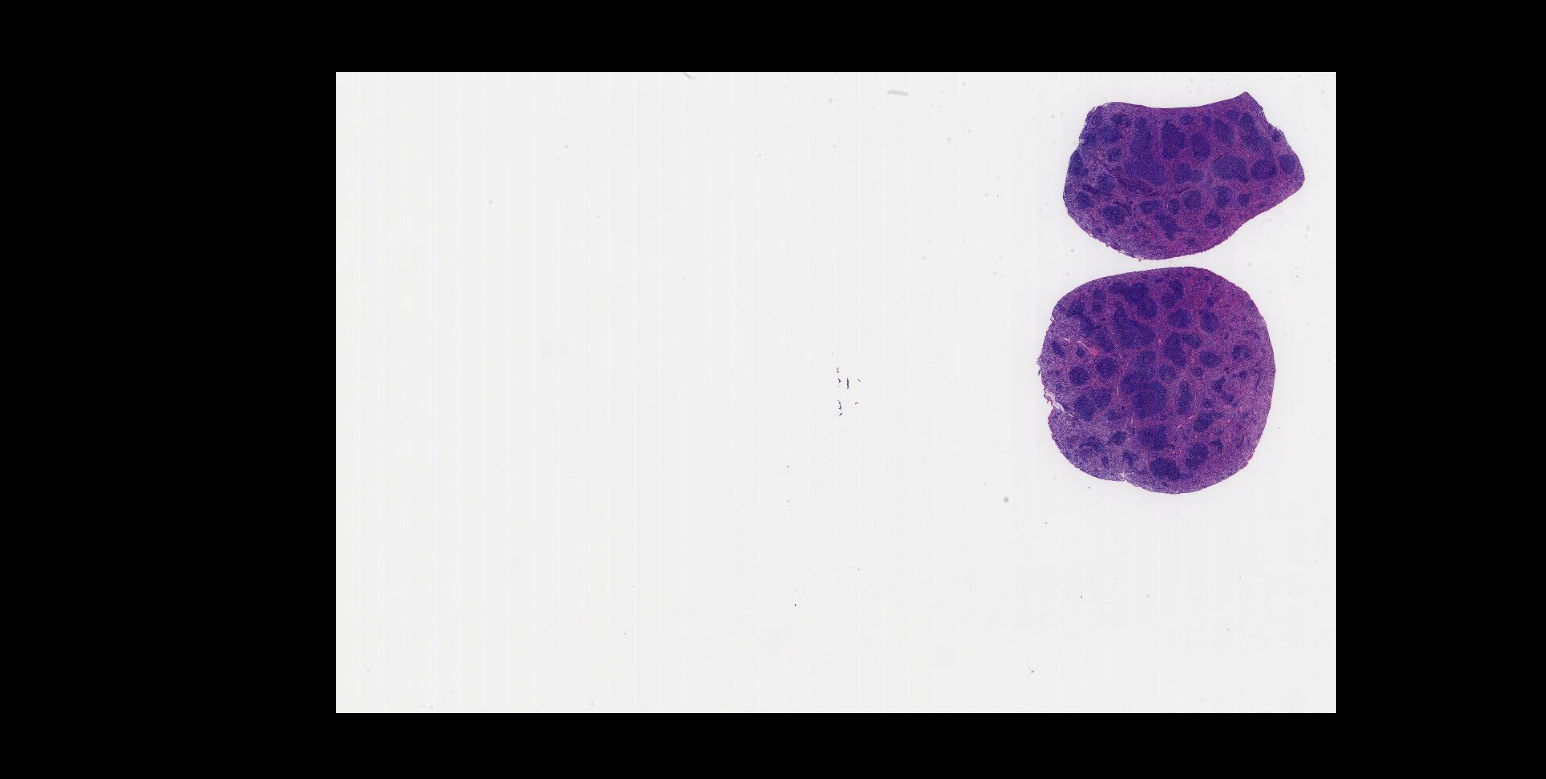

Supplement: Supplementary file 9 — EV Figure Source Data [file 44318_2024_237_MOESM9_ESM.zip › Figure EV3/Figure EV3C/K810R spleen_0.50X_20240705023415.tif]

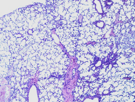

Supplement: Supplementary file 9 — EV Figure Source Data [file 44318_2024_237_MOESM9_ESM.zip › Figure EV3/Figure EV3C/wt lung.tif]

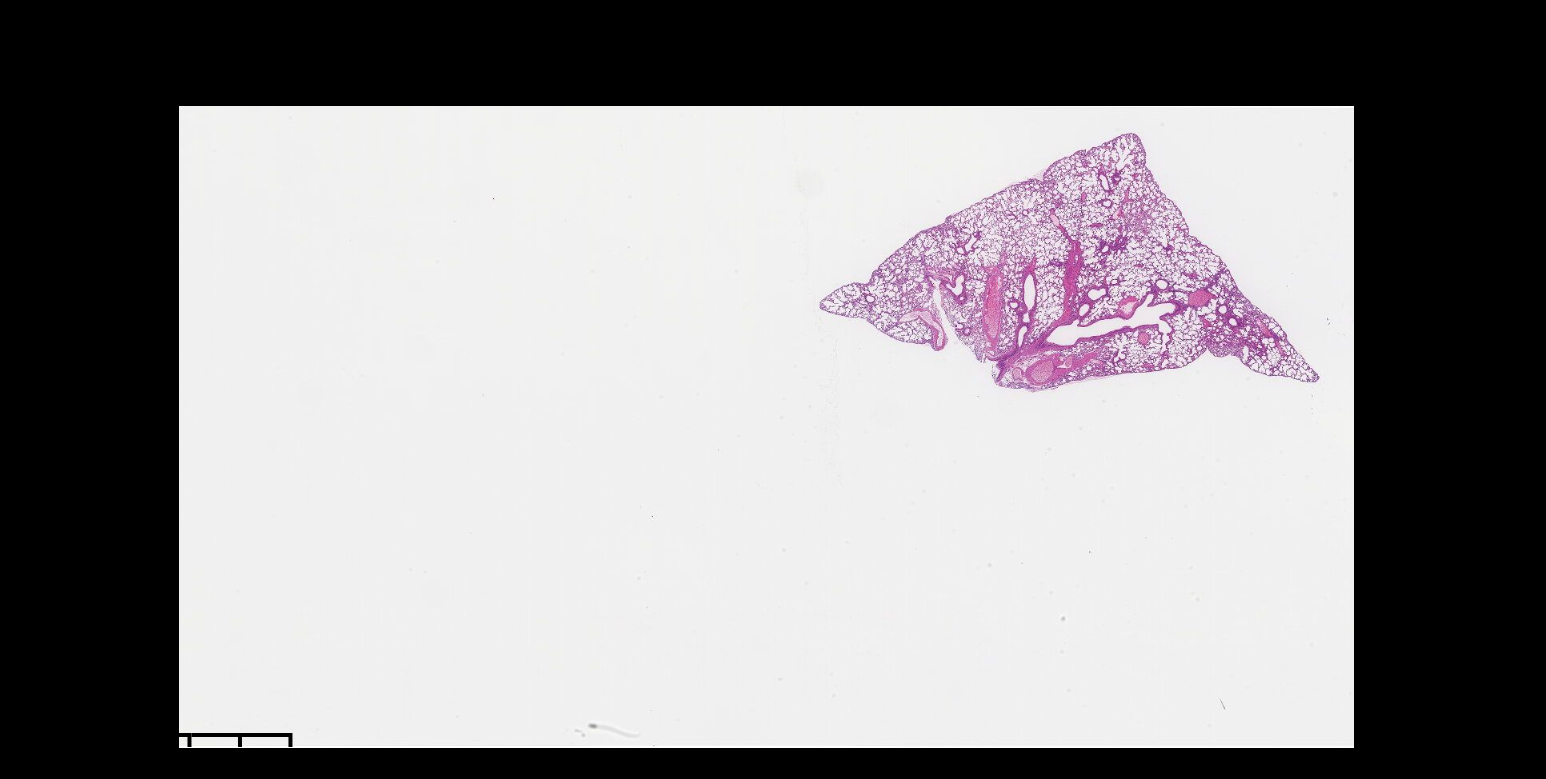

Supplement: Supplementary file 9 — EV Figure Source Data [file 44318_2024_237_MOESM9_ESM.zip › Figure EV3/Figure EV3C/wt lung_0.50X_20240705011640.tif]

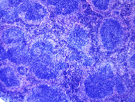

Supplement: Supplementary file 9 — EV Figure Source Data [file 44318_2024_237_MOESM9_ESM.zip › Figure EV3/Figure EV3C/wt spleen.tif]

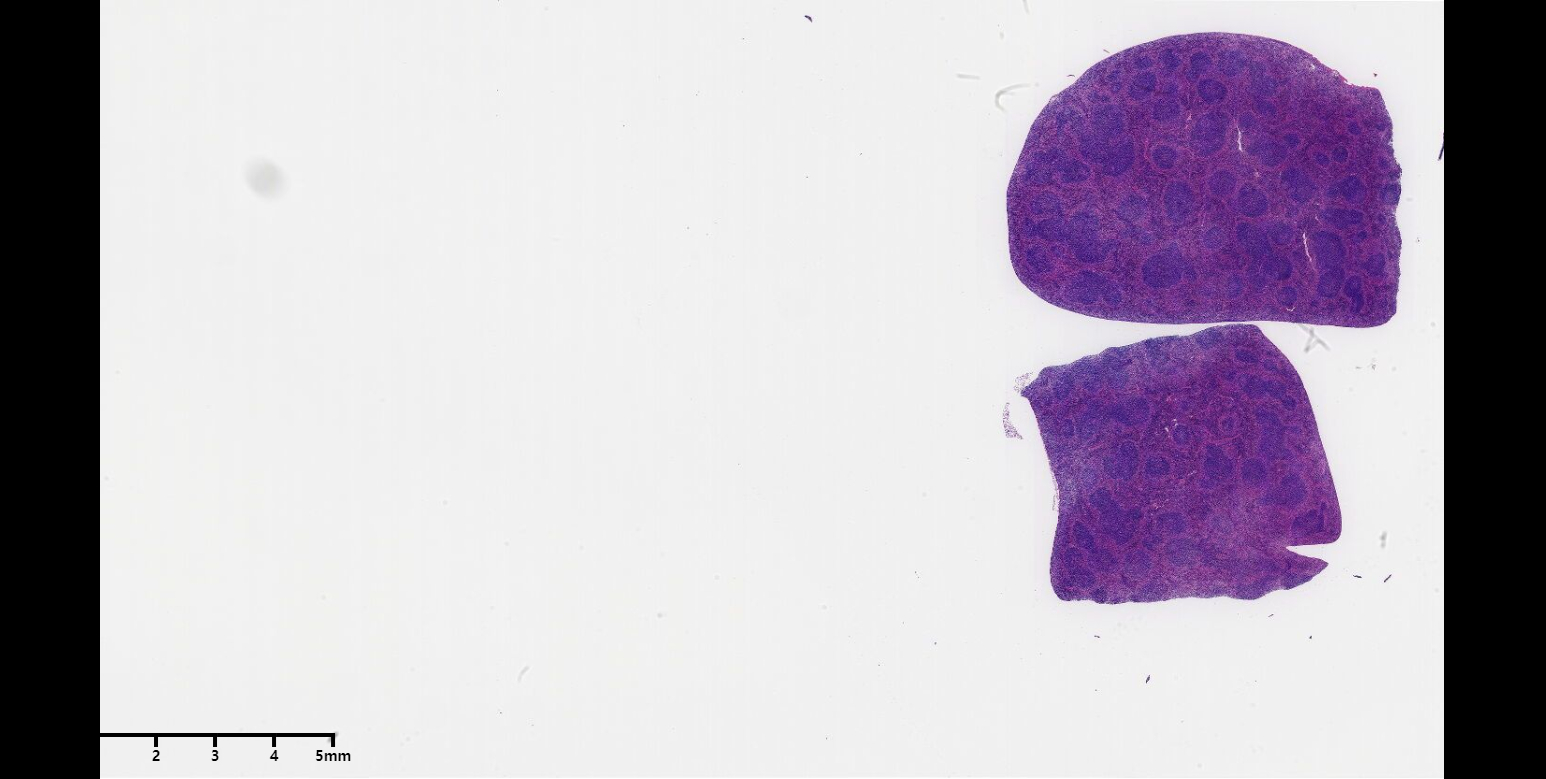

Supplement: Supplementary file 9 — EV Figure Source Data [file 44318_2024_237_MOESM9_ESM.zip › Figure EV3/Figure EV3C/WT_0.5X_20240705124106.tif]

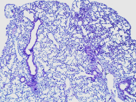

Supplement: Supplementary file 9 — EV Figure Source Data [file 44318_2024_237_MOESM9_ESM.zip › Figure EV3/Figure EV3F/control lung(wt).tif]

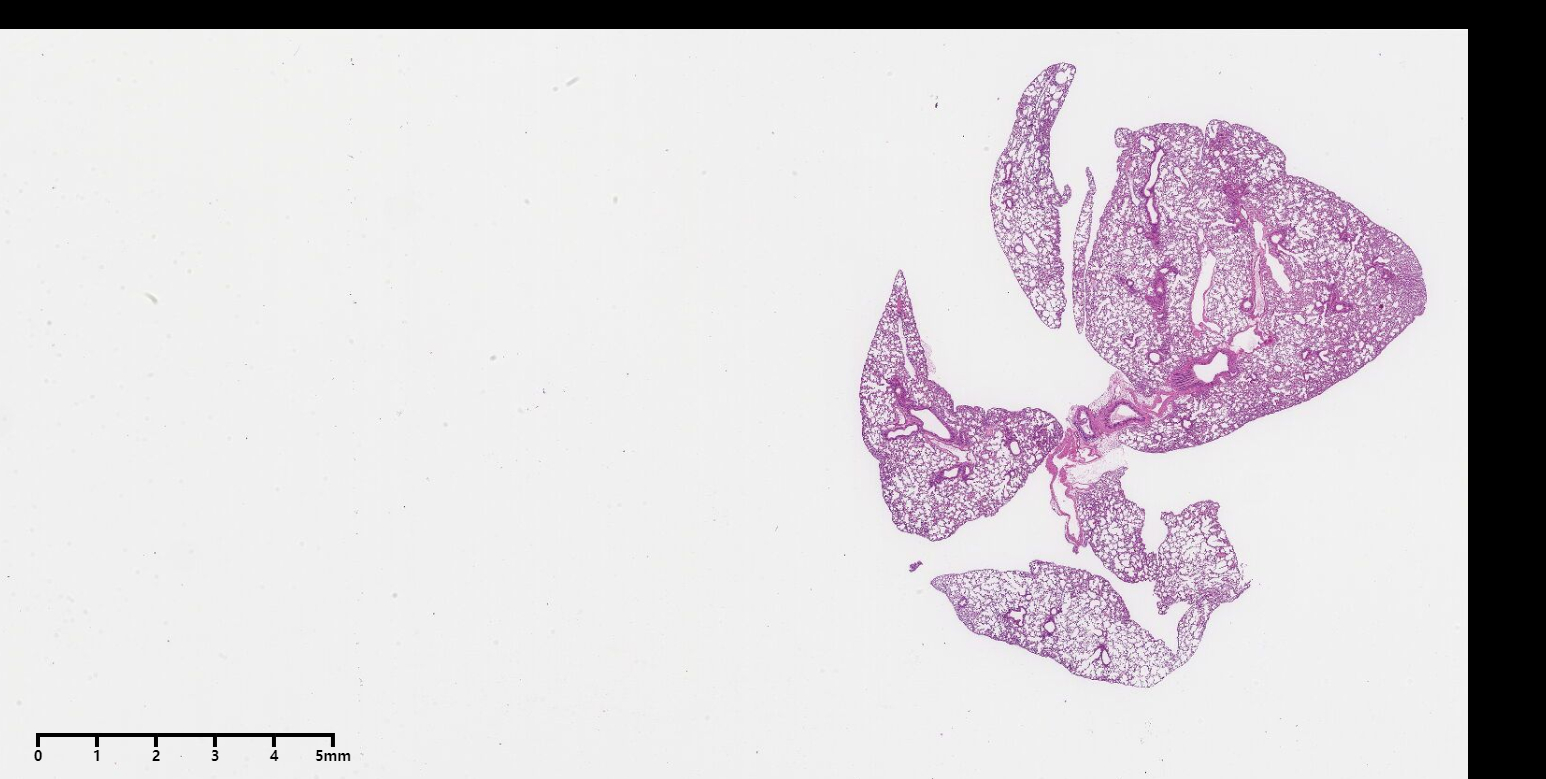

Supplement: Supplementary file 9 — EV Figure Source Data [file 44318_2024_237_MOESM9_ESM.zip › Figure EV3/Figure EV3F/control lungú¿wtú⌐_0.50X_20240705011519.tif]

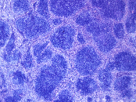

Supplement: Supplementary file 9 — EV Figure Source Data [file 44318_2024_237_MOESM9_ESM.zip › Figure EV3/Figure EV3F/control spleen(wt).tif]

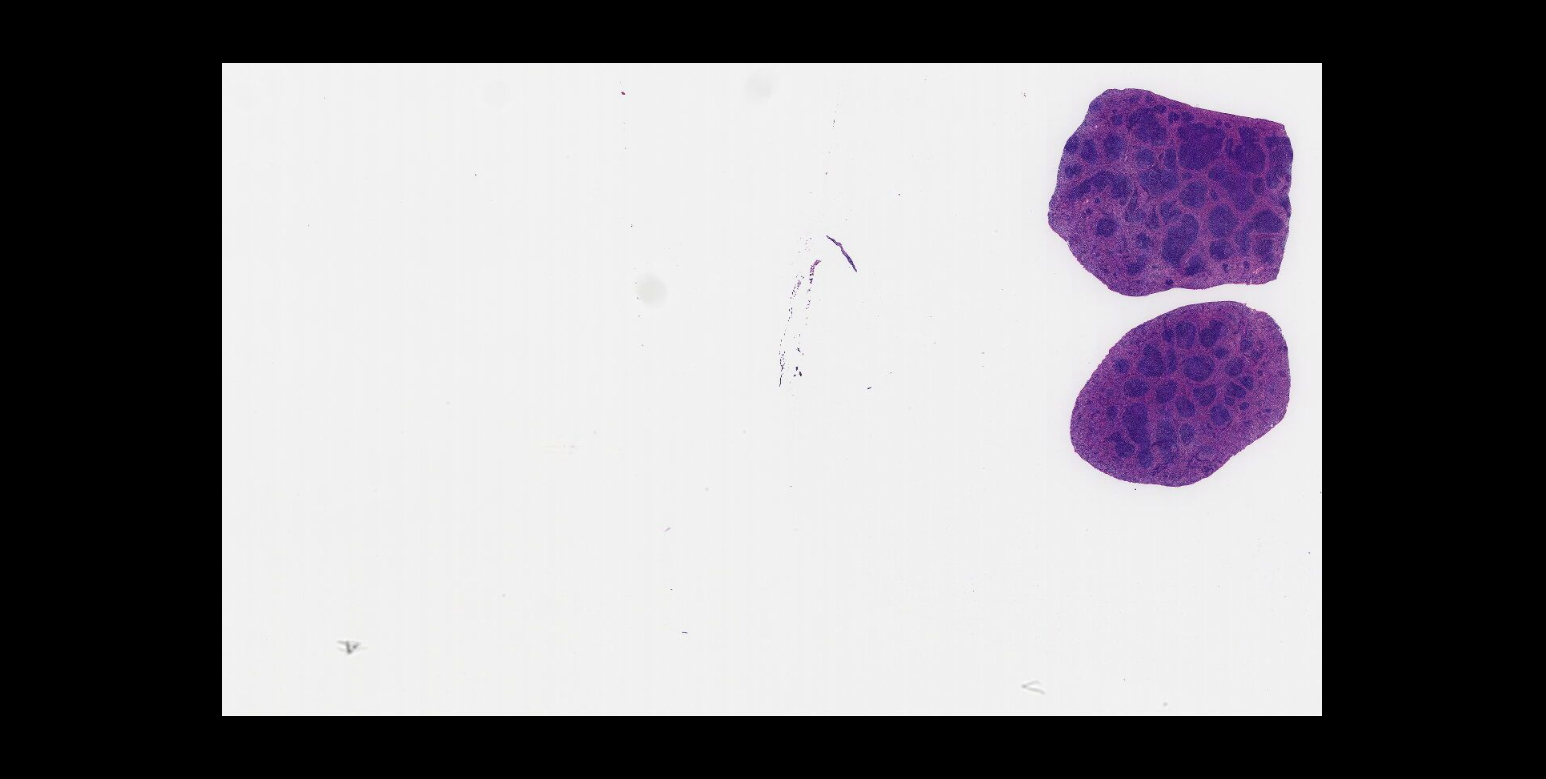

Supplement: Supplementary file 9 — EV Figure Source Data [file 44318_2024_237_MOESM9_ESM.zip › Figure EV3/Figure EV3F/control spleenú¿wtú⌐_0.50X_20240705124315.tif]

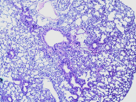

Supplement: Supplementary file 9 — EV Figure Source Data [file 44318_2024_237_MOESM9_ESM.zip › Figure EV3/Figure EV3F/RG2833 lung(wt).tif]

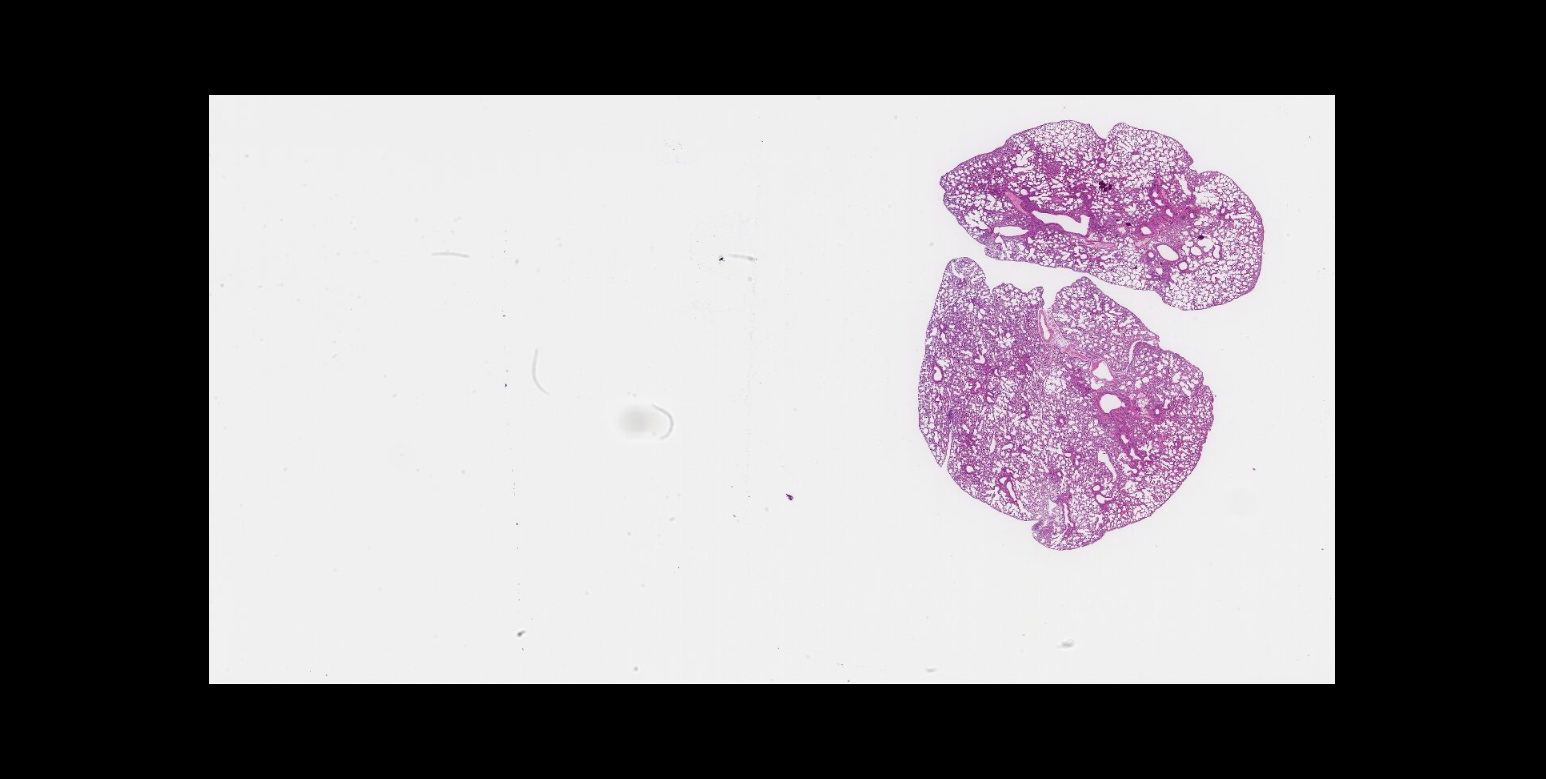

Supplement: Supplementary file 9 — EV Figure Source Data [file 44318_2024_237_MOESM9_ESM.zip › Figure EV3/Figure EV3F/RG2833 lungú¿wtú⌐_0.50X_20240705125201.tif]

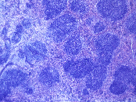

Supplement: Supplementary file 9 — EV Figure Source Data [file 44318_2024_237_MOESM9_ESM.zip › Figure EV3/Figure EV3F/RG2833 spleen(wt).tif]

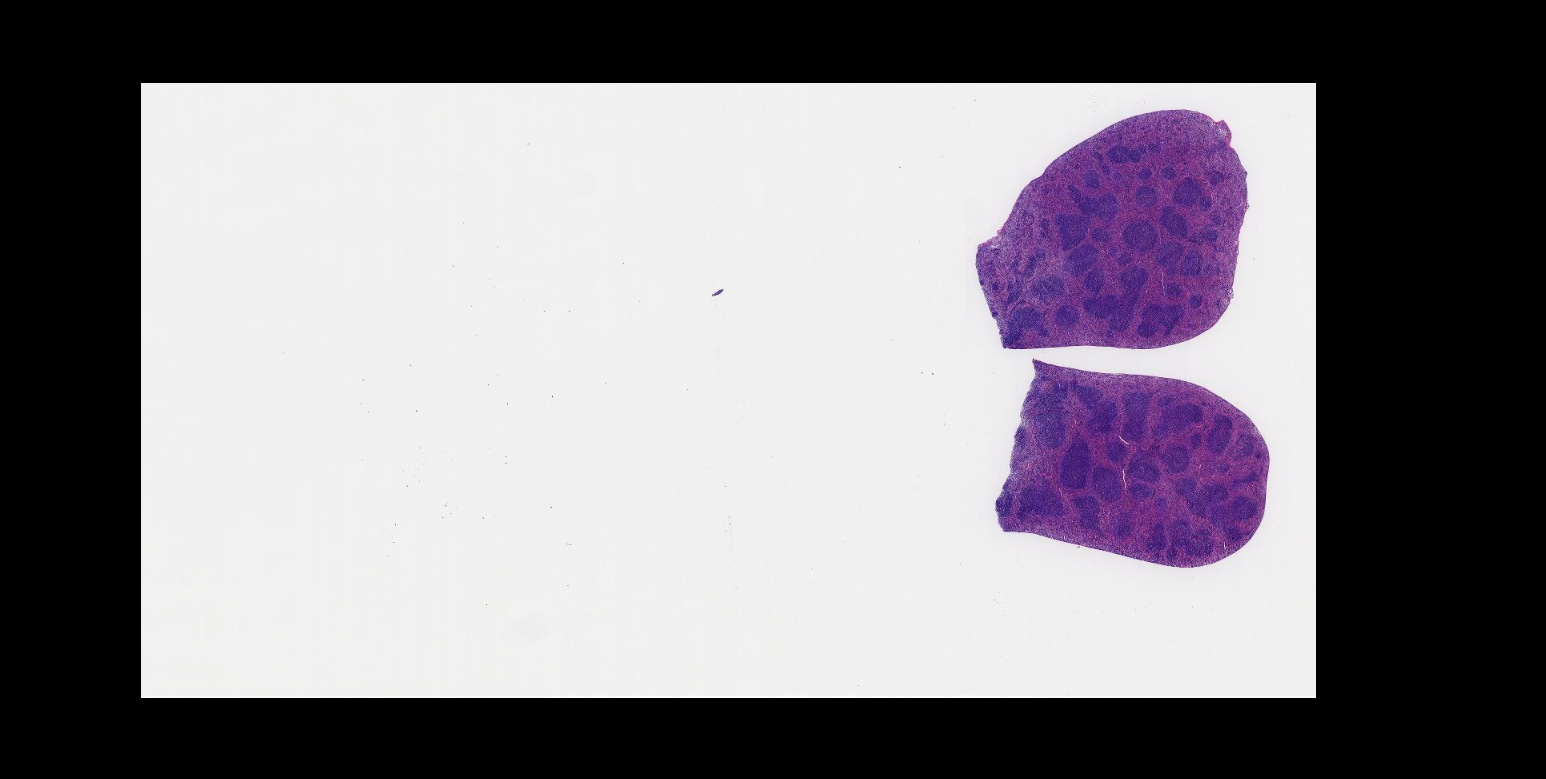

Supplement: Supplementary file 9 — EV Figure Source Data [file 44318_2024_237_MOESM9_ESM.zip › Figure EV3/Figure EV3F/RG2833 spleenú¿wtú⌐_0.50X_20240705124901.tif]

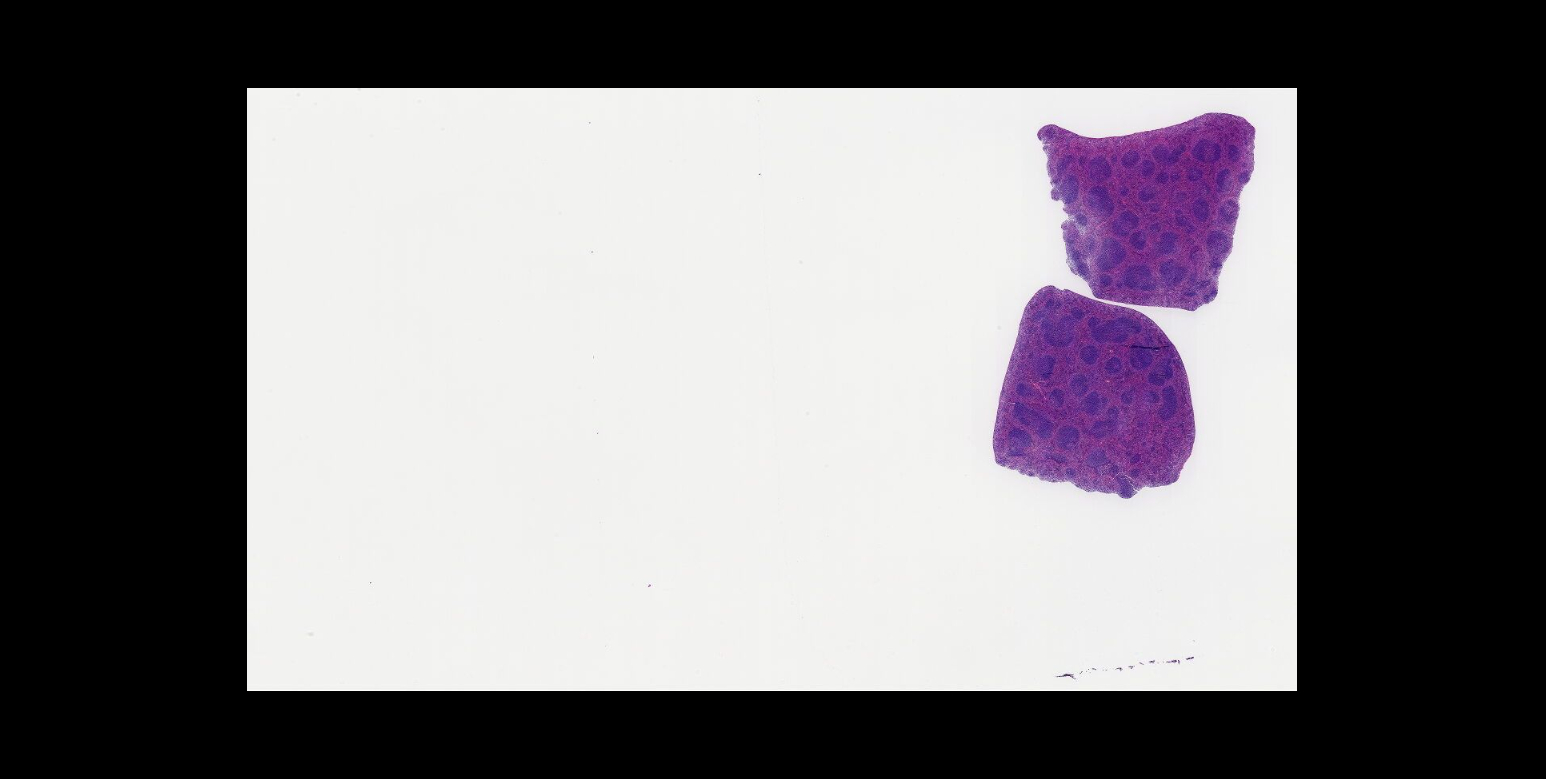

Supplement: Supplementary file 9 — EV Figure Source Data [file 44318_2024_237_MOESM9_ESM.zip › Figure EV3/Figure EV3F/SGC-CBP spleenú¿wtú⌐_0.50X_20240705124422.tif]

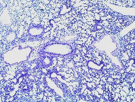

Supplement: Supplementary file 9 — EV Figure Source Data [file 44318_2024_237_MOESM9_ESM.zip › Figure EV3/Figure EV3F/SGC-CBP30 lung(wt).tif]

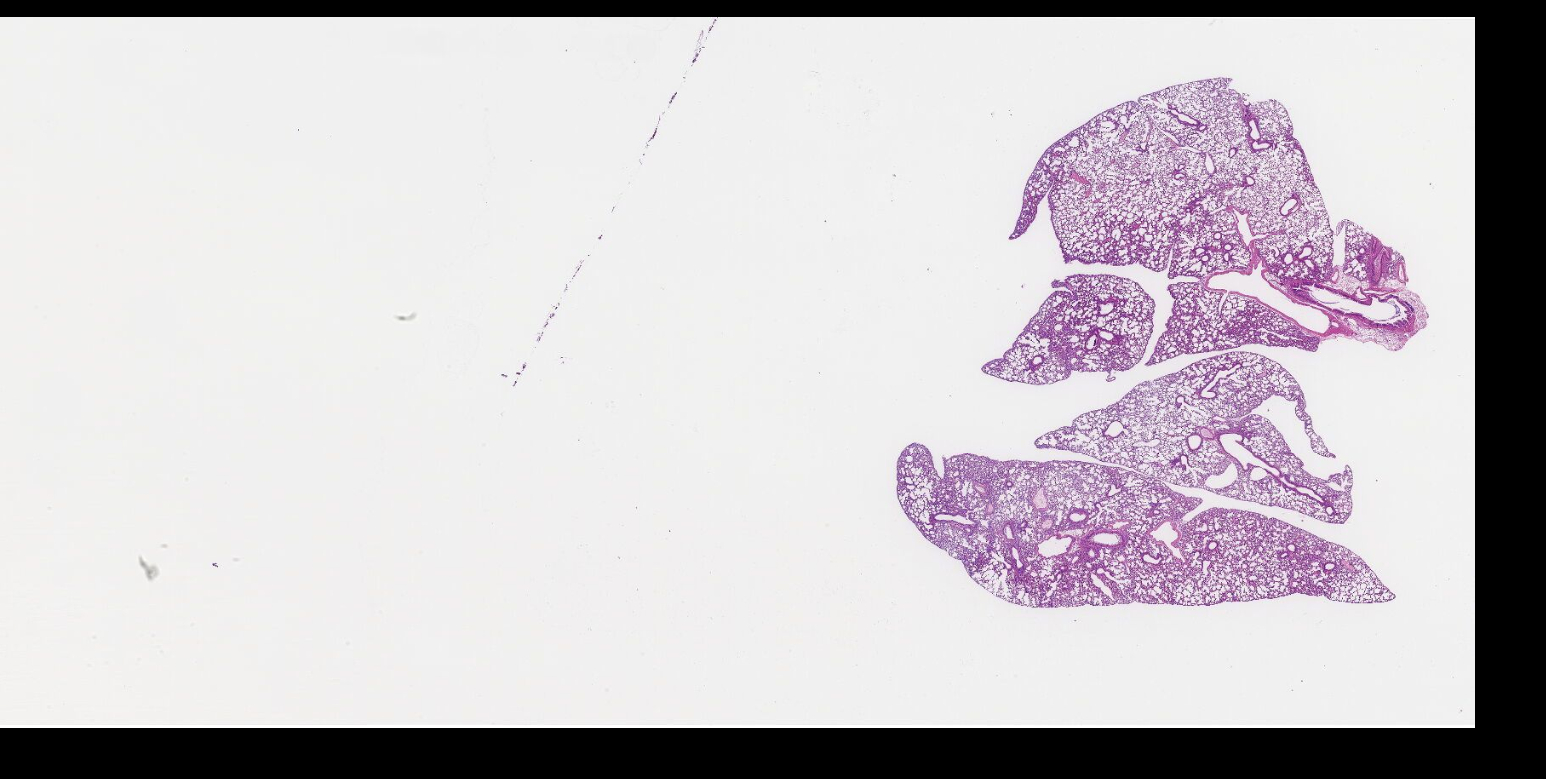

Supplement: Supplementary file 9 — EV Figure Source Data [file 44318_2024_237_MOESM9_ESM.zip › Figure EV3/Figure EV3F/SGC-CBP30 lungú¿wtú⌐_0.5X_20240705125023.tif]

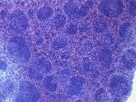

Supplement: Supplementary file 9 — EV Figure Source Data [file 44318_2024_237_MOESM9_ESM.zip › Figure EV3/Figure EV3F/SGC-CBP30 spleen(wt).tif]

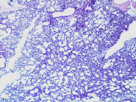

Supplement: Supplementary file 9 — EV Figure Source Data [file 44318_2024_237_MOESM9_ESM.zip › Figure EV3/Figure EV3F/TSA lung(wt).tif]

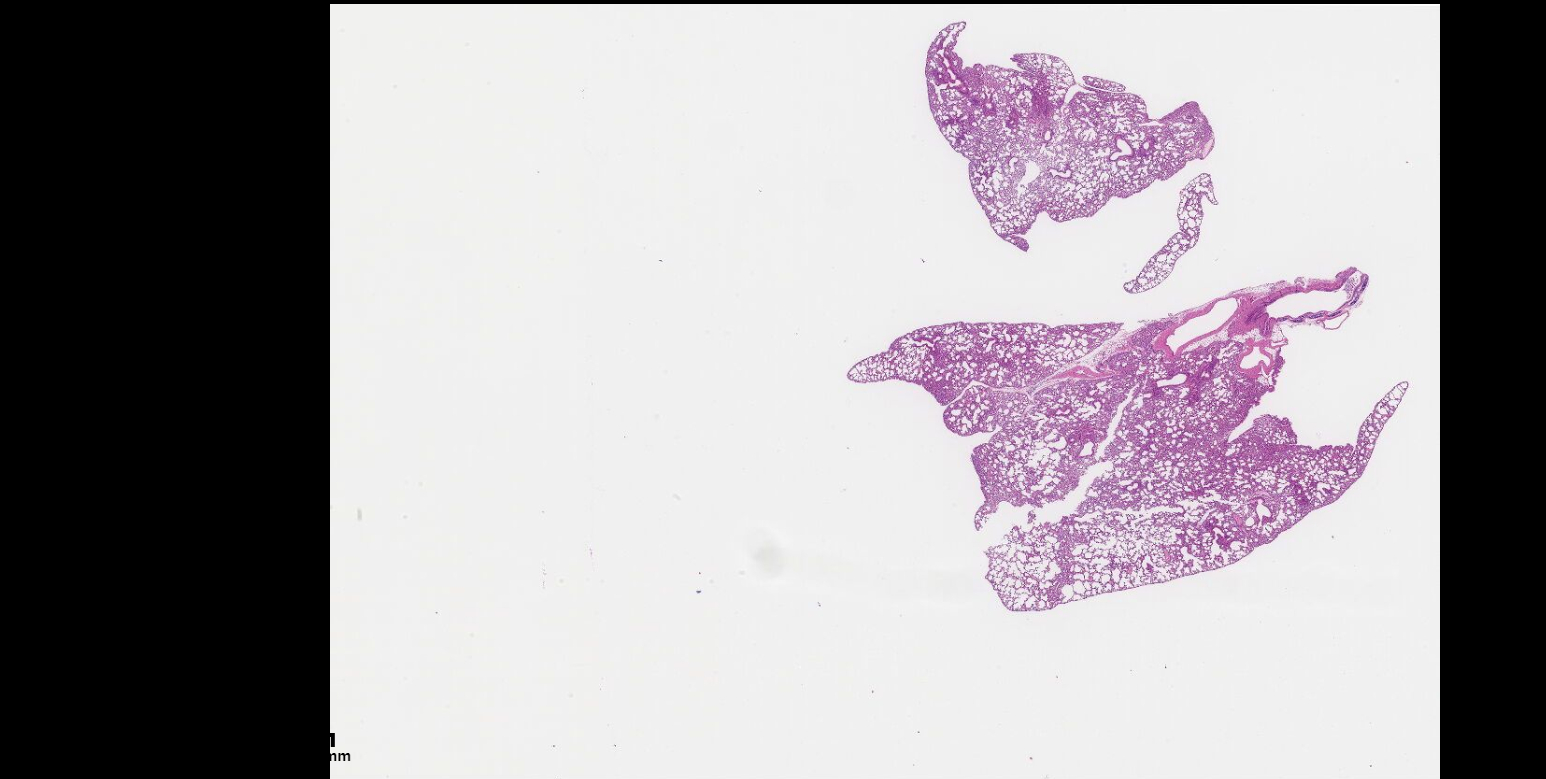

Supplement: Supplementary file 9 — EV Figure Source Data [file 44318_2024_237_MOESM9_ESM.zip › Figure EV3/Figure EV3F/TSA lungú¿wtú⌐_0.50X_20240705011325.tif]

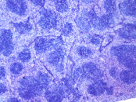

Supplement: Supplementary file 9 — EV Figure Source Data [file 44318_2024_237_MOESM9_ESM.zip › Figure EV3/Figure EV3F/TSA spleen(wt).tif]

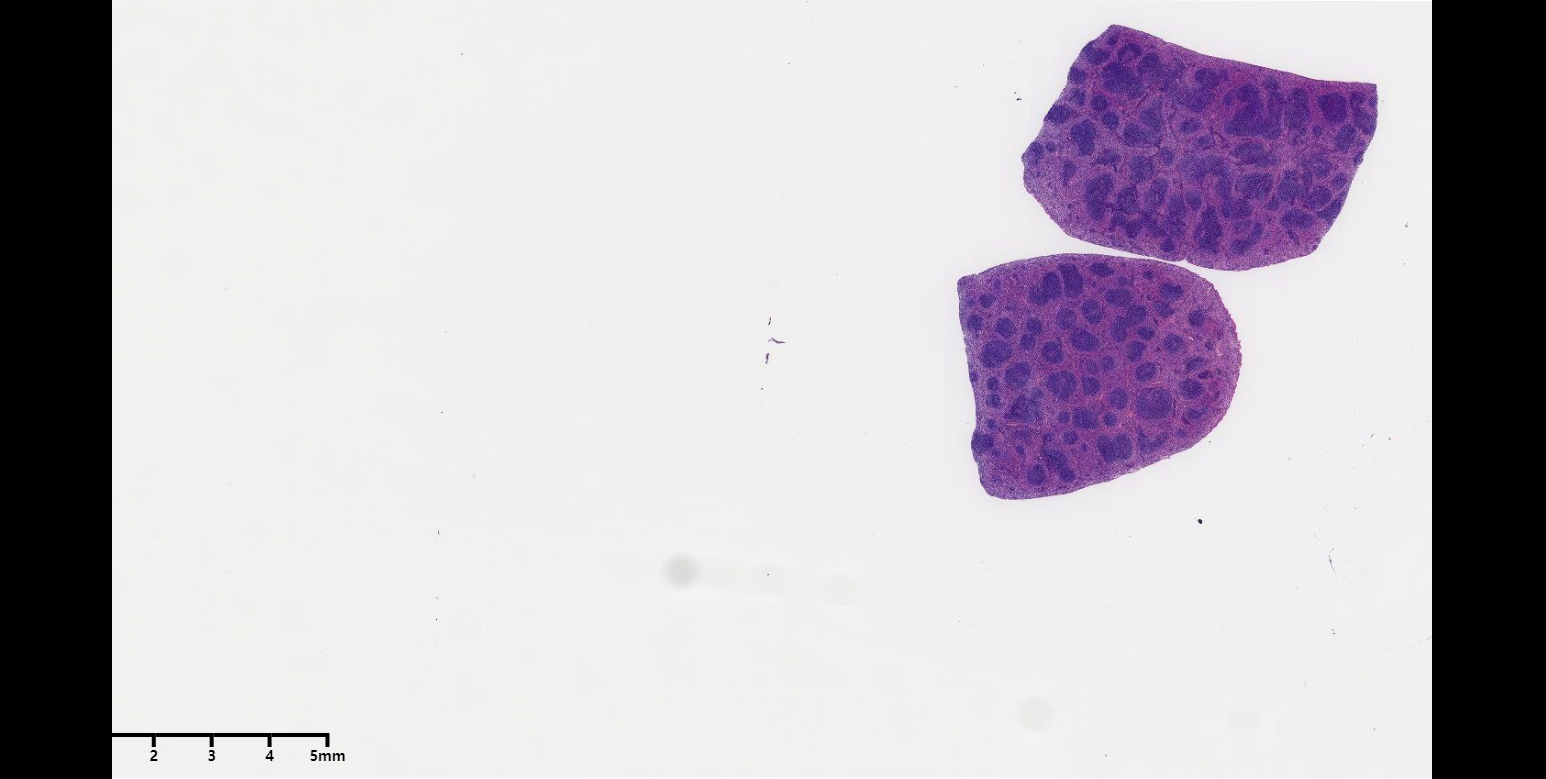

Supplement: Supplementary file 9 — EV Figure Source Data [file 44318_2024_237_MOESM9_ESM.zip › Figure EV3/Figure EV3F/TSA spleenú¿wtú⌐_0.5X_20240705124524.tif]

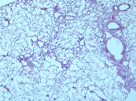

Supplement: Supplementary file 9 — EV Figure Source Data [file 44318_2024_237_MOESM9_ESM.zip › Figure EV3/Figure EV3G/control lungú¿810ú⌐.tif]

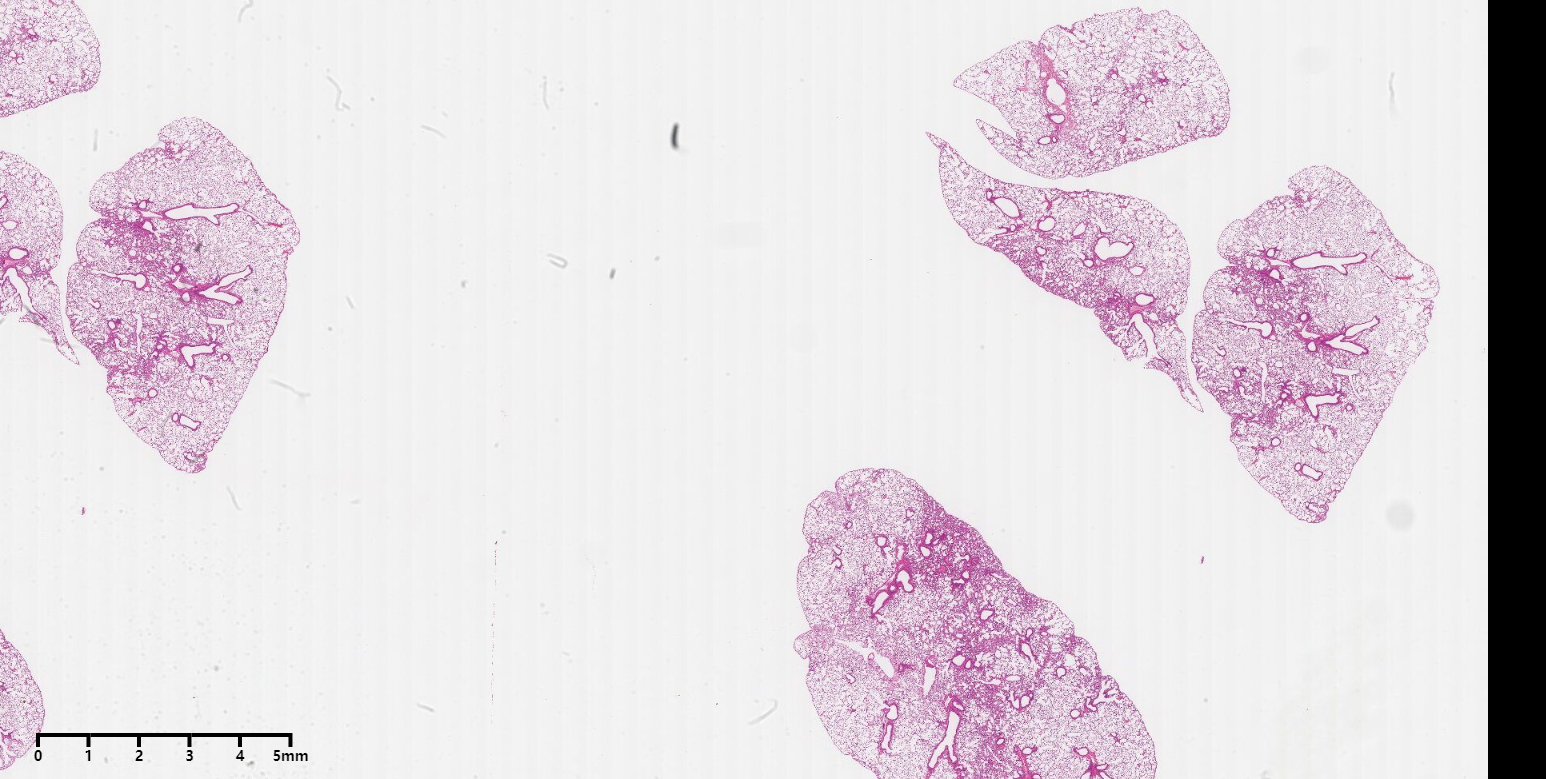

Supplement: Supplementary file 9 — EV Figure Source Data [file 44318_2024_237_MOESM9_ESM.zip › Figure EV3/Figure EV3G/control lungú¿810ú⌐_0.50X_20240705013618.tif]

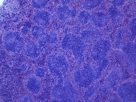

Supplement: Supplementary file 9 — EV Figure Source Data [file 44318_2024_237_MOESM9_ESM.zip › Figure EV3/Figure EV3G/control spleenú¿810ú⌐.tif]

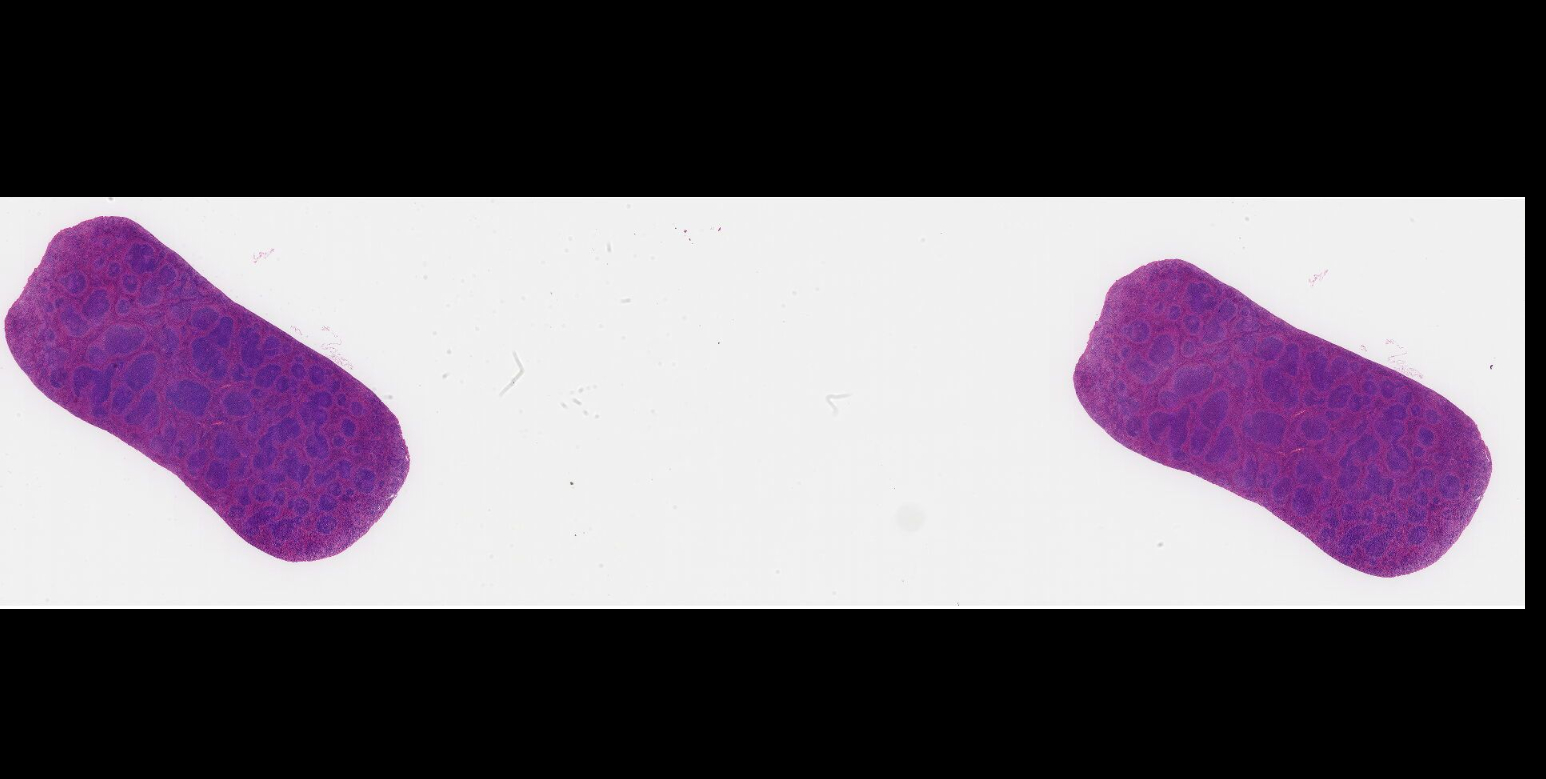

Supplement: Supplementary file 9 — EV Figure Source Data [file 44318_2024_237_MOESM9_ESM.zip › Figure EV3/Figure EV3G/control spleenú¿810ú⌐_0.50X_20240705012844.tif]

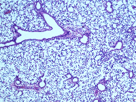

Supplement: Supplementary file 9 — EV Figure Source Data [file 44318_2024_237_MOESM9_ESM.zip › Figure EV3/Figure EV3G/RG2833 lung (810).tif]

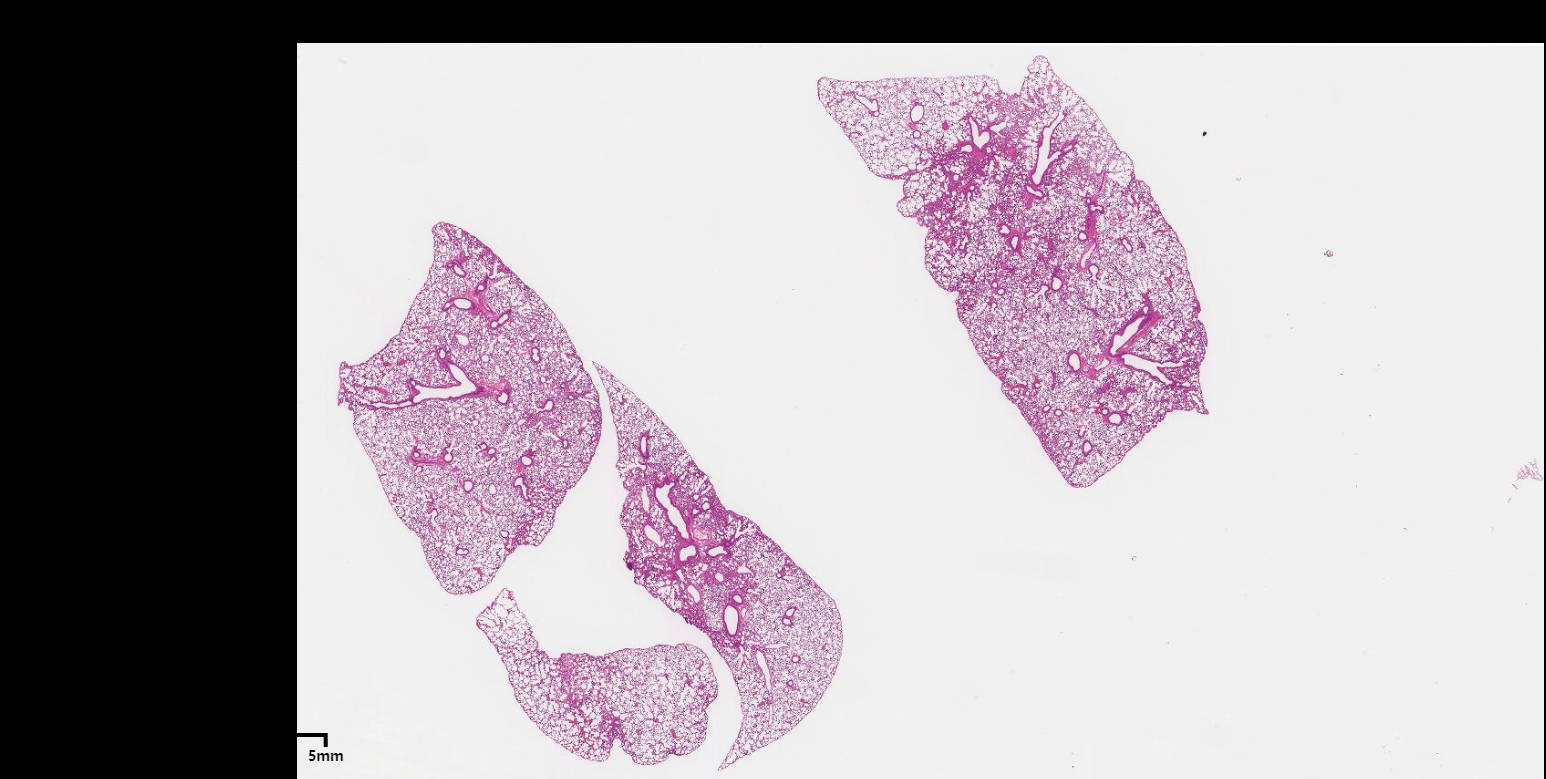

Supplement: Supplementary file 9 — EV Figure Source Data [file 44318_2024_237_MOESM9_ESM.zip › Figure EV3/Figure EV3G/RG2833 lung(810)0.50X_20240705015906.tif]

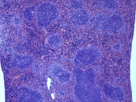

Supplement: Supplementary file 9 — EV Figure Source Data [file 44318_2024_237_MOESM9_ESM.zip › Figure EV3/Figure EV3G/RG2833 spleenú¿810ú⌐.tif]

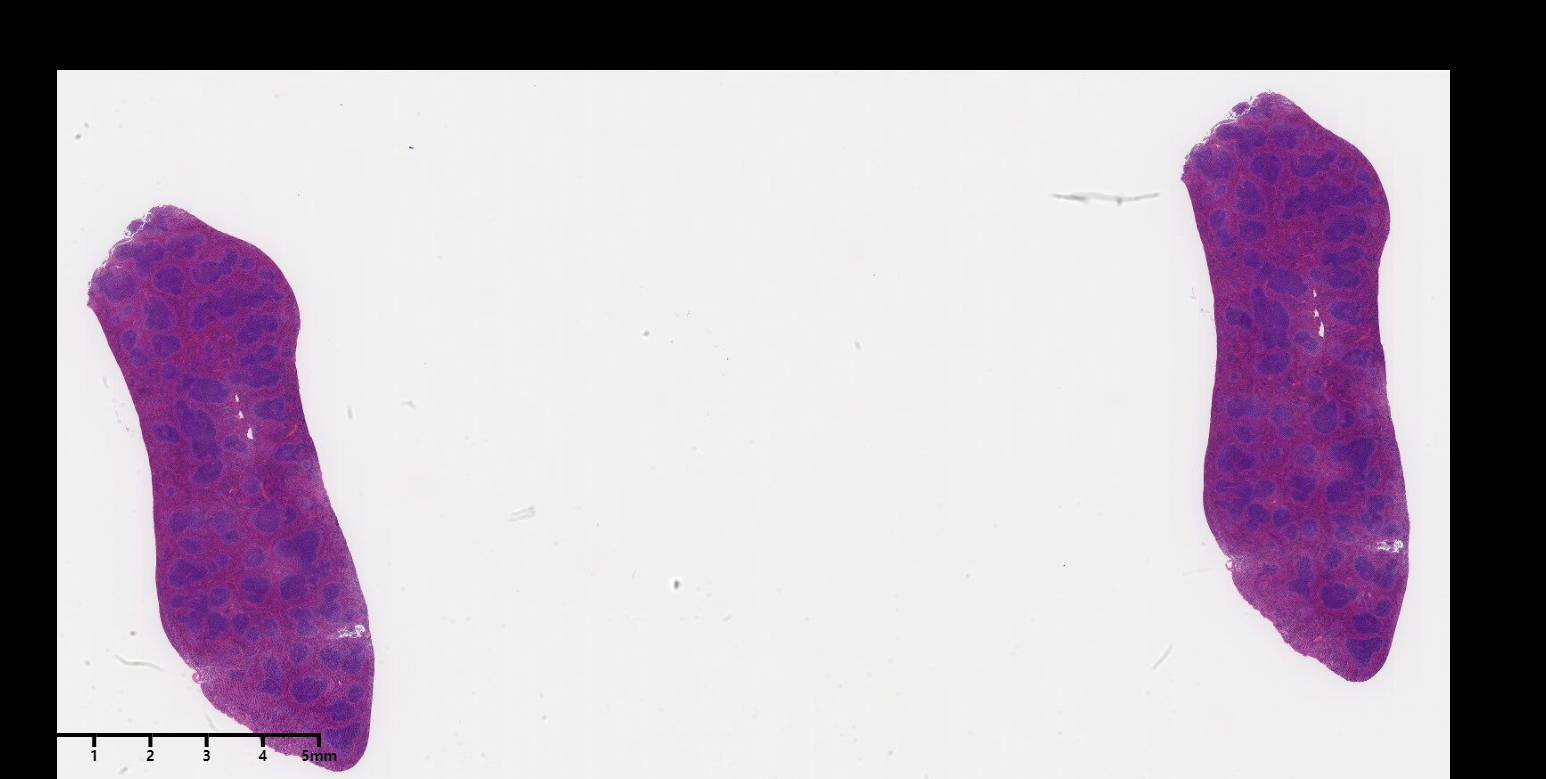

Supplement: Supplementary file 9 — EV Figure Source Data [file 44318_2024_237_MOESM9_ESM.zip › Figure EV3/Figure EV3G/RG2833 spleenú¿810ú⌐_0.50X_20240705032406.tif]

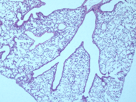

Supplement: Supplementary file 9 — EV Figure Source Data [file 44318_2024_237_MOESM9_ESM.zip › Figure EV3/Figure EV3G/SGC-CBP-30 lungú¿810ú⌐.tif]

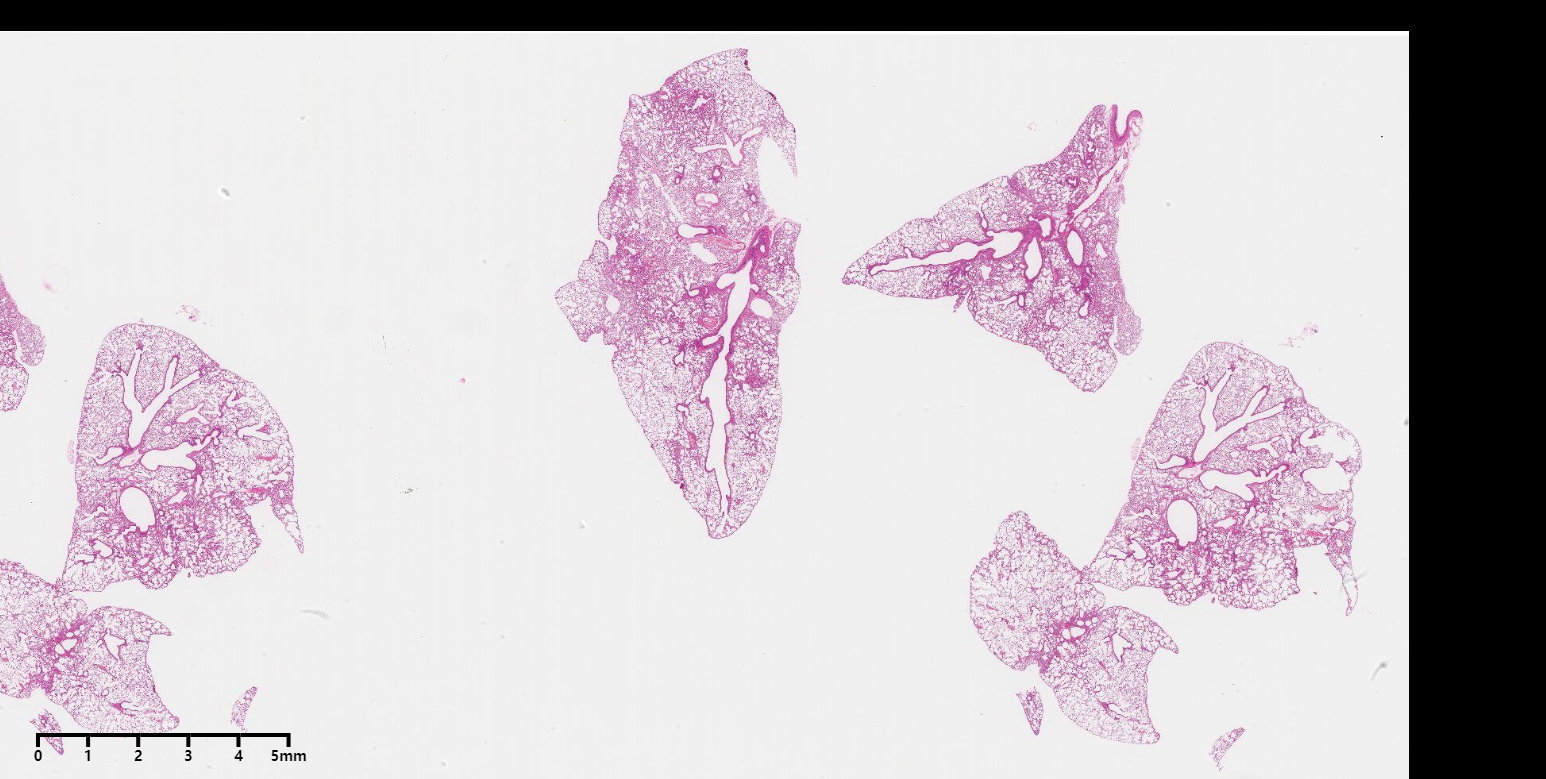

Supplement: Supplementary file 9 — EV Figure Source Data [file 44318_2024_237_MOESM9_ESM.zip › Figure EV3/Figure EV3G/SGC-CBP30 lungú¿810ú⌐_0.50X_20240705025250.tif]

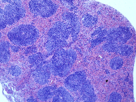

Supplement: Supplementary file 9 — EV Figure Source Data [file 44318_2024_237_MOESM9_ESM.zip › Figure EV3/Figure EV3G/SGC-CBP30 spleenú¿810ú⌐.tif]

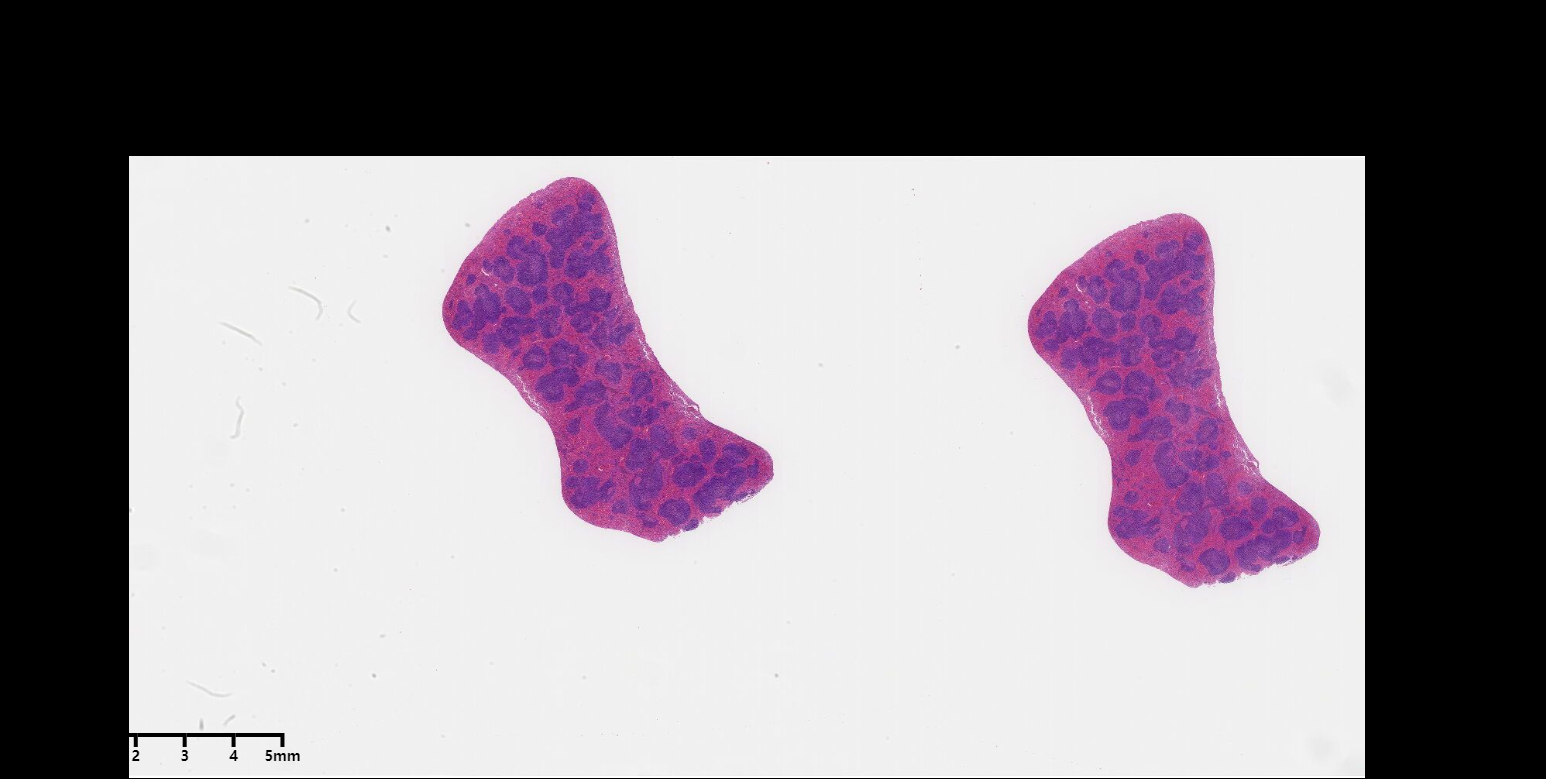

Supplement: Supplementary file 9 — EV Figure Source Data [file 44318_2024_237_MOESM9_ESM.zip › Figure EV3/Figure EV3G/SGC-CBP30 spleenú¿810ú⌐_0.50X_20240705030223.tif]

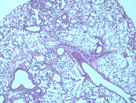

Supplement: Supplementary file 9 — EV Figure Source Data [file 44318_2024_237_MOESM9_ESM.zip › Figure EV3/Figure EV3G/TSA lungú¿810ú⌐.tif]

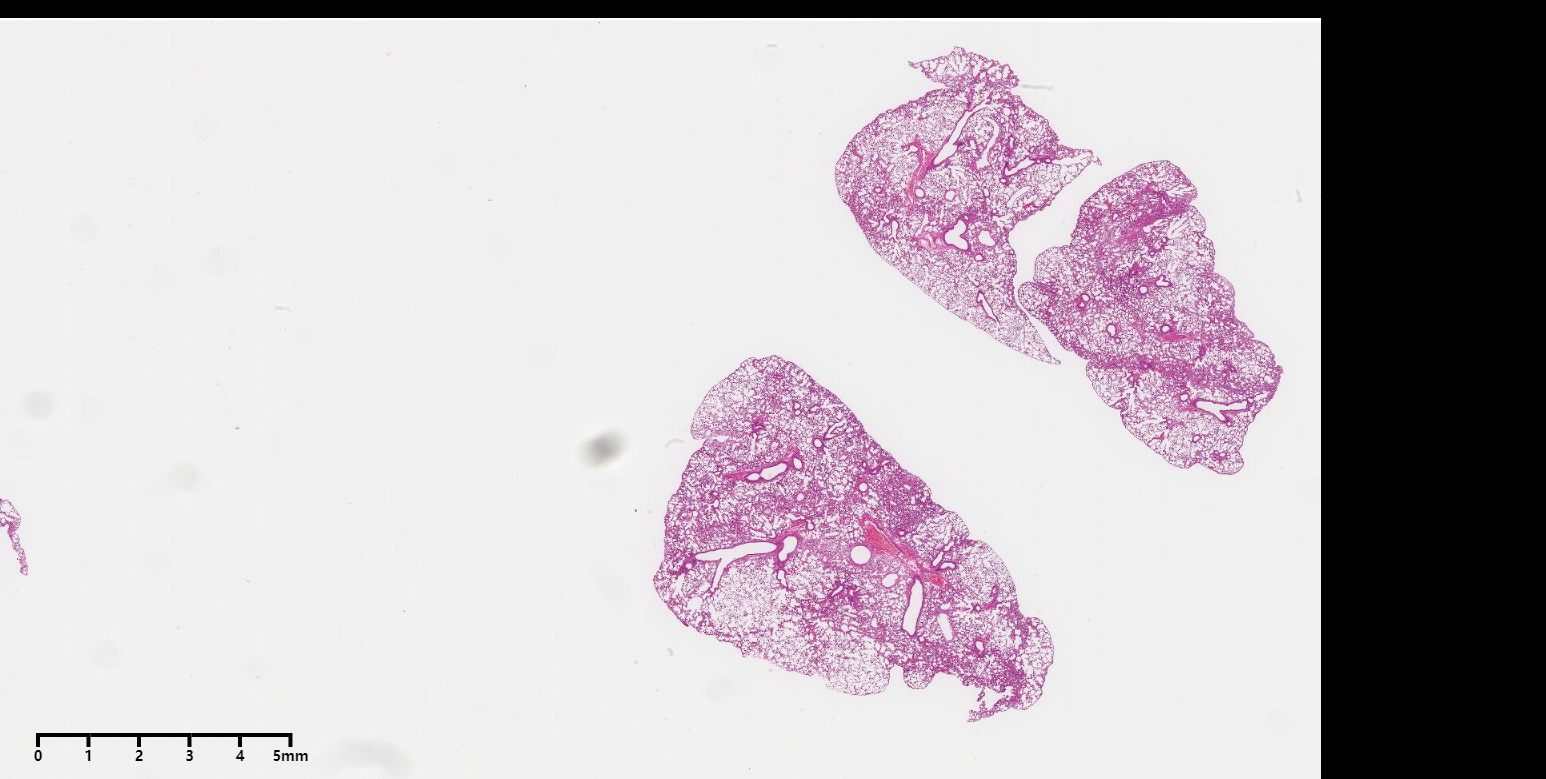

Supplement: Supplementary file 9 — EV Figure Source Data [file 44318_2024_237_MOESM9_ESM.zip › Figure EV3/Figure EV3G/TSA lungú¿810ú⌐_0.50X_20240705020043.tif]

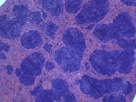

Supplement: Supplementary file 9 — EV Figure Source Data [file 44318_2024_237_MOESM9_ESM.zip › Figure EV3/Figure EV3G/TSA spleenú¿810ú⌐.tif]

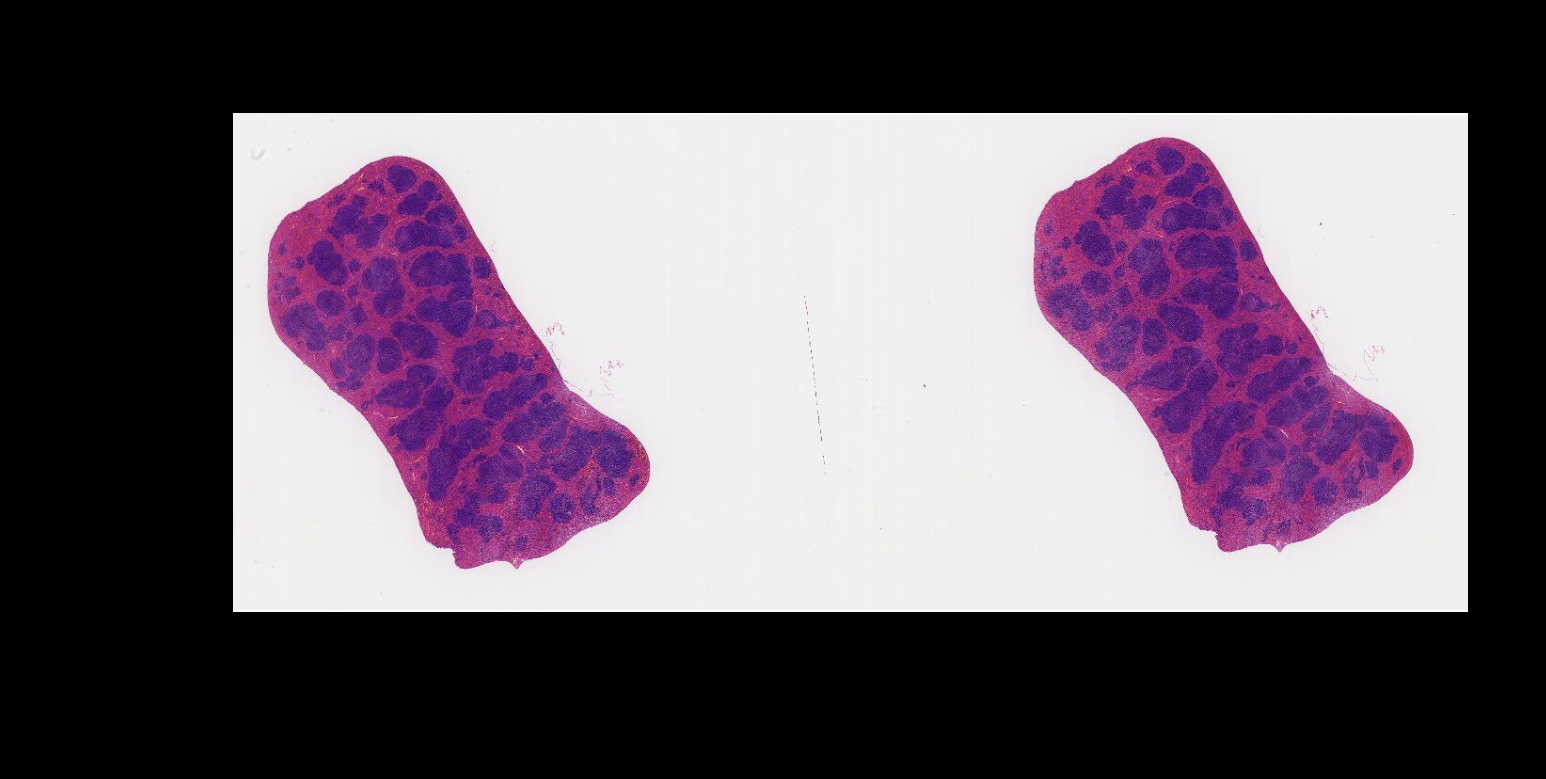

Supplement: Supplementary file 9 — EV Figure Source Data [file 44318_2024_237_MOESM9_ESM.zip › Figure EV3/Figure EV3G/TSA spleenú¿810ú⌐_0.50X_20240705013127.tif]

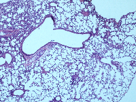

Supplement: Supplementary file 9 — EV Figure Source Data [file 44318_2024_237_MOESM9_ESM.zip › Figure EV3/Figure EV3I/LPS lungú¿810 lpsú⌐.tif]

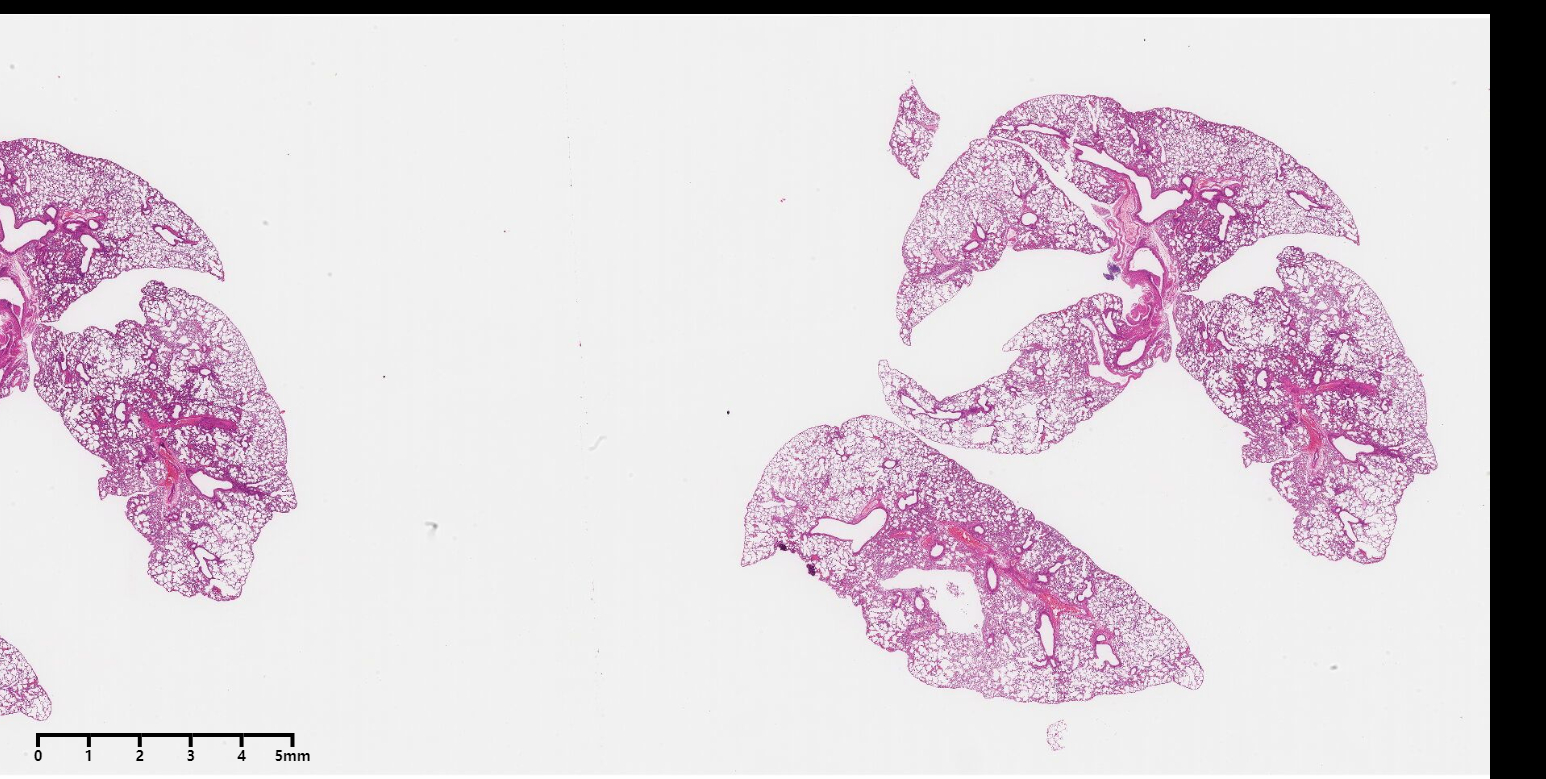

Supplement: Supplementary file 9 — EV Figure Source Data [file 44318_2024_237_MOESM9_ESM.zip › Figure EV3/Figure EV3I/LPS lungú¿810 lpsú⌐_0.50X_20240705022015.tif]

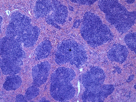

Supplement: Supplementary file 9 — EV Figure Source Data [file 44318_2024_237_MOESM9_ESM.zip › Figure EV3/Figure EV3I/LPS spleenú¿810 lpsú⌐.tif]

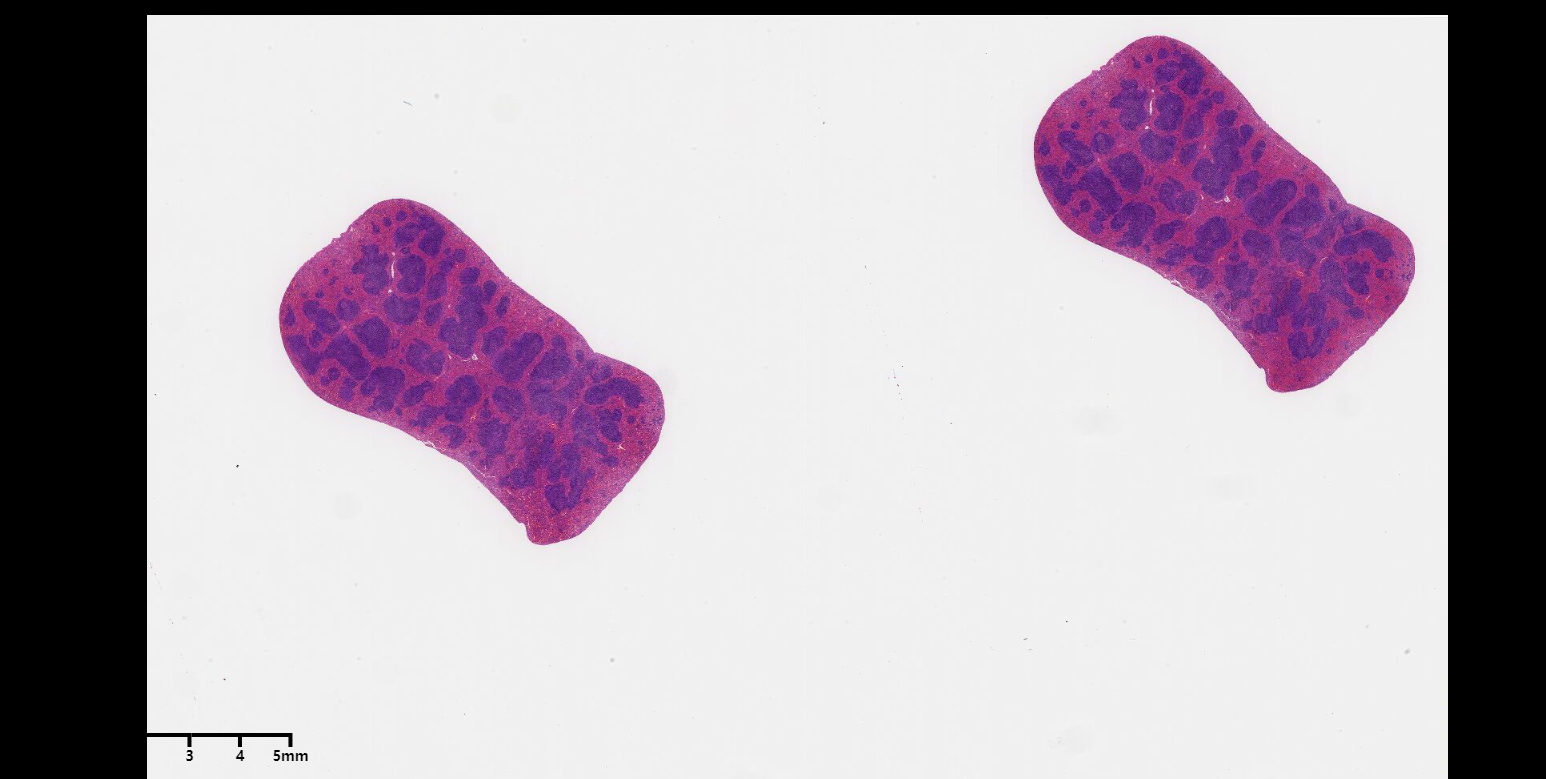

Supplement: Supplementary file 9 — EV Figure Source Data [file 44318_2024_237_MOESM9_ESM.zip › Figure EV3/Figure EV3I/LPS spleenú¿810 lpsú⌐_0.50X_20240705021356.tif]

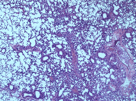

Supplement: Supplementary file 9 — EV Figure Source Data [file 44318_2024_237_MOESM9_ESM.zip › Figure EV3/Figure EV3I/RG2833 lungú¿810 lpsú⌐.tif]

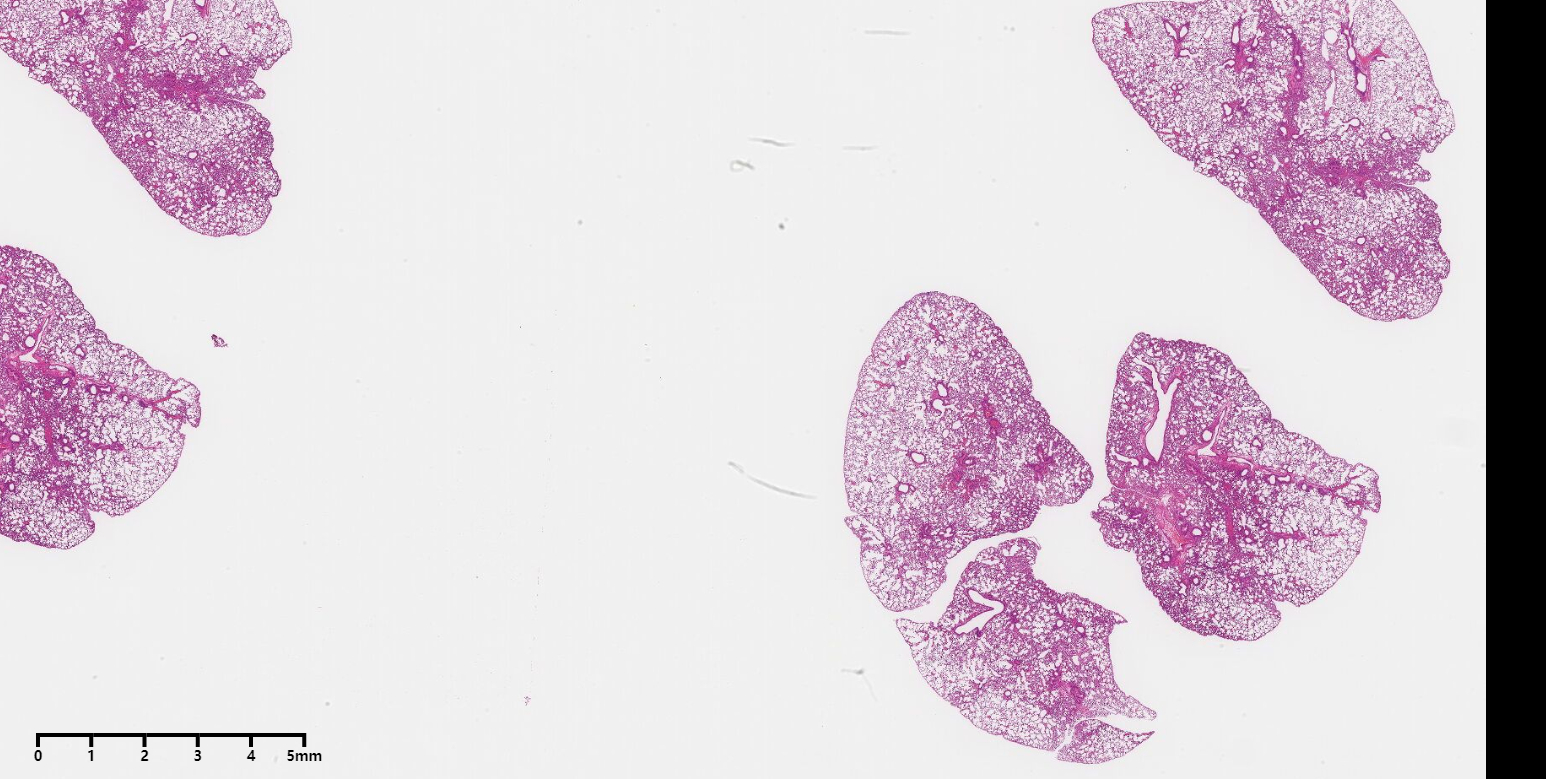

Supplement: Supplementary file 9 — EV Figure Source Data [file 44318_2024_237_MOESM9_ESM.zip › Figure EV3/Figure EV3I/RG2833 lung(810 lps)_0.50X_20240705021842.tif]

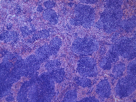

Supplement: Supplementary file 9 — EV Figure Source Data [file 44318_2024_237_MOESM9_ESM.zip › Figure EV3/Figure EV3I/RG2833 spleenú¿810 lpsú⌐.tif]

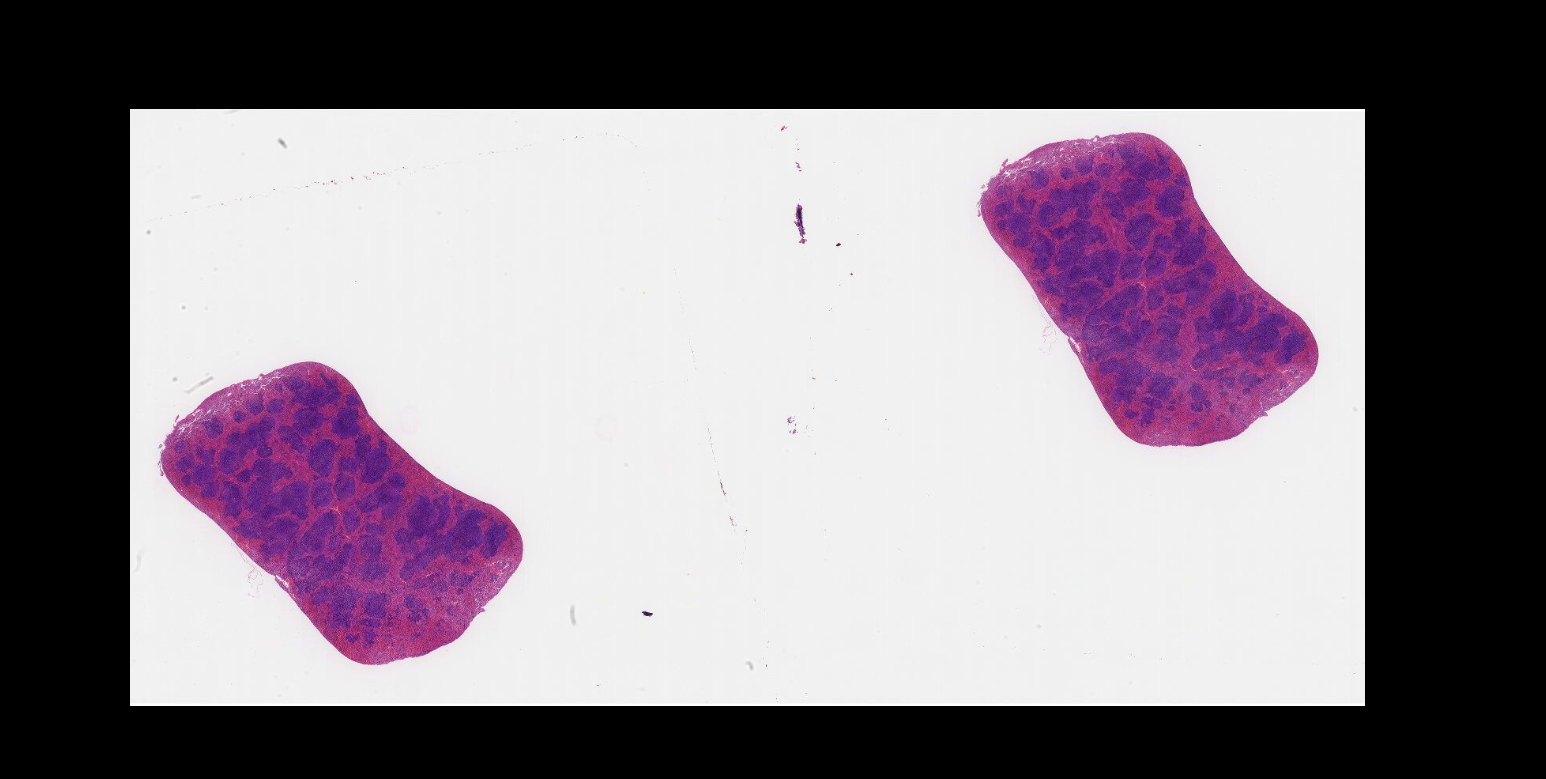

Supplement: Supplementary file 9 — EV Figure Source Data [file 44318_2024_237_MOESM9_ESM.zip › Figure EV3/Figure EV3I/RG2833 spleen(810lps)_0.50X_20240705020744.tif]

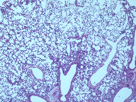

Supplement: Supplementary file 9 — EV Figure Source Data [file 44318_2024_237_MOESM9_ESM.zip › Figure EV3/Figure EV3I/SGC-CBP30 lungú¿810 lpsú⌐.tif]

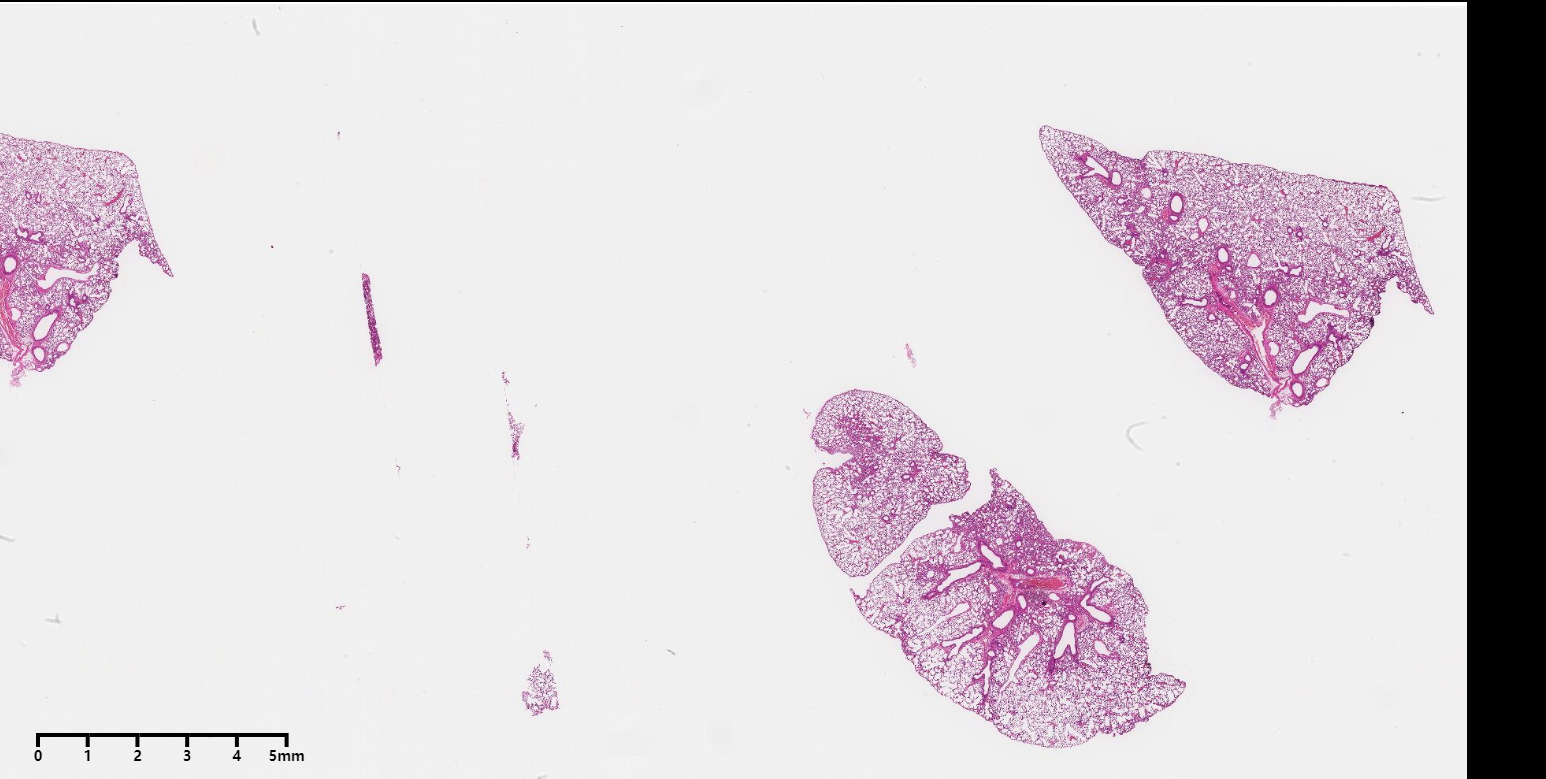

Supplement: Supplementary file 9 — EV Figure Source Data [file 44318_2024_237_MOESM9_ESM.zip › Figure EV3/Figure EV3I/SGC-CBP30 lungú¿810lpsú⌐_0.50X_20240705021650.tif]

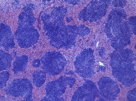

Supplement: Supplementary file 9 — EV Figure Source Data [file 44318_2024_237_MOESM9_ESM.zip › Figure EV3/Figure EV3I/SGC-CBP30 spleenú¿810 lpsú⌐.tif]

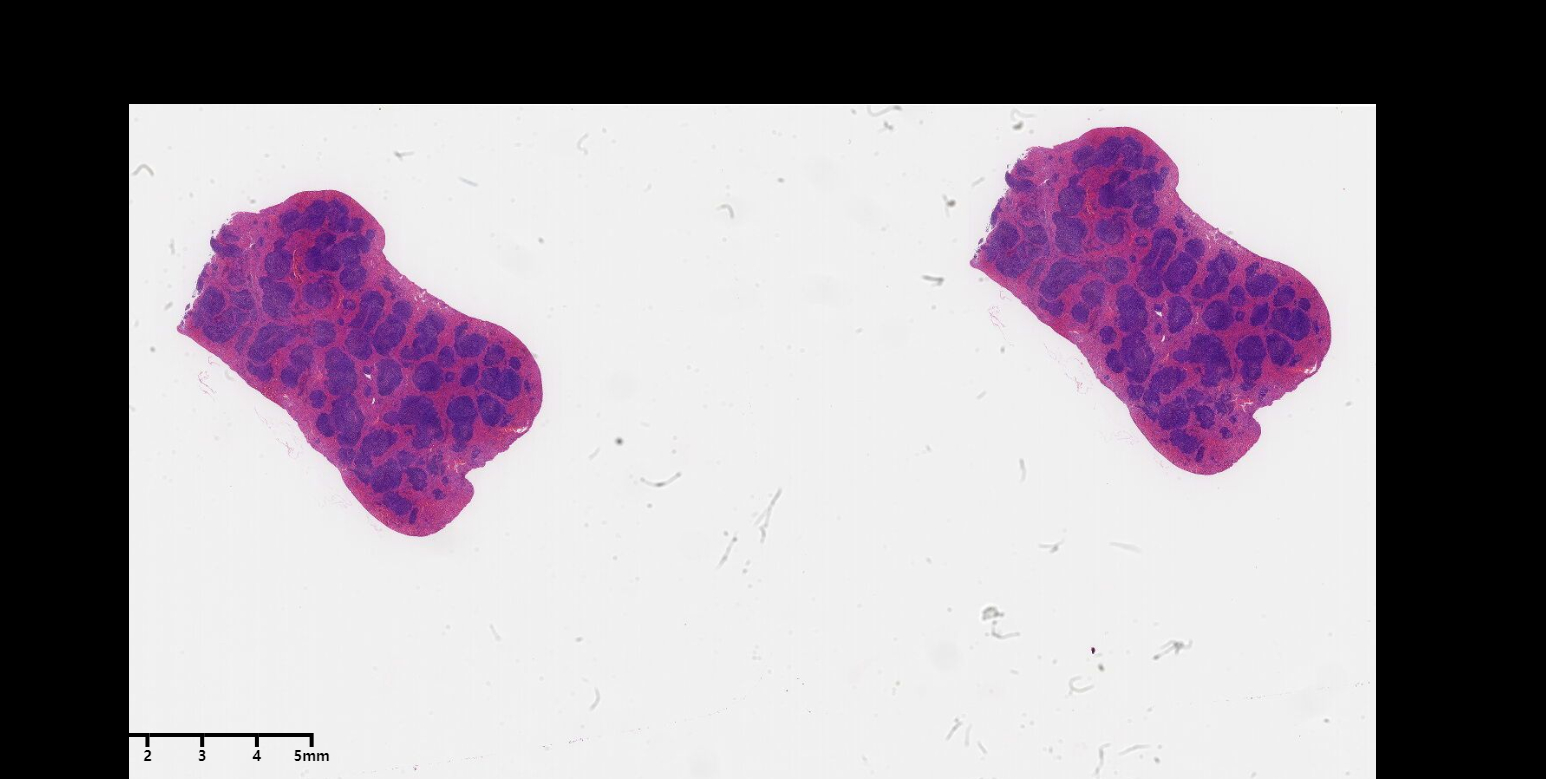

Supplement: Supplementary file 9 — EV Figure Source Data [file 44318_2024_237_MOESM9_ESM.zip › Figure EV3/Figure EV3I/SGC-CBP30 spleenú¿810lpsú⌐_0.50X_20240705021236.tif]

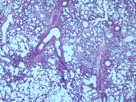

Supplement: Supplementary file 9 — EV Figure Source Data [file 44318_2024_237_MOESM9_ESM.zip › Figure EV3/Figure EV3I/TSA lungú¿810 lpsú⌐.tif]

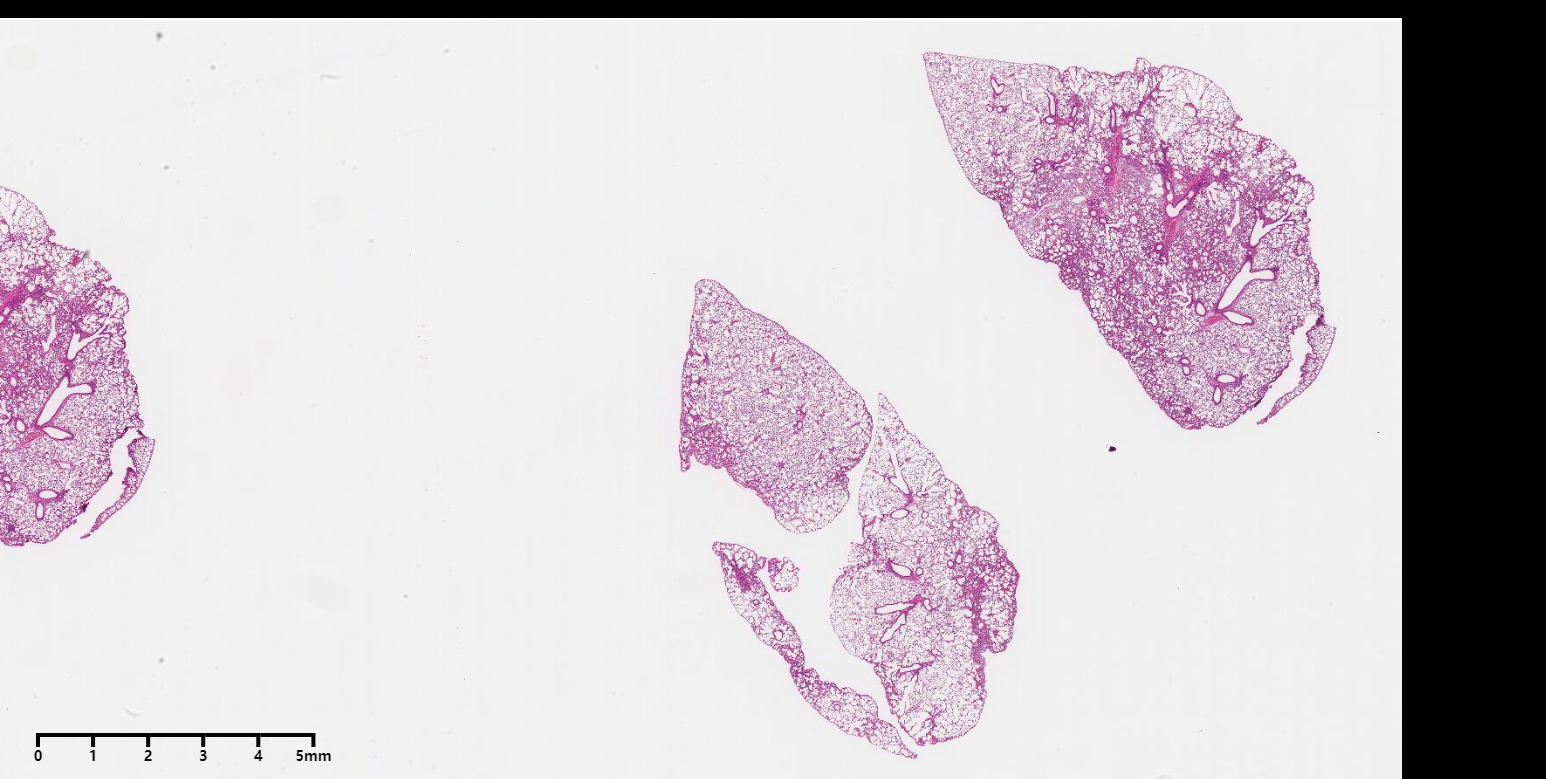

Supplement: Supplementary file 9 — EV Figure Source Data [file 44318_2024_237_MOESM9_ESM.zip › Figure EV3/Figure EV3I/TSA lungú¿810 lpsú⌐_0.50X_20240705021514.tif]
