# Supplementary figures and images for: Acetylation of TIR domains in the TLR4-Mal-MyD88 complex regulates immune responses in sepsis (part 3 of 3)
Source: EMBO J. 2024 Sep 18;43(21):9. doi: 10.1038/s44318-024-00237-8 (PMC11535217; doi:10.1038/s44318-024-00237-8)

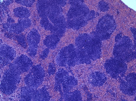

Supplement: Supplementary file 9 — EV Figure Source Data [file 44318_2024_237_MOESM9_ESM.zip › Figure EV3/Figure EV3I/TSA spleenú¿810 lpsú⌐.tif]

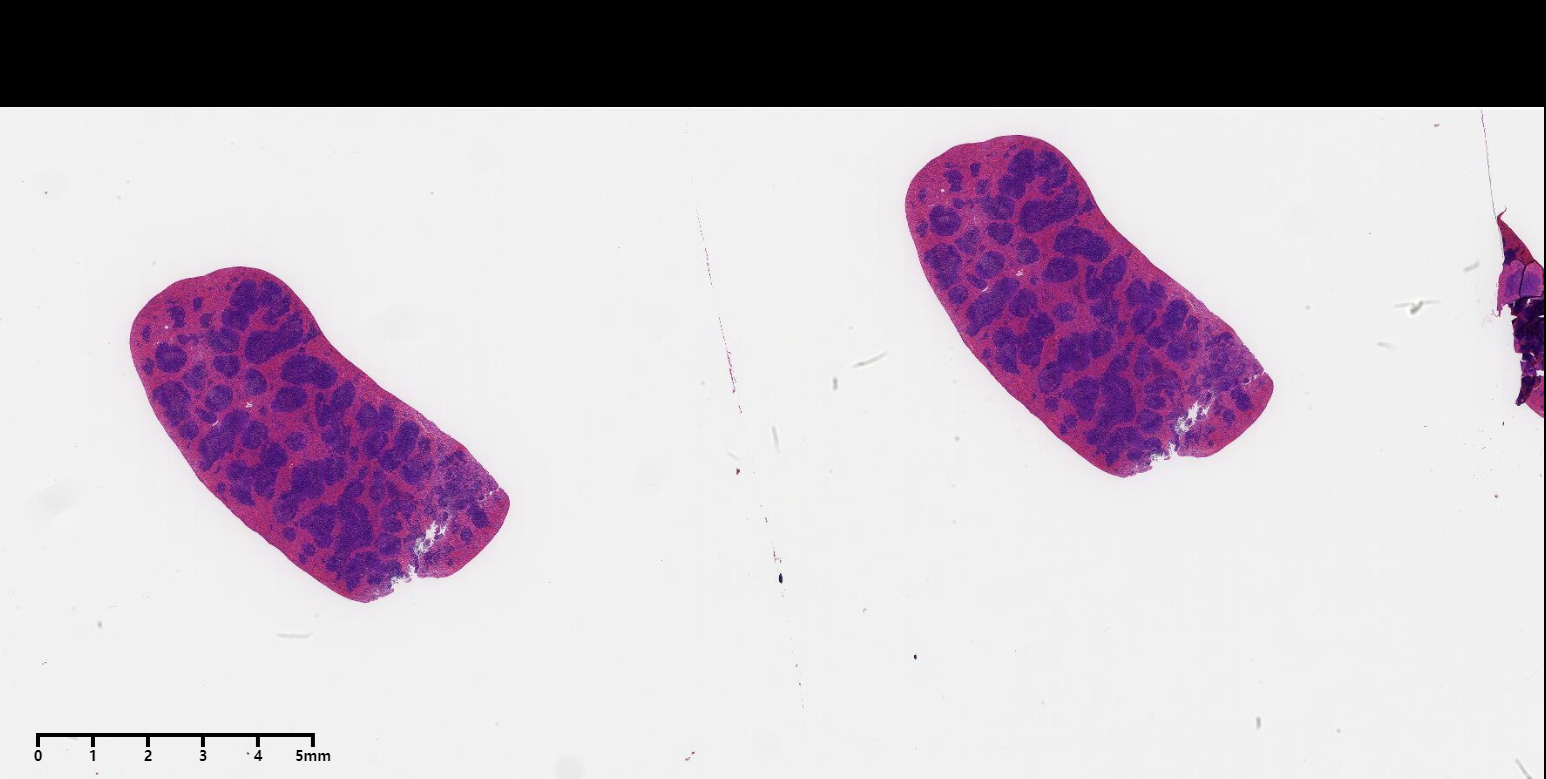

Supplement: Supplementary file 9 — EV Figure Source Data [file 44318_2024_237_MOESM9_ESM.zip › Figure EV3/Figure EV3I/TSA spleenú¿810lpsú⌐_0.50X_20240705021108.tif]
