# Supplementary material for: Acetylation of TIR domains in the TLR4-Mal-MyD88 complex regulates immune responses in sepsis
Source: EMBO J. 2024 Sep 18;43(21):9. doi: 10.1038/s44318-024-00237-8 (PMC11535217; doi:10.1038/s44318-024-00237-8)
Supplement: Supplementary file 10 — Expanded View Figures [file 44318_2024_237_MOESM10_ESM.pdf]

Expanded View Figures

Figure EV1. Cytosolic CBP induced by LPS promotes acetylation of the TIR domain complex.

(**A**) Macrophages derived from mouse bone marrow were exposed to 100 ng/mL LPS for 0, 5, 10, 15, 30, and 60 min. Subsequently, cells were immunoprecipitated with a TLR4 antibody, followed by western blot analysis with pan-acetyl-K and TLR4 antibodies. Whole cell lysate (WCL) was probed with an IκBα antibody. (**B**) The specificity of polyclonal antibodies targeting residues of TLR4-acK732 and TLR4-acK813 acetylation, using synthesized peptides as antigens, was assessed through dot blot analysis. (**C**) HEK293T cells were transfected with the indicated plasmids. Immunoprecipitation with Flag antibody was followed by western blot analysis with acK732 and acK813-TLR4 and Flag antibodies. (**D**) Macrophages derived from the bone marrow of TLR4-WT and TLR4-KR mice were treated with 100 ng/mL LPS for 30 min. Subsequently, the cells were immunoprecipitated with a TLR4 antibody, followed by western blot analysis using TLR4-acK732 and TLR4-acK813 antibodies. (**E**) THP-1 cells were induced into macrophages as described previously. Immunoblotting was performed with TLR4-acK813, TLR4-acK732, and TLR4 antibodies. Whole cell lysate (WCL) was probed with TLR4 and GAPDH antibody. (**F**) HEK293T cells were transfected with the indicated plasmids. Immunoprecipitation with Flag antibody was followed by western blot analysis with pan-acetyl-K and Flag antibodies. (**G**) HEK293T cells were transfected with Myc-TLR4-TIR and HA-CBP. Immunoprecipitation with Myc antibody was followed by western blot analysis with Myc-TLR4-TIR pan-acetyl-K and Myc antibodies. (**H**) Conservation analysis of the K732 and K813 sites in TLR family members. (**I**) Macrophages derived from mouse bone marrow were treated with 100 ng/mL LPS for 30 min. Immunoprecipitation with MyD88 antibody was followed by mass spectrometry analysis of MyD88 to identify acetylation residues, specifically acK231 and acK238, in the MyD88-TIR domain. (**J**) HEK293T cells were transfected with Flag-MAL and HA-CBP. Immunoprecipitation with Flag antibody was followed by mass spectrometry analysis of MAL to identify acetylation residues, specifically acK210 in the MAL-TIR domain.

© The Author(s)

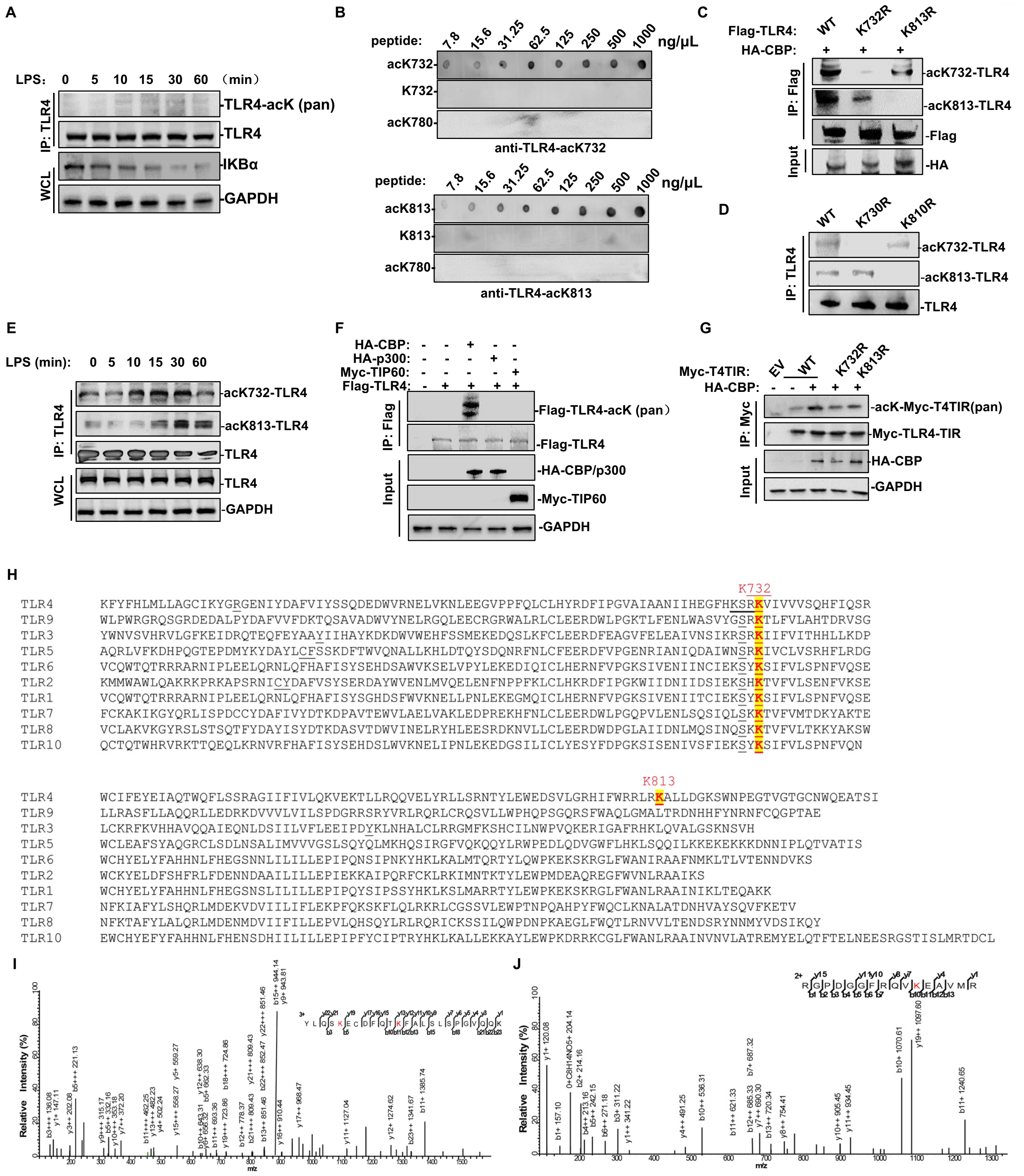
A
LPS: 0 5 10 15 30 60 (min)
IP: TLR4
WCL
TLR4-acK (pan)
TLR4
IKBα
GAPDH
B
peptide: 7.8 15.6 31.25 62.5 125 250 500 1000 ng/μL
acK732
K732
acK780
anti-TLR4-acK732
acK813
K813
acK780
anti-TLR4-acK813
C
Flag-TLR4: WT K732R K813R
HA-CBP: + + +
IP: Flag
Input
acK732-TLR4
acK813-TLR4
Flag
HA
D
WT K730R K810R
IP: TLR4
acK732-TLR4
acK813-TLR4
TLR4
E
LPS (min): 0 5 10 15 30 60
IP: TLR4
WCL
acK732-TLR4
acK813-TLR4
TLR4
TLR4
GAPDH
F
HA-CBP: - - + - -
HA-p300: - - - + -
Myc-TIP60: - - - - +
Flag-TLR4: - + + + +
IP: Flag
Input
Flag-TLR4-acK (pan)
Flag-TLR4
HA-CBP/p300
Myc-TIP60
GAPDH
G
Myc-T4TIR: EV WT K732R K813R
HA-CBP: - - + + +
IP: Myc
Input
acK-Myc-T4TIR(pan)
Myc-TLR4-TIR
HA-CBP
GAPDH
H
K732
TLR4 KFYFHLMLLAGCIKYGRGENIYDAFVIYSSQDEDWVRNELVKNLEEGVPPFQLCLHYRDFIPGVAIAANIIHEGFHKSRKVIVVVSQHFIQSR
TLR9 WLPWRGRQSGRDEDALPYDAFVVFDKTQSAVADWVYNELRGQLEECRGRWALRLCLEERDWLPGKTLFENLWASVYGSRKTLFVLAHTDRVSG
TLR3 YWNVSVHRVLGFKEIDRQTEQFEYAAYIIHAYKDKDWVWEHFSSMEKEDQSLKFCLEERDFEAGVFELEAIVNSIKRSRKIIFVITHHLLKDP
TLR5 AQRLVFKDHPQGTEPDMYKYDAYLCFSSKDFTWVQNALLKHLDTQYSDQNRFNLCFEERDFVPGENRIANIQDAIWNSRKIVCLVSRHFLRDG
TLR6 VCQWTQTRRRARNIPLEELQRNLQFHAFISYSEHDSAWVKSELVPYLEKEDIQICLHERNFVPGKSIVENIINCIEKSYKSIFVLSPNFVQSE
TLR2 KMMWAWLQAKRKPRKAPSRNICYDAFVSYSERDAYWVENLMVQELENFNPPFKLCLHKRDFIPGKWIIDNIIDSIEKSHKTVFVLSENFVKSE
TLR1 VCQWTQTRRRARNIPLEELQRNLQFHAFISYSGHDSFWVKNELLPNLEKEGMQICLHERNFVPGKSIVENIITCIEKSYKSIFVLSPNFVQSE
TLR7 FCKAKIKGYQRLISPDCCYDAFIVYDTKDPAVTEWVLAELVAKLEDPREKHFNLCLEERDWLPGQPVLENLSQSIQLSKKTVFVMTDKYAKTE
TLR8 VCLAKVKGYRSLSTSQTFYDAYISYDTKDASVTDWVINELRYHLEESRDKNVLLCLEERDWDPGLAIIDNLMQSINQSKKTVFVLTKKYAKSW
TLR10 QCTQTWHRVRKTTQEQLKRNVRFHAFISYSEHDSLWVKNELIPNLEKEDGSILICLYESYFDPGKSISENIVSFIEKSYKSIFVLSPNFVQN
K813
TLR4 WCIFEYEIAQTWQFLSSRAGIIFIVLQKVEKTLLRQQVELYRLLSRNTYLEWEDSVLGRHIFWRRLRKALLDGKSWNPEGTVGTGCNWQEATSI
TLR9 LLRASFLLAQQRLLEDRKDVVVLVILSPDGRRSRYVRLRQRLCRQSVLLWPHQPSGQRSFWAQLGMALTRDNHHFYNRNFCQGPTAE
TLR3 LCKRFKVHHAVQQAIEQNLDSIILVFLEEIPDYKLNHALCLRRGMFKSHCILNWPVQKERIGAFRHKLQVALGSKNSVH
TLR5 WCLEAFSYAQGRCLSDLNSALIMVVVGSLSQYQLMKHQSIRGFVQKQQYLRWPEDLQDVGWFLHKLSQQILKKEKEKKKDNNIPLQTVATIS
TLR6 WCHYELYFAHHNLFHEGSNNLILILLEPIPQNSIPNKYHKLKALMTQRTYLQWPKEKSKRGLFWANIRAAFNMKLTLVTENNDVKS
TLR2 WCKYELDFSHFRLFDENNDAAILILLEPIEKKAIPQRFCKLRKIMNTKTYLEWPMDEAQREGFWVNLRAAIKS
TLR1 WCHYELYFAHHNLFHEGSNSLILILLEPIPQYSIPSSYHKLKSLMARRTYLEWPKEKSKRGLFWANLRAAINIKLTEQAKK
TLR7 NFKIAFYLSHQRLMDEKVDVIILIFLEKPFQKSKFLQLRKRLCGSSVLEWPTNPQAHPYFWQCLKNALATDNHVAYSQVFKETV
TLR8 NFKTAFYLALQRLMDENMDVIIFILLEPVLQHSQYLRLRQRICKSSILQWPDNPKAEGLFWQTLRNVVLTENDSRYNNMYVDSIKQY
TLR10 EWCHYEFYFAHHNLFHENSDHIILILLEPIPFYCIPTRYHKLKALLEKKAYLEWPKDRRKCGLFWANLRAAINVNVLATREMYELQTFTELNEESRGSTISLMRTDCL
I
Relative Intensity (%)
m/z
J
Relative Intensity (%)
m/z

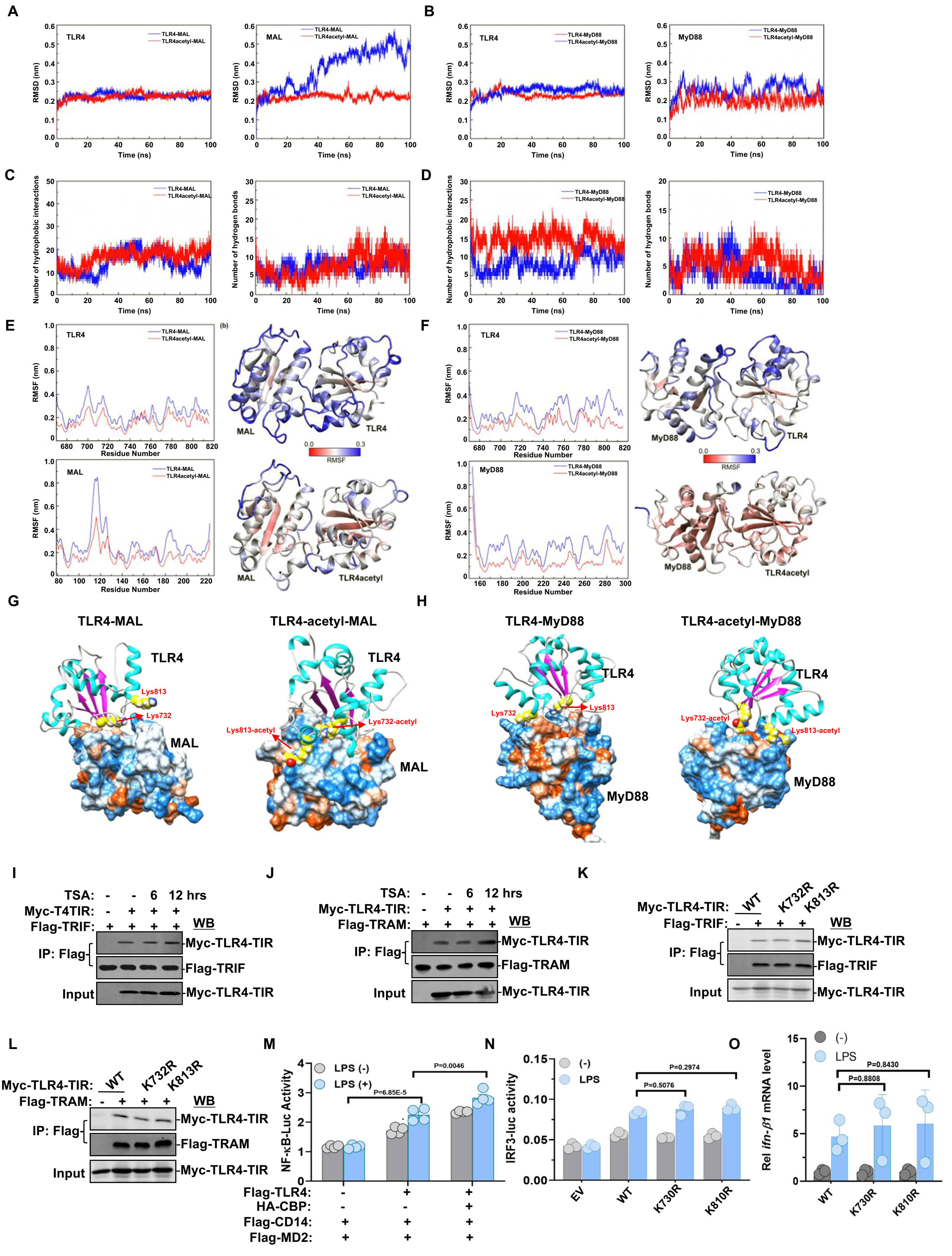
A
B
C
D
E
F
G
H
I
J
K
L
M
N
O
TLR4-MAL
TLR4-acetyl-MAL
TLR4-MyD88
TLR4-acetyl-MyD88
TLR4
MAL
MyD88
Lys813
Lys732
Lys813-acetyl
Lys732-acetyl
RMSD (nm)
Time (ns)
Number of hydrophobic interactions
Number of hydrogen bonds
RMSF (nm)
Residue Number
TLR4acetyl
TSA: - - 6 12 hrs
Myc-T4TIR: - + + +
Flag-TRIF: + + + +
Myc-TLR4-TIR: - + + +
Flag-TRAM: + + + +
Myc-TLR4-TIR: WT K732R K813R
WB
IP: Flag
Input
Myc-TLR4-TIR
Flag-TRIF
Flag-TRAM
LPS (-)
LPS (+)
P=6.85E-5
P=0.0046
NF-κB-Luc Activity
Flag-TLR4: - + +
HA-CBP: - - +
Flag-CD14: + + +
Flag-MD2: + + +
(-)
LPS
P=0.5076
P=0.2974
IRF3-luc activity
EV
WT
K730R
K810R
P=0.8808
P=0.8430
Rel ifn-β1 mRNA level

Figure EV2. Molecular simulation reveals that acetylated TLR4 enhances its binding affinity with adaptors MAL and MyD88.

(**A**) The root mean square deviation (RMSD) of skeleton atoms in the TLR4–MAL and TLR4-acetyl-MAL systems during MD simulation (**B**) The root mean square deviation (RMSD) of skeleton atoms in the TLR4-MyD88 and TLR4-acetyl-MyD88 systems during MD simulation. (**C**) The number of hydrophobic interactions and hydrogen bonds formed between MAL and non-acetylated or K732/K813-acetylated TLR4 during molecular simulation. (**D**) The number of hydrophobic interactions and hydrogen bonds formed between MyD88 and non-acetylated or K732/K813-acetylated TLR4 during molecular simulation. (**E**) The root mean square fluctuation (RMSF) distribution of residues in the TLR4–MAL and TLR4-acetyl-MAL systems and the distribution of RMSF on protein secondary structure. (**F**) The root mean square fluctuation (RMSF) distribution of residues in the TLR4-MyD88 and TLR4-acetyl-MyD88 systems and the distribution of RMSF on protein secondary structure. (**G**) The interaction pattern between MAL and non-acetylated or K732/K813-acetylated TLR4 on the hydrophilic and hydrophobic surfaces after MD simulation (blue and orange regions on the protein surface represent hydrophilic and hydrophobic regions, respectively). (**H**) The interaction pattern between MyD88 and non-acetylated or K732/K813-acetylated TLR4 on the hydrophilic and hydrophobic surfaces after MD simulation (blue and orange regions on the protein surface represent hydrophilic and hydrophobic regions, respectively). (**I**, **J**) HEK293T cells were transfected with the indicated plasmids and then treated with 1 μM TSA for 6 h and 12 h. Cells were subjected to immunoprecipitation with Flag antibody followed by western blot with Myc and Flag antibodies. TSA treatment slightly enhances the interaction of TLR4-TIR with TRIF or TRAM. (**K**, **L**) TLR4-WT or TLR4-K732R or K813R mutation was co-transfected with TRIF or TRAM in HEK293T cells. Neither the K732R nor K813R mutant could abolish the interaction of TLR4-TIR with TRIF or TRAM. (**M**) The effect of TLR4 acetylation on NF-κB transcriptional activity regulation was tested in HEK293T cells. Cells were transfected with the indicated plasmids and NF-κB luciferase reporter for 42 h, and then treated with 1000 ng/ml LPS for another 6 h ($n = 4$). (**N**) The effect of TLR4 acetylation on IRF3 transcriptional activity regulation was tested in HeLa cells. Cells were transfected with the indicated plasmids and IRF3 luciferase reporter for 42 h, and then treated with 1000 ng/mL LPS for another 6 h ($n = 3$). (**O**) Quantitative qRT-PCR validation of IFN-β1 in M1 macrophages from TLR4-WT or TLR4-KR mutant mice bone marrow treated with or without LPS ($n = 3$). One-way ANOVA. Error bars, s.e.m.

 © The Author(s)

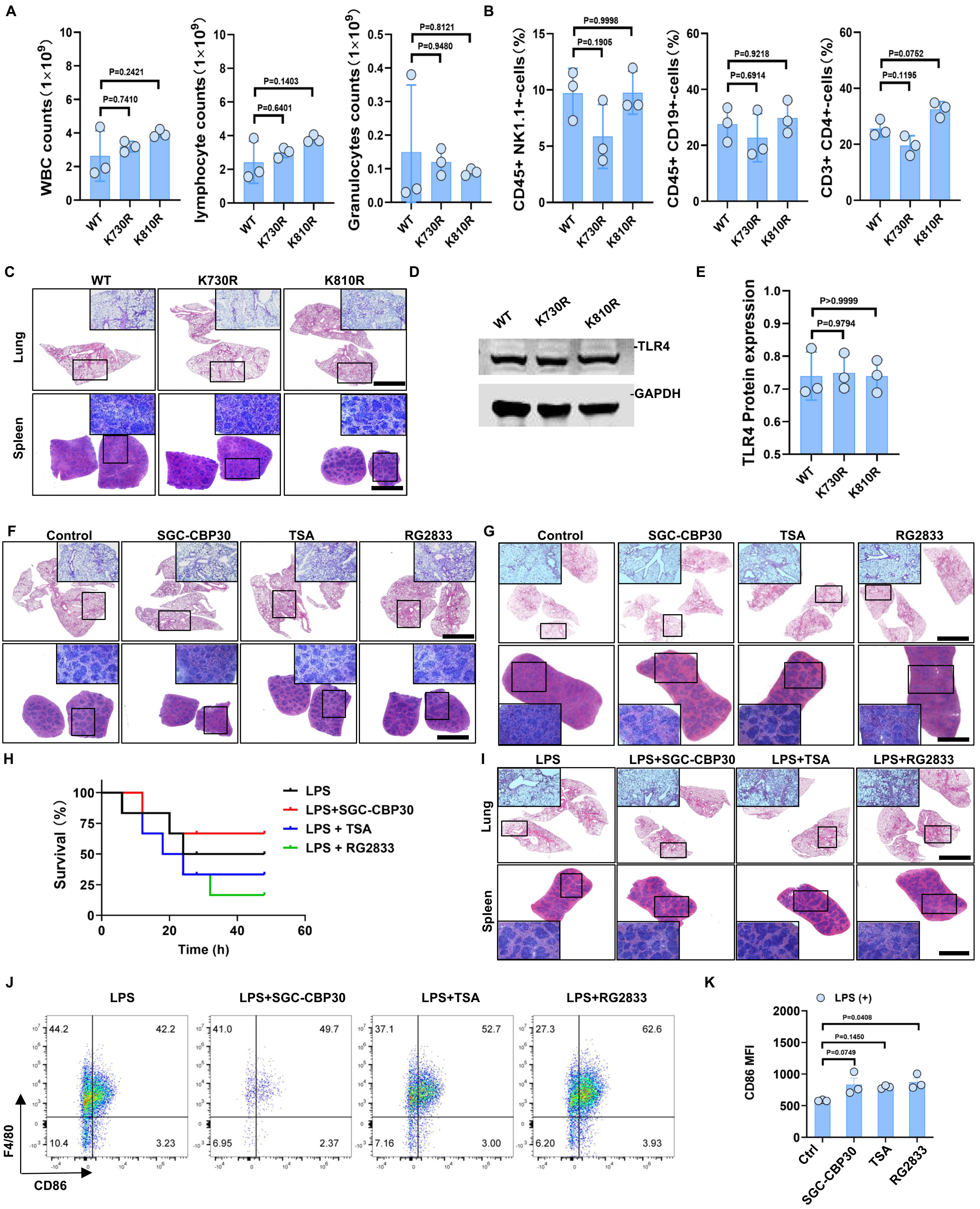
A
WBC counts (1×10⁹)
lymphocyte counts (1×10⁹)
Granulocytes counts (1×10⁹)
P=0.2421
P=0.7410
P=0.1403
P=0.6401
P=0.8121
P=0.9480
WT
K730R
K810R
B
CD45+ NK1.1+-cells (%)
CD45+ CD19+-cells (%)
CD3+ CD4+-cells (%)
P=0.9998
P=0.1905
P=0.9218
P=0.6914
P=0.0752
P=0.1195
C
Lung
Spleen
D
TLR4
GAPDH
E
TLR4 Protein expression
P>0.9999
P=0.9794
F
Control
SGC-CBP30
TSA
RG2833
G
H
Survival (%)
Time (h)
LPS
LPS+SGC-CBP30
LPS + TSA
LPS + RG2833
I
LPS+TSA
LPS+RG2833
J
F4/80
CD86
44.2
42.2
10.4
3.23
41.0
49.7
6.95
2.37
37.1
52.7
7.16
3.00
27.3
62.6
6.20
3.93
K
LPS (+)
CD86 MFI
P=0.0408
P=0.1450
P=0.0749
Ctrl

Figure EV3. Detection of WBC in TLR4-KR mutant mice and therapy in sepsis of TLR4-K810R mice.

(**A**) Blood routine analysis of white blood cells, lymphocytes, and granulocytes in TLR4-WT or TLR4-KR mutant mice blood ($n = 3$). (**B**) Splenic cells obtained from TLR4-WT or TLR4-KR mutant mice. Cells were collected for flow cytometer analysis to detect the polarization of NK, B, $CD4^{+}$T cells ($n = 3$). (**C**) Representative H&E staining images of lung and spleen tissues isolated from TLR4-WT or TLR4-KR mutant mice. Scale bar = 2 mm. (**D**, **E**) Macrophages derived from TLR4-WT or TLR4-KR mutant mice bone marrow and subjected to Western blot analysis with TLR4 antibody (**D**). Densitometry measurements were performed using ImageJ (**E**). Data shown are representative of three independent experiments. (**F**) Representative H&E staining images of lung and spleen tissues isolated from TLR4-WT mice subjected to various treatments. The scale bar = 2 mm. (**G**) Representative H&E staining images of lung and spleen tissues isolated from TLR4-K810R mice subjected to various treatments.The scale bar =2 mm. (**H**) Survival curves of TLR4-K810R mice subjected to different treatments after intraperitoneal injection with 20 mg/kg LPS ($n = 6$). (**I**) Representative H&E staining images of lung and spleen tissues isolated from TLR4-K810R mice subjected to various treatments after intraperitoneal injection with 20 mg/kg LPS for 12 h. The scale bar = 2 mm. (**J**, **K**) Peritoneal macrophages from TLR4- K810R mice were subjected to different treatments after intraperitoneal injection with 20 mg/kg LPS for 12 h. Cells were collected for flow cytometer analysis to detect the polarization of M1 macrophages using anti-CD45, anti-F4/80 and anti-CD86 fluorescence antibodies ($n = 3$). One-way ANOVA. Log-rank test. Error bars, s.e.m. Source data are available online for this figure.

 © The Author(s)

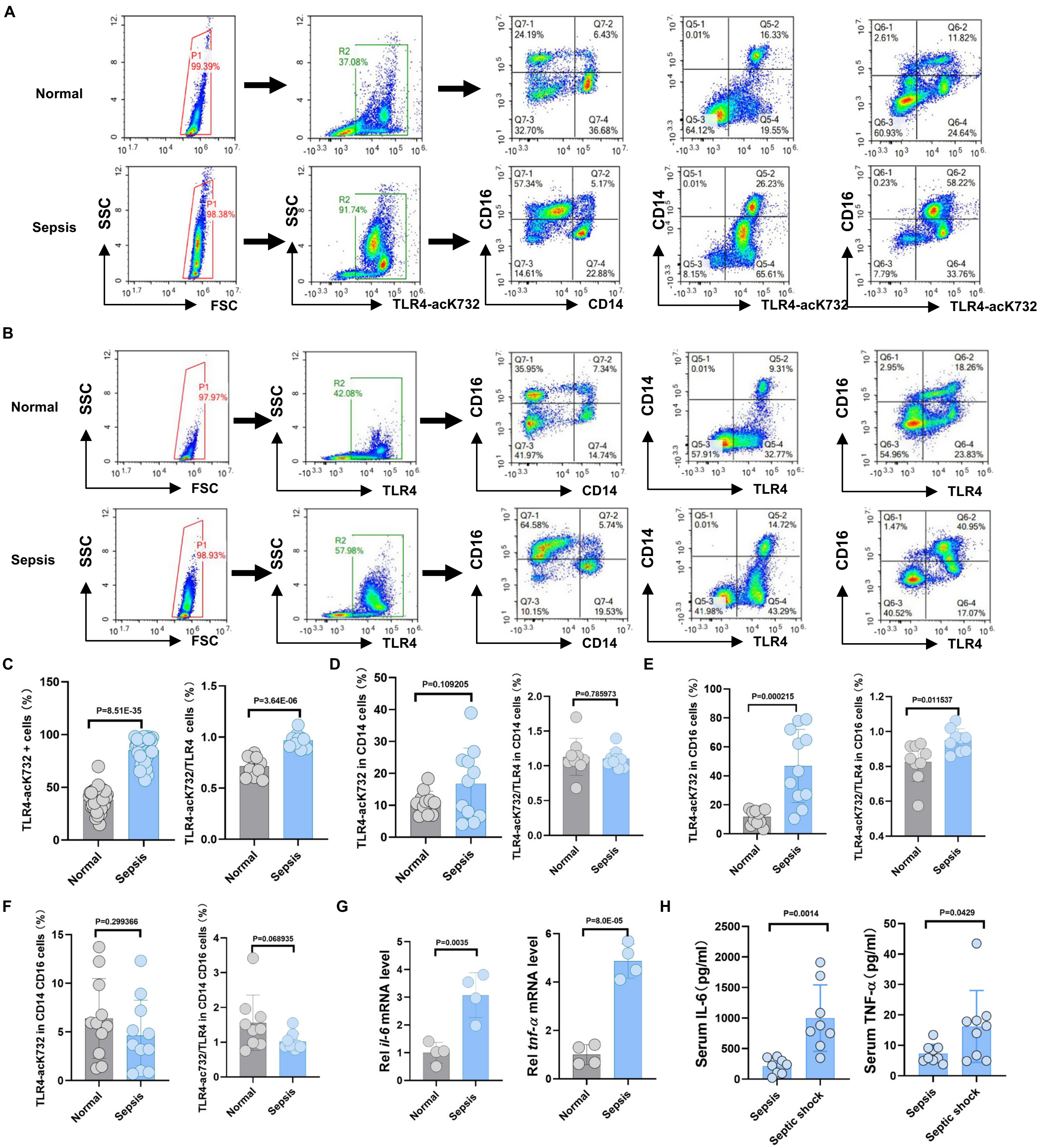
A
Normal
Sepsis
SSC
FSC
P1 99.39%
P1 98.38%
R2 37.08%
R2 91.74%
TLR4-acK732
CD16
CD14
Q7-1 24.19%
Q7-2 6.43%
Q7-3 32.70%
Q7-4 36.68%
Q5-1 0.01%
Q5-2 16.33%
Q5-3 64.12%
Q5-4 19.55%
Q6-1 2.61%
Q6-2 11.82%
Q6-3 60.93%
Q6-4 24.64%
Q7-1 57.34%
Q7-2 5.17%
Q7-3 14.61%
Q7-4 22.88%
Q5-1 0.01%
Q5-2 26.23%
Q5-3 8.15%
Q5-4 65.61%
Q6-1 0.23%
Q6-2 58.22%
Q6-3 7.79%
Q6-4 33.76%
B
Normal
Sepsis
SSC
FSC
TLR4
CD16
CD14
P1 97.97%
P1 98.93%
R2 42.08%
R2 57.98%
Q7-1 35.95%
Q7-2 7.34%
Q7-3 41.97%
Q7-4 14.74%
Q5-1 0.01%
Q5-2 9.31%
Q5-3 57.91%
Q5-4 32.77%
Q6-1 2.95%
Q6-2 18.26%
Q6-3 54.96%
Q6-4 23.83%
Q7-1 64.58%
Q7-2 5.74%
Q7-3 10.15%
Q7-4 19.53%
Q5-1 0.01%
Q5-2 14.72%
Q5-3 41.98%
Q5-4 43.29%
Q6-1 1.47%
Q6-2 40.95%
Q6-3 40.52%
Q6-4 17.07%
C
TLR4-acK732 + cells (%)
P=8.51E-35
TLR4-acK732/TLR4 cells (%)
P=3.64E-06
Normal
Sepsis
D
TLR4-acK732 in CD14 cells (%)
P=0.109205
TLR4-acK732/TLR4 in CD14 cells (%)
P=0.785973
E
TLR4-acK732 in CD16 cells (%)
P=0.000215
TLR4-acK732/TLR4 in CD16 cells (%)
P=0.011537
F
TLR4-acK732 in CD14 CD16 cells (%)
P=0.299366
TLR4-ac732/TLR4 in CD14 CD16 cells (%)
P=0.068935
G
Rel il-6 mRNA level
P=0.0035
Rel tnf-α mRNA level
P=8.0E-05
H
Serum IL-6 (pg/ml)
P=0.0014
Serum TNF-α (pg/ml)
P=0.0429
Sepsis
Septic shock

Figure EV4. TLR4-acK732 and TLR4 expression in monocytes from sepsis patients.

(**A**, **B**) Monocytes were collected for flow cytometry analysis to detect the polarization of TLR4-acK732 (**A**) and TLR4 (**B**) in CD14 + , CD16 + , and CD14 + CD16+ cell types. (**C**) Monocytes were obtained for flow cytometry analysis to detect the ratio of TLR4-acK732 ($n = 43$) and TLR4-acK732/TLR4 ($n = 10$) in normal and sepsis patients. (**D**) Monocytes were obtained for flow cytometry analysis to detect the ratio of TLR4-acK732 ($n = 11$) and TLR4-acK732/TLR4 ($n = 9$) in normal and sepsis patients' CD14+ cell type. (**E**) Monocytes were obtained for flow cytometry analysis to detect the ratio of TLR4-acK732 ($n = 11$) and TLR4-acK732/TLR4 ($n = 9$) in normal and sepsis patients' CD16+ cell type. (**F**) Monocytes were obtained for flow cytometry analysis to detect the ratio of TLR4-acK732 ($n = 11$) and TLR4-acK732/TLR4 ($n = 9$) in normal and sepsis patients' CD14 + CD16+ cell type. (**G**) Mononuclear cells were obtained by sorting with TLR4-acK and CD16 antibodies from both normal individuals and sepsis patients. The expression of IL-6 and TNF-α in these cells was then detected using real-time PCR ($n = 4$). (**H**) Serum IL-6 and TNF-α levels in patients with sepsis and septic shock ($n = 8$). Student's *t* test. Error bars, s.e.m.

 © The Author(s)

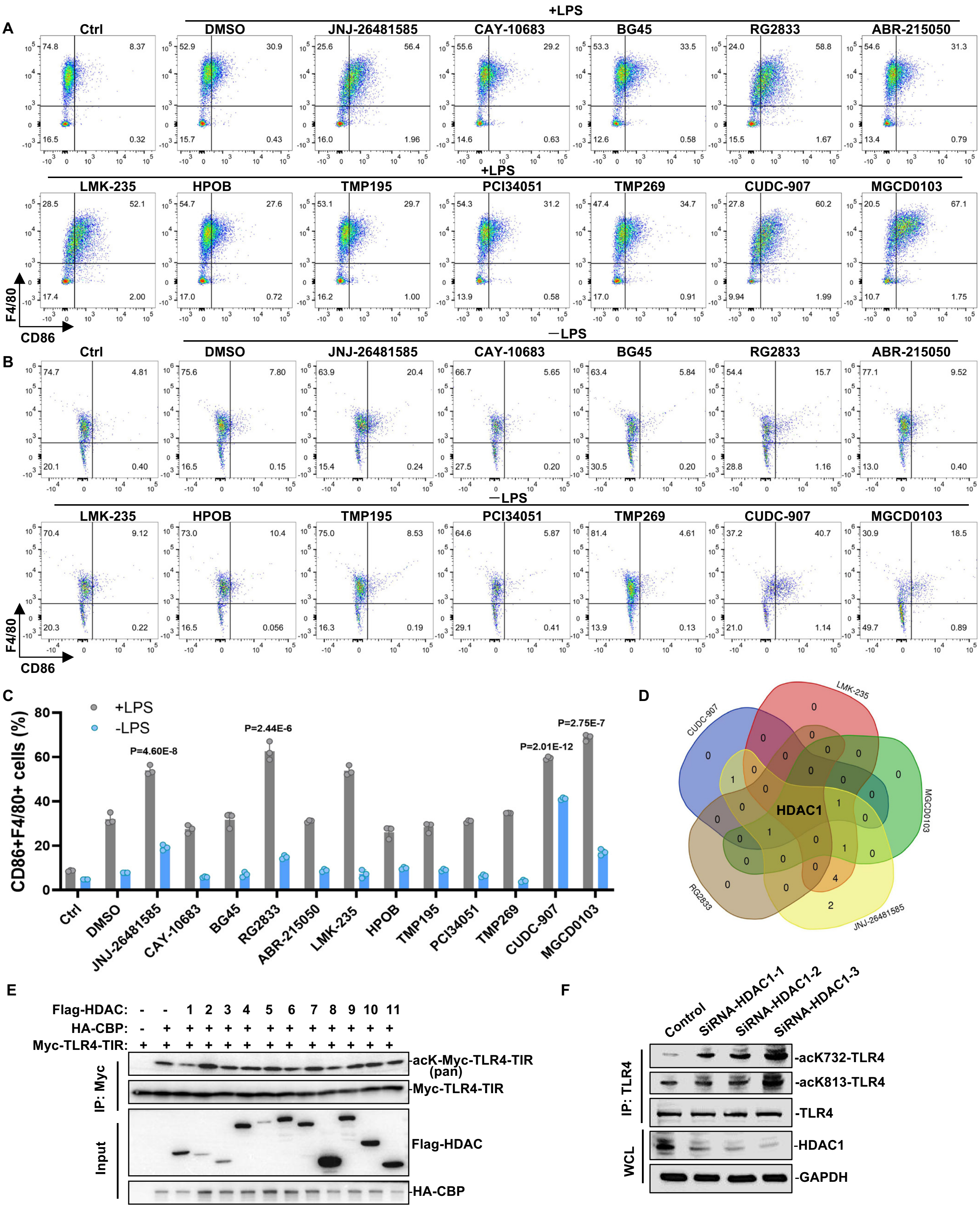
A
+LPS
Ctrl
DMSO
JNJ-26481585
CAY-10683
BG45
RG2833
ABR-215050
LMK-235
HPOB
TMP195
PCI34051
TMP269
CUDC-907
MGCD0103
F4/80
CD86
B
−LPS
C
CD86+F4/80+ cells (%)
+LPS
-LPS
P=4.60E-8
P=2.44E-6
P=2.01E-12
P=2.75E-7
D
HDAC1
E
Flag-HDAC:
HA-CBP:
Myc-TLR4-TIR:
IP: Myc
Input
-acK-Myc-TLR4-TIR (pan)
-Myc-TLR4-TIR
Flag-HDAC
-HA-CBP
F
Control
SiRNA-HDAC1-1
SiRNA-HDAC1-2
SiRNA-HDAC1-3
IP: TLR4
WCL
-acK732-TLR4
-acK813-TLR4
-TLR4
-HDAC1
-GAPDH

Figure EV5. HDAC1 mediated TLR4-TIR deacetylation.

(**A**, **B**) Macrophages derived from TLR4-WT mice bone marrow, as described before, were pretreated with a series of HDAC family inhibitors for 6 h and then treated with (**A**) or without (**B**) 100 ng/mL LPS for 12 h. Cells were collected for flow cytometry analysis to detect the expression of M1 macrophages by staining with anti-F4/80 (macrophage marker) and anti-CD86 (M1 marker) fluorescence antibodies. (**C**) Statistical analysis in the expression of M1 macrophages treated with or without LPS ($n = 3$). (**D**) Venn diagram analysis illustrating HDAC family members targeted by inhibitors in the expression of M1 macrophages. (**E**) HEK293T cells were transfected with Myc-TLR4-TIR, HA-CBP, and Flag-HDAC family plasmids. Cells were subjected to immunoprecipitation with Myc antibody followed by western blot with pan-acetyl-K, Flag, HA, and Myc antibodies. The TLR4-TIR domain was efficiently deacetylated by HDAC1. (**F**) THP-1 cells were induced into macrophages and then were transfected with siRNA for 36 h, followed by 100 ng/ml LPS treatment for 30 min. Cells were subjected to immunoprecipitation with TLR4 antibody followed by western blot with acK732-TLR4、acK813-TLR4 antibodies. One-way ANOVA. Error bars, s.e.m.

 © The Author(s)
